# Supplementary material for: Systematic estimates of the global, regional and national under-5 mortality burden attributable to birth defects in 2000–2019: a summary of findings from the 2020 WHO estimates
Source: BMJ Open. 2023 Jan 30;13(1):e067033. doi: 10.1136/bmjopen-2022-067033 (PMC9887698; doi:10.1136/bmjopen-2022-067033)
Supplement: Supplementary data [file bmjopen-2022-067033supp001.pdf]

Annex 1. WHO Member States by Region

African Region
Angola, Burundi, Benin, Burkina Faso, Botswana, Central African Republic, Cote d'Ivoire, Cameroon, Democratic Republic of the Congo, Congo, Comoros, Cabo Verde, Algeria, Eritrea, Ethiopia, Gabon, Ghana, Guinea, Gambia, Guinea-Bissau, Equatorial Guinea, Kenya, Liberia, Lesotho, Madagascar, Mali, Mozambique, Mauritania, Mauritius, Malawi, Namibia, Niger, Nigeria, Rwanda, Senegal, Sierra Leone, South Sudan, Sao Tome and Principe, Eswatini, Seychelles, Chad, Togo, United Republic of Tanzania, Uganda, South Africa, Zambia, Zimbabwe

Region of the Americas
Argentina, Antigua and Barbuda, Bahamas, Belize, Bolivia (Plurinational State of), Brazil, Barbados, Canada, Chile, Colombia, Costa Rica, Cuba, Dominica, Dominican Republic, Ecuador, Grenada, Guatemala, Guyana, Honduras, Haiti, Jamaica, Saint Kitts and Nevis, Saint Lucia, Mexico, Nicaragua, Panama, Peru, Paraguay, El Salvador, Suriname, Trinidad and Tobago, Uruguay, United States of America, Saint Vincent and the Grenadines, Venezuela (Bolivarian Republic of)

Eastern Mediterranean Region
Afghanistan, United Arab Emirates, Bahrain, Djibouti, Egypt, Iran (Islamic Republic of), Iraq, Jordan, Kuwait, Lebanon, Libya, Morocco, Oman, Pakistan, Qatar, Saudi Arabia, Sudan, Somalia, Syrian Arab Republic, Tunisia, Yemen

European Region
Albania, Andorra, Armenia, Austria, Azerbaijan, Belgium, Bulgaria, Bosnia and Herzegovina, Belarus, Switzerland, Cyprus, Czechia, Germany, Denmark, Spain, Estonia, Finland, France, United Kingdom, Georgia, Greece, Croatia, Hungary, Ireland, Iceland, Israel, Italy, Kazakhstan, Kyrgyzstan, Lithuania, Luxembourg, Latvia, Monaco, Republic of Moldova, Macedonia, Malta, Montenegro, Netherlands, Norway, Poland, Portugal, Romania, Russian Federation, San Marino, Serbia, Slovakia, Slovenia, Sweden, Tajikistan, Turkmenistan, Turkey, Ukraine, Uzbekistan

South East Asian Region
Bangladesh, Bhutan, Indonesia, India, Sri Lanka, Maldives, Myanmar, Nepal, Democratic People's Republic of Korea, Thailand, Timor-Leste

Western Pacific Region
Australia, Brunei Darussalam, China, Cook Islands, Fiji, Micronesia (Federated States of), Japan, Cambodia, Kiribati, Republic of Korea, Lao People's Democratic Republic, Marshall Islands, Mongolia, Malaysia, Niue, Nauru, New Zealand, Philippines, Palau, Papua New Guinea, Singapore, Solomon Islands, Tonga, Tuvalu, Viet Nam, Vanuatu, Samoa

Annex 2. WHO Member States by Income

High Income

Andorra, Australia, Austria, Bahamas, Belgium, Brunei Darussalam, Canada, Croatia, Cyprus, Czechia, Denmark, Estonia, Finland, France, Germany, Greece, Hungary, Iceland, Ireland, Israel, Italy, Japan, Latvia, Lithuania, Luxembourg, Malta, Monaco, Netherlands, New Zealand, Norway, Poland, Portugal, Republic of Korea, San Marino, Singapore, Slovakia, Slovenia, Spain, Sweden, Switzerland, United Kingdom, United States of America

Low and Middle Income

Afghanistan, Albania, Algeria, Angola, Antigua and Barbuda, Argentina, Armenia, Azerbaijan, Bahrain, Bangladesh, Barbados, Belarus, Belize, Benin, Bhutan, Bolivia (Plurinational State of), Bosnia and Herzegovina, Botswana, Brazil, Bulgaria, Burkina Faso, Burundi, Cote d'Ivoire, Cabo Verde, Cambodia, Cameroon, Central African Republic, Chad, Chile, China, Colombia, Comoros, Congo, Cook Islands, Costa Rica, Cuba, Democratic People's Republic of Korea, Democratic Republic of the Congo, Djibouti, Dominica, Dominican Republic, Ecuador, Egypt, El Salvador, Equatorial Guinea, Eritrea, Eswatini, Ethiopia, Fiji, Gabon, Gambia, Georgia, Ghana, Grenada, Guatemala, Guinea, Guinea-Bissau, Guyana, Haiti, Honduras, India, Indonesia, Iran (Islamic Republic of), Iraq, Jamaica, Jordan, Kazakhstan, Kenya, Kiribati, Kuwait, Kyrgyzstan, Lao People's Democratic Republic, Lebanon, Lesotho, Liberia, Libya, Madagascar, Malawi, Malaysia, Maldives, Mali, Marshall Islands, Mauritania, Mauritius, Mexico, Micronesia (Federated States of), Mongolia, Montenegro, Morocco, Mozambique, Myanmar, Namibia, Nauru, Nepal, Nicaragua, Niger, Nigeria, Niue, Oman, Pakistan, Palau, Panama, Papua New Guinea, Paraguay, Peru, Philippines, Qatar, Republic of Moldova, Republic of North Macedonia, Romania, Russian Federation, Rwanda, Saint Kitts and Nevis, Saint Lucia, Saint Vincent and the Grenadines, Samoa, Sao Tome and Principe, Saudi Arabia, Senegal, Serbia, Seychelles, Sierra Leone, Solomon Islands, Somalia, South Africa, South Sudan, Sri Lanka, Sudan, Suriname, Syrian Arab Republic, Tajikistan, Thailand, Timor-Leste, Togo, Tonga, Trinidad and Tobago, Tunisia, Turkey, Turkmenistan, Tuvalu, Uganda, Ukraine, United Arab Emirates, United Republic of Tanzania, Uruguay, Uzbekistan, Vanuatu, Venezuela (Bolivarian Republic of), Viet Nam, Yemen, Zambia, Zimbabwe
