# Supplementary material for: Systematic estimates of the global, regional and national under-5 mortality burden attributable to birth defects in 2000–2019: a summary of findings from the 2020 WHO estimates
Source: BMJ Open. 2023 Jan 30;13(1):e067033. doi: 10.1136/bmjopen-2022-067033 (PMC9887698; doi:10.1136/bmjopen-2022-067033)
Supplement: Supplementary data [file bmjopen-2022-067033supp002.pdf]

iso3	year	level	whoreg6	whoname	nnd	pnd	u5d	nmr	pnmr	u5mr	Livebirths	Neonatal birth defects deaths	1-59 month birth defects deaths	Under five birth defects deaths
AFG	2000	country	5_Emr	Afghanistan	62013	68593	130606	0.0606273	0.06706	0.1276872	1022857	1633	348	1981
AFG	2001	country	5_Emr	Afghanistan	62301	72136	134437	0.0594359	0.0688187	0.1282545	1048206	1712	476	2188
AFG	2002	country	5_Emr	Afghanistan	62343	64259	126602	0.0582014	0.0599898	0.1181913	1071159	1780	478	2258
AFG	2003	country	5_Emr	Afghanistan	62210	56611	118821	0.0569443	0.0518196	0.108764	1092470	1895	559	2454
AFG	2004	country	5_Emr	Afghanistan	61887	55547	117434	0.0556444	0.0499438	0.1055882	1112187	1964	733	2698
AFG	2005	country	5_Emr	Afghanistan	61321	55845	117166	0.0542683	0.0494224	0.1036907	1129961	2035	803	2838
AFG	2006	country	5_Emr	Afghanistan	60571	59132	119703	0.0528926	0.0516361	0.1045287	1145170	2106	891	2997
AFG	2007	country	5_Emr	Afghanistan	59600	52198	111798	0.0514964	0.0451004	0.0965968	1157363	2145	967	3112
AFG	2008	country	5_Emr	Afghanistan	58432	50335	108767	0.0500951	0.0431535	0.0932486	1166422	2173	1105	3278
AFG	2009	country	5_Emr	Afghanistan	57037	50752	107789	0.0486373	0.0432776	0.0919149	1172701	2213	1182	3395
AFG	2010	country	5_Emr	Afghanistan	55545	47502	103047	0.0472009	0.0403659	0.0875668	1176779	2229	1274	3503
AFG	2011	country	5_Emr	Afghanistan	53977	45860	99837	0.0457621	0.0388806	0.0846427	1179513	2264	1347	3612
AFG	2012	country	5_Emr	Afghanistan	52436	43521	95957	0.0443712	0.0368276	0.0811988	1181757	2267	1197	3464
AFG	2013	country	5_Emr	Afghanistan	50916	38054	88970	0.0429949	0.0321338	0.0751287	1184233	2312	1131	3442
AFG	2014	country	5_Emr	Afghanistan	49416	36495	85911	0.0416161	0.0307342	0.0723503	1187426	2331	1260	3591
AFG	2015	country	5_Emr	Afghanistan	47981	35288	83269	0.0402705	0.0296168	0.0698874	1191467	2365	1370	3735
AFG	2016	country	5_Emr	Afghanistan	46717	32912	79629	0.0390523	0.027512	0.0665643	1196268	2410	1375	3785
AFG	2017	country	5_Emr	Afghanistan	45625	32098	77723	0.0379776	0.0267178	0.0646954	1201366	2441	1347	3787
AFG	2018	country	5_Emr	Afghanistan	44503	31643	76146	0.0368932	0.0262323	0.0631254	1206267	2433	1343	3776
AFG	2019	country	5_Emr	Afghanistan	43424	28362	71786	0.0358629	0.0234238	0.0592866	1210834	2437	1314	3751
AGO	2000	country	1_Afr	Angola	39789	113195	152984	0.0502843	0.1430524	0.1933367	791281	1393	139	1531
AGO	2001	country	1_Afr	Angola	40193	115579	155772	0.0492404	0.1415952	0.1908356	816261	1435	314	1749
AGO	2002	country	1_Afr	Angola	40399	117618	158017	0.0479473	0.1395938	0.1875411	842572	1497	235	1732
AGO	2003	country	1_Afr	Angola	40568	112294	152862	0.046634	0.1290847	0.1757187	869924	1536	182	1718
AGO	2004	country	1_Afr	Angola	40565	105424	145989	0.0451695	0.1173909	0.1625604	898062	1603	206	1809
AGO	2005	country	1_Afr	Angola	40414	103204	143618	0.0436084	0.1113619	0.1549704	926747	1639	135	1774
AGO	2006	country	1_Afr	Angola	40110	100670	140780	0.0419708	0.1053401	0.1473109	955664	1696	162	1858
AGO	2007	country	1_Afr	Angola	39809	95599	135408	0.040435	0.0971019	0.1375368	984519	1723	517	2240
AGO	2008	country	1_Afr	Angola	39490	91766	131256	0.0389821	0.0905856	0.1295677	1013028	1803	502	2305
AGO	2009	country	1_Afr	Angola	39122	88699	127821	0.037586	0.0852161	0.1228021	1040866	1841	571	2412
AGO	2010	country	1_Afr	Angola	38656	82804	121460	0.0362039	0.0775517	0.1137556	1067730	1892	970	2862
AGO	2011	country	1_Afr	Angola	38148	79083	117231	0.034895	0.0723393	0.1072342	1093223	1902	942	2844
AGO	2012	country	1_Afr	Angola	37635	74301	111936	0.0336784	0.0664899	0.1001683	1117481	1952	1236	3187
AGO	2013	country	1_Afr	Angola	37255	72502	109757	0.032659	0.0635575	0.0962165	1140726	1976	1126	3102
AGO	2014	country	1_Afr	Angola	36745	73640	110385	0.0315876	0.0633038	0.0948915	1163272	2022	1144	3167
AGO	2015	country	1_Afr	Angola	36317	64216	100533	0.0306335	0.0541659	0.0847994	1185533	2029	1062	3091
AGO	2016	country	1_Afr	Angola	35974	62496	98470	0.0297773	0.0517304	0.0815077	1208103	2069	907	2977
AGO	2017	country	1_Afr	Angola	35717	60968	96685	0.0290027	0.0495069	0.0785096	1231507	2068	836	2904
AGO	2018	country	1_Afr	Angola	35489	59850	95339	0.0282521	0.0476456	0.0758977	1256154	2098	1068	3166
AGO	2019	country	1_Afr	Angola	35358	56999	92357	0.0275735	0.0444498	0.0720233	1282319	2133	1093	3226

iso3	year	level	whoreg6	whoname	nnd	pnd	u5d	nmr	pnmr	u5mr	Livebirths	Neonatal birth defects deaths	1-59 month birth defects deaths	Under five birth defects deaths
ALB	2000	country	4_Eur	Albania	625	823	1448	0.0121939	0.016057	0.0282509	51255	111	158	269
ALB	2001	country	4_Eur	Albania	574	726	1300	0.0118344	0.0149683	0.0268027	48503	104	145	249
ALB	2002	country	4_Eur	Albania	526	637	1163	0.0114477	0.0138634	0.0253111	45948	101	132	233
ALB	2003	country	4_Eur	Albania	485	552	1037	0.0111223	0.0126587	0.023781	43606	97	119	216
ALB	2004	country	4_Eur	Albania	451	468	919	0.0109011	0.011312	0.0222132	41372	92	104	197
ALB	2005	country	4_Eur	Albania	416	398	814	0.0105506	0.0100941	0.0206447	39429	92	92	185
ALB	2006	country	4_Eur	Albania	377	345	722	0.0099582	0.0091129	0.0190712	37858	85	83	168
ALB	2007	country	4_Eur	Albania	337	306	643	0.0091761	0.008332	0.0175081	36726	80	76	156
ALB	2008	country	4_Eur	Albania	298	278	576	0.0082645	0.0077098	0.0159743	36058	72	71	144
ALB	2009	country	4_Eur	Albania	263	257	520	0.0073888	0.0072202	0.014609	35594	66	69	136
ALB	2010	country	4_Eur	Albania	237	235	472	0.0066587	0.0066025	0.0132612	35593	61	66	126
ALB	2011	country	4_Eur	Albania	222	210	432	0.0062332	0.0058963	0.0121295	35616	57	61	118
ALB	2012	country	4_Eur	Albania	214	184	398	0.0060018	0.0051604	0.0111622	35656	56	55	111
ALB	2013	country	4_Eur	Albania	211	161	372	0.0058949	0.004498	0.0103929	35794	55	49	104
ALB	2014	country	4_Eur	Albania	210	142	352	0.0059063	0.0039938	0.0099	35556	55	44	99
ALB	2015	country	4_Eur	Albania	213	126	339	0.0060327	0.0035687	0.0096014	35307	57	40	97
ALB	2016	country	4_Eur	Albania	221	110	331	0.0063319	0.0031516	0.0094835	34903	59	35	94
ALB	2017	country	4_Eur	Albania	232	95	327	0.006746	0.0027624	0.0095084	34391	61	31	92
ALB	2018	country	4_Eur	Albania	243	83	326	0.0071481	0.0024415	0.0095896	33995	65	27	91
ALB	2019	country	4_Eur	Albania	251	76	327	0.007518	0.0022764	0.0097944	33386	67	25	92
AND	2000	country	4_Eur	Andorra	3	2	5	0.0039048	0.0026032	0.006508	768	0	2	2
AND	2001	country	4_Eur	Andorra	3	2	5	0.0036831	0.0024554	0.0061385	815	0	2	2
AND	2002	country	4_Eur	Andorra	3	2	5	0.0034492	0.0022995	0.0057487	870	0	2	2
AND	2003	country	4_Eur	Andorra	3	2	5	0.0032574	0.0021716	0.005429	921	0	2	2
AND	2004	country	4_Eur	Andorra	2	3	5	0.0030787	0.0046181	0.0076968	650	0	3	3
AND	2005	country	4_Eur	Andorra	2	3	5	0.0029133	0.00437	0.0072834	686	0	3	3
AND	2006	country	4_Eur	Andorra	2	3	5	0.0027405	0.0041107	0.0068512	730	0	3	3
AND	2007	country	4_Eur	Andorra	2	2	4	0.0025785	0.0025785	0.0051569	776	0	2	2
AND	2008	country	4_Eur	Andorra	2	2	4	0.0024253	0.0024253	0.0048506	825	0	2	2
AND	2009	country	4_Eur	Andorra	2	2	4	0.0022994	0.0022994	0.0045988	870	0	2	2
AND	2010	country	4_Eur	Andorra	2	2	4	0.0021873	0.0021873	0.0043746	914	0	2	2
AND	2011	country	4_Eur	Andorra	2	1	3	0.0020819	0.001041	0.0031229	961	0	1	1
AND	2012	country	4_Eur	Andorra	1	2	3	0.0019825	0.0039651	0.0059476	504	0	2	2
AND	2013	country	4_Eur	Andorra	1	2	3	0.0018928	0.0037857	0.0056785	528	0	0	0
AND	2014	country	4_Eur	Andorra	1	2	3	0.0018049	0.0036097	0.0054146	554	0	0	0
AND	2015	country	4_Eur	Andorra	1	1	2	0.0017104	0.0017104	0.0034208	585	0	0	0
AND	2016	country	4_Eur	Andorra	1	1	2	0.0016253	0.0016253	0.0032506	615	0	0	0
AND	2017	country	4_Eur	Andorra	1	1	2	0.0015555	0.0015555	0.0031109	643	0	0	0
AND	2018	country	4_Eur	Andorra	1	1	2	0.0014806	0.0014806	0.0029612	675	0	0	0
AND	2019	country	4_Eur	Andorra	1	1	2	0.0014254	0.0014254	0.0028507	702	0	0	0

iso3	year	level	whoreg6	whoname	nnd	pnd	u5d	nmr	pnmr	u5mr	Livebirths	Neonatal birth defects deaths	1-59 month birth defects deaths	Under five birth defects deaths
ARE	2000	country	5_Emr	United Arab Emirat	313	266	579	0.0059167	0.0050283	0.010945	52901	78	101	178
ARE	2001	country	5_Emr	United Arab Emirat	319	265	584	0.0057665	0.0047904	0.0105569	55320	81	101	182
ARE	2002	country	5_Emr	United Arab Emirat	328	267	595	0.0056202	0.004575	0.0101951	58361	83	102	185
ARE	2003	country	5_Emr	United Arab Emirat	340	272	612	0.0054736	0.0043789	0.0098525	62116	85	105	190
ARE	2004	country	5_Emr	United Arab Emirat	353	279	632	0.0053218	0.0042062	0.009528	66330	90	108	198
ARE	2005	country	5_Emr	United Arab Emirat	368	287	655	0.0051797	0.0040396	0.0092192	71047	96	112	208
ARE	2006	country	5_Emr	United Arab Emirat	382	297	679	0.0050335	0.0039135	0.0089469	75892	100	116	216
ARE	2007	country	5_Emr	United Arab Emirat	397	308	705	0.0049057	0.0038059	0.0087116	80927	107	122	228
ARE	2008	country	5_Emr	United Arab Emirat	410	319	729	0.0047787	0.003718	0.0084967	85798	112	127	240
ARE	2009	country	5_Emr	United Arab Emirat	420	330	750	0.004657	0.0036591	0.0083161	90186	117	134	250
ARE	2010	country	5_Emr	United Arab Emirat	427	340	767	0.0045387	0.0036139	0.0081526	94080	121	140	260
ARE	2011	country	5_Emr	United Arab Emirat	431	349	780	0.0044461	0.0036002	0.0080464	96938	121	144	266
ARE	2012	country	5_Emr	United Arab Emirat	433	355	788	0.0043689	0.0035819	0.0079508	99109	123	147	270
ARE	2013	country	5_Emr	United Arab Emirat	433	359	792	0.0043062	0.0035703	0.0078765	100552	124	149	272
ARE	2014	country	5_Emr	United Arab Emirat	431	362	793	0.004252	0.0035713	0.0078232	101365	124	149	273
ARE	2015	country	5_Emr	United Arab Emirat	426	364	790	0.0042033	0.0035915	0.0077948	101350	124	150	274
ARE	2016	country	5_Emr	United Arab Emirat	420	364	784	0.0041574	0.0036031	0.0077604	101026	124	150	274
ARE	2017	country	5_Emr	United Arab Emirat	414	362	776	0.0041099	0.0035937	0.0077036	100732	120	150	270
ARE	2018	country	5_Emr	United Arab Emirat	406	358	764	0.0040505	0.0035716	0.007622	100236	119	148	268
ARE	2019	country	5_Emr	United Arab Emirat	398	352	750	0.0039789	0.003519	0.0074979	100028	117	145	261
ARG	2000	country	2_Amr	Argentina	7865	6212	14077	0.0109346	0.0086365	0.0195711	719275	1783	1565	3348
ARG	2001	country	2_Amr	Argentina	7913	5705	13618	0.0109789	0.0079154	0.0188943	720746	1871	1522	3394
ARG	2002	country	2_Amr	Argentina	7799	5383	13182	0.0107922	0.0074489	0.0182411	722653	1807	1280	3087
ARG	2003	country	2_Amr	Argentina	7466	5285	12751	0.0103032	0.0072934	0.0175967	724626	1747	1234	2981
ARG	2004	country	2_Amr	Argentina	7050	5297	12347	0.0097049	0.0072917	0.0169966	726438	1744	1413	3157
ARG	2005	country	2_Amr	Argentina	6685	5312	11997	0.0091775	0.0072926	0.0164701	728409	1656	1479	3135
ARG	2006	country	2_Amr	Argentina	6392	5310	11702	0.0087491	0.0072681	0.0160172	730587	1728	1467	3195
ARG	2007	country	2_Amr	Argentina	6138	5315	11453	0.0083725	0.0072499	0.0156223	733117	1566	1333	2900
ARG	2008	country	2_Amr	Argentina	5915	5315	11230	0.0080357	0.0072206	0.0152563	736089	1552	1522	3074
ARG	2009	country	2_Amr	Argentina	5758	5226	10984	0.0077867	0.0070672	0.0148539	739468	1597	1440	3037
ARG	2010	country	2_Amr	Argentina	5689	4990	10679	0.0076556	0.006715	0.0143706	743114	1574	1414	2988
ARG	2011	country	2_Amr	Argentina	5658	4652	10310	0.0075772	0.00623	0.0138073	746709	1575	1256	2831
ARG	2012	country	2_Amr	Argentina	5588	4319	9907	0.0074517	0.0057595	0.0132112	749895	1605	1279	2884
ARG	2013	country	2_Amr	Argentina	5439	4051	9490	0.0072278	0.0053833	0.012611	752516	1457	1246	2703
ARG	2014	country	2_Amr	Argentina	5239	3825	9064	0.0069442	0.00507	0.0120141	754444	1514	1201	2715
ARG	2015	country	2_Amr	Argentina	5020	3620	8640	0.0066434	0.0047907	0.0114341	755635	1435	1149	2584
ARG	2016	country	2_Amr	Argentina	4826	3391	8217	0.0063839	0.0044857	0.0108695	755966	1377	1000	2377
ARG	2017	country	2_Amr	Argentina	4706	3086	7792	0.0062281	0.0040841	0.0103122	755609	1349	953	2302
ARG	2018	country	2_Amr	Argentina	4650	2723	7373	0.006161	0.0036078	0.0097688	754752	1330	841	2170
ARG	2019	country	2_Amr	Argentina	4583	2394	6977	0.0060816	0.0031768	0.0092584	753583	1311	739	2050

iso3	year	level	whoreg6	whoname	nnd	pnd	u5d	nmr	pnmr	u5mr	Livebirths	Neonatal birth defects deaths	1-59 month birth defects deaths	Under five birth defects deaths
ARM	2000	country	4_Eur	Armenia	651	590	1241	0.0164448	0.0149039	0.0313487	39587	90	107	196
ARM	2001	country	4_Eur	Armenia	626	535	1161	0.0158569	0.0135518	0.0294087	39478	90	99	189
ARM	2002	country	4_Eur	Armenia	602	501	1103	0.0151027	0.0125689	0.0276716	39860	91	93	184
ARM	2003	country	4_Eur	Armenia	582	478	1060	0.0143532	0.0117884	0.0261416	40548	94	92	186
ARM	2004	country	4_Eur	Armenia	565	459	1024	0.0136461	0.011086	0.0247321	41404	96	91	187
ARM	2005	country	4_Eur	Armenia	548	444	992	0.0129846	0.0105203	0.0235049	42204	99	91	190
ARM	2006	country	4_Eur	Armenia	530	430	960	0.0123288	0.0100026	0.0223314	42989	102	91	193
ARM	2007	country	4_Eur	Armenia	511	416	927	0.0117331	0.0095518	0.0212849	43552	104	91	195
ARM	2008	country	4_Eur	Armenia	492	401	893	0.0111742	0.0091075	0.0202817	44030	104	90	194
ARM	2009	country	4_Eur	Armenia	471	385	856	0.0106228	0.0086832	0.019306	44339	105	89	194
ARM	2010	country	4_Eur	Armenia	449	369	818	0.0100916	0.0082935	0.0183851	44493	106	88	194
ARM	2011	country	4_Eur	Armenia	426	353	779	0.0095984	0.0079536	0.0175519	44383	101	88	188
ARM	2012	country	4_Eur	Armenia	403	336	739	0.009107	0.0075929	0.0166999	44252	98	85	184
ARM	2013	country	4_Eur	Armenia	380	319	699	0.0086504	0.0072618	0.0159122	43929	94	83	177
ARM	2014	country	4_Eur	Armenia	358	303	661	0.0082131	0.0069513	0.0151644	43589	88	81	168
ARM	2015	country	4_Eur	Armenia	338	286	624	0.0078206	0.0066175	0.0144381	43219	85	78	163
ARM	2016	country	4_Eur	Armenia	317	270	587	0.007438	0.0063352	0.0137732	42619	79	75	154
ARM	2017	country	4_Eur	Armenia	297	254	551	0.0070622	0.0060397	0.0131019	42055	74	72	146
ARM	2018	country	4_Eur	Armenia	277	238	515	0.0067185	0.0057726	0.0124911	41229	71	69	140
ARM	2019	country	4_Eur	Armenia	258	224	482	0.0064044	0.0055604	0.0119648	40285	66	66	132
ATG	2000	country	2_Amr	Antigua and Barbud	14	9	23	0.0096292	0.0061902	0.0158194	1454	4	1	5
ATG	2001	country	2_Amr	Antigua and Barbud	14	8	22	0.0094385	0.0053935	0.014832	1483	2	3	4
ATG	2002	country	2_Amr	Antigua and Barbud	14	8	22	0.0091932	0.0052533	0.0144464	1523	1	0	1
ATG	2003	country	2_Amr	Antigua and Barbud	13	8	21	0.0088927	0.0054724	0.0143651	1462	13	6	19
ATG	2004	country	2_Amr	Antigua and Barbud	13	7	20	0.0085436	0.0046004	0.013144	1522	2	0	2
ATG	2005	country	2_Amr	Antigua and Barbud	12	7	19	0.0081375	0.0047469	0.0128844	1475	5	1	6
ATG	2006	country	2_Amr	Antigua and Barbud	11	7	18	0.007685	0.0048905	0.0125755	1431	0	1	1
ATG	2007	country	2_Amr	Antigua and Barbud	11	6	17	0.0072227	0.0039397	0.0111624	1523	3	1	4
ATG	2008	country	2_Amr	Antigua and Barbud	10	6	16	0.0067482	0.0040489	0.0107972	1482	2	0	2
ATG	2009	country	2_Amr	Antigua and Barbud	9	6	15	0.0062949	0.0041966	0.0104916	1430	0	1	1
ATG	2010	country	2_Amr	Antigua and Barbud	9	6	15	0.0058553	0.0039035	0.0097588	1537	2	1	3
ATG	2011	country	2_Amr	Antigua and Barbud	8	6	14	0.0054544	0.0040908	0.0095452	1467	2	1	3
ATG	2012	country	2_Amr	Antigua and Barbud	7	6	13	0.0050826	0.0043565	0.0094392	1377	2	1	3
ATG	2013	country	2_Amr	Antigua and Barbud	7	6	13	0.0047594	0.0040795	0.0088389	1471	2	1	3
ATG	2014	country	2_Amr	Antigua and Barbud	7	5	12	0.0044915	0.0032082	0.0076997	1559	4	1	4
ATG	2015	country	2_Amr	Antigua and Barbud	6	5	11	0.0042682	0.0035568	0.007825	1406	2	3	6
ATG	2016	country	2_Amr	Antigua and Barbud	6	5	11	0.0040861	0.0034051	0.0074912	1468	2	0	2
ATG	2017	country	2_Amr	Antigua and Barbud	6	5	11	0.0039186	0.0032655	0.0071841	1531	2	1	4
ATG	2018	country	2_Amr	Antigua and Barbud	6	4	10	0.0037643	0.0025095	0.0062738	1594	2	1	3
ATG	2019	country	2_Amr	Antigua and Barbud	5	5	10	0.0036323	0.0036323	0.0072646	1377	2	1	3

iso3	year	level	whoreg6	whoname	nnd	pnd	u5d	nmr	pnmr	u5mr	Livebirths	Neonatal birth defects deaths	1-59 month birth defects deaths	Under five birth defects deaths
AUS	2000	country	6_Wpr	Australia	870	662	1532	0.0035252	0.0026824	0.0062075	246797	268	163	432
AUS	2001	country	6_Wpr	Australia	865	639	1504	0.0034875	0.0025763	0.0060638	248028	269	135	404
AUS	2002	country	6_Wpr	Australia	863	628	1491	0.0034379	0.0025018	0.0059397	251022	230	151	381
AUS	2003	country	6_Wpr	Australia	864	628	1492	0.0033752	0.0024533	0.0058286	255981	253	134	387
AUS	2004	country	6_Wpr	Australia	867	635	1502	0.0033021	0.0024185	0.0057206	262558	275	119	394
AUS	2005	country	6_Wpr	Australia	871	640	1511	0.0032258	0.0023703	0.0055961	270012	262	147	409
AUS	2006	country	6_Wpr	Australia	872	642	1514	0.0031359	0.0023087	0.0054446	278073	241	114	354
AUS	2007	country	6_Wpr	Australia	870	638	1508	0.003047	0.0022344	0.0052814	285530	283	144	427
AUS	2008	country	6_Wpr	Australia	861	631	1492	0.0029443	0.0021578	0.0051021	292428	282	143	425
AUS	2009	country	6_Wpr	Australia	846	617	1463	0.0028373	0.0020693	0.0049065	298176	254	158	412
AUS	2010	country	6_Wpr	Australia	825	596	1421	0.0027296	0.0019719	0.0047016	302241	261	148	409
AUS	2011	country	6_Wpr	Australia	800	571	1371	0.0026177	0.0018684	0.0044861	305607	226	140	366
AUS	2012	country	6_Wpr	Australia	777	543	1320	0.0025191	0.0017604	0.0042795	308449	249	102	351
AUS	2013	country	6_Wpr	Australia	759	515	1274	0.0024448	0.0016588	0.0041036	310461	234	121	354
AUS	2014	country	6_Wpr	Australia	746	490	1236	0.0023852	0.0015667	0.0039518	312767	208	124	332
AUS	2015	country	6_Wpr	Australia	739	469	1208	0.0023484	0.0014904	0.0038389	314676	225	90	314
AUS	2016	country	6_Wpr	Australia	735	454	1189	0.0023239	0.0014354	0.0037593	316283	221	114	336
AUS	2017	country	6_Wpr	Australia	734	441	1175	0.0023117	0.0013889	0.0037007	317509	216	102	319
AUS	2018	country	6_Wpr	Australia	732	430	1162	0.0022984	0.0013502	0.0036486	318480	219	100	319
AUS	2019	country	6_Wpr	Australia	726	420	1146	0.0022773	0.0013175	0.0035948	318792	217	98	314
AUT	2000	country	4_Eur	Austria	248	201	449	0.0030867	0.0025017	0.0055883	80346	92	78	171
AUT	2001	country	4_Eur	Austria	244	192	436	0.003074	0.0024189	0.0054929	79375	81	69	149
AUT	2002	country	4_Eur	Austria	239	184	423	0.0030472	0.002346	0.0053931	78433	63	59	122
AUT	2003	country	4_Eur	Austria	234	175	409	0.0029991	0.0022429	0.005242	78023	53	63	116
AUT	2004	country	4_Eur	Austria	227	167	394	0.0029269	0.0021533	0.0050802	77556	59	54	114
AUT	2005	country	4_Eur	Austria	219	160	379	0.0028387	0.0020739	0.0049126	77148	63	52	115
AUT	2006	country	4_Eur	Austria	212	155	367	0.0027529	0.0020127	0.0047656	77010	47	43	90
AUT	2007	country	4_Eur	Austria	207	151	358	0.0026781	0.0019536	0.0046318	77293	51	48	98
AUT	2008	country	4_Eur	Austria	202	149	351	0.0026148	0.0019287	0.0045435	77254	66	48	114
AUT	2009	country	4_Eur	Austria	199	146	345	0.0025594	0.0018777	0.0044371	77753	64	50	113
AUT	2010	country	4_Eur	Austria	197	141	338	0.0025066	0.0017941	0.0043007	78592	55	54	108
AUT	2011	country	4_Eur	Austria	194	137	331	0.0024541	0.0017331	0.0041872	79051	73	47	120
AUT	2012	country	4_Eur	Austria	192	130	322	0.0023966	0.0016227	0.0040193	80114	67	40	107
AUT	2013	country	4_Eur	Austria	191	123	314	0.0023405	0.0015073	0.0038478	81605	73	42	115
AUT	2014	country	4_Eur	Austria	190	120	310	0.00229	0.0014463	0.0037363	82970	64	41	105
AUT	2015	country	4_Eur	Austria	189	119	308	0.0022454	0.0014138	0.0036592	84172	62	44	106
AUT	2016	country	4_Eur	Austria	189	119	308	0.0022014	0.0013861	0.0035875	85854	74	46	120
AUT	2017	country	4_Eur	Austria	188	120	308	0.0021579	0.0013774	0.0035353	87122	51	38	89
AUT	2018	country	4_Eur	Austria	186	122	308	0.0021086	0.0013831	0.0034917	88210	61	43	105
AUT	2019	country	4_Eur	Austria	184	122	306	0.0020628	0.0013677	0.0034305	89201	61	43	104

iso3	year	level	whoreg6	whoname	nnd	pnd	u5d	nmr	pnmr	u5mr	Livebirths	Neonatal birth defects deaths	1-59 month birth defects deaths	Under five birth defects deaths
AZE	2000	country	4_Eur	Azerbaijan	4716	6051	10767	0.0334714	0.0429465	0.0764179	140896	455	274	728
AZE	2001	country	4_Eur	Azerbaijan	4424	5316	9740	0.0320729	0.0385397	0.0706127	137936	442	267	710
AZE	2002	country	4_Eur	Azerbaijan	4178	4693	8871	0.0306607	0.0344401	0.0651008	136266	431	243	673
AZE	2003	country	4_Eur	Azerbaijan	3963	4181	8144	0.0292131	0.03082	0.0600331	135659	406	237	644
AZE	2004	country	4_Eur	Azerbaijan	3781	3785	7566	0.0277643	0.0277937	0.055558	136182	393	229	622
AZE	2005	country	4_Eur	Azerbaijan	3633	3468	7101	0.0263492	0.0251525	0.0515017	137879	391	222	613
AZE	2006	country	4_Eur	Azerbaijan	3524	3229	6753	0.02499	0.0228981	0.0478881	141016	387	229	615
AZE	2007	country	4_Eur	Azerbaijan	3445	3038	6483	0.0236666	0.0208706	0.0445371	145564	378	260	639
AZE	2008	country	4_Eur	Azerbaijan	3388	2886	6274	0.0224047	0.019085	0.0414897	151218	376	282	658
AZE	2009	country	4_Eur	Azerbaijan	3335	2759	6094	0.0211754	0.0175181	0.0386935	157494	379	321	700
AZE	2010	country	4_Eur	Azerbaijan	3268	2651	5919	0.0199404	0.0161757	0.0361161	163888	375	351	727
AZE	2011	country	4_Eur	Azerbaijan	3179	2564	5743	0.0187384	0.0151134	0.0338518	169651	390	380	770
AZE	2012	country	4_Eur	Azerbaijan	3070	2458	5528	0.0176166	0.0141048	0.0317214	174267	409	402	811
AZE	2013	country	4_Eur	Azerbaijan	2937	2338	5275	0.0165643	0.013186	0.0297503	177309	425	420	844
AZE	2014	country	4_Eur	Azerbaijan	2755	2232	4987	0.0154297	0.0125006	0.0279303	178551	425	421	846
AZE	2015	country	4_Eur	Azerbaijan	2533	2126	4659	0.0142428	0.0119543	0.0261971	177844	427	413	839
AZE	2016	country	4_Eur	Azerbaijan	2316	2002	4318	0.0132021	0.0114121	0.0246142	175427	412	402	814
AZE	2017	country	4_Eur	Azerbaijan	2102	1877	3979	0.0122488	0.0109377	0.0231865	171608	409	391	800
AZE	2018	country	4_Eur	Azerbaijan	1925	1735	3660	0.0115197	0.0103827	0.0219025	167105	384	374	758
AZE	2019	country	4_Eur	Azerbaijan	1765	1605	3370	0.0108918	0.0099045	0.0207963	162048	366	357	723
BDI	2000	country	1_Afr	Burundi	10270	33362	43632	0.0369089	0.1198973	0.1568062	278253	392	87	479
BDI	2001	country	1_Afr	Burundi	10279	32750	43029	0.0362416	0.1154686	0.1517102	283624	402	101	504
BDI	2002	country	1_Afr	Burundi	10317	29708	40025	0.0355243	0.1022922	0.1378165	290421	427	206	632
BDI	2003	country	1_Afr	Burundi	10379	28709	39088	0.0347754	0.096192	0.1309674	298458	456	173	629
BDI	2004	country	1_Afr	Burundi	10454	27889	38343	0.0339891	0.0906769	0.124666	307569	492	234	725
BDI	2005	country	1_Afr	Burundi	10490	26946	37436	0.0330302	0.084846	0.1178762	317588	520	554	1074
BDI	2006	country	1_Afr	Burundi	10527	25923	36450	0.0320653	0.0789629	0.1110282	328299	554	479	1032
BDI	2007	country	1_Afr	Burundi	10532	24662	35194	0.0310301	0.0726619	0.103692	339413	581	484	1064
BDI	2008	country	1_Afr	Burundi	10510	23689	34199	0.0299696	0.0675488	0.0975184	350689	612	471	1083
BDI	2009	country	1_Afr	Burundi	10449	22744	33193	0.0288822	0.0628664	0.0917485	361781	650	594	1245
BDI	2010	country	1_Afr	Burundi	10354	21745	32099	0.0277991	0.0583834	0.0861825	372458	697	655	1352
BDI	2011	country	1_Afr	Burundi	10249	20804	31053	0.0267932	0.0543874	0.0811806	382523	693	744	1437
BDI	2012	country	1_Afr	Burundi	10126	19910	30036	0.0258384	0.0508051	0.0766435	391897	711	876	1587
BDI	2013	country	1_Afr	Burundi	9975	18972	28947	0.0248986	0.0473572	0.0722559	400624	736	1025	1761
BDI	2014	country	1_Afr	Burundi	9822	18164	27986	0.0240321	0.0444435	0.0684756	408704	757	971	1728
BDI	2015	country	1_Afr	Burundi	9697	17412	27109	0.0232989	0.0418356	0.0651345	416200	760	926	1687
BDI	2016	country	1_Afr	Burundi	9570	16786	26356	0.0226144	0.0396668	0.0622812	423182	764	832	1596
BDI	2017	country	1_Afr	Burundi	9464	16232	25696	0.0220167	0.0377617	0.0597784	429855	774	731	1505
BDI	2018	country	1_Afr	Burundi	9361	15630	24991	0.0214528	0.0358199	0.0572727	436353	782	795	1577
BDI	2019	country	1_Afr	Burundi	9288	15126	24414	0.0209744	0.0341588	0.0551331	442826	790	789	1579

iso3	year	level	whoreg6	whoname	nnd	pnd	u5d	nmr	pnmr	u5mr	Livebirths	Neonatal birth defects deaths	1-59 month birth defects deaths	Under five birth defects deaths
BEL	2000	country	4_Eur	Belgium	336	327	663	0.0029463	0.0028674	0.0058137	114041	122	82	204
BEL	2001	country	4_Eur	Belgium	324	315	639	0.0028375	0.0027587	0.0055962	114185	108	74	181
BEL	2002	country	4_Eur	Belgium	318	301	619	0.002761	0.0026134	0.0053745	115174	114	67	181
BEL	2003	country	4_Eur	Belgium	315	289	604	0.0027027	0.0024797	0.0051824	116548	112	63	174
BEL	2004	country	4_Eur	Belgium	314	278	592	0.0026554	0.002351	0.0050064	118248	115	76	191
BEL	2005	country	4_Eur	Belgium	314	270	584	0.0026145	0.0022481	0.0048626	120100	92	54	146
BEL	2006	country	4_Eur	Belgium	314	266	580	0.0025738	0.0021804	0.0047542	121996	92	68	160
BEL	2007	country	4_Eur	Belgium	312	265	577	0.0025237	0.0021435	0.0046673	123627	98	65	163
BEL	2008	country	4_Eur	Belgium	308	266	574	0.0024615	0.0021258	0.0045873	125128	94	71	166
BEL	2009	country	4_Eur	Belgium	302	267	569	0.0023959	0.0021182	0.0045141	126051	91	65	157
BEL	2010	country	4_Eur	Belgium	297	266	563	0.0023398	0.0020956	0.0044354	126932	91	70	162
BEL	2011	country	4_Eur	Belgium	292	264	556	0.0022995	0.002079	0.0043785	126985	87	62	149
BEL	2012	country	4_Eur	Belgium	289	257	546	0.0022749	0.002023	0.0042979	127037	112	66	178
BEL	2013	country	4_Eur	Belgium	287	245	532	0.0022603	0.0019295	0.0041898	126974	95	60	155
BEL	2014	country	4_Eur	Belgium	284	231	515	0.0022462	0.001827	0.0040732	126437	81	68	149
BEL	2015	country	4_Eur	Belgium	280	217	497	0.0022224	0.0017224	0.0039448	125988	69	39	108
BEL	2016	country	4_Eur	Belgium	274	203	477	0.0021848	0.0016186	0.0038034	125414	76	56	132
BEL	2017	country	4_Eur	Belgium	267	191	458	0.0021345	0.0015269	0.0036614	125089	72	48	120
BEL	2018	country	4_Eur	Belgium	258	183	441	0.0020704	0.0014686	0.003539	124613	68	46	114
BEL	2019	country	4_Eur	Belgium	249	175	424	0.0020013	0.0014065	0.0034078	124422	67	44	111
BEN	2000	country	1_Afr	Benin	11533	29522	41055	0.0395537	0.1012477	0.1408013	291579	613	199	812
BEN	2001	country	1_Afr	Benin	11554	30923	42477	0.0387903	0.103819	0.1426094	297858	615	189	804
BEN	2002	country	1_Afr	Benin	11577	27331	38908	0.0380352	0.0897929	0.1278281	304376	637	182	818
BEN	2003	country	1_Afr	Benin	11612	26507	38119	0.0373285	0.0852116	0.1225401	311076	650	171	821
BEN	2004	country	1_Afr	Benin	11674	25873	37547	0.0367097	0.0813592	0.1180688	318009	670	153	823
BEN	2005	country	1_Afr	Benin	11771	25997	37768	0.0362029	0.0799552	0.1161582	325140	678	140	819
BEN	2006	country	1_Afr	Benin	11895	25797	37692	0.0357823	0.0776012	0.1133835	332427	711	108	819
BEN	2007	country	1_Afr	Benin	12050	25999	38049	0.035462	0.0765117	0.1119737	339800	722	139	861
BEN	2008	country	1_Afr	Benin	12206	26450	38656	0.0351552	0.0761796	0.1113348	347203	742	154	896
BEN	2009	country	1_Afr	Benin	12346	26763	39109	0.0348212	0.0754842	0.1103054	354554	756	208	964
BEN	2010	country	1_Afr	Benin	12477	26329	38806	0.034484	0.0727694	0.1072535	361820	787	221	1008
BEN	2011	country	1_Afr	Benin	12586	26195	38781	0.0341128	0.0709975	0.1051103	368952	784	264	1048
BEN	2012	country	1_Afr	Benin	12669	25699	38368	0.0336995	0.0683598	0.1020593	375940	805	456	1261
BEN	2013	country	1_Afr	Benin	12738	25945	38683	0.0332752	0.0677744	0.1010496	382808	832	246	1078
BEN	2014	country	1_Afr	Benin	12783	26056	38839	0.0328129	0.0668834	0.0996962	389573	830	223	1053
BEN	2015	country	1_Afr	Benin	12818	25152	37970	0.0323486	0.0634766	0.0958252	396246	842	224	1066
BEN	2016	country	1_Afr	Benin	12853	25094	37947	0.0319012	0.0622822	0.0941834	402901	836	338	1174
BEN	2017	country	1_Afr	Benin	12886	24935	37821	0.0314621	0.0608797	0.0923418	409572	859	406	1265
BEN	2018	country	1_Afr	Benin	12921	25096	38017	0.0310357	0.0602785	0.0913143	416327	877	299	1176
BEN	2019	country	1_Afr	Benin	12931	24617	37548	0.0305589	0.058176	0.0887349	423150	881	302	1183

iso3	year	level	whoreg6	whoname	nnd	pnd	u5d	nmr	pnmr	u5mr	Livebirths	Neonatal birth defects deaths	1-59 month birth defects deaths	Under five birth defects deaths
BFA	2000	country	1_Afr	Burkina Faso	21982	77559	99541	0.0408794	0.1442353	0.1851147	537728	925	39	964
BFA	2001	country	1_Afr	Burkina Faso	22099	71738	93837	0.040124	0.1302502	0.1703741	550768	977	62	1039
BFA	2002	country	1_Afr	Burkina Faso	22147	72987	95134	0.0392751	0.1294344	0.1687094	563895	1001	75	1077
BFA	2003	country	1_Afr	Burkina Faso	22122	70052	92174	0.0383366	0.1213968	0.1597335	577046	1045	129	1173
BFA	2004	country	1_Afr	Burkina Faso	21965	65853	87818	0.0372217	0.1115942	0.1488159	590113	1075	184	1259
BFA	2005	country	1_Afr	Burkina Faso	21709	64020	85729	0.0360017	0.1061696	0.1421713	603000	1092	279	1371
BFA	2006	country	1_Afr	Burkina Faso	21367	63031	84398	0.0347155	0.1024087	0.1371241	615489	1128	367	1495
BFA	2007	country	1_Afr	Burkina Faso	21044	61253	82297	0.0335339	0.0976078	0.1311418	627544	1144	452	1596
BFA	2008	country	1_Afr	Burkina Faso	20789	59205	79994	0.032529	0.0926399	0.1251689	639091	1185	413	1598
BFA	2009	country	1_Afr	Burkina Faso	20562	74987	95549	0.0316223	0.1153228	0.1469451	650238	1205	249	1453
BFA	2010	country	1_Afr	Burkina Faso	20438	58240	78678	0.0309188	0.0881057	0.1190245	661021	1249	370	1619
BFA	2011	country	1_Afr	Burkina Faso	20301	55610	75911	0.0302276	0.082801	0.1130286	671606	1243	270	1513
BFA	2012	country	1_Afr	Burkina Faso	20229	63982	84211	0.0296526	0.0937873	0.12344	682199	1271	449	1721
BFA	2013	country	1_Afr	Burkina Faso	20123	52761	72884	0.0290421	0.0761463	0.1051884	692891	1291	373	1664
BFA	2014	country	1_Afr	Burkina Faso	20074	51104	71178	0.0285214	0.0726091	0.1011305	703823	1327	648	1975
BFA	2015	country	1_Afr	Burkina Faso	20016	48803	68819	0.0279927	0.0682512	0.096244	715043	1354	730	2083
BFA	2016	country	1_Afr	Burkina Faso	19956	47857	67813	0.0274678	0.0658709	0.0933387	726524	1383	782	2165
BFA	2017	country	1_Afr	Burkina Faso	19905	46880	66785	0.0269643	0.0635057	0.09047	738198	1397	801	2198
BFA	2018	country	1_Afr	Burkina Faso	19866	50869	70735	0.0264884	0.0678267	0.094315	749990	1418	818	2236
BFA	2019	country	1_Afr	Burkina Faso	19767	45361	65128	0.0259442	0.0595369	0.085481	761905	1403	829	2231
BGD	2000	country	3_Sear	Bangladesh	150029	152332	302361	0.0427631	0.0434195	0.0861826	3508375	6803	3789	10592
BGD	2001	country	3_Sear	Bangladesh	143195	141061	284256	0.0410848	0.0404724	0.0815572	3485349	6769	4135	10904
BGD	2002	country	3_Sear	Bangladesh	136430	129986	266416	0.0394778	0.0376131	0.0770909	3455862	6607	3990	10596
BGD	2003	country	3_Sear	Bangladesh	129918	121282	251200	0.037985	0.0354601	0.0734451	3420241	6565	4083	10648
BGD	2004	country	3_Sear	Bangladesh	123436	122679	246115	0.0365274	0.0363035	0.0728308	3379274	6548	4532	11081
BGD	2005	country	3_Sear	Bangladesh	117138	112703	229841	0.0351298	0.0337998	0.0689296	3334434	6446	5221	11668
BGD	2006	country	3_Sear	Bangladesh	110896	101009	211905	0.0337312	0.0307239	0.0644551	3287637	6328	4561	10890
BGD	2007	country	3_Sear	Bangladesh	104858	80640	185498	0.0323523	0.0248801	0.0572324	3241133	6217	3623	9840
BGD	2008	country	3_Sear	Bangladesh	99033	76469	175502	0.0309771	0.0239192	0.0548963	3196973	6122	3871	9993
BGD	2009	country	3_Sear	Bangladesh	93498	69986	163484	0.0296218	0.0221728	0.0517947	3156390	6111	4005	10115
BGD	2010	country	3_Sear	Bangladesh	88248	64942	153190	0.0282813	0.0208123	0.0490936	3120367	5917	3580	9497
BGD	2011	country	3_Sear	Bangladesh	83370	62039	145409	0.0269874	0.0200825	0.0470699	3089219	5905	3919	9824
BGD	2012	country	3_Sear	Bangladesh	78881	55758	134639	0.0257616	0.01821	0.0439717	3061959	5771	3426	9198
BGD	2013	country	3_Sear	Bangladesh	74762	51880	126642	0.0246135	0.0170801	0.0416937	3037438	5731	4919	10650
BGD	2014	country	3_Sear	Bangladesh	70921	48413	119334	0.0235217	0.0160569	0.0395786	3015125	5701	5036	10737
BGD	2015	country	3_Sear	Bangladesh	67411	44038	111449	0.0225127	0.014707	0.0372198	2994348	5599	5363	10962
BGD	2016	country	3_Sear	Bangladesh	64134	41226	105360	0.0215614	0.0138599	0.0354213	2974485	5597	3738	9335
BGD	2017	country	3_Sear	Bangladesh	61061	41258	102319	0.0206644	0.0139628	0.0346272	2954887	5481	3107	8587
BGD	2018	country	3_Sear	Bangladesh	58203	39429	97632	0.0198326	0.0134352	0.0332679	2934708	5451	2652	8103
BGD	2019	country	3_Sear	Bangladesh	55542	42217	97759	0.0190645	0.0144907	0.0335552	2913368	5436	2633	8069

iso3	year	level	whoreg6	whoname	nnd	pnd	u5d	nmr	pnmr	u5mr	Livebirths	Neonatal birth defects deaths	1-59 month birth defects deaths	Under five birth defects deaths
BGR	2000	country	4_Eur	Bulgaria	511	644	1155	0.0078271	0.0098642	0.0176913	65286	155	164	319
BGR	2001	country	4_Eur	Bulgaria	495	602	1097	0.0075234	0.0091497	0.0166731	65794	152	164	316
BGR	2002	country	4_Eur	Bulgaria	485	574	1059	0.0072432	0.0085724	0.0158156	66959	154	132	286
BGR	2003	country	4_Eur	Bulgaria	472	551	1023	0.00688	0.0080316	0.0149116	68604	147	119	265
BGR	2004	country	4_Eur	Bulgaria	453	527	980	0.0064233	0.0074726	0.0138958	70525	138	122	260
BGR	2005	country	4_Eur	Bulgaria	433	503	936	0.0059863	0.0069541	0.0129403	72332	147	109	257
BGR	2006	country	4_Eur	Bulgaria	413	485	898	0.0055984	0.0065744	0.0121728	73771	131	73	204
BGR	2007	country	4_Eur	Bulgaria	398	469	867	0.0053204	0.0062696	0.01159	74806	98	94	192
BGR	2008	country	4_Eur	Bulgaria	389	456	845	0.0051813	0.0060737	0.0112549	75078	109	107	216
BGR	2009	country	4_Eur	Bulgaria	385	443	828	0.0051605	0.005938	0.0110985	74605	94	84	178
BGR	2010	country	4_Eur	Bulgaria	377	427	804	0.0051234	0.0058028	0.0109262	73585	94	101	196
BGR	2011	country	4_Eur	Bulgaria	359	403	762	0.0049755	0.0055853	0.0105607	72154	95	110	205
BGR	2012	country	4_Eur	Bulgaria	335	371	706	0.0047421	0.0052517	0.0099939	70643	79	88	167
BGR	2013	country	4_Eur	Bulgaria	309	339	648	0.0044876	0.0049233	0.0094108	68857	79	94	174
BGR	2014	country	4_Eur	Bulgaria	286	307	593	0.0042501	0.0045622	0.0088122	67293	66	66	132
BGR	2015	country	4_Eur	Bulgaria	266	280	546	0.0040365	0.0042489	0.0082854	65899	89	82	171
BGR	2016	country	4_Eur	Bulgaria	250	258	508	0.0038549	0.0039782	0.0078331	64853	68	68	136
BGR	2017	country	4_Eur	Bulgaria	235	241	476	0.0036783	0.0037723	0.0074506	63887	66	63	129
BGR	2018	country	4_Eur	Bulgaria	220	228	448	0.0034935	0.0036206	0.0071141	62973	65	60	125
BGR	2019	country	4_Eur	Bulgaria	206	216	422	0.0033313	0.003493	0.0068243	61837	58	57	115
BHR	2000	country	5_Emr	Bahrain	70	112	182	0.0047514	0.0076023	0.0123537	14732	30	38	67
BHR	2001	country	5_Emr	Bahrain	67	112	179	0.0043838	0.0073281	0.0117119	15284	30	52	83
BHR	2002	country	5_Emr	Bahrain	64	113	177	0.0041238	0.0072811	0.0114049	15520	31	43	74
BHR	2003	country	5_Emr	Bahrain	63	114	177	0.0039199	0.0070932	0.0110132	16072	26	52	78
BHR	2004	country	5_Emr	Bahrain	62	115	177	0.0037437	0.006944	0.0106878	16561	19	34	52
BHR	2005	country	5_Emr	Bahrain	61	116	177	0.0035866	0.0068205	0.0104071	17008	20	34	54
BHR	2006	country	5_Emr	Bahrain	61	115	176	0.0034432	0.0064913	0.0099345	17716	14	39	54
BHR	2007	country	5_Emr	Bahrain	60	113	173	0.0033198	0.0062523	0.009572	18073	24	49	73
BHR	2008	country	5_Emr	Bahrain	60	110	170	0.0032296	0.005921	0.0091507	18578	22	33	55
BHR	2009	country	5_Emr	Bahrain	61	106	167	0.003174	0.0055155	0.0086896	19218	26	30	55
BHR	2010	country	5_Emr	Bahrain	62	102	164	0.0031516	0.0051849	0.0083365	19672	32	31	63
BHR	2011	country	5_Emr	Bahrain	64	99	163	0.0031628	0.0048925	0.0080553	20235	40	35	75
BHR	2012	country	5_Emr	Bahrain	66	96	162	0.0031908	0.0046411	0.0078319	20685	27	30	57
BHR	2013	country	5_Emr	Bahrain	67	94	161	0.0032134	0.0045084	0.0077218	20850	22	34	56
BHR	2014	country	5_Emr	Bahrain	68	93	161	0.0032189	0.0044023	0.0076211	21126	24	31	55
BHR	2015	country	5_Emr	Bahrain	69	92	161	0.0032065	0.0042753	0.0074817	21519	25	31	56
BHR	2016	country	5_Emr	Bahrain	69	90	159	0.0031658	0.0041293	0.0072951	21796	24	30	54
BHR	2017	country	5_Emr	Bahrain	68	89	157	0.0031058	0.0040649	0.0071707	21895	24	30	54
BHR	2018	country	5_Emr	Bahrain	66	88	154	0.003025	0.0040334	0.0070584	21818	24	30	53
BHR	2019	country	5_Emr	Bahrain	65	86	151	0.0029391	0.0038887	0.0068278	22115	23	29	52

iso3	year	level	whoreg6	whoname	nnd	pnd	u5d	nmr	pnmr	u5mr	Livebirths	Neonatal birth defects deaths	1-59 month birth defects deaths	Under five birth defects deaths
BHS	2000	country	2_Amr	Bahamas	43	45	88	0.0080729	0.0084484	0.0165213	5326	7	10	18
BHS	2001	country	2_Amr	Bahamas	42	44	86	0.0082246	0.0086162	0.0168408	5107	5	7	13
BHS	2002	country	2_Amr	Bahamas	42	42	84	0.0083999	0.0083999	0.0167999	5000	6	2	8
BHS	2003	country	2_Amr	Bahamas	42	42	84	0.0085318	0.0085318	0.0170636	4923	6	15	21
BHS	2004	country	2_Amr	Bahamas	43	41	84	0.008598	0.0081981	0.0167962	5001	9	12	21
BHS	2005	country	2_Amr	Bahamas	43	41	84	0.008547	0.0081494	0.0166964	5031	4	6	10
BHS	2006	country	2_Amr	Bahamas	44	40	84	0.0085916	0.0078106	0.0164022	5121	3	21	23
BHS	2007	country	2_Amr	Bahamas	45	39	84	0.0087339	0.0075694	0.0163032	5152	5	13	17
BHS	2008	country	2_Amr	Bahamas	45	39	84	0.0085986	0.0074521	0.0160507	5233	5	6	10
BHS	2009	country	2_Amr	Bahamas	44	39	83	0.008417	0.0074605	0.0158775	5228	4	2	6
BHS	2010	country	2_Amr	Bahamas	43	39	82	0.0080109	0.0072657	0.0152766	5368	10	2	12
BHS	2011	country	2_Amr	Bahamas	42	38	80	0.0078148	0.0070706	0.0148854	5374	4	0	4
BHS	2012	country	2_Amr	Bahamas	41	38	79	0.0076721	0.0071107	0.0147827	5344	8	2	10
BHS	2013	country	2_Amr	Bahamas	41	36	77	0.007791	0.0068409	0.014632	5262	7	9	17
BHS	2014	country	2_Amr	Bahamas	40	36	76	0.0075647	0.0068082	0.0143729	5288	6	11	17
BHS	2015	country	2_Amr	Bahamas	40	34	74	0.007559	0.0064251	0.0139841	5292	7	7	14
BHS	2016	country	2_Amr	Bahamas	39	34	73	0.0073866	0.0064396	0.0138262	5280	7	7	14
BHS	2017	country	2_Amr	Bahamas	38	33	71	0.0069756	0.0060578	0.0130334	5448	7	7	13
BHS	2018	country	2_Amr	Bahamas	37	32	69	0.0068	0.0058811	0.0126811	5441	6	7	13
BHS	2019	country	2_Amr	Bahamas	36	32	68	0.0066485	0.0059098	0.0125583	5415	6	7	13
BIH	2000	country	4_Eur	Bosnia and Herzego	283	142	425	0.0068743	0.0034413	0.0103156	41168	77	43	120
BIH	2001	country	4_Eur	Bosnia and Herzego	255	134	389	0.0065192	0.003414	0.0099332	39115	70	41	111
BIH	2002	country	4_Eur	Bosnia and Herzego	231	130	361	0.0061961	0.0034991	0.0096952	37281	62	41	102
BIH	2003	country	4_Eur	Bosnia and Herzego	213	127	340	0.0059183	0.0035394	0.0094577	35990	57	40	97
BIH	2004	country	4_Eur	Bosnia and Herzego	200	125	325	0.0056913	0.0035662	0.0092575	35141	54	40	94
BIH	2005	country	4_Eur	Bosnia and Herzego	191	121	312	0.0055098	0.0034981	0.0090079	34665	52	39	91
BIH	2006	country	4_Eur	Bosnia and Herzego	186	113	299	0.0053749	0.0032715	0.0086464	34605	51	37	87
BIH	2007	country	4_Eur	Bosnia and Herzego	183	103	286	0.0053031	0.0029923	0.0082954	34508	49	34	83
BIH	2008	country	4_Eur	Bosnia and Herzego	181	91	272	0.0052581	0.0026499	0.007908	34423	50	30	80
BIH	2009	country	4_Eur	Bosnia and Herzego	178	80	258	0.0051949	0.0023406	0.0075354	34264	49	27	76
BIH	2010	country	4_Eur	Bosnia and Herzego	173	72	245	0.0051025	0.0021295	0.0072319	33905	48	24	72
BIH	2011	country	4_Eur	Bosnia and Herzego	167	65	232	0.0049964	0.0019505	0.0069469	33424	46	22	69
BIH	2012	country	4_Eur	Bosnia and Herzego	159	61	220	0.0048766	0.0018768	0.0067534	32605	44	21	65
BIH	2013	country	4_Eur	Bosnia and Herzego	151	58	209	0.0047596	0.0018341	0.0065937	31726	43	20	63
BIH	2014	country	4_Eur	Bosnia and Herzego	144	59	203	0.0046521	0.001891	0.0065431	30954	40	20	60
BIH	2015	country	4_Eur	Bosnia and Herzego	137	55	192	0.0045567	0.0018312	0.0063879	30065	38	19	57
BIH	2016	country	4_Eur	Bosnia and Herzego	130	54	184	0.0044678	0.0018622	0.00633	29097	37	19	56
BIH	2017	country	4_Eur	Bosnia and Herzego	123	53	176	0.0043896	0.0018905	0.0062801	28021	34	18	52
BIH	2018	country	4_Eur	Bosnia and Herzego	117	51	168	0.0043068	0.0018768	0.0061836	27166	33	17	50
BIH	2019	country	4_Eur	Bosnia and Herzego	111	49	160	0.0042179	0.0018538	0.0060716	26317	31	16	48

iso3	year	level	whoreg6	whoname	nnd	pnd	u5d	nmr	pnmr	u5mr	Livebirths	Neonatal birth defects deaths	1-59 month birth defects deaths	Under five birth defects deaths
BLR	2000	country	4_Eur	Belarus	533	618	1151	0.0060414	0.0070048	0.0130462	88225	145	178	322
BLR	2001	country	4_Eur	Belarus	480	566	1046	0.0054618	0.0064404	0.0119022	87883	131	167	298
BLR	2002	country	4_Eur	Belarus	437	523	960	0.0049322	0.0059029	0.0108351	88601	121	158	280
BLR	2003	country	4_Eur	Belarus	401	491	892	0.0044541	0.0054538	0.0099079	90029	115	151	266
BLR	2004	country	4_Eur	Belarus	367	469	836	0.0039863	0.0050942	0.0090806	92065	106	148	254
BLR	2005	country	4_Eur	Belarus	330	457	787	0.003485	0.0048262	0.0083112	94691	97	146	243
BLR	2006	country	4_Eur	Belarus	292	450	742	0.0029919	0.0046109	0.0076028	97596	86	148	234
BLR	2007	country	4_Eur	Belarus	257	443	700	0.0025493	0.0043942	0.0069435	100814	77	151	228
BLR	2008	country	4_Eur	Belarus	228	431	659	0.0021978	0.0041546	0.0063524	103740	68	149	218
BLR	2009	country	4_Eur	Belarus	210	412	622	0.0019588	0.003843	0.0058019	107207	64	145	209
BLR	2010	country	4_Eur	Belarus	202	386	588	0.0018433	0.0035224	0.0053657	109585	62	138	199
BLR	2011	country	4_Eur	Belarus	202	356	558	0.0018102	0.0031902	0.0050004	111591	62	128	191
BLR	2012	country	4_Eur	Belarus	204	327	531	0.0018015	0.0028877	0.0046892	113240	62	119	182
BLR	2013	country	4_Eur	Belarus	204	303	507	0.0017802	0.0026441	0.0044243	114595	63	113	175
BLR	2014	country	4_Eur	Belarus	199	285	484	0.0017304	0.0024782	0.0042086	115004	61	108	169
BLR	2015	country	4_Eur	Belarus	188	273	461	0.0016422	0.0023847	0.004027	114478	59	105	163
BLR	2016	country	4_Eur	Belarus	174	263	437	0.0015299	0.0023125	0.0038424	113731	54	102	156
BLR	2017	country	4_Eur	Belarus	160	251	411	0.0014137	0.0022177	0.0036313	113181	50	99	149
BLR	2018	country	4_Eur	Belarus	146	239	385	0.0013064	0.0021386	0.0034451	111754	46	96	141
BLR	2019	country	4_Eur	Belarus	133	227	360	0.0012143	0.0020726	0.0032869	109525	42	91	133
BLZ	2000	country	2_Amr	Belize	88	83	171	0.0120355	0.0113517	0.0233873	7312	20	9	30
BLZ	2001	country	2_Amr	Belize	86	81	167	0.0115823	0.0109089	0.0224912	7425	18	11	28
BLZ	2002	country	2_Amr	Belize	83	81	164	0.0111098	0.0108421	0.021952	7471	13	9	23
BLZ	2003	country	2_Amr	Belize	80	82	162	0.0106016	0.0108667	0.0214683	7546	18	2	20
BLZ	2004	country	2_Amr	Belize	75	84	159	0.0100238	0.0112267	0.0212506	7482	16	12	28
BLZ	2005	country	2_Amr	Belize	70	86	156	0.009403	0.0115523	0.0209553	7444	10	14	24
BLZ	2006	country	2_Amr	Belize	66	88	154	0.0088431	0.0117908	0.0206339	7463	14	9	22
BLZ	2007	country	2_Amr	Belize	64	87	151	0.0085088	0.0115667	0.0200755	7522	11	21	33
BLZ	2008	country	2_Amr	Belize	64	84	148	0.0085	0.0111562	0.0196562	7529	14	8	21
BLZ	2009	country	2_Amr	Belize	66	79	145	0.0087987	0.0105318	0.0193305	7501	15	12	28
BLZ	2010	country	2_Amr	Belize	71	71	142	0.0093484	0.0093484	0.0186968	7595	19	17	35
BLZ	2011	country	2_Amr	Belize	76	63	139	0.0099496	0.0082477	0.0181974	7638	19	19	39
BLZ	2012	country	2_Amr	Belize	80	55	135	0.010352	0.007117	0.0174689	7728	13	10	24
BLZ	2013	country	2_Amr	Belize	81	49	130	0.0104564	0.0063255	0.0167819	7746	20	13	34
BLZ	2014	country	2_Amr	Belize	80	45	125	0.0103189	0.0058044	0.0161233	7753	14	15	28
BLZ	2015	country	2_Amr	Belize	78	41	119	0.0100103	0.0052618	0.0152721	7792	16	8	24
BLZ	2016	country	2_Amr	Belize	76	38	114	0.0095908	0.0047954	0.0143862	7924	15	9	24
BLZ	2017	country	2_Amr	Belize	72	36	108	0.0091035	0.0045517	0.0136552	7909	13	9	23
BLZ	2018	country	2_Amr	Belize	69	34	103	0.0086079	0.0042416	0.0128495	8016	13	9	22
BLZ	2019	country	2_Amr	Belize	65	33	98	0.0081374	0.0041313	0.0122687	7988	12	8	21

iso3	year	level	whoreg6	whoname	nnd	pnd	u5d	nmr	pnmr	u5mr	Livebirths	Neonatal birth defects deaths	1-59 month birth defects deaths	Under five birth defects deaths
BOL	2000	country	2_Amr	Bolivia (Plurination	7427	11602	19029	0.0290939	0.0454487	0.0745426	255277	584	825	1410
BOL	2001	country	2_Amr	Bolivia (Plurination	7197	10804	18001	0.0281416	0.0422456	0.0703872	255743	586	913	1499
BOL	2002	country	2_Amr	Bolivia (Plurination	7002	9987	16989	0.0273648	0.0390306	0.0663954	255876	575	966	1541
BOL	2003	country	2_Amr	Bolivia (Plurination	6840	9180	16020	0.0267439	0.0358931	0.0626369	255760	586	1046	1632
BOL	2004	country	2_Amr	Bolivia (Plurination	6680	8405	15085	0.0261543	0.0329082	0.0590625	255407	572	888	1460
BOL	2005	country	2_Amr	Bolivia (Plurination	6501	7680	14181	0.0255112	0.0301378	0.055649	254829	572	833	1405
BOL	2006	country	2_Amr	Bolivia (Plurination	6293	7029	13322	0.0247594	0.0276552	0.0524146	254166	553	780	1332
BOL	2007	country	2_Amr	Bolivia (Plurination	6062	6440	12502	0.0239191	0.0254105	0.0493296	253438	544	714	1258
BOL	2008	country	2_Amr	Bolivia (Plurination	5821	5917	11738	0.0230353	0.0234152	0.0464505	252699	532	788	1320
BOL	2009	country	2_Amr	Bolivia (Plurination	5569	5455	11024	0.0220938	0.0216415	0.0437352	252062	517	774	1291
BOL	2010	country	2_Amr	Bolivia (Plurination	5318	5036	10354	0.0211507	0.0200292	0.0411799	251433	508	668	1176
BOL	2011	country	2_Amr	Bolivia (Plurination	5074	4664	9738	0.0202303	0.0185956	0.038826	250812	490	746	1237
BOL	2012	country	2_Amr	Bolivia (Plurination	4837	4323	9160	0.0193338	0.0172793	0.0366132	250183	493	654	1147
BOL	2013	country	2_Amr	Bolivia (Plurination	4617	3997	8614	0.0185081	0.0160227	0.0345309	249458	495	542	1037
BOL	2014	country	2_Amr	Bolivia (Plurination	4411	3702	8113	0.0177369	0.014886	0.0326228	248691	495	514	1008
BOL	2015	country	2_Amr	Bolivia (Plurination	4231	3441	7672	0.0170592	0.013874	0.0309332	248018	503	500	1003
BOL	2016	country	2_Amr	Bolivia (Plurination	4082	3198	7280	0.0164948	0.0129227	0.0294175	247472	520	431	951
BOL	2017	country	2_Amr	Bolivia (Plurination	3907	3044	6951	0.0158125	0.0123197	0.0281322	247083	524	359	883
BOL	2018	country	2_Amr	Bolivia (Plurination	3742	2909	6651	0.0151501	0.0117775	0.0269276	246995	515	352	867
BOL	2019	country	2_Amr	Bolivia (Plurination	3598	2801	6399	0.0145618	0.0113362	0.025898	247085	508	352	860
BRA	2000	country	2_Amr	Brazil	63185	58880	122065	0.0179792	0.0167542	0.0347334	3514344	8545	8813	17357
BRA	2001	country	2_Amr	Brazil	60028	52763	112791	0.017345	0.0152458	0.0325908	3460822	8615	7734	16349
BRA	2002	country	2_Amr	Brazil	56725	47075	103800	0.016683	0.0138449	0.030528	3400158	8273	7649	15922
BRA	2003	country	2_Amr	Brazil	52967	42320	95287	0.0158783	0.0126866	0.0285649	3335812	8109	7098	15207
BRA	2004	country	2_Amr	Brazil	48888	38493	87381	0.0149483	0.0117699	0.0267182	3270464	7945	6773	14718
BRA	2005	country	2_Amr	Brazil	45195	34972	80167	0.0140902	0.010903	0.0249932	3207554	7246	6380	13627
BRA	2006	country	2_Amr	Brazil	41966	31796	73762	0.013322	0.0100936	0.0234156	3150118	7159	6202	13361
BRA	2007	country	2_Amr	Brazil	39220	29019	68239	0.0126519	0.0093612	0.0220131	3099926	6791	6094	12886
BRA	2008	country	2_Amr	Brazil	37002	26478	63480	0.0121	0.0086585	0.0207585	3058024	6758	5768	12527
BRA	2009	country	2_Amr	Brazil	35179	24351	59530	0.0116289	0.0080495	0.0196784	3025142	6449	5393	11841
BRA	2010	country	2_Amr	Brazil	33515	22786	56301	0.0111662	0.0075916	0.0187579	3001462	6426	5280	11706
BRA	2011	country	2_Amr	Brazil	31952	21689	53641	0.0106991	0.0072625	0.0179616	2986429	6250	5299	11549
BRA	2012	country	2_Amr	Brazil	30649	20806	51455	0.0102939	0.006988	0.0172819	2977393	6051	5233	11284
BRA	2013	country	2_Amr	Brazil	29693	19941	49634	0.0099927	0.0067108	0.0167035	2971471	5867	5089	10956
BRA	2014	country	2_Amr	Brazil	28855	19165	48020	0.0097265	0.0064602	0.0161867	2966633	6061	5055	11116
BRA	2015	country	2_Amr	Brazil	27799	18701	46500	0.0093909	0.0063175	0.0157083	2960212	5903	5219	11122
BRA	2016	country	2_Amr	Brazil	28463	19823	48286	0.0096478	0.0067192	0.016367	2950207	7780	6266	14046
BRA	2017	country	2_Amr	Brazil	24969	18534	43503	0.0085057	0.0063136	0.0148193	2935563	5323	5035	10357
BRA	2018	country	2_Amr	Brazil	23728	18231	41959	0.0081381	0.0062528	0.0143909	2915671	5083	4950	10033
BRA	2019	country	2_Amr	Brazil	22736	17693	40429	0.0078661	0.0061214	0.0139875	2890368	4885	4805	9689

iso3	year	level	whoreg6	whoname	nnd	pnd	u5d	nmr	pnmr	u5mr	Livebirths	Neonatal birth defects deaths	1-59 month birth defects deaths	Under five birth defects deaths
BRB	2000	country	2_Amr	Barbados	35	22	57	0.0092714	0.0058277	0.0150991	3775	3	5	8
BRB	2001	country	2_Amr	Barbados	35	23	58	0.0093603	0.0061511	0.0155114	3739	2	0	2
BRB	2002	country	2_Amr	Barbados	35	23	58	0.0094874	0.0062346	0.015722	3689	8	7	15
BRB	2003	country	2_Amr	Barbados	36	22	58	0.0096369	0.0058892	0.0155261	3736	3	3	6
BRB	2004	country	2_Amr	Barbados	36	22	58	0.00979	0.0059828	0.0157727	3677	5	3	8
BRB	2005	country	2_Amr	Barbados	36	21	57	0.0099398	0.0057982	0.0157381	3622	12	3	15
BRB	2006	country	2_Amr	Barbados	36	20	56	0.0100345	0.0055747	0.0156092	3588	3	4	7
BRB	2007	country	2_Amr	Barbados	35	20	55	0.010076	0.0057577	0.0158337	3474	2	0	2
BRB	2008	country	2_Amr	Barbados	35	18	53	0.0100646	0.0051761	0.0152407	3478	11	5	16
BRB	2009	country	2_Amr	Barbados	34	18	52	0.0100224	0.005306	0.0153283	3392	8	0	8
BRB	2010	country	2_Amr	Barbados	33	17	50	0.0099591	0.0051305	0.0150896	3314	9	2	11
BRB	2011	country	2_Amr	Barbados	32	17	49	0.0098873	0.0052527	0.01514	3236	6	1	7
BRB	2012	country	2_Amr	Barbados	31	16	47	0.0097986	0.0050573	0.0148559	3164	9	2	11
BRB	2013	country	2_Amr	Barbados	30	16	46	0.0096831	0.0051643	0.0148474	3098	10	7	18
BRB	2014	country	2_Amr	Barbados	30	15	45	0.0095324	0.0047662	0.0142986	3147	8	3	11
BRB	2015	country	2_Amr	Barbados	29	14	43	0.0093386	0.0045083	0.0138469	3105	9	3	12
BRB	2016	country	2_Amr	Barbados	28	14	42	0.0091009	0.0045505	0.0136514	3077	9	3	12
BRB	2017	country	2_Amr	Barbados	27	14	41	0.0088618	0.004595	0.0134569	3047	8	3	11
BRB	2018	country	2_Amr	Barbados	26	14	40	0.0086132	0.0046379	0.013251	3019	8	3	11
BRB	2019	country	2_Amr	Barbados	26	13	39	0.0083706	0.0041853	0.0125559	3106	8	3	11
BRN	2000	country	6_Wpr	Brunei Darussalam	38	40	78	0.0049635	0.0052247	0.0101882	7656	14	13	27
BRN	2001	country	6_Wpr	Brunei Darussalam	37	39	76	0.0049729	0.0052417	0.0102145	7440	14	16	30
BRN	2002	country	6_Wpr	Brunei Darussalam	37	37	74	0.0049864	0.0049864	0.0099728	7420	14	16	30
BRN	2003	country	6_Wpr	Brunei Darussalam	37	34	71	0.0051274	0.0047116	0.009839	7216	14	8	22
BRN	2004	country	6_Wpr	Brunei Darussalam	36	33	69	0.0051052	0.0046798	0.009785	7052	13	7	20
BRN	2005	country	6_Wpr	Brunei Darussalam	35	32	67	0.0050607	0.004627	0.0096877	6916	13	12	25
BRN	2006	country	6_Wpr	Brunei Darussalam	34	32	66	0.0050105	0.0047157	0.0097262	6786	12	4	16
BRN	2007	country	6_Wpr	Brunei Darussalam	33	32	65	0.0048233	0.0046771	0.0095004	6842	12	11	23
BRN	2008	country	6_Wpr	Brunei Darussalam	32	32	64	0.0048018	0.0048018	0.0096036	6664	12	9	21
BRN	2009	country	6_Wpr	Brunei Darussalam	32	32	64	0.0047963	0.0047963	0.0095925	6672	11	7	19
BRN	2010	country	6_Wpr	Brunei Darussalam	33	32	65	0.0049433	0.0047935	0.0097369	6676	12	10	22
BRN	2011	country	6_Wpr	Brunei Darussalam	33	34	67	0.0048066	0.0049522	0.0097588	6866	15	9	24
BRN	2012	country	6_Wpr	Brunei Darussalam	34	34	68	0.0049663	0.0049663	0.0099326	6846	8	1	9
BRN	2013	country	6_Wpr	Brunei Darussalam	34	36	70	0.0048712	0.0051577	0.0100289	6980	12	7	19
BRN	2014	country	6_Wpr	Brunei Darussalam	34	37	71	0.0049583	0.0053958	0.0103541	6857	10	12	21
BRN	2015	country	6_Wpr	Brunei Darussalam	35	37	72	0.0050915	0.0053824	0.010474	6874	16	13	28
BRN	2016	country	6_Wpr	Brunei Darussalam	36	37	73	0.0054054	0.0055555	0.0109609	6660	9	7	16
BRN	2017	country	6_Wpr	Brunei Darussalam	36	37	73	0.0055922	0.0057476	0.0113398	6437	12	10	22
BRN	2018	country	6_Wpr	Brunei Darussalam	37	36	73	0.0057577	0.0056021	0.0113598	6426	12	10	23
BRN	2019	country	6_Wpr	Brunei Darussalam	38	35	73	0.0060305	0.0055544	0.0115849	6301	11	10	21

iso3	year	level	whoreg6	whoname	nnd	pnd	u5d	nmr	pnmr	u5mr	Livebirths	Neonatal birth defects deaths	1-59 month birth defects deaths	Under five birth defects deaths
BTN	2000	country	3_Sear	Bhutan	505	765	1270	0.0316704	0.0479852	0.0796557	15945	29	34	63
BTN	2001	country	3_Sear	Bhutan	480	712	1192	0.0306891	0.045534	0.0762231	15641	29	33	62
BTN	2002	country	3_Sear	Bhutan	456	621	1077	0.0297077	0.0404728	0.0701805	15350	29	32	61
BTN	2003	country	3_Sear	Bhutan	432	560	992	0.028686	0.0371896	0.0658756	15060	29	39	68
BTN	2004	country	3_Sear	Bhutan	410	508	918	0.0277181	0.0343471	0.0620653	14792	28	37	65
BTN	2005	country	3_Sear	Bhutan	389	459	848	0.0267568	0.0315781	0.0583349	14538	28	41	69
BTN	2006	country	3_Sear	Bhutan	369	414	783	0.0257779	0.0288879	0.0546658	14315	28	36	64
BTN	2007	country	3_Sear	Bhutan	350	370	720	0.024898	0.0263208	0.0512188	14057	28	39	67
BTN	2008	country	3_Sear	Bhutan	332	333	665	0.0240251	0.0240975	0.0481226	13819	27	40	67
BTN	2009	country	3_Sear	Bhutan	314	297	611	0.0231223	0.0219066	0.0450289	13580	27	33	60
BTN	2010	country	3_Sear	Bhutan	297	271	568	0.022237	0.0202759	0.0425129	13356	26	32	59
BTN	2011	country	3_Sear	Bhutan	281	248	529	0.0213634	0.0188238	0.0401871	13153	26	31	56
BTN	2012	country	3_Sear	Bhutan	268	228	496	0.0205827	0.0175281	0.0381108	13021	25	30	55
BTN	2013	country	3_Sear	Bhutan	258	213	471	0.0198893	0.0164041	0.0362933	12972	25	29	54
BTN	2014	country	3_Sear	Bhutan	249	199	448	0.0192216	0.0153742	0.0345958	12954	27	29	56
BTN	2015	country	3_Sear	Bhutan	242	187	429	0.018655	0.0143866	0.0330416	12972	29	27	55
BTN	2016	country	3_Sear	Bhutan	236	175	411	0.0181246	0.0134424	0.031567	13021	30	25	55
BTN	2017	country	3_Sear	Bhutan	229	169	398	0.0176093	0.0130155	0.0306247	13005	32	25	56
BTN	2018	country	3_Sear	Bhutan	223	161	384	0.0171062	0.0123525	0.0294587	13036	32	24	56
BTN	2019	country	3_Sear	Bhutan	215	155	370	0.0165691	0.0119142	0.0284832	12976	32	24	55
BWA	2000	country	1_Afr	Botswana	278	3528	3806	0.0057812	0.0733648	0.079146	48087	25	106	131
BWA	2001	country	1_Afr	Botswana	272	2857	3129	0.0055716	0.0585184	0.0640899	48819	25	110	135
BWA	2002	country	1_Afr	Botswana	276	2654	2930	0.0055704	0.0535707	0.0591411	49547	26	117	143
BWA	2003	country	1_Afr	Botswana	286	2505	2791	0.0056808	0.0497564	0.0554372	50345	29	120	149
BWA	2004	country	1_Afr	Botswana	304	2311	2615	0.0059557	0.0452774	0.0512331	51044	33	123	156
BWA	2005	country	1_Afr	Botswana	329	1989	2318	0.0063529	0.0383975	0.0447504	51787	39	122	162
BWA	2006	country	1_Afr	Botswana	361	1668	2029	0.0068814	0.0317989	0.0386804	52460	45	121	166
BWA	2007	country	1_Afr	Botswana	401	1533	1934	0.007547	0.0288605	0.0364076	53133	54	121	175
BWA	2008	country	1_Afr	Botswana	449	1486	1935	0.0083406	0.0276073	0.0359479	53833	61	119	180
BWA	2009	country	1_Afr	Botswana	508	1417	1925	0.0093254	0.0260045	0.0353299	54475	70	120	191
BWA	2010	country	1_Afr	Botswana	578	1402	1980	0.0104835	0.0254248	0.0359083	55134	81	121	201
BWA	2011	country	1_Afr	Botswana	660	1407	2067	0.0118573	0.0252715	0.0371288	55662	88	127	214
BWA	2012	country	1_Afr	Botswana	752	1336	2088	0.0134045	0.0238115	0.037216	56101	95	139	234
BWA	2013	country	1_Afr	Botswana	847	1254	2101	0.0150214	0.0222336	0.0372551	56386	101	148	248
BWA	2014	country	1_Afr	Botswana	915	1262	2177	0.0161795	0.0223218	0.0385013	56553	104	160	264
BWA	2015	country	1_Afr	Botswana	963	1356	2319	0.0170164	0.023967	0.0409834	56593	103	166	270
BWA	2016	country	1_Afr	Botswana	990	1472	2462	0.0175265	0.0260625	0.0435891	56486	103	168	270
BWA	2017	country	1_Afr	Botswana	1004	1457	2461	0.0178353	0.0258825	0.0437178	56293	104	170	274
BWA	2018	country	1_Afr	Botswana	1007	1401	2408	0.0179668	0.0249901	0.0429569	56048	102	171	273
BWA	2019	country	1_Afr	Botswana	999	1321	2320	0.0179206	0.023699	0.0416196	55746	101	174	275

iso3	year	level	whoreg6	whoname	nnd	pnd	u5d	nmr	pnmr	u5mr	Livebirths	Neonatal birth defects deaths	1-59 month birth defects deaths	Under five birth defects deaths
CAF	2000	country	1_Afr	Central African Rep	7426	17878	25304	0.0493981	0.1189267	0.1683248	150330	262	17	279
CAF	2001	country	1_Afr	Central African Rep	7562	18001	25563	0.0492647	0.1172738	0.1665385	153497	267	17	284
CAF	2002	country	1_Afr	Central African Rep	7689	18299	25988	0.0491219	0.1169072	0.1660291	156529	275	20	295
CAF	2003	country	1_Afr	Central African Rep	7811	18164	25975	0.0490078	0.1139651	0.1629729	159383	282	23	305
CAF	2004	country	1_Afr	Central African Rep	7905	18980	26885	0.0488019	0.117176	0.1659779	161982	285	24	309
CAF	2005	country	1_Afr	Central African Rep	7999	18275	26274	0.0487041	0.1112708	0.1599749	164237	291	28	319
CAF	2006	country	1_Afr	Central African Rep	8057	17337	25394	0.0485218	0.1044068	0.1529287	166049	297	18	315
CAF	2007	country	1_Afr	Central African Rep	8089	17269	25358	0.0483241	0.1031645	0.1514885	167391	298	18	316
CAF	2008	country	1_Afr	Central African Rep	8075	17094	25169	0.047992	0.1015954	0.1495873	168257	307	32	339
CAF	2009	country	1_Afr	Central African Rep	7996	16595	24591	0.0473993	0.0983749	0.1457742	168694	308	25	332
CAF	2010	country	1_Afr	Central African Rep	7896	16257	24153	0.0468078	0.0963722	0.14318	168690	307	27	335
CAF	2011	country	1_Afr	Central African Rep	7756	16538	24294	0.0460936	0.0982824	0.1443759	168266	301	29	330
CAF	2012	country	1_Afr	Central African Rep	7580	15317	22897	0.0452324	0.0914026	0.136635	167579	298	32	330
CAF	2013	country	1_Afr	Central African Rep	7415	15503	22918	0.0444672	0.0929707	0.1374379	166752	289	18	307
CAF	2014	country	1_Afr	Central African Rep	7251	14230	21481	0.0436968	0.0857569	0.1294537	165939	289	37	325
CAF	2015	country	1_Afr	Central African Rep	7091	13674	20765	0.0428934	0.0827125	0.1256059	165317	280	40	319
CAF	2016	country	1_Afr	Central African Rep	6954	13181	20135	0.0421243	0.0798453	0.1219696	165083	278	43	321
CAF	2017	country	1_Afr	Central African Rep	6846	12534	19380	0.0414175	0.0758292	0.1172467	165293	278	67	346
CAF	2018	country	1_Afr	Central African Rep	6752	12106	18858	0.0406683	0.0729152	0.1135835	166026	271	47	318
CAF	2019	country	1_Afr	Central African Rep	6646	12800	19446	0.0397348	0.0765294	0.1162642	167259	270	48	317
CAN	2000	country	2_Amr	Canada	1240	849	2089	0.0037314	0.0025548	0.0062862	332315	375	255	631
CAN	2001	country	2_Amr	Canada	1249	805	2054	0.0037779	0.0024349	0.0062128	330607	377	241	618
CAN	2002	country	2_Amr	Canada	1276	765	2041	0.0038462	0.0023059	0.006152	331760	386	229	615
CAN	2003	country	2_Amr	Canada	1308	736	2044	0.0039018	0.0021955	0.0060974	335227	397	221	617
CAN	2004	country	2_Amr	Canada	1332	725	2057	0.00391	0.0021282	0.0060382	340666	401	217	618
CAN	2005	country	2_Amr	Canada	1347	727	2074	0.0038745	0.0020912	0.0059657	347653	401	218	619
CAN	2006	country	2_Amr	Canada	1360	731	2091	0.00383	0.0020586	0.0058886	355093	403	219	622
CAN	2007	country	2_Amr	Canada	1374	731	2105	0.003787	0.0020148	0.0058018	362818	412	219	631
CAN	2008	country	2_Amr	Canada	1389	725	2114	0.003759	0.001962	0.005721	369517	416	217	633
CAN	2009	country	2_Amr	Canada	1406	712	2118	0.0037498	0.0018989	0.0056487	374953	415	213	628
CAN	2010	country	2_Amr	Canada	1420	698	2118	0.0037475	0.0018421	0.0055895	378922	419	209	629
CAN	2011	country	2_Amr	Canada	1422	690	2112	0.0037253	0.0018077	0.005533	381710	416	207	623
CAN	2012	country	2_Amr	Canada	1411	692	2103	0.0036792	0.0018044	0.0054837	383503	417	207	624
CAN	2013	country	2_Amr	Canada	1392	695	2087	0.003619	0.0018069	0.0054258	384640	413	208	621
CAN	2014	country	2_Amr	Canada	1374	690	2064	0.0035681	0.0017918	0.0053599	385080	408	207	615
CAN	2015	country	2_Amr	Canada	1357	677	2034	0.0035182	0.0017552	0.0052734	385707	400	203	603
CAN	2016	country	2_Amr	Canada	1343	656	1999	0.0034797	0.0016997	0.0051794	385951	398	197	595
CAN	2017	country	2_Amr	Canada	1328	632	1960	0.0034411	0.0016376	0.0050787	385924	391	189	580
CAN	2018	country	2_Amr	Canada	1310	608	1918	0.0033925	0.0015745	0.004967	386148	381	182	563
CAN	2019	country	2_Amr	Canada	1285	588	1873	0.003326	0.0015219	0.004848	386348	381	176	557

iso3	year	level	whoreg6	whoname	nnd	pnd	u5d	nmr	pnmr	u5mr	Livebirths	Neonatal birth defects deaths	1-59 month birth defects deaths	Under five birth defects deaths
CHE	2000	country	4_Eur	Switzerland	264	171	435	0.003478	0.0022528	0.0057308	75905	79	51	130
CHE	2001	country	4_Eur	Switzerland	259	162	421	0.0034655	0.0021676	0.0056331	74736	77	47	124
CHE	2002	country	4_Eur	Switzerland	254	153	407	0.0034332	0.002068	0.0055012	73983	76	48	124
CHE	2003	country	4_Eur	Switzerland	248	146	394	0.0033664	0.0019818	0.0053482	73670	75	48	123
CHE	2004	country	4_Eur	Switzerland	244	139	383	0.0033224	0.0018927	0.0052151	73440	74	48	122
CHE	2005	country	4_Eur	Switzerland	243	130	373	0.0032935	0.001762	0.0050555	73781	72	50	122
CHE	2006	country	4_Eur	Switzerland	243	124	367	0.0032596	0.0016633	0.0049229	74549	73	37	111
CHE	2007	country	4_Eur	Switzerland	246	116	362	0.0032457	0.0015305	0.0047761	75793	74	42	117
CHE	2008	country	4_Eur	Switzerland	249	110	359	0.0032356	0.0014294	0.0046649	76957	75	34	110
CHE	2009	country	4_Eur	Switzerland	250	108	358	0.0031879	0.0013772	0.0045651	78422	75	31	106
CHE	2010	country	4_Eur	Switzerland	251	107	358	0.0031478	0.0013419	0.0044897	79739	76	42	118
CHE	2011	country	4_Eur	Switzerland	252	107	359	0.0031054	0.0013185	0.0044239	81150	78	43	121
CHE	2012	country	4_Eur	Switzerland	253	108	361	0.0030687	0.00131	0.0043787	82445	77	40	117
CHE	2013	country	4_Eur	Switzerland	255	109	364	0.0030479	0.0013028	0.0043508	83663	78	44	122
CHE	2014	country	4_Eur	Switzerland	256	109	365	0.0030159	0.0012841	0.0043	84883	77	34	111
CHE	2015	country	4_Eur	Switzerland	256	110	366	0.0029856	0.0012829	0.0042685	85744	77	44	121
CHE	2016	country	4_Eur	Switzerland	255	110	365	0.0029449	0.0012703	0.0042152	86590	76	52	128
CHE	2017	country	4_Eur	Switzerland	253	109	362	0.0029029	0.0012506	0.0041535	87155	76	43	119
CHE	2018	country	4_Eur	Switzerland	249	109	358	0.0028418	0.001244	0.0040858	87620	75	43	117
CHE	2019	country	4_Eur	Switzerland	244	109	353	0.0027672	0.0012362	0.0040033	88177	73	43	115
CHL	2000	country	2_Amr	Chile	1450	1364	2814	0.0057086	0.00537	0.0110786	254004	555	387	942
CHL	2001	country	2_Amr	Chile	1356	1243	2599	0.0054304	0.0049779	0.0104083	249704	482	366	848
CHL	2002	country	2_Amr	Chile	1308	1137	2445	0.0053053	0.0046117	0.009917	246547	511	397	908
CHL	2003	country	2_Amr	Chile	1300	1044	2344	0.0053146	0.0042681	0.0095827	244608	526	351	878
CHL	2004	country	2_Amr	Chile	1311	961	2272	0.0053833	0.0039461	0.0093294	243531	494	341	834
CHL	2005	country	2_Amr	Chile	1327	891	2218	0.0054483	0.0036582	0.0091066	243560	487	333	819
CHL	2006	country	2_Amr	Chile	1336	849	2185	0.0054683	0.003475	0.0089434	244315	499	284	783
CHL	2007	country	2_Amr	Chile	1331	847	2178	0.0054206	0.0034495	0.0088701	245543	476	304	780
CHL	2008	country	2_Amr	Chile	1320	863	2183	0.0053412	0.003492	0.0088332	247135	476	292	768
CHL	2009	country	2_Amr	Chile	1316	865	2181	0.005294	0.0034797	0.0087737	248583	508	316	824
CHL	2010	country	2_Amr	Chile	1324	835	2159	0.0053091	0.0033483	0.0086574	249382	483	305	788
CHL	2011	country	2_Amr	Chile	1330	796	2126	0.0053318	0.0031911	0.0085229	249445	507	345	851
CHL	2012	country	2_Amr	Chile	1321	768	2089	0.0053139	0.0030894	0.0084033	248593	513	314	827
CHL	2013	country	2_Amr	Chile	1298	749	2047	0.0052576	0.0030338	0.0082914	246882	541	292	833
CHL	2014	country	2_Amr	Chile	1267	724	1991	0.0051845	0.0029626	0.0081471	244382	486	290	775
CHL	2015	country	2_Amr	Chile	1230	690	1920	0.0050998	0.0028609	0.0079607	241186	463	277	740
CHL	2016	country	2_Amr	Chile	1188	652	1840	0.0049974	0.0027427	0.0077401	237722	514	274	788
CHL	2017	country	2_Amr	Chile	1141	619	1760	0.0048656	0.0026396	0.0075052	234503	454	252	706
CHL	2018	country	2_Amr	Chile	1092	591	1683	0.0047152	0.0025519	0.0072672	231589	440	241	680
CHL	2019	country	2_Amr	Chile	1044	568	1612	0.0045556	0.0024785	0.0070341	229170	429	231	660

iso3	year	level	whoreg6	whoname	nnd	pnd	u5d	nmr	pnmr	u5mr	Livebirths	Neonatal birth defects deaths	1-59 month birth defects deaths	Under five birth defects deaths
CHN	2000	country	6_Wpr	China	368525	283613	652138	0.0209709	0.016139	0.0371099	17600000	44205	35785	79990
CHN	2001	country	6_Wpr	China	343637	254109	597746	0.019791	0.0146348	0.0344259	17400000	43905	36514	80419
CHN	2002	country	6_Wpr	China	319321	225298	544619	0.018556	0.0130922	0.0316482	17200000	43189	35544	78733
CHN	2003	country	6_Wpr	China	294328	200324	494652	0.0171971	0.0117046	0.0289017	17100000	41510	32731	74242
CHN	2004	country	6_Wpr	China	265423	183671	449094	0.0155434	0.010756	0.0262994	17100000	40258	31984	72242
CHN	2005	country	6_Wpr	China	239226	169557	408783	0.014001	0.0099235	0.0239245	17100000	39154	30623	69777
CHN	2006	country	6_Wpr	China	215662	158053	373715	0.0125826	0.0092214	0.021804	17100000	37082	30332	67414
CHN	2007	country	6_Wpr	China	195041	148686	343727	0.0113235	0.0086323	0.0199558	17200000	34420	30125	64545
CHN	2008	country	6_Wpr	China	176975	140990	317965	0.0102146	0.0081376	0.0183523	17300000	31220	28172	59392
CHN	2009	country	6_Wpr	China	160947	134210	295157	0.009235	0.0077008	0.0169358	17400000	28561	28071	56632
CHN	2010	country	6_Wpr	China	147011	127267	274278	0.0083927	0.0072655	0.0156582	17500000	26618	27815	54433
CHN	2011	country	6_Wpr	China	134420	120637	255057	0.0076464	0.0068623	0.0145087	17600000	24923	26523	51445
CHN	2012	country	6_Wpr	China	123143	113646	236789	0.0069924	0.0064532	0.0134456	17600000	23176	24872	48048
CHN	2013	country	6_Wpr	China	112714	106806	219520	0.0064024	0.0060668	0.0124692	17600000	21176	23182	44358
CHN	2014	country	6_Wpr	China	103120	99816	202936	0.005874	0.0056858	0.0115598	17600000	19022	21386	40408
CHN	2015	country	6_Wpr	China	93912	93254	187166	0.0053808	0.0053431	0.0107239	17500000	16901	20197	37098
CHN	2016	country	6_Wpr	China	85305	86815	172120	0.0049327	0.00502	0.0099528	17300000	15352	18850	34202
CHN	2017	country	6_Wpr	China	77398	80323	157721	0.0045307	0.0047019	0.0092326	17100000	13929	17507	31435
CHN	2018	country	6_Wpr	China	70170	74162	144332	0.0041691	0.0044063	0.0085755	16800000	12628	16184	28812
CHN	2019	country	6_Wpr	China	63895	68361	132256	0.0038615	0.0041314	0.007993	16500000	11499	14929	26428
CIV	2000	country	1_Afr	Côte d'Ivoire	30334	62352	92686	0.0451165	0.0927379	0.1378544	672349	1405	111	1516
CIV	2001	country	1_Afr	Côte d'Ivoire	30504	62714	93218	0.0445905	0.0916748	0.1362653	684092	1412	115	1526
CIV	2002	country	1_Afr	Côte d'Ivoire	30610	62729	93339	0.0440801	0.0903329	0.134413	694418	1458	116	1574
CIV	2003	country	1_Afr	Côte d'Ivoire	30609	61663	92272	0.0435068	0.0876458	0.1311526	703546	1479	121	1600
CIV	2004	country	1_Afr	Côte d'Ivoire	30518	59881	90399	0.042874	0.0841252	0.1269992	711806	1483	245	1729
CIV	2005	country	1_Afr	Côte d'Ivoire	30400	57068	87468	0.0422419	0.0792979	0.1215397	719665	1513	322	1835
CIV	2006	country	1_Afr	Côte d'Ivoire	30258	54890	85148	0.0415819	0.0754319	0.1170138	727672	1498	258	1757
CIV	2007	country	1_Afr	Côte d'Ivoire	30113	53878	83991	0.0408936	0.0731664	0.1140601	736374	1536	153	1689
CIV	2008	country	1_Afr	Côte d'Ivoire	30007	52608	82615	0.040217	0.0705087	0.1107258	746127	1595	215	1810
CIV	2009	country	1_Afr	Côte d'Ivoire	29932	50645	80577	0.0395289	0.0668831	0.106412	757218	1603	180	1782
CIV	2010	country	1_Afr	Côte d'Ivoire	29892	49752	79644	0.0388304	0.0646286	0.103459	769810	1604	246	1850
CIV	2011	country	1_Afr	Côte d'Ivoire	29901	49141	79042	0.038148	0.062695	0.100843	783815	1660	195	1855
CIV	2012	country	1_Afr	Côte d'Ivoire	29958	47531	77489	0.037498	0.0594932	0.0969913	798923	1697	766	2463
CIV	2013	country	1_Afr	Côte d'Ivoire	30009	46611	76620	0.0368325	0.0572095	0.094042	814743	1718	569	2287
CIV	2014	country	1_Afr	Côte d'Ivoire	30080	45610	75690	0.0361969	0.0548847	0.0910816	831010	1773	488	2261
CIV	2015	country	1_Afr	Côte d'Ivoire	30142	44146	74288	0.0355642	0.0520874	0.0876515	847539	1823	618	2441
CIV	2016	country	1_Afr	Côte d'Ivoire	30175	43715	73890	0.0349222	0.0505919	0.0855141	864063	1829	608	2437
CIV	2017	country	1_Afr	Côte d'Ivoire	30182	43092	73274	0.0342793	0.0489418	0.0832212	880472	1889	567	2456
CIV	2018	country	1_Afr	Côte d'Ivoire	30190	42266	72456	0.0336684	0.0471353	0.0808037	896687	1901	716	2617
CIV	2019	country	1_Afr	Côte d'Ivoire	30156	40084	70240	0.0330441	0.0439233	0.0769674	912600	1886	725	2611

iso3	year	level	whoreg6	whoname	nnd	pnd	u5d	nmr	pnmr	u5mr	Livebirths	Neonatal birth defects deaths	1-59 month birth defects deaths	Under five birth defects deaths
CMR	2000	country	1_Afr	Cameroon	22647	69252	91899	0.0351816	0.1075819	0.1427635	643717	1168	223	1392
CMR	2001	country	1_Afr	Cameroon	22630	71698	94328	0.0343301	0.108767	0.1430971	659188	1197	362	1559
CMR	2002	country	1_Afr	Cameroon	22740	65205	87945	0.033673	0.0965537	0.1302266	675319	1222	264	1486
CMR	2003	country	1_Afr	Cameroon	23021	64781	87802	0.0332726	0.0936283	0.1269008	691892	1242	329	1571
CMR	2004	country	1_Afr	Cameroon	23376	64540	87916	0.0329858	0.0910724	0.1240582	708668	1279	354	1633
CMR	2005	country	1_Afr	Cameroon	23762	64429	88191	0.0327583	0.0888216	0.1215799	725373	1309	395	1704
CMR	2006	country	1_Afr	Cameroon	24123	63906	88029	0.032516	0.08614	0.118656	741880	1371	474	1845
CMR	2007	country	1_Afr	Cameroon	24431	63273	87704	0.0322301	0.0834715	0.1157016	758018	1399	815	2214
CMR	2008	country	1_Afr	Cameroon	24680	62488	87168	0.0319017	0.0807729	0.1126747	773625	1412	1134	2547
CMR	2009	country	1_Afr	Cameroon	24887	61785	86672	0.0315595	0.0783507	0.1099102	788574	1450	782	2232
CMR	2010	country	1_Afr	Cameroon	25004	59999	85003	0.0311503	0.0747479	0.1058982	802689	1481	1008	2488
CMR	2011	country	1_Afr	Cameroon	25069	58465	83534	0.0307253	0.0716564	0.1023817	815907	1482	1617	3099
CMR	2012	country	1_Afr	Cameroon	25075	56602	81677	0.0302734	0.0683359	0.0986093	828284	1493	1421	2913
CMR	2013	country	1_Afr	Cameroon	25010	54139	79149	0.029776	0.0644561	0.0942322	839937	1510	1488	2999
CMR	2014	country	1_Afr	Cameroon	24863	52125	76988	0.029215	0.0612486	0.0904635	851037	1520	1418	2939
CMR	2015	country	1_Afr	Cameroon	24657	49984	74641	0.0286155	0.0580091	0.0866246	861665	1501	1375	2876
CMR	2016	country	1_Afr	Cameroon	24412	47314	71726	0.0279933	0.0542554	0.0822487	872066	1504	1303	2808
CMR	2017	country	1_Afr	Cameroon	24132	45584	69716	0.0273493	0.0516609	0.0790102	882364	1524	1271	2795
CMR	2018	country	1_Afr	Cameroon	23817	44168	67985	0.0266783	0.049474	0.0761523	892747	1488	1156	2644
CMR	2019	country	1_Afr	Cameroon	23557	43297	66854	0.0260778	0.0479302	0.074008	903337	1488	1152	2641
COD	2000	country	1_Afr	Democratic Republ	83892	241588	325480	0.0385087	0.1108955	0.1494042	2178521	4217	247	4464
COD	2001	country	1_Afr	Democratic Republ	85360	240793	326153	0.0381234	0.1075428	0.1456662	2239043	4351	296	4647
COD	2002	country	1_Afr	Democratic Republ	86784	274085	360869	0.0376939	0.1190464	0.1567403	2302337	4594	369	4963
COD	2003	country	1_Afr	Democratic Republ	88264	255556	343820	0.037262	0.1078867	0.1451487	2368742	4772	443	5216
COD	2004	country	1_Afr	Democratic Republ	89560	297235	386795	0.0367304	0.1219022	0.1586327	2438306	4973	358	5331
COD	2005	country	1_Afr	Democratic Republ	90595	245686	336281	0.036078	0.0978405	0.1339185	2511087	5199	603	5801
COD	2006	country	1_Afr	Democratic Republ	91447	223474	314921	0.0353507	0.0863885	0.1217392	2586853	5334	447	5780
COD	2007	country	1_Afr	Democratic Republ	92190	216523	308713	0.0345953	0.0812526	0.1158479	2664812	5487	834	6321
COD	2008	country	1_Afr	Democratic Republ	93029	219881	312910	0.0339036	0.0801335	0.1140371	2743928	5701	893	6594
COD	2009	country	1_Afr	Democratic Republ	93745	209571	303316	0.0332028	0.0742264	0.1074291	2823409	5832	906	6738
COD	2010	country	1_Afr	Democratic Republ	94325	210421	304746	0.0325007	0.0725029	0.1050036	2902244	6117	1192	7309
COD	2011	country	1_Afr	Democratic Republ	94820	219454	314274	0.0318233	0.0736528	0.1054761	2979578	6276	1513	7789
COD	2012	country	1_Afr	Democratic Republ	95258	209435	304693	0.0311813	0.0685553	0.0997366	3054973	6386	1849	8235
COD	2013	country	1_Afr	Democratic Republ	95695	207687	303382	0.0305913	0.0663925	0.0969837	3128178	6537	2480	9017
COD	2014	country	1_Afr	Democratic Republ	96346	196909	293255	0.0301171	0.0615524	0.0916696	3199042	6845	2880	9724
COD	2015	country	1_Afr	Democratic Republ	96813	201508	298321	0.0296281	0.0616682	0.0912963	3267607	6958	3137	10095
COD	2016	country	1_Afr	Democratic Republ	96793	219766	316559	0.02903	0.0659119	0.0949419	3334236	7098	2882	9979
COD	2017	country	1_Afr	Democratic Republ	96846	188149	284995	0.0284871	0.0553437	0.0838307	3399650	7236	2998	10234
COD	2018	country	1_Afr	Democratic Republ	96929	187825	284754	0.0279771	0.0542127	0.0821898	3464588	7271	3141	10412
COD	2019	country	1_Afr	Democratic Republ	96760	242791	339551	0.0274149	0.0687895	0.0962043	3529471	7366	3194	10560

iso3	year	level	whoreg6	whoname	nnd	pnd	u5d	nmr	pnmr	u5mr	Livebirths	Neonatal birth defects deaths	1-59 month birth defects deaths	Under five birth defects deaths
COG	2000	country	1_Afr	Congo	3698	9963	13661	0.0306563	0.0825934	0.1132497	120628	250	31	281
COG	2001	country	1_Afr	Congo	3745	10329	14074	0.0301245	0.0830861	0.1132105	124318	258	35	293
COG	2002	country	1_Afr	Congo	3765	10619	14384	0.0293817	0.082866	0.1122477	128141	271	42	314
COG	2003	country	1_Afr	Congo	3755	9355	13110	0.0284418	0.0708584	0.0993002	132024	271	65	336
COG	2004	country	1_Afr	Congo	3721	8748	12469	0.0273652	0.0643347	0.0916999	135976	277	102	379
COG	2005	country	1_Afr	Congo	3678	7481	11159	0.0262881	0.0534676	0.0797557	139911	281	84	365
COG	2006	country	1_Afr	Congo	3622	7053	10675	0.0251871	0.0490435	0.0742306	143804	282	108	390
COG	2007	country	1_Afr	Congo	3565	6648	10213	0.024155	0.0450476	0.0692026	147588	281	114	395
COG	2008	country	1_Afr	Congo	3531	6157	9688	0.0233461	0.0407113	0.0640574	151246	280	162	442
COG	2009	country	1_Afr	Congo	3515	5938	9453	0.0227342	0.038408	0.0611422	154613	284	152	435
COG	2010	country	1_Afr	Congo	3523	5773	9296	0.0223363	0.0366037	0.05894	157725	289	207	496
COG	2011	country	1_Afr	Congo	3534	5835	9369	0.0220317	0.0363775	0.0584092	160405	290	253	543
COG	2012	country	1_Afr	Congo	3545	5744	9289	0.0217808	0.035294	0.0570748	162758	300	243	543
COG	2013	country	1_Afr	Congo	3539	5659	9198	0.0214848	0.0343528	0.0558376	164721	303	261	565
COG	2014	country	1_Afr	Congo	3527	5482	9009	0.0211891	0.0329332	0.0541223	166453	301	262	563
COG	2015	country	1_Afr	Congo	3505	6458	9963	0.0208699	0.0384525	0.0593224	167945	305	261	566
COG	2016	country	1_Afr	Congo	3473	5589	9062	0.0205027	0.0329952	0.0534979	169392	303	178	481
COG	2017	country	1_Afr	Congo	3433	5595	9028	0.0200894	0.0327404	0.0528298	170886	302	176	477
COG	2018	country	1_Afr	Congo	3401	5097	8498	0.0197096	0.0295358	0.0492454	172555	295	195	489
COG	2019	country	1_Afr	Congo	3369	4873	8242	0.0193143	0.0279345	0.0472488	174430	302	191	494
COK	2000	country	6_Wpr	Cook Islands	4	4	8	0.0099909	0.0099909	0.0199819	400	1	1	2
COK	2001	country	6_Wpr	Cook Islands	4	4	8	0.0095093	0.0095093	0.0190187	421	1	1	2
COK	2002	country	6_Wpr	Cook Islands	4	3	7	0.0090672	0.0068004	0.0158675	441	1	1	2
COK	2003	country	6_Wpr	Cook Islands	3	3	6	0.0086356	0.0086356	0.0172712	347	1	1	1
COK	2004	country	6_Wpr	Cook Islands	3	3	6	0.0081971	0.0081971	0.0163943	366	1	1	1
COK	2005	country	6_Wpr	Cook Islands	3	2	5	0.0077656	0.0051771	0.0129427	386	1	1	1
COK	2006	country	6_Wpr	Cook Islands	3	2	5	0.0073456	0.0048971	0.0122427	408	1	1	1
COK	2007	country	6_Wpr	Cook Islands	2	3	5	0.0069327	0.010399	0.0173317	288	0	1	1
COK	2008	country	6_Wpr	Cook Islands	2	2	4	0.0065575	0.0065575	0.0131151	305	0	1	1
COK	2009	country	6_Wpr	Cook Islands	2	2	4	0.0061984	0.0061984	0.0123968	323	0	1	1
COK	2010	country	6_Wpr	Cook Islands	2	2	4	0.0058739	0.0058739	0.0117477	340	0	1	1
COK	2011	country	6_Wpr	Cook Islands	2	1	3	0.0056091	0.0028046	0.0084137	357	0	0	1
COK	2012	country	6_Wpr	Cook Islands	2	1	3	0.0053615	0.0026808	0.0080423	373	0	0	1
COK	2013	country	6_Wpr	Cook Islands	2	1	3	0.0051461	0.0025731	0.0077192	389	0	0	1
COK	2014	country	6_Wpr	Cook Islands	1	2	3	0.0049317	0.0098634	0.0147952	203	0	1	1
COK	2015	country	6_Wpr	Cook Islands	1	2	3	0.0047536	0.0095072	0.0142608	210	0	1	1
COK	2016	country	6_Wpr	Cook Islands	1	1	2	0.0045695	0.0045695	0.0091389	219	0	0	1
COK	2017	country	6_Wpr	Cook Islands	1	1	2	0.0044089	0.0044089	0.0088179	227	0	0	1
COK	2018	country	6_Wpr	Cook Islands	1	1	2	0.0042569	0.0042569	0.0085137	235	0	0	1
COK	2019	country	6_Wpr	Cook Islands	1	1	2	0.0041198	0.0041198	0.0082396	243	0	0	1

iso3	year	level	whoreg6	whoname	nnd	pnd	u5d	nmr	pnmr	u5mr	Livebirths	Neonatal birth defects deaths	1-59 month birth defects deaths	Under five birth defects deaths
COL	2000	country	2_Amr	Colombia	11900	10141	22041	0.0134832	0.0114901	0.0249733	882583	2020	1353	3373
COL	2001	country	2_Amr	Colombia	11422	9775	21197	0.0130672	0.011183	0.0242502	874096	2063	1403	3466
COL	2002	country	2_Amr	Colombia	10963	9393	20356	0.0126924	0.0108747	0.0235671	863746	2155	1472	3627
COL	2003	country	2_Amr	Colombia	10514	9006	19520	0.0123494	0.0105781	0.0229275	851380	2207	1415	3622
COL	2004	country	2_Amr	Colombia	10065	8620	18685	0.012022	0.0102961	0.0223181	837212	2200	1477	3677
COL	2005	country	2_Amr	Colombia	9614	8227	17841	0.0116954	0.0100081	0.0217035	822032	2021	1353	3374
COL	2006	country	2_Amr	Colombia	9164	7837	17001	0.0113619	0.0097166	0.0210786	806554	2140	1374	3514
COL	2007	country	2_Amr	Colombia	8728	7471	16199	0.0110232	0.0094356	0.0204588	791785	2042	1356	3398
COL	2008	country	2_Amr	Colombia	8320	7111	15431	0.0106866	0.0091337	0.0198203	778545	1964	1548	3512
COL	2009	country	2_Amr	Colombia	7940	6777	14717	0.0103484	0.0088326	0.019181	767271	1883	1345	3229
COL	2010	country	2_Amr	Colombia	7600	6468	14068	0.0100198	0.0085273	0.0185471	758501	2096	1405	3501
COL	2011	country	2_Amr	Colombia	7295	6188	13483	0.009699	0.0082272	0.0179262	752138	1940	1382	3322
COL	2012	country	2_Amr	Colombia	7017	5922	12939	0.0093816	0.0079176	0.0172991	747957	1952	1299	3251
COL	2013	country	2_Amr	Colombia	6769	5683	12452	0.0090847	0.0076272	0.0167118	745101	1857	1311	3168
COL	2014	country	2_Amr	Colombia	6533	5479	12012	0.008788	0.0073702	0.0161582	743398	1763	1262	3025
COL	2015	country	2_Amr	Colombia	6314	5276	11590	0.0085091	0.0071103	0.0156194	742026	1750	1271	3021
COL	2016	country	2_Amr	Colombia	6103	5107	11210	0.0082389	0.0068943	0.0151333	740753	1671	1194	2865
COL	2017	country	2_Amr	Colombia	5907	4932	10839	0.0079935	0.0066741	0.0146676	738978	1616	1154	2770
COL	2018	country	2_Amr	Colombia	5701	4747	10448	0.0077427	0.0064471	0.0141898	736304	1567	1111	2678
COL	2019	country	2_Amr	Colombia	5482	4609	10091	0.0074836	0.0062919	0.0137755	732532	1502	1079	2582
COM	2000	country	1_Afr	Comoros	826	1157	1983	0.0406334	0.0569162	0.0975496	20328	43	26	69
COM	2001	country	1_Afr	Comoros	840	1168	2008	0.0406314	0.056497	0.0971285	20674	44	26	70
COM	2002	country	1_Afr	Comoros	851	1186	2037	0.0404019	0.0563062	0.0967081	21063	47	27	74
COM	2003	country	1_Afr	Comoros	864	1199	2063	0.0402709	0.0558852	0.0961562	21455	49	25	74
COM	2004	country	1_Afr	Comoros	872	1206	2078	0.0399311	0.0552259	0.095157	21838	50	33	82
COM	2005	country	1_Afr	Comoros	880	1200	2080	0.0395488	0.0539301	0.0934789	22251	51	27	78
COM	2006	country	1_Afr	Comoros	881	1192	2073	0.0388927	0.0526222	0.0915149	22652	53	26	79
COM	2007	country	1_Afr	Comoros	884	1173	2057	0.0382997	0.0508208	0.0891206	23081	53	27	80
COM	2008	country	1_Afr	Comoros	887	1154	2041	0.0377512	0.0491148	0.086866	23496	55	37	92
COM	2009	country	1_Afr	Comoros	885	1134	2019	0.0370005	0.0474108	0.0844113	23919	54	40	95
COM	2010	country	1_Afr	Comoros	881	1116	1997	0.0362345	0.0458997	0.0821342	24314	56	34	90
COM	2011	country	1_Afr	Comoros	878	1083	1961	0.0355412	0.0438395	0.0793807	24704	56	42	99
COM	2012	country	1_Afr	Comoros	873	1061	1934	0.03484	0.0423428	0.0771828	25057	56	48	104
COM	2013	country	1_Afr	Comoros	864	1039	1903	0.0340436	0.040939	0.0749826	25379	57	49	106
COM	2014	country	1_Afr	Comoros	858	1008	1866	0.0334662	0.039317	0.0727832	25638	59	57	115
COM	2015	country	1_Afr	Comoros	846	980	1826	0.0326423	0.0378126	0.070455	25917	58	62	121
COM	2016	country	1_Afr	Comoros	833	951	1784	0.0318835	0.0364	0.0682834	26126	58	62	120
COM	2017	country	1_Afr	Comoros	824	920	1744	0.0312894	0.0349347	0.0662241	26335	57	62	119
COM	2018	country	1_Afr	Comoros	809	890	1699	0.030484	0.0335362	0.0640203	26538	58	59	117
COM	2019	country	1_Afr	Comoros	797	859	1656	0.0298207	0.0321405	0.0619612	26726	58	58	116

iso3	year	level	whoreg6	whoname	nnd	pnd	u5d	nmr	pnmr	u5mr	Livebirths	Neonatal birth defects deaths	1-59 month birth defects deaths	Under five birth defects deaths
CPV	2000	country	1_Afr	Cabo Verde	208	244	452	0.0175922	0.0206212	0.0382134	11823	18	23	41
CPV	2001	country	1_Afr	Cabo Verde	196	212	408	0.0168032	0.0182093	0.0350125	11664	19	19	38
CPV	2002	country	1_Afr	Cabo Verde	187	190	377	0.0161539	0.01645	0.0326039	11576	19	16	35
CPV	2003	country	1_Afr	Cabo Verde	180	176	356	0.0157355	0.015355	0.0310905	11439	20	15	35
CPV	2004	country	1_Afr	Cabo Verde	176	167	343	0.0154787	0.0146508	0.0301295	11371	20	14	34
CPV	2005	country	1_Afr	Cabo Verde	173	163	336	0.0153957	0.0144726	0.0298683	11237	21	14	35
CPV	2006	country	1_Afr	Cabo Verde	173	142	315	0.0154931	0.012676	0.0281692	11166	21	14	35
CPV	2007	country	1_Afr	Cabo Verde	174	139	313	0.0156289	0.0124749	0.0281038	11133	22	15	36
CPV	2008	country	1_Afr	Cabo Verde	174	136	310	0.0156884	0.0122523	0.0279407	11091	22	15	37
CPV	2009	country	1_Afr	Cabo Verde	173	131	304	0.0156076	0.0117869	0.0273945	11084	22	15	38
CPV	2010	country	1_Afr	Cabo Verde	169	122	291	0.0153251	0.0110969	0.026422	11028	23	16	38
CPV	2011	country	1_Afr	Cabo Verde	164	116	280	0.0148421	0.0104638	0.0253059	11050	23	16	39
CPV	2012	country	1_Afr	Cabo Verde	157	108	265	0.0141946	0.0097469	0.0239415	11061	24	16	40
CPV	2013	country	1_Afr	Cabo Verde	148	101	249	0.0134319	0.0091615	0.0225934	11019	24	16	40
CPV	2014	country	1_Afr	Cabo Verde	138	92	230	0.0126023	0.0084015	0.0210038	10950	24	16	40
CPV	2015	country	1_Afr	Cabo Verde	128	85	213	0.0117452	0.0077996	0.0195448	10898	24	16	40
CPV	2016	country	1_Afr	Cabo Verde	118	78	196	0.0109195	0.007218	0.0181375	10806	24	15	39
CPV	2017	country	1_Afr	Cabo Verde	109	72	181	0.0101613	0.006712	0.0168733	10727	24	15	38
CPV	2018	country	1_Afr	Cabo Verde	101	67	168	0.0095205	0.0063156	0.0158361	10609	22	15	37
CPV	2019	country	1_Afr	Cabo Verde	94	63	157	0.0089824	0.0060201	0.0150024	10465	20	14	35
CRI	2000	country	2_Amr	Costa Rica	587	424	1011	0.007687	0.0055525	0.0132395	76362	204	116	320
CRI	2001	country	2_Amr	Costa Rica	563	393	956	0.0075022	0.0052369	0.0127391	75045	183	120	303
CRI	2002	country	2_Amr	Costa Rica	541	366	907	0.0073194	0.0049518	0.0122712	73913	162	118	280
CRI	2003	country	2_Amr	Costa Rica	525	343	868	0.0072072	0.0047087	0.0119159	72844	156	123	279
CRI	2004	country	2_Amr	Costa Rica	514	324	838	0.0071252	0.0044914	0.0116166	72138	172	86	257
CRI	2005	country	2_Amr	Costa Rica	507	310	817	0.0070586	0.0043159	0.0113745	71828	185	97	282
CRI	2006	country	2_Amr	Costa Rica	500	303	803	0.0069793	0.0042295	0.0112088	71640	148	100	248
CRI	2007	country	2_Amr	Costa Rica	492	300	792	0.0068785	0.0041942	0.0110728	71527	167	109	276
CRI	2008	country	2_Amr	Costa Rica	487	295	782	0.0068007	0.0041195	0.0109202	71611	150	108	258
CRI	2009	country	2_Amr	Costa Rica	483	286	769	0.0067312	0.0039858	0.010717	71755	200	111	311
CRI	2010	country	2_Amr	Costa Rica	480	273	753	0.0066684	0.0037926	0.010461	71982	176	99	274
CRI	2011	country	2_Amr	Costa Rica	475	258	733	0.0065826	0.0035754	0.010158	72160	181	95	276
CRI	2012	country	2_Amr	Costa Rica	469	242	711	0.0065073	0.0033577	0.0098651	72072	190	96	286
CRI	2013	country	2_Amr	Costa Rica	461	227	688	0.0063944	0.0031487	0.0095431	72094	168	82	250
CRI	2014	country	2_Amr	Costa Rica	453	214	667	0.0062967	0.0029746	0.0092713	71942	187	75	262
CRI	2015	country	2_Amr	Costa Rica	445	203	648	0.0062202	0.0028375	0.0090578	71541	175	75	251
CRI	2016	country	2_Amr	Costa Rica	439	194	633	0.0061743	0.0027285	0.0089027	71102	172	72	243
CRI	2017	country	2_Amr	Costa Rica	434	187	621	0.0061606	0.0026545	0.008815	70448	173	69	242
CRI	2018	country	2_Amr	Costa Rica	430	180	610	0.0061562	0.002577	0.0087332	69849	170	67	236
CRI	2019	country	2_Amr	Costa Rica	425	174	599	0.0061514	0.0025185	0.0086699	69090	168	64	232

iso3	year	level	whoreg6	whoname	nnd	pnd	u5d	nmr	pnmr	u5mr	Livebirths	Neonatal birth defects deaths	1-59 month birth defects deaths	Under five birth defects deaths
CUB	2000	country	2_Amr	Cuba	624	671	1295	0.0042929	0.0046162	0.0089091	145358	161	195	356
CUB	2001	country	2_Amr	Cuba	594	630	1224	0.0041801	0.0044334	0.0086135	142102	155	185	340
CUB	2002	country	2_Amr	Cuba	557	598	1155	0.0040102	0.0043054	0.0083155	138897	148	193	341
CUB	2003	country	2_Amr	Cuba	515	573	1088	0.0037987	0.0042265	0.0080251	135574	136	147	283
CUB	2004	country	2_Amr	Cuba	474	553	1027	0.0035813	0.0041782	0.0077594	132355	121	169	290
CUB	2005	country	2_Amr	Cuba	440	532	972	0.0033974	0.0041078	0.0075051	129511	120	149	269
CUB	2006	country	2_Amr	Cuba	416	507	923	0.0032661	0.0039805	0.0072466	127370	103	122	225
CUB	2007	country	2_Amr	Cuba	400	480	880	0.0031705	0.0038047	0.0069752	126161	88	132	220
CUB	2008	country	2_Amr	Cuba	387	458	845	0.0030818	0.0036472	0.0067291	125574	77	107	183
CUB	2009	country	2_Amr	Cuba	374	441	815	0.002982	0.0035162	0.0064981	125420	87	103	190
CUB	2010	country	2_Amr	Cuba	361	431	792	0.0028699	0.0034263	0.0062962	125790	69	117	186
CUB	2011	country	2_Amr	Cuba	347	424	771	0.0027515	0.003362	0.0061135	126115	71	110	181
CUB	2012	country	2_Amr	Cuba	332	420	752	0.0026355	0.003334	0.0059695	125974	95	93	187
CUB	2013	country	2_Amr	Cuba	317	417	734	0.0025307	0.0033291	0.0058598	125260	59	103	162
CUB	2014	country	2_Amr	Cuba	302	411	713	0.0024331	0.0033113	0.0057444	124120	65	86	151
CUB	2015	country	2_Amr	Cuba	288	402	690	0.0023523	0.0032835	0.0056358	122432	55	95	150
CUB	2016	country	2_Amr	Cuba	275	391	666	0.0022913	0.0032578	0.005549	120021	48	100	148
CUB	2017	country	2_Amr	Cuba	265	376	641	0.0022569	0.0032022	0.0054591	117419	51	88	139
CUB	2018	country	2_Amr	Cuba	255	361	616	0.0022122	0.0031318	0.005344	115270	47	84	132
CUB	2019	country	2_Amr	Cuba	246	345	591	0.0021794	0.0030564	0.0052358	112877	45	81	126
CYP	2000	country	4_Eur	Cyprus	45	38	83	0.0036525	0.0030843	0.0067368	12320	8	24	32
CYP	2001	country	4_Eur	Cyprus	42	35	77	0.003441	0.0028675	0.0063084	12206	8	21	29
CYP	2002	country	4_Eur	Cyprus	39	33	72	0.0032478	0.0027482	0.005996	12008	7	19	26
CYP	2003	country	4_Eur	Cyprus	37	30	67	0.0030718	0.0024907	0.0055625	12045	7	17	23
CYP	2004	country	4_Eur	Cyprus	35	28	63	0.002893	0.0023144	0.0052074	12098	6	15	21
CYP	2005	country	4_Eur	Cyprus	33	26	59	0.0027194	0.0021425	0.0048619	12135	6	13	19
CYP	2006	country	4_Eur	Cyprus	31	25	56	0.0025475	0.0020544	0.0046019	12169	6	12	18
CYP	2007	country	4_Eur	Cyprus	30	23	53	0.0023904	0.0018326	0.004223	12550	5	10	15
CYP	2008	country	4_Eur	Cyprus	28	22	50	0.002247	0.0017655	0.0040124	12461	6	9	15
CYP	2009	country	4_Eur	Cyprus	27	20	47	0.0021239	0.0015733	0.0036972	12712	5	8	12
CYP	2010	country	4_Eur	Cyprus	26	19	45	0.0020113	0.0014698	0.0034811	12927	3	7	10
CYP	2011	country	4_Eur	Cyprus	24	19	43	0.0019106	0.0015126	0.0034232	12561	7	4	11
CYP	2012	country	4_Eur	Cyprus	23	18	41	0.001817	0.001422	0.003239	12658	3	7	9
CYP	2013	country	4_Eur	Cyprus	22	17	39	0.0017325	0.0013388	0.0030713	12698	0	5	5
CYP	2014	country	4_Eur	Cyprus	21	16	37	0.0016524	0.0012589	0.0029113	12709	6	2	8
CYP	2015	country	4_Eur	Cyprus	20	15	35	0.00158	0.001185	0.002765	12658	4	3	6
CYP	2016	country	4_Eur	Cyprus	19	14	33	0.0015065	0.0011101	0.0026166	12612	6	3	9
CYP	2017	country	4_Eur	Cyprus	18	14	32	0.0014415	0.0011212	0.0025627	12487	5	2	7
CYP	2018	country	4_Eur	Cyprus	17	13	30	0.0013767	0.0010527	0.0024294	12349	4	2	6
CYP	2019	country	4_Eur	Cyprus	16	13	29	0.0013209	0.0010732	0.002394	12113	4	2	6

iso3	year	level	whoreg6	whoname	nnd	pnd	u5d	nmr	pnmr	u5mr	Livebirths	Neonatal birth defects deaths	1-59 month birth defects deaths	Under five birth defects deaths
CZE	2000	country	4_Eur	Czechia	239	250	489	0.0027118	0.0028366	0.0055483	88135	50	47	98
CZE	2001	country	4_Eur	Czechia	228	236	464	0.0025495	0.0026389	0.0051884	89430	66	65	130
CZE	2002	country	4_Eur	Czechia	225	226	451	0.0024345	0.0024453	0.0048798	92422	58	47	105
CZE	2003	country	4_Eur	Czechia	225	219	444	0.0023544	0.0022916	0.004646	95567	60	57	118
CZE	2004	country	4_Eur	Czechia	227	212	439	0.002285	0.002134	0.004419	99344	37	41	78
CZE	2005	country	4_Eur	Czechia	227	207	434	0.002196	0.0020025	0.0041985	103370	53	55	108
CZE	2006	country	4_Eur	Czechia	223	204	427	0.002088	0.0019101	0.003998	106803	44	44	89
CZE	2007	country	4_Eur	Czechia	216	201	417	0.0019734	0.0018364	0.0038098	109454	64	34	97
CZE	2008	country	4_Eur	Czechia	207	198	405	0.0018574	0.0017766	0.003634	111448	39	61	101
CZE	2009	country	4_Eur	Czechia	198	194	392	0.0017647	0.0017291	0.0034938	112199	39	50	89
CZE	2010	country	4_Eur	Czechia	189	189	378	0.0016789	0.0016789	0.0033579	112571	34	49	83
CZE	2011	country	4_Eur	Czechia	182	184	366	0.0016211	0.0016389	0.0032601	112268	45	38	83
CZE	2012	country	4_Eur	Czechia	177	179	356	0.0015886	0.0016066	0.0031952	111418	49	38	87
CZE	2013	country	4_Eur	Czechia	175	175	350	0.0015765	0.0015765	0.003153	111006	37	37	74
CZE	2014	country	4_Eur	Czechia	175	174	349	0.001576	0.001567	0.003143	111041	35	59	94
CZE	2015	country	4_Eur	Czechia	176	174	350	0.0015805	0.0015626	0.0031431	111356	35	38	73
CZE	2016	country	4_Eur	Czechia	179	174	353	0.0016133	0.0015683	0.0031816	110950	33	28	61
CZE	2017	country	4_Eur	Czechia	181	173	354	0.0016305	0.0015585	0.003189	111006	37	34	71
CZE	2018	country	4_Eur	Czechia	181	173	354	0.0016371	0.0015647	0.0032017	110565	35	33	69
CZE	2019	country	4_Eur	Czechia	179	171	350	0.0016326	0.0015597	0.0031923	109638	35	33	68
DEU	2000	country	4_Eur	Germany	2074	1979	4053	0.0027696	0.0026428	0.0054124	748834	694	661	1355
DEU	2001	country	4_Eur	Germany	2022	1870	3892	0.0027456	0.0025392	0.0052848	736448	620	622	1243
DEU	2002	country	4_Eur	Germany	1966	1772	3738	0.0027164	0.0024483	0.0051647	723763	597	576	1173
DEU	2003	country	4_Eur	Germany	1905	1684	3589	0.0026764	0.0023659	0.0050423	711774	589	564	1153
DEU	2004	country	4_Eur	Germany	1849	1600	3449	0.0026383	0.002283	0.0049214	700822	540	512	1052
DEU	2005	country	4_Eur	Germany	1807	1507	3314	0.002612	0.0021783	0.0047903	691813	525	489	1015
DEU	2006	country	4_Eur	Germany	1768	1421	3189	0.0025821	0.0020753	0.0046574	684714	458	469	927
DEU	2007	country	4_Eur	Germany	1716	1358	3074	0.0025267	0.0019996	0.0045262	679151	477	419	895
DEU	2008	country	4_Eur	Germany	1648	1324	2972	0.0024393	0.0019597	0.004399	675605	463	416	879
DEU	2009	country	4_Eur	Germany	1588	1299	2887	0.0023537	0.0019254	0.0042791	674679	496	435	931
DEU	2010	country	4_Eur	Germany	1559	1262	2821	0.0023047	0.0018657	0.0041704	676434	473	443	917
DEU	2011	country	4_Eur	Germany	1560	1218	2778	0.002288	0.0017864	0.0040745	681803	464	409	873
DEU	2012	country	4_Eur	Germany	1578	1183	2761	0.0022828	0.0017114	0.0039942	691250	456	407	863
DEU	2013	country	4_Eur	Germany	1605	1163	2768	0.0022786	0.0016511	0.0039297	704386	511	397	908
DEU	2014	country	4_Eur	Germany	1643	1152	2795	0.0022829	0.0016006	0.0038835	719714	471	421	892
DEU	2015	country	4_Eur	Germany	1692	1145	2837	0.0022979	0.001555	0.0038529	736336	475	390	865
DEU	2016	country	4_Eur	Germany	1742	1143	2885	0.0023146	0.0015187	0.0038334	752599	500	428	929
DEU	2017	country	4_Eur	Germany	1779	1148	2927	0.0023195	0.0014968	0.0038164	766960	507	414	921
DEU	2018	country	4_Eur	Germany	1794	1160	2954	0.0023053	0.0014906	0.0037959	778212	510	418	928
DEU	2019	country	4_Eur	Germany	1788	1171	2959	0.0022751	0.00149	0.0037651	785897	510	422	932

iso3	year	level	whoreg6	whoname	nnd	pnd	u5d	nmr	pnmr	u5mr	Livebirths	Neonatal birth defects deaths	1-59 month birth defects deaths	Under five birth defects deaths
DJI	2000	country	5_Emr	Djibouti	986	1299	2285	0.0439442	0.0579081	0.1018523	22438	51	14	65
DJI	2001	country	5_Emr	Djibouti	972	1203	2175	0.0433258	0.0536138	0.0969396	22435	53	14	67
DJI	2002	country	5_Emr	Djibouti	958	1147	2105	0.0427138	0.0511557	0.0938696	22428	51	20	71
DJI	2003	country	5_Emr	Djibouti	942	1093	2035	0.0421304	0.0488857	0.0910161	22359	53	23	75
DJI	2004	country	5_Emr	Djibouti	923	1116	2039	0.0415007	0.0501601	0.0916607	22241	53	20	73
DJI	2005	country	5_Emr	Djibouti	902	1210	2112	0.0408487	0.0547903	0.095639	22081	52	23	75
DJI	2006	country	5_Emr	Djibouti	880	987	1867	0.040187	0.0450789	0.0852659	21898	52	25	77
DJI	2007	country	5_Emr	Djibouti	857	939	1796	0.0394812	0.0432364	0.0827176	21707	53	31	84
DJI	2008	country	5_Emr	Djibouti	835	979	1814	0.0387748	0.0454627	0.0842375	21535	52	31	83
DJI	2009	country	5_Emr	Djibouti	815	872	1687	0.0380776	0.0407301	0.0788077	21404	52	41	93
DJI	2010	country	5_Emr	Djibouti	796	818	1614	0.0373496	0.0383865	0.0757361	21312	52	42	94
DJI	2011	country	5_Emr	Djibouti	776	803	1579	0.0365335	0.0378194	0.074353	21241	52	41	94
DJI	2012	country	5_Emr	Djibouti	757	812	1569	0.035725	0.038323	0.074048	21190	52	40	92
DJI	2013	country	5_Emr	Djibouti	737	730	1467	0.0349054	0.0345888	0.0694941	21114	50	42	91
DJI	2014	country	5_Emr	Djibouti	719	691	1410	0.0341598	0.0328301	0.0669899	21048	50	33	83
DJI	2015	country	5_Emr	Djibouti	701	685	1386	0.0334541	0.0326811	0.0661352	20954	51	36	87
DJI	2016	country	5_Emr	Djibouti	682	623	1305	0.0327343	0.0299245	0.0626589	20834	49	36	85
DJI	2017	country	5_Emr	Djibouti	662	598	1260	0.0319996	0.0288904	0.06089	20688	49	41	90
DJI	2018	country	5_Emr	Djibouti	642	592	1234	0.0312148	0.0287763	0.059991	20567	49	44	93
DJI	2019	country	5_Emr	Djibouti	623	546	1169	0.0305082	0.0267495	0.0572577	20421	48	42	90
DMA	2000	country	2_Amr	Dominica	16	5	21	0.0128685	0.0040214	0.0168899	1243	2	1	3
DMA	2001	country	2_Amr	Dominica	15	6	21	0.0133075	0.005323	0.0186305	1127	4	2	6
DMA	2002	country	2_Amr	Dominica	15	5	20	0.013791	0.004597	0.0183879	1088	0	1	1
DMA	2003	country	2_Amr	Dominica	15	5	20	0.0143136	0.0047712	0.0190848	1048	0	1	1
DMA	2004	country	2_Amr	Dominica	15	5	20	0.0148908	0.0049636	0.0198544	1007	1	1	2
DMA	2005	country	2_Amr	Dominica	15	5	20	0.0155164	0.0051721	0.0206886	967	5	0	5
DMA	2006	country	2_Amr	Dominica	15	5	20	0.0161983	0.0053994	0.0215978	926	2	3	5
DMA	2007	country	2_Amr	Dominica	16	4	20	0.0169401	0.004235	0.0211752	945	2	1	3
DMA	2008	country	2_Amr	Dominica	16	5	21	0.0177267	0.0055396	0.0232663	903	2	0	2
DMA	2009	country	2_Amr	Dominica	17	4	21	0.0185881	0.0043737	0.0229618	915	2	1	3
DMA	2010	country	2_Amr	Dominica	17	5	22	0.019514	0.0057394	0.0252534	871	0	2	2
DMA	2011	country	2_Amr	Dominica	18	5	23	0.0205029	0.0056953	0.0261982	878	4	1	6
DMA	2012	country	2_Amr	Dominica	19	5	24	0.0215613	0.005674	0.0272353	881	2	1	3
DMA	2013	country	2_Amr	Dominica	20	5	25	0.0226329	0.0056582	0.0282912	884	3	1	3
DMA	2014	country	2_Amr	Dominica	22	5	27	0.0236542	0.005376	0.0290302	930	1	2	3
DMA	2015	country	2_Amr	Dominica	23	5	28	0.0246097	0.0053499	0.0299596	935	3	2	5
DMA	2016	country	2_Amr	Dominica	24	6	30	0.0255415	0.0063854	0.0319269	940	3	2	4
DMA	2017	country	2_Amr	Dominica	25	6	31	0.0264119	0.0063389	0.0327508	947	3	2	4
DMA	2018	country	2_Amr	Dominica	26	6	32	0.0272798	0.0062953	0.0335752	953	3	2	5
DMA	2019	country	2_Amr	Dominica	27	6	33	0.0280823	0.0062405	0.0343228	961	3	2	5

iso3	year	level	whoreg6	whoname	nnd	pnd	u5d	nmr	pnmr	u5mr	Livebirths	Neonatal birth defects deaths	1-59 month birth defects deaths	Under five birth defects deaths
DNK	2000	country	4_Eur	Denmark	230	146	376	0.0034979	0.0022204	0.0057182	65755	72	53	124
DNK	2001	country	4_Eur	Denmark	219	150	369	0.0033456	0.0022915	0.0056371	65459	64	45	109
DNK	2002	country	4_Eur	Denmark	211	149	360	0.0032396	0.0022877	0.0055273	65131	71	46	117
DNK	2003	country	4_Eur	Denmark	206	143	349	0.0031683	0.0021993	0.0053676	65019	63	58	121
DNK	2004	country	4_Eur	Denmark	202	134	336	0.0031175	0.002068	0.0051855	64796	50	51	101
DNK	2005	country	4_Eur	Denmark	199	123	322	0.0030671	0.0018957	0.0049628	64883	57	36	93
DNK	2006	country	4_Eur	Denmark	193	115	308	0.0029874	0.0017801	0.0047675	64604	53	44	97
DNK	2007	country	4_Eur	Denmark	186	107	293	0.0029012	0.001669	0.0045702	64111	48	36	83
DNK	2008	country	4_Eur	Denmark	179	99	278	0.0028335	0.0015671	0.0044006	63174	39	39	77
DNK	2009	country	4_Eur	Denmark	176	89	265	0.0028156	0.0014238	0.0042394	62509	27	24	50
DNK	2010	country	4_Eur	Denmark	175	80	255	0.0028478	0.0013018	0.0041496	61452	36	26	63
DNK	2011	country	4_Eur	Denmark	178	70	248	0.0029467	0.0011588	0.0041056	60406	34	23	57
DNK	2012	country	4_Eur	Denmark	183	61	244	0.0030773	0.0010258	0.0041031	59468	34	20	54
DNK	2013	country	4_Eur	Denmark	188	55	243	0.0031835	0.0009314	0.0041149	59054	37	18	55
DNK	2014	country	4_Eur	Denmark	191	51	242	0.0032453	0.0008666	0.0041119	58854	36	17	53
DNK	2015	country	4_Eur	Denmark	193	49	242	0.003256	0.0008267	0.0040827	59275	37	16	53
DNK	2016	country	4_Eur	Denmark	194	47	241	0.0032508	0.0007876	0.0040383	59678	37	16	53
DNK	2017	country	4_Eur	Denmark	193	46	239	0.0031899	0.0007603	0.0039502	60503	37	15	52
DNK	2018	country	4_Eur	Denmark	191	45	236	0.0031045	0.0007314	0.0038359	61524	36	15	51
DNK	2019	country	4_Eur	Denmark	187	45	232	0.0029978	0.0007214	0.0037192	62378	36	15	50
DOM	2000	country	2_Amr	Dominican Republi	4908	3610	8518	0.0233865	0.0172016	0.0405881	209864	513	311	824
DOM	2001	country	2_Amr	Dominican Republi	4870	3401	8271	0.023252	0.0162382	0.0394902	209445	517	320	837
DOM	2002	country	2_Amr	Dominican Republi	4828	3233	8061	0.0230996	0.0154683	0.0385678	209008	516	299	815
DOM	2003	country	2_Amr	Dominican Republi	4796	3079	7875	0.0229939	0.0147619	0.0377558	208578	529	273	803
DOM	2004	country	2_Amr	Dominican Republi	4774	2933	7707	0.022941	0.0140942	0.0370352	208100	532	255	786
DOM	2005	country	2_Amr	Dominican Republi	4758	2808	7566	0.0229123	0.013522	0.0364343	207661	523	237	760
DOM	2006	country	2_Amr	Dominican Republi	4752	2685	7437	0.0229181	0.0129493	0.0358674	207347	533	240	774
DOM	2007	country	2_Amr	Dominican Republi	4749	2575	7324	0.0229263	0.0124311	0.0353574	207142	528	239	767
DOM	2008	country	2_Amr	Dominican Republi	4740	2480	7220	0.0228888	0.0119756	0.0348644	207088	544	241	785
DOM	2009	country	2_Amr	Dominican Republi	4730	2401	7131	0.0228249	0.0115862	0.0344111	207230	535	247	783
DOM	2010	country	2_Amr	Dominican Republi	4721	2330	7051	0.0227534	0.0112297	0.033983	207486	543	254	797
DOM	2011	country	2_Amr	Dominican Republi	4704	2273	6977	0.0226273	0.0109336	0.0335609	207890	532	266	798
DOM	2012	country	2_Amr	Dominican Republi	4670	2230	6900	0.0224228	0.0107072	0.03313	208271	541	275	816
DOM	2013	country	2_Amr	Dominican Republi	4626	2167	6793	0.0221712	0.0103858	0.032557	208649	523	263	786
DOM	2014	country	2_Amr	Dominican Republi	4563	2104	6667	0.0218446	0.0100726	0.0319172	208884	526	294	820
DOM	2015	country	2_Amr	Dominican Republi	4473	2037	6510	0.0214093	0.0097498	0.031159	208928	519	298	816
DOM	2016	country	2_Amr	Dominican Republi	4372	1970	6342	0.0209519	0.0094408	0.0303927	208668	503	284	787
DOM	2017	country	2_Amr	Dominican Republi	4268	1892	6160	0.0205083	0.0090913	0.0295996	208111	508	293	801
DOM	2018	country	2_Amr	Dominican Republi	4138	1834	5972	0.0199735	0.0088524	0.0288259	207175	487	315	802
DOM	2019	country	2_Amr	Dominican Republi	3989	1779	5768	0.0193673	0.0086373	0.0280046	205966	479	310	789

iso3	year	level	whoreg6	whoname	nnd	pnd	u5d	nmr	pnmr	u5mr	Livebirths	Neonatal birth defects deaths	1-59 month birth defects deaths	Under five birth defects deaths
DZA	2000	country	1_Afr	Algeria	12772	12099	24871	0.021049	0.01994	0.040989	606775	1349	1286	2635
DZA	2001	country	1_Afr	Algeria	12654	11703	24357	0.0208824	0.0193127	0.0401952	605964	1361	1311	2672
DZA	2002	country	1_Afr	Algeria	12685	11296	23981	0.020635	0.0183748	0.0390099	614731	1365	1207	2571
DZA	2003	country	1_Afr	Algeria	12888	11876	24764	0.0203766	0.0187768	0.0391534	632492	1376	1234	2610
DZA	2004	country	1_Afr	Algeria	13246	8846	22092	0.0201191	0.0134359	0.0335551	658378	1425	1110	2535
DZA	2005	country	1_Afr	Algeria	13736	8350	22086	0.0198772	0.012083	0.0319602	691044	1520	1131	2651
DZA	2006	country	1_Afr	Algeria	14325	7911	22236	0.0196558	0.0108548	0.0305106	728792	1629	1256	2886
DZA	2007	country	1_Afr	Algeria	14896	7574	22470	0.0193669	0.0098476	0.0292144	769149	1784	1255	3038
DZA	2008	country	1_Afr	Algeria	15300	7441	22741	0.0189004	0.0091924	0.0280929	809506	1939	1244	3183
DZA	2009	country	1_Afr	Algeria	15414	7599	23013	0.018174	0.0089599	0.0271339	848137	2056	1353	3408
DZA	2010	country	1_Afr	Algeria	15477	7818	23295	0.0175113	0.0088457	0.026357	883829	2159	1443	3602
DZA	2011	country	1_Afr	Algeria	15703	7927	23630	0.0171369	0.0086506	0.0257875	916327	2287	1483	3770
DZA	2012	country	1_Afr	Algeria	15961	8051	24012	0.0168708	0.0085095	0.0253802	946075	2468	1525	3993
DZA	2013	country	1_Afr	Algeria	15952	8480	24432	0.0163972	0.0087171	0.0251143	972846	2531	1626	4157
DZA	2014	country	1_Afr	Algeria	15574	9206	24780	0.015639	0.0092445	0.0248835	995844	2571	1794	4365
DZA	2015	country	1_Afr	Algeria	15251	9779	25030	0.0150486	0.0096491	0.0246977	1013453	2559	1932	4491
DZA	2016	country	1_Afr	Algeria	15430	9620	25050	0.0150662	0.009393	0.0244591	1024149	2595	1921	4516
DZA	2017	country	1_Afr	Algeria	15965	8844	24809	0.01554	0.0086085	0.0241485	1027349	2671	1785	4456
DZA	2018	country	1_Afr	Algeria	16407	7901	24308	0.0160343	0.0077213	0.0237557	1023242	2736	1620	4356
DZA	2019	country	1_Afr	Algeria	16484	7139	23623	0.0162836	0.0070525	0.0233361	1012309	2728	1486	4213
ECU	2000	country	2_Amr	Ecuador	4675	4618	9293	0.0144736	0.0142972	0.0287708	323001	1164	1020	2184
ECU	2001	country	2_Amr	Ecuador	4395	4381	8776	0.0136436	0.0136001	0.0272437	322129	1094	965	2059
ECU	2002	country	2_Amr	Ecuador	4156	4161	8317	0.0129437	0.0129593	0.025903	321083	1034	919	1953
ECU	2003	country	2_Amr	Ecuador	3950	3963	7913	0.012344	0.0123846	0.0247286	319994	983	877	1860
ECU	2004	country	2_Amr	Ecuador	3765	3799	7564	0.0117991	0.0119056	0.0237047	319093	937	841	1778
ECU	2005	country	2_Amr	Ecuador	3607	3628	7235	0.011321	0.0113869	0.0227079	318612	897	804	1702
ECU	2006	country	2_Amr	Ecuador	3463	3470	6933	0.0108723	0.0108943	0.0217666	318515	862	770	1632
ECU	2007	country	2_Amr	Ecuador	3324	3322	6646	0.0104265	0.0104202	0.0208467	318803	828	737	1565
ECU	2008	country	2_Amr	Ecuador	3198	3171	6369	0.0100052	0.0099207	0.0199259	319634	795	704	1498
ECU	2009	country	2_Amr	Ecuador	3082	3022	6104	0.0096087	0.0094217	0.0190304	320750	768	671	1438
ECU	2010	country	2_Amr	Ecuador	2978	2876	5854	0.0092398	0.0089233	0.0181631	322302	743	638	1382
ECU	2011	country	2_Amr	Ecuador	2888	2735	5623	0.0089104	0.0084383	0.0173487	324117	713	607	1320
ECU	2012	country	2_Amr	Ecuador	2809	2612	5421	0.0086104	0.0080066	0.016617	326232	705	580	1285
ECU	2013	country	2_Amr	Ecuador	2739	2503	5242	0.0083414	0.0076227	0.0159641	328361	688	556	1243
ECU	2014	country	2_Amr	Ecuador	2679	2417	5096	0.0081079	0.007315	0.0154229	330419	639	537	1175
ECU	2015	country	2_Amr	Ecuador	2621	2363	4984	0.0078862	0.0071099	0.0149961	332353	690	548	1238
ECU	2016	country	2_Amr	Ecuador	2562	2336	4898	0.0076695	0.0069929	0.0146624	334051	645	532	1176
ECU	2017	country	2_Amr	Ecuador	2501	2334	4835	0.0074575	0.0069595	0.014417	335368	628	530	1158
ECU	2018	country	2_Amr	Ecuador	2438	2336	4774	0.0072471	0.0069439	0.014191	336411	623	531	1153
ECU	2019	country	2_Amr	Ecuador	2379	2317	4696	0.0070573	0.0068734	0.0139306	337099	601	526	1127

iso3	year	level	whoreg6	whoname	nnd	pnd	u5d	nmr	pnmr	u5mr	Livebirths	Neonatal birth defects deaths	1-59 month birth defects deaths	Under five birth defects deaths
EGY	2000	country	5_Emr	Egypt	39847	43586	83433	0.0223113	0.024405	0.0467163	1785959	3360	5376	8736
EGY	2001	country	5_Emr	Egypt	38589	40023	78612	0.0215232	0.0223231	0.0438463	1792904	3352	5034	8386
EGY	2002	country	5_Emr	Egypt	37491	36537	74028	0.0207917	0.0202625	0.0410542	1803168	3348	4818	8166
EGY	2003	country	5_Emr	Egypt	36552	33865	70417	0.0201017	0.018624	0.0387257	1818358	3350	4726	8076
EGY	2004	country	5_Emr	Egypt	35842	31846	67688	0.0194759	0.0173046	0.0367805	1840330	3545	4451	7996
EGY	2005	country	5_Emr	Egypt	35400	30167	65567	0.0188935	0.0161004	0.034994	1873657	3808	4378	8186
EGY	2006	country	5_Emr	Egypt	35227	29567	64794	0.0183137	0.0153714	0.0336851	1923533	4098	4274	8372
EGY	2007	country	5_Emr	Egypt	35290	28740	64030	0.0177234	0.0144338	0.0321572	1991151	4449	4235	8684
EGY	2008	country	5_Emr	Egypt	35564	27462	63026	0.0171407	0.0132358	0.0303765	2074825	4787	4114	8901
EGY	2009	country	5_Emr	Egypt	35945	26888	62833	0.0165586	0.0123865	0.028945	2170779	5220	4520	9740
EGY	2010	country	5_Emr	Egypt	36190	26422	62612	0.015929	0.0116297	0.0275587	2271956	5629	4909	10538
EGY	2011	country	5_Emr	Egypt	36054	26555	62609	0.0152156	0.0112069	0.0264225	2369547	5906	5357	11263
EGY	2012	country	5_Emr	Egypt	35686	26821	62507	0.0145326	0.0109224	0.025455	2455589	6299	5826	12126
EGY	2013	country	5_Emr	Egypt	35119	26919	62038	0.0139138	0.010665	0.0245788	2524040	6482	6373	12854
EGY	2014	country	5_Emr	Egypt	34357	27174	61531	0.0133585	0.0105655	0.023924	2571911	6718	6938	13656
EGY	2015	country	5_Emr	Egypt	33391	27707	61098	0.0128499	0.0106623	0.0235122	2598551	6753	7102	13855
EGY	2016	country	5_Emr	Egypt	32326	25973	58299	0.0124036	0.009966	0.0223696	2606187	6788	7187	13975
EGY	2017	country	5_Emr	Egypt	31104	25185	56289	0.0119562	0.0096811	0.0216373	2601496	6994	7188	14183
EGY	2018	country	5_Emr	Egypt	29877	24500	54377	0.0115328	0.0094574	0.0209902	2590605	6726	7174	13900
EGY	2019	country	5_Emr	Egypt	28710	23555	52265	0.0111415	0.0091412	0.0202827	2576850	6681	7066	13747
ERI	2000	country	1_Afr	Eritrea	2047	4414	6461	0.02653	0.0572032	0.0837333	77158	94	87	182
ERI	2001	country	1_Afr	Eritrea	2113	4327	6440	0.0260785	0.0534029	0.0794815	81025	99	108	207
ERI	2002	country	1_Afr	Eritrea	2204	3866	6070	0.0256644	0.045018	0.0706824	85878	107	111	218
ERI	2003	country	1_Afr	Eritrea	2301	3773	6074	0.0252299	0.0413655	0.0665954	91201	113	144	258
ERI	2004	country	1_Afr	Eritrea	2396	3493	5889	0.0247839	0.0361263	0.0609102	96676	121	169	290
ERI	2005	country	1_Afr	Eritrea	2476	3579	6055	0.0243112	0.035137	0.0594482	101846	127	176	302
ERI	2006	country	1_Afr	Eritrea	2534	3906	6440	0.023836	0.0367426	0.0605785	106310	132	187	319
ERI	2007	country	1_Afr	Eritrea	2561	3718	6279	0.0233272	0.0338625	0.0571897	109786	135	204	340
ERI	2008	country	1_Afr	Eritrea	2557	3650	6207	0.0227819	0.0325194	0.0553012	112238	137	218	355
ERI	2009	country	1_Afr	Eritrea	2521	3769	6290	0.0222	0.033193	0.055393	113558	140	232	372
ERI	2010	country	1_Afr	Eritrea	2465	3589	6054	0.0216602	0.0315405	0.0532007	113803	141	233	374
ERI	2011	country	1_Afr	Eritrea	2396	3526	5922	0.021178	0.031163	0.052341	113137	141	238	378
ERI	2012	country	1_Afr	Eritrea	2321	3799	6120	0.0207311	0.0339313	0.0546625	111957	141	235	376
ERI	2013	country	1_Afr	Eritrea	2241	3281	5522	0.0202631	0.0296624	0.0499255	110595	137	209	346
ERI	2014	country	1_Afr	Eritrea	2165	3345	5510	0.0198251	0.0306313	0.0504564	109205	136	185	320
ERI	2015	country	1_Afr	Eritrea	2095	3391	5486	0.0194048	0.0314082	0.0508131	107963	142	215	357
ERI	2016	country	1_Afr	Eritrea	2030	3074	5104	0.018988	0.0287555	0.0477435	106910	144	237	381
ERI	2017	country	1_Afr	Eritrea	1968	3529	5497	0.0185757	0.0333068	0.0518825	105945	149	227	376
ERI	2018	country	1_Afr	Eritrea	1912	2741	4653	0.0181846	0.0260711	0.0442557	105144	150	220	370
ERI	2019	country	1_Afr	Eritrea	1863	2218	4081	0.017823	0.0212233	0.0390463	104528	152	202	354

iso3	year	level	whoreg6	whoname	nnd	pnd	u5d	nmr	pnmr	u5mr	Livebirths	Neonatal birth defects deaths	1-59 month birth defects deaths	Under five birth defects deaths
ESP	2000	country	4_Eur	Spain	1123	996	2119	0.0028266	0.0025069	0.0053335	397302	366	385	751
ESP	2001	country	4_Eur	Spain	1139	972	2111	0.0027766	0.0023695	0.0051461	410217	336	362	699
ESP	2002	country	4_Eur	Spain	1148	976	2124	0.0026948	0.0022911	0.0049859	426001	347	345	692
ESP	2003	country	4_Eur	Spain	1155	993	2148	0.0026003	0.0022356	0.0048359	444175	373	318	691
ESP	2004	country	4_Eur	Spain	1163	1007	2170	0.002513	0.0021759	0.0046889	462797	300	368	668
ESP	2005	country	4_Eur	Spain	1166	1016	2182	0.0024301	0.0021174	0.0045475	479823	327	352	679
ESP	2006	country	4_Eur	Spain	1153	1020	2173	0.002338	0.0020684	0.0044064	493146	337	335	672
ESP	2007	country	4_Eur	Spain	1123	1018	2141	0.0022396	0.0020302	0.0042699	501421	289	304	593
ESP	2008	country	4_Eur	Spain	1089	995	2084	0.0021615	0.0019749	0.0041364	503815	296	337	633
ESP	2009	country	4_Eur	Spain	1064	941	2005	0.0021269	0.001881	0.004008	500253	257	296	553
ESP	2010	country	4_Eur	Spain	1042	866	1908	0.0021204	0.0017622	0.0038826	491420	310	285	595
ESP	2011	country	4_Eur	Spain	1009	793	1802	0.0021091	0.0016576	0.0037667	478405	291	294	585
ESP	2012	country	4_Eur	Spain	963	732	1695	0.0020791	0.0015804	0.0036594	463187	245	224	469
ESP	2013	country	4_Eur	Spain	912	682	1594	0.002035	0.0015218	0.0035568	448150	230	196	425
ESP	2014	country	4_Eur	Spain	861	645	1506	0.0019831	0.0014856	0.0034686	434179	220	179	399
ESP	2015	country	4_Eur	Spain	815	616	1431	0.0019327	0.0014608	0.0033935	421691	195	173	368
ESP	2016	country	4_Eur	Spain	778	591	1369	0.001892	0.0014372	0.0033292	411214	209	164	374
ESP	2017	country	4_Eur	Spain	749	568	1317	0.0018629	0.0014127	0.0032757	402054	191	158	349
ESP	2018	country	4_Eur	Spain	724	545	1269	0.0018356	0.0013818	0.0032174	394418	184	152	336
ESP	2019	country	4_Eur	Spain	698	523	1221	0.0018033	0.0013512	0.0031545	387068	181	146	327
EST	2000	country	4_Eur	Estonia	67	73	140	0.0053088	0.0057842	0.011093	12621	15	24	39
EST	2001	country	4_Eur	Estonia	62	67	129	0.0048508	0.005242	0.0100928	12781	16	20	36
EST	2002	country	4_Eur	Estonia	58	62	120	0.0044241	0.0047292	0.0091533	13110	19	19	38
EST	2003	country	4_Eur	Estonia	55	57	112	0.0040361	0.0041828	0.0082189	13627	15	18	34
EST	2004	country	4_Eur	Estonia	52	54	106	0.0036861	0.0038279	0.007514	14107	13	16	30
EST	2005	country	4_Eur	Estonia	49	51	100	0.003364	0.0035013	0.0068652	14566	17	11	28
EST	2006	country	4_Eur	Estonia	46	48	94	0.0030724	0.003206	0.0062784	14972	17	18	34
EST	2007	country	4_Eur	Estonia	42	46	88	0.0028034	0.0030704	0.0058738	14982	11	17	27
EST	2008	country	4_Eur	Estonia	39	43	82	0.0025547	0.0028168	0.0053715	15266	14	10	24
EST	2009	country	4_Eur	Estonia	36	39	75	0.0023929	0.0025923	0.0049853	15044	11	10	22
EST	2010	country	4_Eur	Estonia	32	37	69	0.0021161	0.0024467	0.0045628	15122	8	7	14
EST	2011	country	4_Eur	Estonia	29	34	63	0.0019307	0.0022636	0.0041943	15021	10	9	19
EST	2012	country	4_Eur	Estonia	26	32	58	0.0017743	0.0021838	0.0039581	14653	9	11	20
EST	2013	country	4_Eur	Estonia	24	29	53	0.0016463	0.0019893	0.0036357	14578	6	6	12
EST	2014	country	4_Eur	Estonia	22	26	48	0.0015421	0.0018225	0.0033646	14266	7	5	12
EST	2015	country	4_Eur	Estonia	20	24	44	0.0014517	0.0017421	0.0031938	13777	7	4	11
EST	2016	country	4_Eur	Estonia	18	23	41	0.0012969	0.0016572	0.0029541	13879	5	10	15
EST	2017	country	4_Eur	Estonia	17	21	38	0.0012237	0.0015116	0.0027353	13892	5	6	11
EST	2018	country	4_Eur	Estonia	15	20	35	0.0010832	0.0014443	0.0025274	13848	5	5	10
EST	2019	country	4_Eur	Estonia	14	19	33	0.0010252	0.0013913	0.0024165	13656	4	5	9

iso3	year	level	whoreg6	whoname	nnd	pnd	u5d	nmr	pnmr	u5mr	Livebirths	Neonatal birth defects deaths	1-59 month birth defects deaths	Under five birth defects deaths
ETH	2000	country	1_Afr	Ethiopia	139801	255677	395478	0.0482399	0.0882243	0.1364643	2898036	3782	984	4766
ETH	2001	country	1_Afr	Ethiopia	138515	236342	374857	0.0473348	0.0807652	0.1280999	2926284	3853	996	4849
ETH	2002	country	1_Afr	Ethiopia	136822	239676	376498	0.0464044	0.0812882	0.1276926	2948470	3992	1111	5103
ETH	2003	country	1_Afr	Ethiopia	134964	221058	356022	0.0454851	0.0745002	0.1199853	2967212	3933	1145	5078
ETH	2004	country	1_Afr	Ethiopia	133056	203124	336180	0.0445822	0.0680594	0.1126416	2984508	3958	1193	5152
ETH	2005	country	1_Afr	Ethiopia	130931	187059	317990	0.0436055	0.0622984	0.1059039	3002625	4140	1497	5637
ETH	2006	country	1_Afr	Ethiopia	128661	179841	308502	0.0425513	0.0594777	0.102029	3023668	4150	1143	5293
ETH	2007	country	1_Afr	Ethiopia	126353	156071	282424	0.0414457	0.0511938	0.0926395	3048638	4198	1391	5589
ETH	2008	country	1_Afr	Ethiopia	124074	156703	280777	0.0403075	0.0509074	0.0912149	3078188	4323	2580	6903
ETH	2009	country	1_Afr	Ethiopia	122027	154326	276353	0.0392013	0.0495775	0.0887788	3112829	4292	1895	6187
ETH	2010	country	1_Afr	Ethiopia	119835	134014	253849	0.03801	0.0425072	0.0805172	3152726	4414	2165	6579
ETH	2011	country	1_Afr	Ethiopia	117547	119922	237469	0.0367633	0.0375062	0.0742695	3197398	4492	2519	7011
ETH	2012	country	1_Afr	Ethiopia	115053	113814	228867	0.0354528	0.0350712	0.070524	3245244	4407	2210	6617
ETH	2013	country	1_Afr	Ethiopia	112613	109860	222473	0.0341813	0.0333455	0.0675267	3294584	4444	1821	6266
ETH	2014	country	1_Afr	Ethiopia	110169	119351	229520	0.0329415	0.035687	0.0686285	3344380	4551	2881	7432
ETH	2015	country	1_Afr	Ethiopia	107799	117377	225176	0.0317646	0.0345869	0.0663515	3393685	4682	2606	7288
ETH	2016	country	1_Afr	Ethiopia	105482	97015	202497	0.0306461	0.0281861	0.0588323	3441935	4749	2814	7563
ETH	2017	country	1_Afr	Ethiopia	103170	83728	186898	0.0295712	0.0239987	0.0535699	3488867	4819	4195	9014
ETH	2018	country	1_Afr	Ethiopia	100960	78410	179370	0.028568	0.022187	0.0507551	3534024	4807	2561	7368
ETH	2019	country	1_Afr	Ethiopia	98795	78539	177334	0.027621	0.0219578	0.0495789	3576804	4800	2559	7359
FIN	2000	country	4_Eur	Finland	142	107	249	0.0024703	0.0018614	0.0043317	57483	55	44	99
FIN	2001	country	4_Eur	Finland	136	104	240	0.0023801	0.0018201	0.0042002	57140	52	43	94
FIN	2002	country	4_Eur	Finland	132	101	233	0.0023024	0.0017617	0.0040642	57330	52	26	79
FIN	2003	country	4_Eur	Finland	128	100	228	0.0022341	0.0017454	0.0039795	57293	47	33	80
FIN	2004	country	4_Eur	Finland	125	98	223	0.0021555	0.0016899	0.0038454	57992	47	28	75
FIN	2005	country	4_Eur	Finland	123	94	217	0.0021115	0.0016137	0.0037252	58252	43	28	72
FIN	2006	country	4_Eur	Finland	120	91	211	0.0020447	0.0015505	0.0035952	58690	44	35	80
FIN	2007	country	4_Eur	Finland	116	88	204	0.0019526	0.0014813	0.0034339	59407	41	37	78
FIN	2008	country	4_Eur	Finland	112	84	196	0.0018815	0.0014111	0.0032926	59527	39	22	60
FIN	2009	country	4_Eur	Finland	106	82	188	0.0017643	0.0013648	0.0031292	60080	37	30	67
FIN	2010	country	4_Eur	Finland	100	80	180	0.0016723	0.0013378	0.0030101	59798	36	22	58
FIN	2011	country	4_Eur	Finland	93	78	171	0.0015633	0.0013112	0.0028745	59490	36	22	57
FIN	2012	country	4_Eur	Finland	88	75	163	0.0014955	0.0012746	0.00277	58844	33	13	46
FIN	2013	country	4_Eur	Finland	83	71	154	0.0014358	0.0012283	0.0026641	57806	32	25	57
FIN	2014	country	4_Eur	Finland	80	67	147	0.0014059	0.0011774	0.0025833	56905	29	22	51
FIN	2015	country	4_Eur	Finland	77	63	140	0.0014023	0.0011473	0.0025496	54911	29	20	49
FIN	2016	country	4_Eur	Finland	75	60	135	0.0014002	0.0011202	0.0025204	53563	27	20	47
FIN	2017	country	4_Eur	Finland	73	57	130	0.0013994	0.0010927	0.0024921	52164	26	18	45
FIN	2018	country	4_Eur	Finland	71	55	126	0.0013817	0.0010703	0.002452	51387	26	18	43
FIN	2019	country	4_Eur	Finland	70	52	122	0.0014045	0.0010434	0.0024479	49839	25	17	42

iso3	year	level	whoreg6	whoname	nnd	pnd	u5d	nmr	pnmr	u5mr	Livebirths	Neonatal birth defects deaths	1-59 month birth defects deaths	Under five birth defects deaths
FJI	2000	country	6_Wpr	Fiji	182	276	458	0.0091018	0.0138228	0.0229246	19996	36	57	93
FJI	2001	country	6_Wpr	Fiji	182	263	445	0.0091869	0.0132823	0.0224692	19811	36	56	92
FJI	2002	country	6_Wpr	Fiji	186	273	459	0.0094827	0.013903	0.0233857	19615	37	54	91
FJI	2003	country	6_Wpr	Fiji	193	261	454	0.0099326	0.0134577	0.0233903	19431	38	53	91
FJI	2004	country	6_Wpr	Fiji	201	241	442	0.0104269	0.0124978	0.0229247	19277	39	51	90
FJI	2005	country	6_Wpr	Fiji	207	238	445	0.0108394	0.0124532	0.0232926	19097	40	50	90
FJI	2006	country	6_Wpr	Fiji	209	242	451	0.01096	0.0126745	0.0236345	19069	40	50	90
FJI	2007	country	6_Wpr	Fiji	204	244	448	0.0107351	0.0128191	0.0235541	19003	39	51	90
FJI	2008	country	6_Wpr	Fiji	196	252	448	0.0103229	0.0132941	0.023617	18987	38	53	91
FJI	2009	country	6_Wpr	Fiji	188	261	449	0.0099181	0.0137693	0.0236874	18955	36	55	91
FJI	2010	country	6_Wpr	Fiji	183	265	448	0.009611	0.0139175	0.0235284	19041	36	56	92
FJI	2011	country	6_Wpr	Fiji	180	268	448	0.009429	0.0140387	0.0234676	19090	35	57	92
FJI	2012	country	6_Wpr	Fiji	180	270	450	0.0093903	0.0140855	0.0234758	19169	36	57	93
FJI	2013	country	6_Wpr	Fiji	182	271	453	0.0094735	0.0141061	0.0235795	19212	36	57	93
FJI	2014	country	6_Wpr	Fiji	186	272	458	0.0096687	0.0141392	0.0238079	19237	37	57	94
FJI	2015	country	6_Wpr	Fiji	191	274	465	0.0099321	0.0142481	0.0241802	19231	38	57	95
FJI	2016	country	6_Wpr	Fiji	196	277	473	0.0102334	0.0144625	0.0246959	19153	38	57	95
FJI	2017	country	6_Wpr	Fiji	200	279	479	0.0105231	0.0146798	0.0252029	19006	39	56	95
FJI	2018	country	6_Wpr	Fiji	202	281	483	0.0107366	0.0149356	0.0256722	18814	39	56	95
FJI	2019	country	6_Wpr	Fiji	202	281	483	0.0108447	0.0150712	0.025916	18627	39	55	94
FRA	2000	country	4_Eur	France	2076	1953	4029	0.0027485	0.0025857	0.0053342	755320	724	404	1128
FRA	2001	country	4_Eur	France	2082	1879	3961	0.0027327	0.0024663	0.005199	761873	636	387	1023
FRA	2002	country	4_Eur	France	2074	1798	3872	0.0026988	0.0023396	0.0050384	768499	564	377	942
FRA	2003	country	4_Eur	France	2030	1735	3765	0.0026194	0.0022387	0.0048581	774999	628	341	969
FRA	2004	country	4_Eur	France	1969	1691	3660	0.0025202	0.0021644	0.0046846	781277	583	378	960
FRA	2005	country	4_Eur	France	1908	1667	3575	0.0024256	0.0021193	0.0045449	786598	552	348	900
FRA	2006	country	4_Eur	France	1866	1648	3514	0.0023593	0.0020837	0.004443	790901	523	401	924
FRA	2007	country	4_Eur	France	1850	1623	3473	0.0023282	0.0020426	0.0043708	794593	535	377	912
FRA	2008	country	4_Eur	France	1860	1587	3447	0.0023348	0.0019922	0.004327	796626	538	342	880
FRA	2009	country	4_Eur	France	1858	1569	3427	0.0023291	0.0019668	0.0042959	797732	546	354	900
FRA	2010	country	4_Eur	France	1814	1588	3402	0.0022768	0.0019931	0.0042699	796734	511	454	966
FRA	2011	country	4_Eur	France	1754	1614	3368	0.0022115	0.002035	0.0042465	793120	454	424	878
FRA	2012	country	4_Eur	France	1732	1585	3317	0.0022004	0.0020136	0.004214	787130	459	375	834
FRA	2013	country	4_Eur	France	1767	1486	3253	0.002268	0.0019073	0.0041753	779109	442	390	833
FRA	2014	country	4_Eur	France	1812	1382	3194	0.0023563	0.0017971	0.0041534	769014	439	365	804
FRA	2015	country	4_Eur	France	1826	1333	3159	0.0024088	0.0017584	0.0041672	758058	402	394	796
FRA	2016	country	4_Eur	France	1822	1330	3152	0.0024387	0.0017802	0.0042189	747121	433	364	797
FRA	2017	country	4_Eur	France	1833	1340	3173	0.0024852	0.0018168	0.004302	737570	427	367	795
FRA	2018	country	4_Eur	France	1874	1340	3214	0.00257	0.0018377	0.0044077	729178	431	367	799
FRA	2019	country	4_Eur	France	1920	1331	3251	0.0026537	0.0018396	0.0044934	723511	449	365	813

iso3	year	level	whoreg6	whoname	nnd	pnd	u5d	nmr	pnmr	u5mr	Livebirths	Neonatal birth defects deaths	1-59 month birth defects deaths	Under five birth defects deaths
FSM	2000	country	6_Wpr	Micronesia (Federa	77	95	172	0.0239761	0.0295809	0.0535569	3212	8	9	16
FSM	2001	country	6_Wpr	Micronesia (Federa	74	90	164	0.0235603	0.0286544	0.0522148	3141	7	8	15
FSM	2002	country	6_Wpr	Micronesia (Federa	71	86	157	0.0231136	0.0279968	0.0511104	3072	7	9	16
FSM	2003	country	6_Wpr	Micronesia (Federa	67	82	149	0.0227007	0.0277829	0.0504836	2951	7	9	16
FSM	2004	country	6_Wpr	Micronesia (Federa	64	76	140	0.0222769	0.0264538	0.0487307	2873	7	8	14
FSM	2005	country	6_Wpr	Micronesia (Federa	61	71	132	0.0218629	0.0254469	0.0473098	2790	6	9	15
FSM	2006	country	6_Wpr	Micronesia (Federa	58	66	124	0.0214276	0.0243831	0.0458108	2707	6	7	13
FSM	2007	country	6_Wpr	Micronesia (Federa	55	62	117	0.0208893	0.0235479	0.0444372	2633	6	8	14
FSM	2008	country	6_Wpr	Micronesia (Federa	53	57	110	0.0203971	0.0219366	0.0423337	2598	6	6	12
FSM	2009	country	6_Wpr	Micronesia (Federa	51	54	105	0.0199126	0.0210839	0.0409965	2561	6	6	12
FSM	2010	country	6_Wpr	Micronesia (Federa	49	51	100	0.0194829	0.0202781	0.039761	2515	6	6	11
FSM	2011	country	6_Wpr	Micronesia (Federa	48	48	96	0.0190274	0.0190274	0.0380547	2523	6	7	12
FSM	2012	country	6_Wpr	Micronesia (Federa	46	47	93	0.0185377	0.0189407	0.0374784	2481	5	7	12
FSM	2013	country	6_Wpr	Micronesia (Federa	45	45	90	0.0180973	0.0180973	0.0361946	2487	5	6	12
FSM	2014	country	6_Wpr	Micronesia (Federa	44	43	87	0.0176474	0.0172463	0.0348937	2493	5	5	11
FSM	2015	country	6_Wpr	Micronesia (Federa	44	40	84	0.0172988	0.0157261	0.0330249	2544	6	4	10
FSM	2016	country	6_Wpr	Micronesia (Federa	43	39	82	0.0169537	0.0153766	0.0323304	2536	6	4	10
FSM	2017	country	6_Wpr	Micronesia (Federa	42	38	80	0.0165286	0.0149544	0.031483	2541	6	5	10
FSM	2018	country	6_Wpr	Micronesia (Federa	42	36	78	0.0161966	0.0138828	0.0300795	2593	6	4	10
FSM	2019	country	6_Wpr	Micronesia (Federa	41	34	75	0.0158625	0.0131543	0.0290168	2585	6	4	10
GAB	2000	country	1_Afr	Gabon	1166	2299	3465	0.0284673	0.0561207	0.084588	40959	78	20	99
GAB	2001	country	1_Afr	Gabon	1173	3368	4541	0.0281809	0.080925	0.1091059	41624	82	22	104
GAB	2002	country	1_Afr	Gabon	1182	2985	4167	0.0278649	0.0703705	0.0982353	42419	82	25	107
GAB	2003	country	1_Afr	Gabon	1191	2816	4007	0.027491	0.0649982	0.0924892	43323	85	30	114
GAB	2004	country	1_Afr	Gabon	1207	2647	3854	0.0271824	0.0596151	0.0867975	44404	87	34	122
GAB	2005	country	1_Afr	Gabon	1225	1819	3044	0.0268182	0.0398185	0.0666368	45678	90	38	129
GAB	2006	country	1_Afr	Gabon	1246	1878	3124	0.026434	0.0398377	0.0662717	47136	92	41	133
GAB	2007	country	1_Afr	Gabon	1271	1910	3181	0.02604	0.0391347	0.0651748	48809	97	50	147
GAB	2008	country	1_Afr	Gabon	1298	1755	3053	0.0256586	0.0346925	0.0603511	50587	99	57	156
GAB	2009	country	1_Afr	Gabon	1322	1765	3087	0.0251956	0.0336446	0.0588401	52470	103	53	156
GAB	2010	country	1_Afr	Gabon	1346	1784	3130	0.0247487	0.0328047	0.0575534	54387	106	51	157
GAB	2011	country	1_Afr	Gabon	1367	1813	3180	0.0242313	0.0321431	0.0563744	56415	111	64	175
GAB	2012	country	1_Afr	Gabon	1386	1832	3218	0.023728	0.0313581	0.0550861	58412	110	60	170
GAB	2013	country	1_Afr	Gabon	1402	1672	3074	0.0232404	0.0277127	0.0509531	60326	115	56	171
GAB	2014	country	1_Afr	Gabon	1412	1633	3045	0.0227196	0.0262682	0.0489877	62149	118	44	162
GAB	2015	country	1_Afr	Gabon	1416	1631	3047	0.0222157	0.0255825	0.0477982	63739	117	53	170
GAB	2016	country	1_Afr	Gabon	1412	1806	3218	0.0217079	0.0277714	0.0494794	65045	118	50	168
GAB	2017	country	1_Afr	Gabon	1398	1685	3083	0.0211632	0.0255004	0.0466635	66058	118	51	169
GAB	2018	country	1_Afr	Gabon	1382	1457	2839	0.0207081	0.0218287	0.0425368	66737	119	47	166
GAB	2019	country	1_Afr	Gabon	1359	1504	2863	0.0202478	0.0224039	0.0426517	67118	119	46	165

iso3	year	level	whoreg6	whoname	nnd	pnd	u5d	nmr	pnmr	u5mr	Livebirths	Neonatal birth defects deaths	1-59 month birth defects deaths	Under five birth defects deaths
GBR	2000	country	4_Eur	United Kingdom of (	2636	1976	4612	0.0037852	0.0028375	0.0066227	696389	586	505	1091
GBR	2001	country	4_Eur	United Kingdom of (	2554	1933	4487	0.0036989	0.0027995	0.0064984	690478	677	460	1137
GBR	2002	country	4_Eur	United Kingdom of (	2497	1895	4392	0.0036178	0.0027456	0.0063633	690208	639	460	1099
GBR	2003	country	4_Eur	United Kingdom of (	2482	1850	4332	0.0035682	0.0026596	0.0062278	695594	626	453	1078
GBR	2004	country	4_Eur	United Kingdom of (	2494	1809	4303	0.0035278	0.0025589	0.0060867	706951	594	428	1022
GBR	2005	country	4_Eur	United Kingdom of (	2512	1784	4296	0.0034761	0.0024687	0.0059448	722644	653	473	1126
GBR	2006	country	4_Eur	United Kingdom of (	2525	1771	4296	0.0034066	0.0023893	0.0057959	741217	658	447	1106
GBR	2007	country	4_Eur	United Kingdom of (	2527	1759	4286	0.0033252	0.0023146	0.0056399	759944	673	419	1092
GBR	2008	country	4_Eur	United Kingdom of (	2509	1738	4247	0.0032293	0.002237	0.0054662	776950	687	400	1087
GBR	2009	country	4_Eur	United Kingdom of (	2476	1702	4178	0.0031326	0.0021533	0.0052859	790404	709	429	1138
GBR	2010	country	4_Eur	United Kingdom of (	2430	1650	4080	0.0030397	0.002064	0.0051036	799433	702	407	1109
GBR	2011	country	4_Eur	United Kingdom of (	2373	1588	3961	0.0029533	0.0019763	0.0049296	803519	665	392	1057
GBR	2012	country	4_Eur	United Kingdom of (	2305	1534	3839	0.0028672	0.0019082	0.0047754	803914	703	387	1090
GBR	2013	country	4_Eur	United Kingdom of (	2235	1491	3726	0.0027882	0.00186	0.0046482	801604	629	381	1010
GBR	2014	country	4_Eur	United Kingdom of (	2182	1446	3628	0.002738	0.0018144	0.0045524	796941	662	360	1022
GBR	2015	country	4_Eur	United Kingdom of (	2157	1391	3548	0.0027265	0.0017583	0.0044848	791116	658	305	963
GBR	2016	country	4_Eur	United Kingdom of (	2154	1327	3481	0.0027446	0.0016908	0.0044354	784825	628	350	978
GBR	2017	country	4_Eur	United Kingdom of (	2158	1265	3423	0.0027687	0.001623	0.0043916	779434	648	308	956
GBR	2018	country	4_Eur	United Kingdom of (	2156	1214	3370	0.0027807	0.0015658	0.0043465	775343	644	296	940
GBR	2019	country	4_Eur	United Kingdom of (	2142	1171	3313	0.0027726	0.0015158	0.0042884	772548	636	286	921
GEO	2000	country	4_Eur	Georgia	1204	831	2035	0.0224266	0.0154788	0.0379054	53686	133	95	229
GEO	2001	country	4_Eur	Georgia	1118	702	1820	0.0215325	0.0135204	0.035053	51921	126	86	212
GEO	2002	country	4_Eur	Georgia	1043	588	1631	0.0204886	0.0115506	0.0320392	50906	120	83	203
GEO	2003	country	4_Eur	Georgia	975	491	1466	0.0192663	0.0097023	0.0289687	50606	117	80	197
GEO	2004	country	4_Eur	Georgia	899	427	1326	0.0176833	0.0083991	0.0260824	50839	118	81	199
GEO	2005	country	4_Eur	Georgia	830	375	1205	0.0160987	0.0072735	0.0233722	51557	119	77	196
GEO	2006	country	4_Eur	Georgia	769	331	1100	0.0146294	0.0062969	0.0209263	52566	122	73	195
GEO	2007	country	4_Eur	Georgia	714	295	1009	0.0132759	0.0054851	0.018761	53782	127	68	195
GEO	2008	country	4_Eur	Georgia	663	265	928	0.0120657	0.0048226	0.0168883	54949	126	65	191
GEO	2009	country	4_Eur	Georgia	616	241	857	0.0109959	0.004302	0.0152979	56021	125	62	187
GEO	2010	country	4_Eur	Georgia	574	220	794	0.0101022	0.0038719	0.0139741	56820	123	60	183
GEO	2011	country	4_Eur	Georgia	534	205	739	0.009325	0.0035798	0.0129048	57265	119	58	177
GEO	2012	country	4_Eur	Georgia	496	197	693	0.0086164	0.0034223	0.0120387	57564	113	57	169
GEO	2013	country	4_Eur	Georgia	457	198	655	0.0079459	0.0034426	0.0113885	57514	106	59	165
GEO	2014	country	4_Eur	Georgia	418	207	625	0.0073037	0.0036169	0.0109206	57231	99	62	161
GEO	2015	country	4_Eur	Georgia	380	219	599	0.0066887	0.0038548	0.0105434	56813	90	67	156
GEO	2016	country	4_Eur	Georgia	342	235	577	0.0061146	0.0042016	0.0103162	55931	83	72	155
GEO	2017	country	4_Eur	Georgia	308	248	556	0.0056025	0.0045111	0.0101137	54975	74	77	151
GEO	2018	country	4_Eur	Georgia	280	255	535	0.0051927	0.0047291	0.0099218	53922	68	79	148
GEO	2019	country	4_Eur	Georgia	258	255	513	0.0048825	0.0048258	0.0097083	52841	63	80	143

iso3	year	level	whoreg6	whoname	nnd	pnd	u5d	nmr	pnmr	u5mr	Livebirths	Neonatal birth defects deaths	1-59 month birth defects deaths	Under five birth defects deaths
GHA	2000	country	1_Afr	Ghana	24858	46415	71273	0.0361186	0.0674406	0.1035592	688232	1217	731	1949
GHA	2001	country	1_Afr	Ghana	24542	42833	67375	0.0352351	0.0614964	0.0967315	696521	1253	631	1884
GHA	2002	country	1_Afr	Ghana	24273	41027	65300	0.0344225	0.0581815	0.0926041	705148	1251	763	2014
GHA	2003	country	1_Afr	Ghana	24104	35963	60067	0.0337383	0.0503375	0.0840758	714441	1284	918	2201
GHA	2004	country	1_Afr	Ghana	24039	35366	59405	0.0331786	0.0488126	0.0819911	724534	1319	1092	2410
GHA	2005	country	1_Afr	Ghana	24052	34880	58932	0.0326966	0.0474169	0.0801135	735612	1375	1162	2536
GHA	2006	country	1_Afr	Ghana	24118	34298	58416	0.0322517	0.0458644	0.0781161	747806	1376	843	2219
GHA	2007	country	1_Afr	Ghana	24166	33206	57372	0.0317591	0.0436393	0.0753983	760916	1411	1140	2550
GHA	2008	country	1_Afr	Ghana	24151	32417	56568	0.0311787	0.0418496	0.0730282	774601	1435	1337	2773
GHA	2009	country	1_Afr	Ghana	24046	31368	55414	0.030492	0.0397763	0.0702683	788600	1469	1001	2470
GHA	2010	country	1_Afr	Ghana	23883	30213	54096	0.0297663	0.0376552	0.0674215	802350	1473	1387	2860
GHA	2011	country	1_Afr	Ghana	23634	28627	52261	0.0289838	0.0351075	0.0640912	815422	1518	834	2353
GHA	2012	country	1_Afr	Ghana	23315	27578	50893	0.0281794	0.0333316	0.061511	827379	1512	1236	2748
GHA	2013	country	1_Afr	Ghana	22955	26227	49182	0.0273914	0.0312954	0.0586868	838036	1528	1013	2541
GHA	2014	country	1_Afr	Ghana	22548	24741	47289	0.0266103	0.0291979	0.0558082	847340	1529	1246	2775
GHA	2015	country	1_Afr	Ghana	22103	23653	45756	0.0258406	0.027653	0.0534936	855359	1533	1295	2829
GHA	2016	country	1_Afr	Ghana	21660	22652	44312	0.0251142	0.0262646	0.0513789	862459	1540	2073	3613
GHA	2017	country	1_Afr	Ghana	21213	21648	42861	0.0244111	0.0249112	0.0493224	868989	1494	1689	3182
GHA	2018	country	1_Afr	Ghana	20788	20817	41605	0.023745	0.023778	0.047523	875467	1522	1490	3012
GHA	2019	country	1_Afr	Ghana	20399	19852	40251	0.0231268	0.0225067	0.0456335	882051	1493	1459	2952
GIN	2000	country	1_Afr	Guinea	16520	46283	62803	0.045527	0.1275507	0.1730776	362862	627	45	672
GIN	2001	country	1_Afr	Guinea	15953	46570	62523	0.0434373	0.1268033	0.1702406	367265	641	53	694
GIN	2002	country	1_Afr	Guinea	15555	40561	56116	0.0418739	0.1091894	0.1510633	371472	636	67	703
GIN	2003	country	1_Afr	Guinea	15290	38708	53998	0.0407137	0.1030699	0.1437836	375549	657	84	741
GIN	2004	country	1_Afr	Guinea	15051	34626	49677	0.0396571	0.0912347	0.1308918	379528	665	107	772
GIN	2005	country	1_Afr	Guinea	14783	34333	49116	0.0385466	0.0895233	0.1280699	383510	662	126	788
GIN	2006	country	1_Afr	Guinea	14544	33991	48535	0.0375342	0.0877205	0.1252546	387487	670	134	804
GIN	2007	country	1_Afr	Guinea	14354	32859	47213	0.0366605	0.0839219	0.1205823	391539	676	161	837
GIN	2008	country	1_Afr	Guinea	14223	32792	47015	0.0359409	0.0828651	0.118806	395733	682	223	906
GIN	2009	country	1_Afr	Guinea	14121	32805	46926	0.0352963	0.0819992	0.1172955	400070	682	134	815
GIN	2010	country	1_Afr	Guinea	14072	31691	45763	0.0347746	0.0783141	0.1130887	404663	694	147	841
GIN	2011	country	1_Afr	Guinea	14017	31761	45778	0.0342259	0.0775531	0.111779	409543	705	161	866
GIN	2012	country	1_Afr	Guinea	13948	31820	45768	0.033633	0.0767284	0.1103614	414712	702	151	853
GIN	2013	country	1_Afr	Guinea	13873	31102	44975	0.0330181	0.0740244	0.1070425	420164	719	230	949
GIN	2014	country	1_Afr	Guinea	13814	31770	45584	0.032435	0.0745963	0.1070313	425898	724	160	884
GIN	2015	country	1_Afr	Guinea	13781	31672	45453	0.0319005	0.0733139	0.1052144	431999	733	171	904
GIN	2016	country	1_Afr	Guinea	13787	31784	45571	0.0314425	0.0724853	0.1039277	438484	747	189	935
GIN	2017	country	1_Afr	Guinea	13835	31582	45417	0.0310682	0.0709223	0.1019906	445311	765	201	966
GIN	2018	country	1_Afr	Guinea	13911	30842	44753	0.0307489	0.0681742	0.0989231	452407	768	220	988
GIN	2019	country	1_Afr	Guinea	13960	35318	49278	0.0303672	0.0768273	0.1071945	459707	784	225	1009

iso3	year	level	whoreg6	whoname	nnd	pnd	u5d	nmr	pnmr	u5mr	Livebirths	Neonatal birth defects deaths	1-59 month birth defects deaths	Under five birth defects deaths
GMB	2000	country	1_Afr	Gambia	2121	4127	6248	0.0375961	0.073154	0.1107501	56415	102	114	216
GMB	2001	country	1_Afr	Gambia	2147	3863	6010	0.0371611	0.0668708	0.104032	57775	105	185	290
GMB	2002	country	1_Afr	Gambia	2181	3670	5851	0.0368323	0.0619729	0.0988051	59214	107	136	243
GMB	2003	country	1_Afr	Gambia	2220	3541	5761	0.0365377	0.0582835	0.0948212	60759	111	143	254
GMB	2004	country	1_Afr	Gambia	2260	3309	5569	0.0362194	0.0530386	0.089258	62397	114	158	272
GMB	2005	country	1_Afr	Gambia	2296	3194	5490	0.0358175	0.0498199	0.0856374	64103	118	159	277
GMB	2006	country	1_Afr	Gambia	2327	3089	5416	0.0353308	0.0469046	0.0822354	65863	122	156	278
GMB	2007	country	1_Afr	Gambia	2352	2993	5345	0.0347314	0.0441942	0.0789256	67720	121	156	277
GMB	2008	country	1_Afr	Gambia	2377	2875	5252	0.0341464	0.0412958	0.0754421	69612	124	172	297
GMB	2009	country	1_Afr	Gambia	2396	2801	5197	0.033497	0.0391636	0.0726607	71529	130	192	323
GMB	2010	country	1_Afr	Gambia	2414	2728	5142	0.0328554	0.0371258	0.0699812	73473	133	192	324
GMB	2011	country	1_Afr	Gambia	2435	2650	5085	0.03229	0.0351422	0.0674322	75410	134	191	325
GMB	2012	country	1_Afr	Gambia	2455	2540	4995	0.0317436	0.0328468	0.0645904	77338	140	214	354
GMB	2013	country	1_Afr	Gambia	2469	2470	4939	0.0311457	0.0311637	0.0623094	79273	141	218	359
GMB	2014	country	1_Afr	Gambia	2477	2399	4876	0.0305308	0.0295644	0.0600952	81131	141	221	362
GMB	2015	country	1_Afr	Gambia	2479	2348	4827	0.0298872	0.0283116	0.0581988	82945	143	224	367
GMB	2016	country	1_Afr	Gambia	2476	2286	4762	0.0292397	0.0269951	0.0562348	84679	145	221	367
GMB	2017	country	1_Afr	Gambia	2464	2193	4657	0.0285411	0.0254076	0.0539487	86332	149	187	336
GMB	2018	country	1_Afr	Gambia	2447	2135	4582	0.0278282	0.0242807	0.0521089	87932	147	184	331
GMB	2019	country	1_Afr	Gambia	2427	2083	4510	0.0271302	0.0232892	0.0504194	89458	149	181	330
GNB	2000	country	1_Afr	Guinea-Bissau	2759	6030	8789	0.0547408	0.1196444	0.1743851	50401	87	11	98
GNB	2001	country	1_Afr	Guinea-Bissau	2747	5433	8180	0.0537447	0.1063011	0.1600457	51112	87	16	103
GNB	2002	country	1_Afr	Guinea-Bissau	2743	5651	8394	0.0528469	0.1088762	0.1617231	51905	90	23	112
GNB	2003	country	1_Afr	Guinea-Bissau	2744	8276	11020	0.0520158	0.1568771	0.208893	52753	91	30	121
GNB	2004	country	1_Afr	Guinea-Bissau	2747	6679	9426	0.0512058	0.124507	0.1757128	53646	96	38	134
GNB	2005	country	1_Afr	Guinea-Bissau	2745	4848	7593	0.050277	0.0888032	0.1390801	54598	96	44	140
GNB	2006	country	1_Afr	Guinea-Bissau	2741	4512	7253	0.0493253	0.0811934	0.1305188	55570	98	46	145
GNB	2007	country	1_Afr	Guinea-Bissau	2727	4247	6974	0.0481889	0.0750423	0.1232312	56590	103	49	152
GNB	2008	country	1_Afr	Guinea-Bissau	2705	4124	6829	0.0469401	0.0715691	0.1185093	57627	104	50	153
GNB	2009	country	1_Afr	Guinea-Bissau	2673	3982	6655	0.0455776	0.06789	0.1134677	58647	106	50	156
GNB	2010	country	1_Afr	Guinea-Bissau	2638	3817	6455	0.0442175	0.0639871	0.1082045	59660	107	63	170
GNB	2011	country	1_Afr	Guinea-Bissau	2603	3658	6261	0.0429326	0.0603279	0.1032605	60630	109	78	187
GNB	2012	country	1_Afr	Guinea-Bissau	2566	3520	6086	0.0416866	0.0571867	0.0988733	61555	109	79	188
GNB	2013	country	1_Afr	Guinea-Bissau	2532	3364	5896	0.0405665	0.0538954	0.0944619	62416	111	86	197
GNB	2014	country	1_Afr	Guinea-Bissau	2496	3265	5761	0.0394802	0.051638	0.0911182	63221	112	97	209
GNB	2015	country	1_Afr	Guinea-Bissau	2459	3541	6000	0.0384544	0.0553807	0.0938351	63946	114	104	219
GNB	2016	country	1_Afr	Guinea-Bissau	2425	3081	5506	0.0375194	0.0476619	0.0851813	64633	114	120	234
GNB	2017	country	1_Afr	Guinea-Bissau	2392	3003	5395	0.0366596	0.0460187	0.0826783	65249	116	122	237
GNB	2018	country	1_Afr	Guinea-Bissau	2358	2936	5294	0.0358205	0.0445991	0.0804196	65828	116	115	232
GNB	2019	country	1_Afr	Guinea-Bissau	2329	2884	5213	0.0350852	0.0434412	0.0785264	66381	115	115	230

iso3	year	level	whoreg6	whoname	nnd	pnd	u5d	nmr	pnmr	u5mr	Livebirths	Neonatal birth defects deaths	1-59 month birth defects deaths	Under five birth defects deaths
GNQ	2000	country	1_Afr	Equatorial Guinea	1111	2574	3685	0.0444228	0.1029046	0.1473274	25010	56	7	63
GNQ	2001	country	1_Afr	Equatorial Guinea	1136	2670	3806	0.0437469	0.1028024	0.1465493	25968	57	8	65
GNQ	2002	country	1_Afr	Equatorial Guinea	1161	2750	3911	0.0430529	0.1019922	0.1450452	26967	60	8	68
GNQ	2003	country	1_Afr	Equatorial Guinea	1180	2780	3960	0.0421687	0.0993638	0.1415326	27983	61	9	70
GNQ	2004	country	1_Afr	Equatorial Guinea	1198	2802	4000	0.0412834	0.0965407	0.1378241	29019	65	10	74
GNQ	2005	country	1_Afr	Equatorial Guinea	1211	2827	4038	0.0402283	0.0939018	0.1341301	30103	68	10	79
GNQ	2006	country	1_Afr	Equatorial Guinea	1218	2565	3783	0.0390511	0.0822332	0.1212843	31190	69	12	81
GNQ	2007	country	1_Afr	Equatorial Guinea	1225	2604	3829	0.0379156	0.0806096	0.1185252	32309	71	13	84
GNQ	2008	country	1_Afr	Equatorial Guinea	1230	2647	3877	0.0368306	0.0792504	0.116081	33396	74	15	90
GNQ	2009	country	1_Afr	Equatorial Guinea	1235	2680	3915	0.0358055	0.0777097	0.1135152	34492	76	17	93
GNQ	2010	country	1_Afr	Equatorial Guinea	1242	2426	3668	0.0349314	0.0682196	0.1031511	35555	78	18	96
GNQ	2011	country	1_Afr	Equatorial Guinea	1251	2448	3699	0.0341636	0.066854	0.1010176	36618	79	18	96
GNQ	2012	country	1_Afr	Equatorial Guinea	1261	2442	3703	0.0335117	0.0648979	0.0984096	37629	83	12	94
GNQ	2013	country	1_Afr	Equatorial Guinea	1268	2373	3641	0.0328134	0.0614079	0.0942214	38643	82	14	96
GNQ	2014	country	1_Afr	Equatorial Guinea	1277	2384	3661	0.0322113	0.0601352	0.0923465	39645	85	15	100
GNQ	2015	country	1_Afr	Equatorial Guinea	1283	2525	3808	0.0315923	0.0621839	0.0937762	40611	87	11	98
GNQ	2016	country	1_Afr	Equatorial Guinea	1286	2631	3917	0.0309335	0.063296	0.0942295	41573	87	14	101
GNQ	2017	country	1_Afr	Equatorial Guinea	1289	2263	3552	0.030293	0.0531765	0.0834695	42551	90	15	105
GNQ	2018	country	1_Afr	Equatorial Guinea	1288	2272	3560	0.0296164	0.0522431	0.0818594	43489	91	16	107
GNQ	2019	country	1_Afr	Equatorial Guinea	1289	2289	3578	0.0290052	0.0515121	0.0805173	44440	91	16	107
GRC	2000	country	4_Eur	Greece	421	265	686	0.0039286	0.0024729	0.0064014	107164	117	142	260
GRC	2001	country	4_Eur	Greece	386	255	641	0.0035845	0.002368	0.0059524	107687	123	151	273
GRC	2002	country	4_Eur	Greece	354	247	601	0.0032648	0.002278	0.0055428	108430	99	133	232
GRC	2003	country	4_Eur	Greece	325	240	565	0.002974	0.0021962	0.0051702	109280	80	135	214
GRC	2004	country	4_Eur	Greece	300	234	534	0.0027203	0.0021218	0.0048421	110284	78	127	204
GRC	2005	country	4_Eur	Greece	280	224	504	0.0025142	0.0020114	0.0045256	111367	77	109	186
GRC	2006	country	4_Eur	Greece	262	217	479	0.0023475	0.0019443	0.0042918	111609	76	115	192
GRC	2007	country	4_Eur	Greece	248	210	458	0.0022221	0.0018816	0.0041037	111606	72	117	189
GRC	2008	country	4_Eur	Greece	237	206	443	0.0021446	0.0018641	0.0040088	110508	53	109	162
GRC	2009	country	4_Eur	Greece	229	201	430	0.002111	0.0018529	0.0039638	108480	58	115	172
GRC	2010	country	4_Eur	Greece	226	193	419	0.0021356	0.0018237	0.0039593	105827	70	126	196
GRC	2011	country	4_Eur	Greece	226	185	411	0.0022026	0.001803	0.0040056	102607	79	108	187
GRC	2012	country	4_Eur	Greece	229	178	407	0.0023125	0.0017975	0.00411	99027	75	110	185
GRC	2013	country	4_Eur	Greece	235	173	408	0.0024614	0.001812	0.0042734	95474	80	91	172
GRC	2014	country	4_Eur	Greece	239	170	409	0.0025911	0.0018431	0.0044342	92237	53	51	104
GRC	2015	country	4_Eur	Greece	237	166	403	0.0026587	0.0018622	0.0045208	89143	58	43	101
GRC	2016	country	4_Eur	Greece	228	160	388	0.0026424	0.0018543	0.0044968	86284	52	51	103
GRC	2017	country	4_Eur	Greece	214	151	365	0.0025508	0.0017999	0.0043507	83895	49	44	94
GRC	2018	country	4_Eur	Greece	196	138	334	0.0024094	0.0016964	0.0041057	81350	46	41	86
GRC	2019	country	4_Eur	Greece	178	124	302	0.0022588	0.0015736	0.0038324	78802	41	37	77

iso3	year	level	whoreg6	whoname	nnd	pnd	u5d	nmr	pnmr	u5mr	Livebirths	Neonatal birth defects deaths	1-59 month birth defects deaths	Under five birth defects deaths
GRD	2000	country	2_Amr	Grenada	15	15	30	0.0080287	0.0080287	0.0160574	1868	1	1	2
GRD	2001	country	2_Amr	Grenada	15	14	29	0.0080517	0.0075149	0.0155667	1863	0	0	0
GRD	2002	country	2_Amr	Grenada	15	13	28	0.0080515	0.006978	0.0150295	1863	0	2	2
GRD	2003	country	2_Amr	Grenada	15	12	27	0.008033	0.0064264	0.0144593	1867	2	1	3
GRD	2004	country	2_Amr	Grenada	15	12	27	0.0080062	0.006405	0.0144112	1874	3	5	8
GRD	2005	country	2_Amr	Grenada	15	12	27	0.0080012	0.006401	0.0144022	1875	0	3	3
GRD	2006	country	2_Amr	Grenada	15	12	27	0.0080436	0.0064349	0.0144785	1865	3	2	5
GRD	2007	country	2_Amr	Grenada	15	12	27	0.0081416	0.0065133	0.0146549	1842	2	1	3
GRD	2008	country	2_Amr	Grenada	15	12	27	0.0083158	0.0066527	0.0149685	1804	3	0	3
GRD	2009	country	2_Amr	Grenada	16	12	28	0.0085845	0.0064384	0.0150229	1864	6	6	12
GRD	2010	country	2_Amr	Grenada	17	11	28	0.0089283	0.0057772	0.0147055	1904	2	2	4
GRD	2011	country	2_Amr	Grenada	17	11	28	0.0093169	0.0060286	0.0153456	1825	3	4	7
GRD	2012	country	2_Amr	Grenada	18	11	29	0.0097093	0.0059335	0.0156428	1854	2	3	5
GRD	2013	country	2_Amr	Grenada	19	10	29	0.0100765	0.0053034	0.0153799	1886	0	3	3
GRD	2014	country	2_Amr	Grenada	19	11	30	0.010401	0.0060216	0.0164226	1827	2	2	3
GRD	2015	country	2_Amr	Grenada	20	10	30	0.010663	0.0053315	0.0159945	1876	3	1	4
GRD	2016	country	2_Amr	Grenada	20	11	31	0.0108822	0.0059852	0.0168675	1838	4	0	4
GRD	2017	country	2_Amr	Grenada	20	11	31	0.0110228	0.0060625	0.0170853	1814	2	2	4
GRD	2018	country	2_Amr	Grenada	20	11	31	0.0110642	0.0060853	0.0171494	1808	3	1	4
GRD	2019	country	2_Amr	Grenada	20	10	30	0.0110018	0.0055009	0.0165028	1818	3	1	4
GTM	2000	country	2_Amr	Guatemala	8745	12378	21123	0.0211759	0.0299731	0.0511489	412970	652	1052	1704
GTM	2001	country	2_Amr	Guatemala	8554	11886	20440	0.0206103	0.0286385	0.0492487	415036	644	1165	1809
GTM	2002	country	2_Amr	Guatemala	8342	11384	19726	0.0200596	0.0273746	0.0474342	415861	629	1174	1803
GTM	2003	country	2_Amr	Guatemala	8117	10883	19000	0.0195372	0.0261948	0.0457319	415465	652	1212	1864
GTM	2004	country	2_Amr	Guatemala	7874	10379	18253	0.0190207	0.0250718	0.0440925	413971	668	1235	1902
GTM	2005	country	2_Amr	Guatemala	7622	9864	17486	0.018513	0.0239586	0.0424716	411711	675	1183	1857
GTM	2006	country	2_Amr	Guatemala	7370	9359	16729	0.0180058	0.0228652	0.040871	409312	693	1220	1914
GTM	2007	country	2_Amr	Guatemala	7133	8849	15982	0.0175187	0.0217332	0.0392519	407165	711	989	1700
GTM	2008	country	2_Amr	Guatemala	6920	8355	15275	0.0170572	0.0205943	0.0376515	405695	722	1173	1895
GTM	2009	country	2_Amr	Guatemala	6737	7883	14620	0.0166249	0.0194528	0.0360777	405236	749	1040	1788
GTM	2010	country	2_Amr	Guatemala	6575	7430	14005	0.0162051	0.0183124	0.0345175	405737	769	1029	1797
GTM	2011	country	2_Amr	Guatemala	6424	7037	13461	0.0157746	0.0172799	0.0330545	407237	792	841	1633
GTM	2012	country	2_Amr	Guatemala	6284	6682	12966	0.0153523	0.0163247	0.031677	409319	807	795	1602
GTM	2013	country	2_Amr	Guatemala	6157	6336	12493	0.0149538	0.0153886	0.0303424	411734	827	625	1452
GTM	2014	country	2_Amr	Guatemala	6018	6054	12072	0.0145264	0.0146133	0.0291398	414279	850	444	1294
GTM	2015	country	2_Amr	Guatemala	5842	5839	11681	0.0140167	0.0140095	0.0280263	416787	857	486	1342
GTM	2016	country	2_Amr	Guatemala	5665	5652	11317	0.0135122	0.0134812	0.0269935	419250	870	498	1368
GTM	2017	country	2_Amr	Guatemala	5497	5473	10970	0.0130375	0.0129806	0.0260181	421629	882	467	1348
GTM	2018	country	2_Amr	Guatemala	5329	5335	10664	0.0125739	0.0125881	0.025162	423814	878	446	1325
GTM	2019	country	2_Amr	Guatemala	5180	5184	10364	0.0121663	0.0121757	0.024342	425767	882	459	1340

iso3	year	level	whoreg6	whoname	nnd	pnd	u5d	nmr	pnmr	u5mr	Livebirths	Neonatal birth defects deaths	1-59 month birth defects deaths	Under five birth defects deaths
GUY	2000	country	2_Amr	Guyana	516	392	908	0.026736	0.0203111	0.0470471	19300	59	40	99
GUY	2001	country	2_Amr	Guyana	493	373	866	0.0262038	0.0198256	0.0460293	18814	53	33	86
GUY	2002	country	2_Amr	Guyana	471	353	824	0.0257439	0.0192942	0.0450381	18296	44	40	85
GUY	2003	country	2_Amr	Guyana	451	333	784	0.0253195	0.0186949	0.0440144	17812	40	54	94
GUY	2004	country	2_Amr	Guyana	432	315	747	0.0249016	0.0181574	0.043059	17348	36	52	87
GUY	2005	country	2_Amr	Guyana	414	298	712	0.0244888	0.0176272	0.0421161	16906	40	28	68
GUY	2006	country	2_Amr	Guyana	398	283	681	0.0240493	0.0171004	0.0411497	16549	51	36	87
GUY	2007	country	2_Amr	Guyana	385	269	654	0.0236613	0.0165322	0.0401935	16271	31	46	77
GUY	2008	country	2_Amr	Guyana	375	256	631	0.0232724	0.0158873	0.0391596	16114	37	27	64
GUY	2009	country	2_Amr	Guyana	366	245	611	0.022894	0.0153252	0.0382192	15987	60	25	85
GUY	2010	country	2_Amr	Guyana	358	237	595	0.0224978	0.0148938	0.0373916	15913	52	28	80
GUY	2011	country	2_Amr	Guyana	352	228	580	0.0221597	0.0143534	0.0365131	15885	32	24	57
GUY	2012	country	2_Amr	Guyana	345	221	566	0.0217205	0.0139137	0.0356342	15884	40	24	65
GUY	2013	country	2_Amr	Guyana	339	215	554	0.021312	0.0135165	0.0348285	15907	41	32	73
GUY	2014	country	2_Amr	Guyana	332	207	539	0.0209219	0.0130447	0.0339666	15869	47	37	84
GUY	2015	country	2_Amr	Guyana	324	199	523	0.0204987	0.0125902	0.0330889	15806	41	31	72
GUY	2016	country	2_Amr	Guyana	315	191	506	0.0200377	0.0121498	0.0321875	15720	41	30	71
GUY	2017	country	2_Amr	Guyana	306	183	489	0.0195761	0.0117073	0.0312833	15631	40	28	68
GUY	2018	country	2_Amr	Guyana	297	174	471	0.0190889	0.0111834	0.0302723	15559	38	26	65
GUY	2019	country	2_Amr	Guyana	287	166	453	0.0186002	0.0107583	0.0293585	15430	37	25	63
HND	2000	country	2_Amr	Honduras	3857	4288	8145	0.0173536	0.0192928	0.0366465	222259	413	716	1129
HND	2001	country	2_Amr	Honduras	3773	4020	7793	0.0169847	0.0180966	0.0350813	222141	413	715	1128
HND	2002	country	2_Amr	Honduras	3678	3749	7427	0.0166066	0.0169271	0.0335337	221479	419	609	1029
HND	2003	country	2_Amr	Honduras	3579	3487	7066	0.0162351	0.0158177	0.0320528	220449	417	496	913
HND	2004	country	2_Amr	Honduras	3475	3236	6711	0.0158546	0.0147641	0.0306187	219180	422	399	820
HND	2005	country	2_Amr	Honduras	3374	2992	6366	0.015496	0.0137416	0.0292376	217733	420	355	775
HND	2006	country	2_Amr	Honduras	3231	2806	6037	0.0149528	0.0129859	0.0279387	216080	419	298	718
HND	2007	country	2_Amr	Honduras	3085	2639	5724	0.0144045	0.0123221	0.0267266	214169	425	254	679
HND	2008	country	2_Amr	Honduras	2942	2482	5424	0.0138742	0.0117049	0.0255791	212049	427	218	645
HND	2009	country	2_Amr	Honduras	2794	2347	5141	0.013306	0.0111773	0.0244833	209980	423	202	625
HND	2010	country	2_Amr	Honduras	2654	2223	4877	0.0127615	0.0106891	0.0234506	207969	418	204	621
HND	2011	country	2_Amr	Honduras	2525	2113	4638	0.0122379	0.0102411	0.0224791	206325	415	206	620
HND	2012	country	2_Amr	Honduras	2411	2016	4427	0.0117525	0.0098271	0.0215796	205147	407	208	615
HND	2013	country	2_Amr	Honduras	2318	1924	4242	0.011334	0.0094075	0.0207415	204517	412	209	621
HND	2014	country	2_Amr	Honduras	2232	1850	4082	0.0109204	0.0090514	0.0199719	204388	409	212	621
HND	2015	country	2_Amr	Honduras	2153	1781	3934	0.0105173	0.0087001	0.0192173	204711	406	214	620
HND	2016	country	2_Amr	Honduras	2080	1728	3808	0.0101229	0.0084098	0.0185327	205475	408	217	625
HND	2017	country	2_Amr	Honduras	2021	1663	3684	0.0097918	0.0080573	0.0178491	206397	396	218	614
HND	2018	country	2_Amr	Honduras	1965	1619	3584	0.0094769	0.0078082	0.0172852	207345	389	221	611
HND	2019	country	2_Amr	Honduras	1907	1573	3480	0.009164	0.007559	0.016723	208097	387	224	611

iso3	year	level	whoreg6	whoname	nnd	pnd	u5d	nmr	pnmr	u5mr	Livebirths	Neonatal birth defects deaths	1-59 month birth defects deaths	Under five birth defects deaths
HRV	2000	country	4_Eur	Croatia	251	131	382	0.0055579	0.0029008	0.0084587	45161	73	42	115
HRV	2001	country	4_Eur	Croatia	233	126	359	0.0053016	0.0028669	0.0081685	43949	70	53	123
HRV	2002	country	4_Eur	Croatia	215	121	336	0.0050156	0.0028227	0.0078383	42867	58	46	104
HRV	2003	country	4_Eur	Croatia	200	116	316	0.0047304	0.0027436	0.007474	42280	52	40	92
HRV	2004	country	4_Eur	Croatia	189	110	299	0.0044901	0.0026133	0.0071033	42093	53	41	94
HRV	2005	country	4_Eur	Croatia	179	106	285	0.0042309	0.0025054	0.0067363	42308	64	44	108
HRV	2006	country	4_Eur	Croatia	171	102	273	0.0040071	0.0023902	0.0063972	42675	57	44	101
HRV	2007	country	4_Eur	Croatia	167	96	263	0.0038827	0.0022319	0.0061146	43012	52	30	82
HRV	2008	country	4_Eur	Croatia	163	90	253	0.0037744	0.002084	0.0058585	43185	48	27	75
HRV	2009	country	4_Eur	Croatia	159	85	244	0.0036765	0.0019654	0.005642	43247	60	30	90
HRV	2010	country	4_Eur	Croatia	152	82	234	0.0035483	0.0019142	0.0054625	42838	39	33	72
HRV	2011	country	4_Eur	Croatia	143	82	225	0.0033795	0.0019379	0.0053175	42313	37	23	60
HRV	2012	country	4_Eur	Croatia	136	80	216	0.0032747	0.0019263	0.0052011	41530	36	27	64
HRV	2013	country	4_Eur	Croatia	132	77	209	0.0032313	0.0018849	0.0051162	40851	29	26	55
HRV	2014	country	4_Eur	Croatia	128	74	202	0.0032016	0.0018509	0.0050525	39980	38	30	68
HRV	2015	country	4_Eur	Croatia	122	74	196	0.0031147	0.0018892	0.0050039	39169	37	32	69
HRV	2016	country	4_Eur	Croatia	116	75	191	0.0030197	0.0019524	0.0049721	38414	46	28	74
HRV	2017	country	4_Eur	Croatia	111	75	186	0.0029371	0.0019846	0.0049217	37792	37	30	67
HRV	2018	country	4_Eur	Croatia	108	73	181	0.0029429	0.0019892	0.0049321	36698	37	30	67
HRV	2019	country	4_Eur	Croatia	105	71	176	0.0029187	0.0019736	0.0048924	35974	37	29	66
HTI	2000	country	2_Amr	Haiti	8000	19498	27498	0.0297401	0.0724841	0.1022243	268997	438	288	727
HTI	2001	country	2_Amr	Haiti	7930	18753	26683	0.0293973	0.0695191	0.0989164	269753	436	296	732
HTI	2002	country	2_Amr	Haiti	7908	18026	25934	0.0292351	0.0666404	0.0958755	270497	451	308	759
HTI	2003	country	2_Amr	Haiti	7943	17316	25259	0.0292853	0.0638428	0.0931281	271229	449	320	769
HTI	2004	country	2_Amr	Haiti	7996	16656	24652	0.0293994	0.0612403	0.0906397	271978	464	323	787
HTI	2005	country	2_Amr	Haiti	8023	16086	24109	0.0294248	0.0589963	0.0884211	272661	462	332	795
HTI	2006	country	2_Amr	Haiti	8035	15567	23602	0.0293997	0.056959	0.0863587	273302	477	343	820
HTI	2007	country	2_Amr	Haiti	8013	15093	23106	0.0292548	0.0551033	0.0843582	273904	485	356	841
HTI	2008	country	2_Amr	Haiti	7978	14651	22629	0.0290727	0.0533899	0.0824627	274415	487	375	862
HTI	2009	country	2_Amr	Haiti	7928	14232	22160	0.0288456	0.0517824	0.0806281	274842	489	387	876
HTI	2010	country	2_Amr	Haiti	8474	50293	58767	0.0308031	0.1828155	0.2136185	275103	499	395	894
HTI	2011	country	2_Amr	Haiti	7819	13166	20985	0.0284141	0.047845	0.0762591	275180	507	412	919
HTI	2012	country	2_Amr	Haiti	7747	12856	20603	0.0281665	0.0467418	0.0749084	275043	512	432	944
HTI	2013	country	2_Amr	Haiti	7659	12483	20142	0.0278822	0.0454437	0.0733258	274692	523	467	990
HTI	2014	country	2_Amr	Haiti	7558	12109	19667	0.0275687	0.0441691	0.0717378	274151	517	489	1006
HTI	2015	country	2_Amr	Haiti	7420	11756	19176	0.0271344	0.0429909	0.0701253	273453	531	497	1028
HTI	2016	country	2_Amr	Haiti	7278	11306	18584	0.0266951	0.0414694	0.0681645	272634	529	489	1018
HTI	2017	country	2_Amr	Haiti	7139	10880	18019	0.0262704	0.0400367	0.0663072	271750	524	481	1006
HTI	2018	country	2_Amr	Haiti	6987	10457	17444	0.0258019	0.038616	0.0644179	270795	520	476	995
HTI	2019	country	2_Amr	Haiti	6827	10063	16890	0.0252971	0.0372879	0.0625849	269873	499	467	966

iso3	year	level	whoreg6	whoname	nnd	pnd	u5d	nmr	pnmr	u5mr	Livebirths	Neonatal birth defects deaths	1-59 month birth defects deaths	Under five birth defects deaths
HUN	2000	country	4_Eur	Hungary	552	438	990	0.0057511	0.0045634	0.0103144	95982	108	129	237
HUN	2001	country	4_Eur	Hungary	519	398	917	0.0054473	0.0041773	0.0096246	95276	110	123	233
HUN	2002	country	4_Eur	Hungary	486	368	854	0.0051149	0.003873	0.008988	95016	95	106	201
HUN	2003	country	4_Eur	Hungary	451	348	799	0.0047514	0.0036663	0.0084177	94919	86	98	185
HUN	2004	country	4_Eur	Hungary	419	335	754	0.0044019	0.0035194	0.0079213	95187	72	84	156
HUN	2005	country	4_Eur	Hungary	395	323	718	0.0041491	0.0033928	0.007542	95201	92	95	187
HUN	2006	country	4_Eur	Hungary	375	310	685	0.0039436	0.0032601	0.0072037	95090	102	90	192
HUN	2007	country	4_Eur	Hungary	358	293	651	0.0037716	0.0030868	0.0068585	94919	86	86	171
HUN	2008	country	4_Eur	Hungary	342	273	615	0.0036328	0.0028999	0.0065327	94141	72	80	152
HUN	2009	country	4_Eur	Hungary	326	256	582	0.0034959	0.0027453	0.0062412	93252	73	93	166
HUN	2010	country	4_Eur	Hungary	311	246	557	0.0033701	0.0026658	0.0060359	92281	75	86	161
HUN	2011	country	4_Eur	Hungary	298	241	539	0.0032626	0.0026386	0.0059012	91337	78	75	152
HUN	2012	country	4_Eur	Hungary	286	239	525	0.003155	0.0026365	0.0057915	90650	66	63	128
HUN	2013	country	4_Eur	Hungary	275	235	510	0.0030478	0.0026045	0.0056523	90229	56	64	121
HUN	2014	country	4_Eur	Hungary	262	228	490	0.0029044	0.0025275	0.0054319	90208	65	73	137
HUN	2015	country	4_Eur	Hungary	248	216	464	0.0027342	0.0023814	0.0051156	90702	63	61	124
HUN	2016	country	4_Eur	Hungary	231	200	431	0.0025308	0.0021912	0.004722	91275	60	70	130
HUN	2017	country	4_Eur	Hungary	214	183	397	0.0023407	0.0020016	0.0043423	91426	56	58	114
HUN	2018	country	4_Eur	Hungary	198	167	365	0.0021592	0.0018211	0.0039804	91700	51	53	104
HUN	2019	country	4_Eur	Hungary	182	153	335	0.0019873	0.0016707	0.003658	91580	47	48	96
IDN	2000	country	3_Sear	Indonesia	104919	134113	239032	0.0227603	0.0290934	0.0518538	4609728	8976	9823	18799
IDN	2001	country	3_Sear	Indonesia	102905	127820	230725	0.0219818	0.027304	0.0492858	4681380	9070	6841	15911
IDN	2002	country	3_Sear	Indonesia	101347	119466	220813	0.0213147	0.0251254	0.0464401	4754788	9180	6266	15445
IDN	2003	country	3_Sear	Indonesia	100019	115839	215858	0.0207407	0.0240211	0.0447618	4822361	9291	6585	15876
IDN	2004	country	3_Sear	Indonesia	99189	142964	242153	0.0203265	0.0292972	0.0496237	4879792	9388	6864	16252
IDN	2005	country	3_Sear	Indonesia	97328	115883	213211	0.0197613	0.0235286	0.0432899	4925191	9597	6924	16521
IDN	2006	country	3_Sear	Indonesia	95731	114951	210682	0.0193032	0.0231788	0.042482	4959330	9914	7126	17041
IDN	2007	country	3_Sear	Indonesia	94031	96402	190433	0.0188596	0.0193352	0.0381947	4985848	10238	6503	16741
IDN	2008	country	3_Sear	Indonesia	92091	87527	179618	0.0183915	0.0174801	0.0358716	5007251	10490	6341	16831
IDN	2009	country	3_Sear	Indonesia	89913	88247	178160	0.0178985	0.0175669	0.0354655	5023488	10738	5897	16635
IDN	2010	country	3_Sear	Indonesia	87474	84262	171736	0.0173798	0.0167416	0.0341213	5033089	10872	6386	17258
IDN	2011	country	3_Sear	Indonesia	84801	83156	167958	0.0168449	0.0165183	0.0333632	5034214	10943	6751	17694
IDN	2012	country	3_Sear	Indonesia	81867	76174	158041	0.01629	0.0151572	0.0314471	5025612	11063	7156	18219
IDN	2013	country	3_Sear	Indonesia	78781	72146	150927	0.0157332	0.0144081	0.0301413	5007307	11110	7372	18483
IDN	2014	country	3_Sear	Indonesia	75553	70545	146098	0.0151696	0.0141641	0.0293338	4980545	11029	7470	18499
IDN	2015	country	3_Sear	Indonesia	71869	69160	141029	0.0145273	0.0139796	0.0285069	4947183	10973	8062	19035
IDN	2016	country	3_Sear	Indonesia	68412	63583	131995	0.0139336	0.0129502	0.0268838	4909852	10894	8539	19432
IDN	2017	country	3_Sear	Indonesia	65254	61089	126343	0.0133951	0.0125402	0.0259353	4871493	10807	8927	19734
IDN	2018	country	3_Sear	Indonesia	62270	57984	120254	0.0128799	0.0119934	0.0248733	4834669	10669	9314	19983
IDN	2019	country	3_Sear	Indonesia	59591	53439	113030	0.0124125	0.011131	0.0235434	4800899	10486	9281	19767

iso3	year	level	whoreg6	whoname	nnd	pnd	u5d	nmr	pnmr	u5mr	Livebirths	Neonatal birth defects deaths	1-59 month birth defects deaths	Under five birth defects deaths
IND	2000	country	3_Sear	India	1254908	1278727	2533635	0.0449919	0.0458459	0.0908378	27900000	64158	18888	83046
IND	2001	country	3_Sear	India	1215240	1239343	2454583	0.0435763	0.0444406	0.0880169	27900000	64830	19458	84288
IND	2002	country	3_Sear	India	1174949	1170130	2345079	0.0421605	0.0419876	0.0841481	27900000	64763	29512	94275
IND	2003	country	3_Sear	India	1134823	1121733	2256556	0.040778	0.0403076	0.0810856	27800000	64893	29648	94541
IND	2004	country	3_Sear	India	1094376	1079289	2173665	0.0394202	0.0388767	0.0782969	27800000	63993	29747	93740
IND	2005	country	3_Sear	India	1053741	985351	2039092	0.0381151	0.0356414	0.0737565	27600000	63813	29804	93617
IND	2006	country	3_Sear	India	1012132	958440	1970572	0.0368587	0.0349034	0.0717622	27500000	65035	31867	96902
IND	2007	country	3_Sear	India	969440	880713	1850153	0.0356479	0.0323853	0.0680332	27200000	62824	23086	85910
IND	2008	country	3_Sear	India	924724	824228	1748952	0.0344325	0.0306905	0.0651229	26900000	62317	34232	96549
IND	2009	country	3_Sear	India	878704	779098	1657802	0.033212	0.0294472	0.0626592	26500000	60932	34390	95322
IND	2010	country	3_Sear	India	832286	697448	1529734	0.0319796	0.0267986	0.0587782	26000000	59798	23776	83574
IND	2011	country	3_Sear	India	786477	640122	1426599	0.030728	0.0250098	0.0557378	25600000	59995	24440	84435
IND	2012	country	3_Sear	India	743101	570262	1313363	0.0294898	0.0226307	0.0521205	25200000	56583	25319	81902
IND	2013	country	3_Sear	India	702601	513266	1215867	0.0282596	0.0206443	0.0489038	24900000	55402	23760	79163
IND	2014	country	3_Sear	India	665584	483051	1148635	0.0270574	0.019637	0.0466944	24600000	54684	22120	76804
IND	2015	country	3_Sear	India	631542	442808	1074349	0.0258703	0.0181391	0.0440094	24400000	52626	20831	73457
IND	2016	country	3_Sear	India	600955	398363	999318	0.0247384	0.0163987	0.041137	24300000	50577	19826	70402
IND	2017	country	3_Sear	India	572765	362873	935638	0.0236524	0.0149849	0.0386373	24200000	49199	19034	68233
IND	2018	country	3_Sear	India	546427	333134	879561	0.0226178	0.0137892	0.036407	24200000	49003	18409	67412
IND	2019	country	3_Sear	India	522249	297763	820012	0.0216603	0.0123498	0.0340101	24100000	46915	17278	64193
IRL	2000	country	4_Eur	Ireland	222	165	387	0.0040023	0.0029747	0.006977	55468	111	51	162
IRL	2001	country	4_Eur	Ireland	224	160	384	0.0039196	0.0027997	0.0067192	57149	112	53	165
IRL	2002	country	4_Eur	Ireland	222	152	374	0.0037397	0.0025605	0.0063001	59364	111	50	160
IRL	2003	country	4_Eur	Ireland	216	144	360	0.0035176	0.0023451	0.0058627	61405	107	53	160
IRL	2004	country	4_Eur	Ireland	207	139	346	0.0032433	0.0021779	0.0054211	63824	105	39	144
IRL	2005	country	4_Eur	Ireland	197	135	332	0.0029939	0.0020517	0.0050455	65801	97	44	141
IRL	2006	country	4_Eur	Ireland	187	134	321	0.0027524	0.0019723	0.0047248	67940	91	50	141
IRL	2007	country	4_Eur	Ireland	180	134	314	0.0025901	0.0019282	0.0045183	69495	98	46	144
IRL	2008	country	4_Eur	Ireland	177	132	309	0.0025	0.0018644	0.0043643	70801	77	59	136
IRL	2009	country	4_Eur	Ireland	177	127	304	0.002461	0.0017658	0.0042267	71923	85	45	130
IRL	2010	country	4_Eur	Ireland	179	119	298	0.0024816	0.0016498	0.0041313	72132	80	44	124
IRL	2011	country	4_Eur	Ireland	181	111	292	0.0025251	0.0015486	0.0040737	71679	86	52	138
IRL	2012	country	4_Eur	Ireland	179	105	284	0.0025111	0.001473	0.0039842	71282	76	39	116
IRL	2013	country	4_Eur	Ireland	176	99	275	0.0025169	0.0014158	0.0039327	69927	78	43	121
IRL	2014	country	4_Eur	Ireland	169	96	265	0.0024538	0.0013939	0.0038477	68873	73	45	118
IRL	2015	country	4_Eur	Ireland	161	92	253	0.0024012	0.0013721	0.0037734	67049	70	39	109
IRL	2016	country	4_Eur	Ireland	152	89	241	0.0023252	0.0013615	0.0036867	65369	67	38	104
IRL	2017	country	4_Eur	Ireland	144	84	228	0.0022556	0.0013157	0.0035713	63842	63	36	98
IRL	2018	country	4_Eur	Ireland	135	80	215	0.0021762	0.0012896	0.0034659	62033	59	34	93
IRL	2019	country	4_Eur	Ireland	128	75	203	0.0021035	0.0012325	0.0033361	60850	56	32	88

iso3	year	level	whoreg6	whoname	nnd	pnd	u5d	nmr	pnmr	u5mr	Livebirths	Neonatal birth defects deaths	1-59 month birth defects deaths	Under five birth defects deaths
IRN	2000	country	5_Emr	Iran (Islamic Repub	21810	18582	40392	0.0187674	0.0159897	0.0347571	1162121	2592	4708	7299
IRN	2001	country	5_Emr	Iran (Islamic Repub	21129	16590	37719	0.0182296	0.0143135	0.0325431	1159047	2649	4068	6717
IRN	2002	country	5_Emr	Iran (Islamic Repub	20559	14980	35539	0.0176527	0.0128624	0.030515	1164639	2744	3877	6621
IRN	2003	country	5_Emr	Iran (Islamic Repub	20061	13683	33744	0.0170497	0.0116291	0.0286788	1176618	2847	3623	6470
IRN	2004	country	5_Emr	Iran (Islamic Repub	19467	12746	32213	0.0163142	0.0106817	0.0269958	1193259	2933	3460	6393
IRN	2005	country	5_Emr	Iran (Islamic Repub	18915	11980	30895	0.0155904	0.0098743	0.0254647	1213247	2990	3312	6302
IRN	2006	country	5_Emr	Iran (Islamic Repub	18399	11310	29709	0.0148919	0.0091541	0.024046	1235506	3158	3334	6492
IRN	2007	country	5_Emr	Iran (Islamic Repub	17867	10715	28582	0.0141824	0.0085053	0.0226876	1259805	3271	3258	6529
IRN	2008	country	5_Emr	Iran (Islamic Repub	17379	10177	27556	0.0135177	0.0079159	0.0214336	1285646	3359	3184	6543
IRN	2009	country	5_Emr	Iran (Islamic Repub	16881	9777	26658	0.0128637	0.0074503	0.020314	1312296	3457	3137	6594
IRN	2010	country	5_Emr	Iran (Islamic Repub	16408	9496	25904	0.012244	0.0070861	0.0193301	1340083	3585	3114	6699
IRN	2011	country	5_Emr	Iran (Islamic Repub	16025	9246	25271	0.0116965	0.0067485	0.018445	1370073	3656	3091	6747
IRN	2012	country	5_Emr	Iran (Islamic Repub	15700	9079	24779	0.0111942	0.0064734	0.0176677	1402506	3719	3087	6806
IRN	2013	country	5_Emr	Iran (Islamic Repub	15421	8960	24381	0.0107354	0.0062376	0.016973	1436458	3851	3094	6945
IRN	2014	country	5_Emr	Iran (Islamic Repub	15186	8827	24013	0.0103302	0.0060045	0.0163348	1470054	3906	3094	6999
IRN	2015	country	5_Emr	Iran (Islamic Repub	14942	8697	23639	0.0099615	0.0057981	0.0157596	1499977	3862	3125	6987
IRN	2016	country	5_Emr	Iran (Islamic Repub	14601	8604	23205	0.0095919	0.0056523	0.0152442	1522221	3771	3135	6906
IRN	2017	country	5_Emr	Iran (Islamic Repub	14166	8499	22665	0.0092314	0.0055384	0.0147698	1534551	3783	3137	6920
IRN	2018	country	5_Emr	Iran (Islamic Repub	13633	8405	22038	0.0088793	0.0054742	0.0143535	1535372	3659	3139	6798
IRN	2019	country	5_Emr	Iran (Islamic Repub	13075	8173	21248	0.0085758	0.0053606	0.0139365	1524633	3510	3088	6598
IRQ	2000	country	5_Emr	Iraq	19821	16456	36277	0.02357	0.0195689	0.0431389	840942	1801	1826	3627
IRQ	2001	country	5_Emr	Iraq	19924	16371	36295	0.0232694	0.0191202	0.0423895	856233	1850	1693	3544
IRQ	2002	country	5_Emr	Iraq	19979	16499	36478	0.0229598	0.0189609	0.0419207	870173	1910	1529	3439
IRQ	2003	country	5_Emr	Iraq	19994	15842	35836	0.0226346	0.0179337	0.0405682	883340	1889	1401	3291
IRQ	2004	country	5_Emr	Iraq	19955	16088	36043	0.0222677	0.0179524	0.0402201	896141	1927	1286	3213
IRQ	2005	country	5_Emr	Iraq	19912	15405	35317	0.0218872	0.0169336	0.0388208	909755	1928	1284	3212
IRQ	2006	country	5_Emr	Iraq	19879	15209	35088	0.021478	0.0164321	0.0379101	925554	1927	1096	3022
IRQ	2007	country	5_Emr	Iraq	19882	14981	34863	0.0210599	0.015868	0.036928	944068	1937	1142	3079
IRQ	2008	country	5_Emr	Iraq	19900	14731	34631	0.0206152	0.0152608	0.035876	965307	1987	1480	3467
IRQ	2009	country	5_Emr	Iraq	19938	15052	34990	0.0201636	0.0152223	0.035386	988811	2044	1638	3681
IRQ	2010	country	5_Emr	Iraq	19950	14265	34215	0.0196914	0.01408	0.0337714	1013131	2096	1461	3557
IRQ	2011	country	5_Emr	Iraq	19936	14006	33942	0.0192387	0.0135163	0.032755	1036246	2212	1735	3947
IRQ	2012	country	5_Emr	Iraq	19850	13809	33659	0.0187906	0.0130724	0.031863	1056379	2306	1746	4053
IRQ	2013	country	5_Emr	Iraq	19676	13470	33146	0.0183441	0.0125582	0.0309022	1072608	2409	1879	4288
IRQ	2014	country	5_Emr	Iraq	19401	13131	32532	0.0178858	0.0121054	0.0299913	1084713	2497	1965	4462
IRQ	2015	country	5_Emr	Iraq	19064	12789	31853	0.0174363	0.0116973	0.0291335	1093354	2583	2163	4747
IRQ	2016	country	5_Emr	Iraq	18587	12492	31079	0.0168979	0.0113565	0.0282545	1099957	2681	2418	5099
IRQ	2017	country	5_Emr	Iraq	18070	12250	30320	0.0163324	0.0110716	0.027404	1106391	2752	2613	5365
IRQ	2018	country	5_Emr	Iraq	17625	11934	29559	0.015819	0.010711	0.0265299	1114168	2799	2692	5491
IRQ	2019	country	5_Emr	Iraq	17203	11632	28835	0.0153065	0.0103499	0.0256564	1123900	2839	2688	5528

iso3	year	level	whoreg6	whoname	nnd	pnd	u5d	nmr	pnmr	u5mr	Livebirths	Neonatal birth defects deaths	1-59 month birth defects deaths	Under five birth defects deaths
ISL	2000	country	4_Eur	Iceland	9	8	17	0.0020959	0.001863	0.0039589	4294	1	4	5
ISL	2001	country	4_Eur	Iceland	8	8	16	0.0019754	0.0019754	0.0039508	4050	0	1	1
ISL	2002	country	4_Eur	Iceland	8	7	15	0.0018627	0.0016299	0.0034926	4295	5	1	6
ISL	2003	country	4_Eur	Iceland	7	7	14	0.0017608	0.0017608	0.0035215	3976	2	0	2
ISL	2004	country	4_Eur	Iceland	7	7	14	0.0016689	0.0016689	0.0033377	4194	4	0	4
ISL	2005	country	4_Eur	Iceland	7	7	14	0.0015885	0.0015885	0.0031771	4407	2	1	3
ISL	2006	country	4_Eur	Iceland	7	6	13	0.0015185	0.0013015	0.00282	4610	2	0	2
ISL	2007	country	4_Eur	Iceland	7	6	13	0.0014586	0.0012502	0.0027089	4799	1	1	3
ISL	2008	country	4_Eur	Iceland	6	7	13	0.001406	0.0016403	0.0030462	4268	1	0	1
ISL	2009	country	4_Eur	Iceland	6	6	12	0.001359	0.001359	0.002718	4415	0	2	2
ISL	2010	country	4_Eur	Iceland	6	6	12	0.0013143	0.0013143	0.0026285	4565	2	1	3
ISL	2011	country	4_Eur	Iceland	6	6	12	0.0012727	0.0012727	0.0025453	4715	0	0	0
ISL	2012	country	4_Eur	Iceland	6	5	11	0.0012339	0.0010282	0.0022621	4863	0	1	1
ISL	2013	country	4_Eur	Iceland	5	6	11	0.0011962	0.0014355	0.0026317	4180	2	0	2
ISL	2014	country	4_Eur	Iceland	5	5	10	0.0011614	0.0011614	0.0023229	4305	2	2	3
ISL	2015	country	4_Eur	Iceland	5	5	10	0.0011271	0.0011271	0.0022542	4436	1	1	2
ISL	2016	country	4_Eur	Iceland	5	4	9	0.0010937	0.0008749	0.0019686	4572	1	1	2
ISL	2017	country	4_Eur	Iceland	4	5	9	0.0010619	0.0013274	0.0023893	3767	1	0	1
ISL	2018	country	4_Eur	Iceland	4	4	8	0.0010275	0.0010275	0.002055	3893	1	1	2
ISL	2019	country	4_Eur	Iceland	4	4	8	0.0009952	0.0009952	0.0019904	4019	1	1	2
ISR	2000	country	4_Eur	Israel	452	397	849	0.0035885	0.0031519	0.0067404	125958	138	85	223
ISR	2001	country	4_Eur	Israel	445	390	835	0.0034652	0.0030369	0.0065022	128419	154	100	254
ISR	2002	country	4_Eur	Israel	436	384	820	0.0033292	0.0029322	0.0062614	130960	129	97	225
ISR	2003	country	4_Eur	Israel	421	381	802	0.0031556	0.0028558	0.0060113	133415	132	100	232
ISR	2004	country	4_Eur	Israel	400	381	781	0.0029377	0.0027981	0.0057358	136163	154	101	255
ISR	2005	country	4_Eur	Israel	380	380	760	0.0027306	0.0027306	0.0054611	139165	138	142	280
ISR	2006	country	4_Eur	Israel	368	373	741	0.0025895	0.0026247	0.0052142	142113	122	111	234
ISR	2007	country	4_Eur	Israel	363	363	726	0.0024948	0.0024948	0.0049896	145504	135	125	261
ISR	2008	country	4_Eur	Israel	364	350	714	0.0024386	0.0023448	0.0047834	149265	132	115	247
ISR	2009	country	4_Eur	Israel	369	335	704	0.0024158	0.0021932	0.0046091	152742	128	135	262
ISR	2010	country	4_Eur	Israel	373	323	696	0.0023906	0.0020702	0.0044608	156026	123	113	236
ISR	2011	country	4_Eur	Israel	373	314	687	0.0023421	0.0019716	0.0043137	159260	131	112	243
ISR	2012	country	4_Eur	Israel	369	308	677	0.0022744	0.0018984	0.0041728	162240	136	120	256
ISR	2013	country	4_Eur	Israel	363	305	668	0.0022036	0.0018515	0.0040551	164731	134	100	234
ISR	2014	country	4_Eur	Israel	357	303	660	0.0021399	0.0018162	0.0039562	166828	132	116	248
ISR	2015	country	4_Eur	Israel	351	301	652	0.0020851	0.0017881	0.0038733	168333	126	123	249
ISR	2016	country	4_Eur	Israel	345	300	645	0.0020378	0.001772	0.0038097	169303	117	116	233
ISR	2017	country	4_Eur	Israel	339	300	639	0.0019973	0.0017676	0.0037649	169725	121	118	239
ISR	2018	country	4_Eur	Israel	334	298	632	0.0019642	0.0017524	0.0037166	170048	117	117	235
ISR	2019	country	4_Eur	Israel	329	295	624	0.0019351	0.0017351	0.0036702	170019	115	116	231

iso3	year	level	whoreg6	whoname	nnd	pnd	u5d	nmr	pnmr	u5mr	Livebirths	Neonatal birth defects deaths	1-59 month birth defects deaths	Under five birth defects deaths
ITA	2000	country	4_Eur	Italy	1847	1117	2964	0.0034584	0.0020915	0.0055499	534067	530	460	990
ITA	2001	country	4_Eur	Italy	1756	1081	2837	0.0032571	0.0020051	0.0052622	539133	506	418	924
ITA	2002	country	4_Eur	Italy	1683	1046	2729	0.0030857	0.0019178	0.0050035	545414	466	400	866
ITA	2003	country	4_Eur	Italy	1623	1016	2639	0.0029398	0.0018403	0.0047801	552086	456	372	829
ITA	2004	country	4_Eur	Italy	1574	989	2563	0.0028172	0.0017701	0.0045873	558719	388	329	717
ITA	2005	country	4_Eur	Italy	1530	970	2500	0.0027128	0.0017199	0.0044327	563994	420	331	751
ITA	2006	country	4_Eur	Italy	1490	957	2447	0.0026249	0.001686	0.0043109	567630	411	339	750
ITA	2007	country	4_Eur	Italy	1452	949	2401	0.0025507	0.0016671	0.0042178	569260	350	312	662
ITA	2008	country	4_Eur	Italy	1416	937	2353	0.0024916	0.0016488	0.0041404	568305	380	301	682
ITA	2009	country	4_Eur	Italy	1381	915	2296	0.0024471	0.0016214	0.0040684	564343	324	268	592
ITA	2010	country	4_Eur	Italy	1342	884	2226	0.0024069	0.0015855	0.0039924	557555	306	263	569
ITA	2011	country	4_Eur	Italy	1294	851	2145	0.00236	0.0015521	0.003912	548306	351	262	613
ITA	2012	country	4_Eur	Italy	1234	818	2052	0.0022984	0.0015236	0.003822	536895	291	247	538
ITA	2013	country	4_Eur	Italy	1170	784	1954	0.002233	0.0014963	0.0037292	523967	259	261	520
ITA	2014	country	4_Eur	Italy	1111	744	1855	0.0021778	0.0014584	0.0036362	510149	274	227	501
ITA	2015	country	4_Eur	Italy	1055	702	1757	0.0021273	0.0014155	0.0035428	495934	250	234	484
ITA	2016	country	4_Eur	Italy	1002	664	1666	0.0020771	0.0013765	0.0034536	482400	235	215	451
ITA	2017	country	4_Eur	Italy	951	633	1584	0.0020249	0.0013478	0.0033726	469664	228	205	433
ITA	2018	country	4_Eur	Italy	903	603	1506	0.0019747	0.0013186	0.0032933	457292	214	196	410
ITA	2019	country	4_Eur	Italy	858	574	1432	0.0019186	0.0012835	0.0032021	447207	203	186	390
JAM	2000	country	2_Amr	Jamaica	946	312	1258	0.0167047	0.0055094	0.022214	56631	121	34	155
JAM	2001	country	2_Amr	Jamaica	905	300	1205	0.0162692	0.0053931	0.0216624	55626	116	35	151
JAM	2002	country	2_Amr	Jamaica	865	291	1156	0.0158225	0.0053229	0.0211454	54669	110	38	148
JAM	2003	country	2_Amr	Jamaica	826	288	1114	0.0153517	0.0053526	0.0207043	53805	106	43	149
JAM	2004	country	2_Amr	Jamaica	792	287	1079	0.0149273	0.0054093	0.0203366	53057	102	48	150
JAM	2005	country	2_Amr	Jamaica	764	286	1050	0.014562	0.0054512	0.0200132	52465	95	43	138
JAM	2006	country	2_Amr	Jamaica	740	284	1024	0.0142469	0.0054677	0.0197147	51941	98	80	178
JAM	2007	country	2_Amr	Jamaica	719	280	999	0.0139882	0.0054474	0.0194356	51401	93	67	159
JAM	2008	country	2_Amr	Jamaica	698	274	972	0.0136984	0.0053773	0.0190757	50955	91	75	166
JAM	2009	country	2_Amr	Jamaica	676	265	941	0.0134107	0.0052571	0.0186678	50408	84	64	148
JAM	2010	country	2_Amr	Jamaica	652	258	910	0.0130569	0.0051667	0.0182236	49935	89	70	159
JAM	2011	country	2_Amr	Jamaica	627	251	878	0.0126885	0.0050795	0.017768	49415	86	81	167
JAM	2012	country	2_Amr	Jamaica	604	242	846	0.0123299	0.0049401	0.01727	48987	64	74	138
JAM	2013	country	2_Amr	Jamaica	582	232	814	0.0119532	0.0047649	0.0167181	48690	68	67	136
JAM	2014	country	2_Amr	Jamaica	561	223	784	0.0116034	0.0046124	0.0162158	48348	68	63	131
JAM	2015	country	2_Amr	Jamaica	539	214	753	0.0112109	0.0044511	0.015662	48078	62	61	123
JAM	2016	country	2_Amr	Jamaica	520	208	728	0.0108776	0.0043511	0.0152287	47805	61	60	121
JAM	2017	country	2_Amr	Jamaica	500	202	702	0.0105081	0.0042453	0.0147533	47583	59	61	120
JAM	2018	country	2_Amr	Jamaica	481	196	677	0.0101774	0.0041471	0.0143245	47262	56	57	114
JAM	2019	country	2_Amr	Jamaica	461	191	652	0.0098393	0.0040766	0.0139159	46853	54	56	110

iso3	year	level	whoreg6	whoname	nnd	pnd	u5d	nmr	pnmr	u5mr	Livebirths	Neonatal birth defects deaths	1-59 month birth defects deaths	Under five birth defects deaths
JOR	2000	country	5_Emr	Jordan	2622	1672	4294	0.0161187	0.0102816	0.0264003	162669	481	457	938
JOR	2001	country	5_Emr	Jordan	2598	1647	4245	0.0157193	0.0099636	0.0256829	165275	482	478	960
JOR	2002	country	5_Emr	Jordan	2572	1628	4200	0.0152853	0.0096724	0.0249577	168267	483	490	973
JOR	2003	country	5_Emr	Jordan	2551	1614	4165	0.0148444	0.0093929	0.0242373	171849	485	500	984
JOR	2004	country	5_Emr	Jordan	2532	1606	4138	0.0143931	0.0091276	0.0235206	175918	490	504	994
JOR	2005	country	5_Emr	Jordan	2517	1604	4121	0.0139284	0.0088763	0.0228047	180710	492	510	1003
JOR	2006	country	5_Emr	Jordan	2511	1605	4116	0.0134861	0.0086199	0.022106	186192	500	518	1017
JOR	2007	country	5_Emr	Jordan	2510	1611	4121	0.0130662	0.0083842	0.0214504	192098	502	525	1027
JOR	2008	country	5_Emr	Jordan	2514	1618	4132	0.0126764	0.0081569	0.0208333	198321	514	533	1047
JOR	2009	country	5_Emr	Jordan	2518	1623	4141	0.0123164	0.0079385	0.0202549	204443	519	540	1059
JOR	2010	country	5_Emr	Jordan	2513	1633	4146	0.011965	0.0077743	0.0197393	210029	523	547	1070
JOR	2011	country	5_Emr	Jordan	2496	1639	4135	0.0116336	0.0076387	0.0192724	214551	515	554	1069
JOR	2012	country	5_Emr	Jordan	2464	1637	4101	0.0113062	0.0075119	0.018818	217934	511	557	1069
JOR	2013	country	5_Emr	Jordan	2415	1635	4050	0.0109817	0.0074343	0.018416	219911	506	559	1065
JOR	2014	country	5_Emr	Jordan	2353	1611	3964	0.010669	0.0073046	0.0179736	220546	491	558	1049
JOR	2015	country	5_Emr	Jordan	2279	1584	3863	0.010356	0.0071978	0.0175538	220067	481	553	1034
JOR	2016	country	5_Emr	Jordan	2197	1544	3741	0.0100403	0.0070561	0.0170965	218817	480	543	1023
JOR	2017	country	5_Emr	Jordan	2120	1490	3610	0.0097571	0.0068566	0.0166137	217278	461	528	989
JOR	2018	country	5_Emr	Jordan	2045	1436	3481	0.0094718	0.0066491	0.016121	215903	448	513	960
JOR	2019	country	5_Emr	Jordan	1974	1384	3358	0.0091915	0.006443	0.0156345	214763	432	497	929
JPN	2000	country	6_Wpr	Japan	2061	3234	5295	0.0017676	0.0027736	0.0045413	1165976	975	1135	2111
JPN	2001	country	6_Wpr	Japan	1987	3030	5017	0.0017199	0.0026227	0.0043425	1155315	936	1102	2038
JPN	2002	country	6_Wpr	Japan	1903	2872	4775	0.0016611	0.002507	0.0041681	1145613	1003	1014	2017
JPN	2003	country	6_Wpr	Japan	1804	2767	4571	0.0015862	0.002433	0.0040192	1137281	837	979	1816
JPN	2004	country	6_Wpr	Japan	1700	2695	4395	0.0015038	0.002384	0.0038878	1130447	834	970	1804
JPN	2005	country	6_Wpr	Japan	1601	2636	4237	0.0014234	0.0023436	0.003767	1124771	729	892	1621
JPN	2006	country	6_Wpr	Japan	1511	2579	4090	0.0013478	0.0023004	0.0036482	1121089	676	881	1557
JPN	2007	country	6_Wpr	Japan	1429	2520	3949	0.0012788	0.0022551	0.003534	1117445	648	921	1569
JPN	2008	country	6_Wpr	Japan	1354	2458	3812	0.0012155	0.0022065	0.003422	1113957	612	895	1507
JPN	2009	country	6_Wpr	Japan	1282	2396	3678	0.0011547	0.0021581	0.0033129	1110221	580	902	1482
JPN	2010	country	6_Wpr	Japan	1211	2336	3547	0.0010975	0.0021171	0.0032146	1103396	575	883	1458
JPN	2011	country	6_Wpr	Japan	1145	2389	3534	0.0010467	0.0021838	0.0032305	1093955	516	746	1261
JPN	2012	country	6_Wpr	Japan	1083	2200	3283	0.0010029	0.0020374	0.0030403	1079820	481	848	1329
JPN	2013	country	6_Wpr	Japan	1033	2113	3146	0.0009719	0.001988	0.0029599	1062866	496	804	1300
JPN	2014	country	6_Wpr	Japan	986	2020	3006	0.0009457	0.0019375	0.0028832	1042577	465	777	1242
JPN	2015	country	6_Wpr	Japan	940	1926	2866	0.0009226	0.0018903	0.0028129	1018887	445	743	1188
JPN	2016	country	6_Wpr	Japan	894	1832	2726	0.0008993	0.0018428	0.0027421	994142	388	726	1114
JPN	2017	country	6_Wpr	Japan	853	1738	2591	0.0008792	0.0017913	0.0026705	970241	392	676	1068
JPN	2018	country	6_Wpr	Japan	816	1648	2464	0.000861	0.0017389	0.0025999	947735	372	641	1013
JPN	2019	country	6_Wpr	Japan	782	1561	2343	0.0008435	0.0016837	0.0025272	927108	352	607	959

iso3	year	level	whoreg6	whoname	nnd	pnd	u5d	nmr	pnmr	u5mr	Livebirths	Neonatal birth defects deaths	1-59 month birth defects deaths	Under five birth defects deaths
KAZ	2000	country	4_Eur	Kazakhstan	5372	4678	10050	0.0227275	0.0197914	0.0425189	236366	609	829	1439
KAZ	2001	country	4_Eur	Kazakhstan	5286	4151	9437	0.0220503	0.0173157	0.039366	239724	595	700	1295
KAZ	2002	country	4_Eur	Kazakhstan	5300	3743	9043	0.0213818	0.0151004	0.0364821	247875	600	648	1248
KAZ	2003	country	4_Eur	Kazakhstan	5387	3438	8825	0.0207155	0.0132207	0.0339362	260047	626	658	1284
KAZ	2004	country	4_Eur	Kazakhstan	5520	3204	8724	0.0200474	0.0116362	0.0316836	275348	643	616	1259
KAZ	2005	country	4_Eur	Kazakhstan	5644	3021	8665	0.0192905	0.0103254	0.029616	292579	709	583	1292
KAZ	2006	country	4_Eur	Kazakhstan	5686	2892	8578	0.0183109	0.0093132	0.0276241	310526	754	562	1316
KAZ	2007	country	4_Eur	Kazakhstan	5540	2874	8414	0.0168908	0.0087625	0.0256532	327990	812	573	1385
KAZ	2008	country	4_Eur	Kazakhstan	5259	2889	8148	0.0152965	0.008403	0.0236995	343805	860	604	1465
KAZ	2009	country	4_Eur	Kazakhstan	4845	2931	7776	0.0135624	0.0082046	0.021767	357238	885	654	1539
KAZ	2010	country	4_Eur	Kazakhstan	4343	2957	7300	0.0118041	0.008037	0.0198411	367924	875	694	1570
KAZ	2011	country	4_Eur	Kazakhstan	3806	2935	6741	0.0101192	0.0078034	0.0179226	376116	865	724	1589
KAZ	2012	country	4_Eur	Kazakhstan	3291	2854	6145	0.0086101	0.0074668	0.0160768	382227	791	739	1530
KAZ	2013	country	4_Eur	Kazakhstan	2835	2729	5564	0.0073241	0.0070503	0.0143744	387078	686	739	1426
KAZ	2014	country	4_Eur	Kazakhstan	2460	2577	5037	0.0063011	0.0066008	0.0129019	390408	622	727	1348
KAZ	2015	country	4_Eur	Kazakhstan	2177	2427	4604	0.0055512	0.0061887	0.0117399	392166	548	707	1255
KAZ	2016	country	4_Eur	Kazakhstan	1989	2297	4286	0.0050781	0.0058644	0.0109425	391682	512	685	1197
KAZ	2017	country	4_Eur	Kazakhstan	1873	2210	4083	0.0048166	0.0056833	0.0104999	388862	491	674	1164
KAZ	2018	country	4_Eur	Kazakhstan	1802	2185	3987	0.0046958	0.0056938	0.0103895	383751	475	677	1152
KAZ	2019	country	4_Eur	Kazakhstan	1757	2219	3976	0.0046656	0.0058924	0.010558	376587	464	696	1160
KEN	2000	country	1_Afr	Kenya	36541	85218	121759	0.0285349	0.0665465	0.0950814	1280572	1953	1460	3412
KEN	2001	country	1_Afr	Kenya	37125	81948	119073	0.0283843	0.0626544	0.0910387	1307943	2052	1388	3440
KEN	2002	country	1_Afr	Kenya	37558	78133	115691	0.0281459	0.058553	0.0866989	1334404	2058	1553	3611
KEN	2003	country	1_Afr	Kenya	37893	73848	111741	0.0278538	0.0542831	0.0821369	1360425	2066	1456	3522
KEN	2004	country	1_Afr	Kenya	38103	70277	108380	0.0274983	0.050718	0.0782164	1385649	2103	1673	3776
KEN	2005	country	1_Afr	Kenya	38145	66212	104357	0.0270668	0.0469825	0.0740493	1409292	2104	2545	4649
KEN	2006	country	1_Afr	Kenya	37975	62524	100499	0.0265496	0.0437127	0.0702622	1430344	2125	3269	5394
KEN	2007	country	1_Afr	Kenya	37661	58900	96561	0.0260092	0.0406769	0.066686	1447989	2167	2438	4605
KEN	2008	country	1_Afr	Kenya	37203	54302	91505	0.0254516	0.0371495	0.0626011	1461717	2186	3136	5322
KEN	2009	country	1_Afr	Kenya	36701	50609	87310	0.0249411	0.0343926	0.0593337	1471508	2152	3027	5179
KEN	2010	country	1_Afr	Kenya	36195	48137	84332	0.024503	0.0325871	0.0570902	1477164	2122	2876	4998
KEN	2011	country	1_Afr	Kenya	35652	46530	82182	0.0241082	0.0314638	0.055572	1478832	2144	3176	5320
KEN	2012	country	1_Afr	Kenya	35070	45239	80309	0.023737	0.0306201	0.0543571	1477439	2077	3601	5678
KEN	2013	country	1_Afr	Kenya	34536	42968	77504	0.0234252	0.0291443	0.0525695	1474312	2122	3600	5722
KEN	2014	country	1_Afr	Kenya	33919	41323	75242	0.0230636	0.0280977	0.0511613	1470675	2122	3632	5754
KEN	2015	country	1_Afr	Kenya	33339	39346	72685	0.0227091	0.0268005	0.0495095	1468092	2098	3591	5689
KEN	2016	country	1_Afr	Kenya	32755	37217	69972	0.0223113	0.0253504	0.0476618	1468087	2120	3388	5508
KEN	2017	country	1_Afr	Kenya	32272	35591	67863	0.0219312	0.0241867	0.0461179	1471512	2095	2711	4806
KEN	2018	country	1_Afr	Kenya	31765	33661	65426	0.0214807	0.0227632	0.0442439	1478766	2059	2674	4732
KEN	2019	country	1_Afr	Kenya	31343	32314	63657	0.0210365	0.0216885	0.042725	1489933	2078	2605	4682

iso3	year	level	whoreg6	whoname	nnd	pnd	u5d	nmr	pnmr	u5mr	Livebirths	Neonatal birth defects deaths	1-59 month birth defects deaths	Under five birth defects deaths
KGZ	2000	country	4_Eur	Kyrgyzstan	2166	3161	5327	0.0205229	0.0299505	0.0504734	105541	249	150	398
KGZ	2001	country	4_Eur	Kyrgyzstan	2093	2878	4971	0.0201331	0.0276842	0.0478173	103958	236	200	436
KGZ	2002	country	4_Eur	Kyrgyzstan	2061	2621	4682	0.0198995	0.0253064	0.0452059	103571	237	199	437
KGZ	2003	country	4_Eur	Kyrgyzstan	2061	2405	4466	0.019739	0.0230336	0.0427726	104413	236	182	418
KGZ	2004	country	4_Eur	Kyrgyzstan	2086	2226	4312	0.0195609	0.0208737	0.0404346	106641	241	254	496
KGZ	2005	country	4_Eur	Kyrgyzstan	2133	2089	4222	0.0193283	0.0189296	0.038258	110356	253	195	448
KGZ	2006	country	4_Eur	Kyrgyzstan	2207	1985	4192	0.0190704	0.0171521	0.0362225	115729	269	195	464
KGZ	2007	country	4_Eur	Kyrgyzstan	2300	1896	4196	0.0187877	0.0154876	0.0342754	122420	283	206	490
KGZ	2008	country	4_Eur	Kyrgyzstan	2386	1806	4192	0.0183464	0.0138867	0.0322331	130053	303	200	503
KGZ	2009	country	4_Eur	Kyrgyzstan	2454	1711	4165	0.0177732	0.012392	0.0301652	138073	317	202	519
KGZ	2010	country	4_Eur	Kyrgyzstan	2505	1614	4119	0.0171814	0.0110701	0.0282515	145798	342	213	555
KGZ	2011	country	4_Eur	Kyrgyzstan	2519	1543	4062	0.0165136	0.0101153	0.0266288	152541	360	218	578
KGZ	2012	country	4_Eur	Kyrgyzstan	2518	1472	3990	0.015964	0.0093324	0.0252964	157730	370	191	562
KGZ	2013	country	4_Eur	Kyrgyzstan	2509	1382	3891	0.0155679	0.0085751	0.024143	161165	390	221	611
KGZ	2014	country	4_Eur	Kyrgyzstan	2466	1297	3763	0.0151634	0.0079752	0.0231387	162628	390	237	627
KGZ	2015	country	4_Eur	Kyrgyzstan	2384	1218	3602	0.0146907	0.0075056	0.0221962	162280	395	268	663
KGZ	2016	country	4_Eur	Kyrgyzstan	2265	1145	3410	0.0141052	0.0071305	0.0212357	160579	392	254	646
KGZ	2017	country	4_Eur	Kyrgyzstan	2125	1077	3202	0.0134459	0.0068147	0.0202605	158041	383	224	607
KGZ	2018	country	4_Eur	Kyrgyzstan	1995	1007	3002	0.012843	0.0064826	0.0193256	155338	375	210	585
KGZ	2019	country	4_Eur	Kyrgyzstan	1880	944	2824	0.0123037	0.006178	0.0184817	152800	357	197	554
KHM	2000	country	6_Wpr	Cambodia	11980	24982	36962	0.0352513	0.0735098	0.1087611	339845	735	291	1026
KHM	2001	country	6_Wpr	Cambodia	11291	21737	33028	0.0333964	0.0642947	0.0976911	338090	709	269	978
KHM	2002	country	6_Wpr	Cambodia	10649	18360	29009	0.0314977	0.0543044	0.0858021	338088	704	235	939
KHM	2003	country	6_Wpr	Cambodia	10108	15873	25981	0.0297418	0.0467054	0.0764473	339858	708	329	1037
KHM	2004	country	6_Wpr	Cambodia	9660	14069	23729	0.0281492	0.0409967	0.0691459	343172	705	481	1186
KHM	2005	country	6_Wpr	Cambodia	9319	12690	22009	0.0268121	0.0365121	0.0633242	347567	701	475	1175
KHM	2006	country	6_Wpr	Cambodia	9023	11634	20657	0.0256003	0.0330081	0.0586084	352457	705	461	1166
KHM	2007	country	6_Wpr	Cambodia	8746	10745	19491	0.0244885	0.0300846	0.0545731	357147	709	479	1187
KHM	2008	country	6_Wpr	Cambodia	8440	10230	18670	0.023377	0.0283353	0.0517124	361038	706	602	1308
KHM	2009	country	6_Wpr	Cambodia	8101	9545	17646	0.0222605	0.0262284	0.0484889	363918	690	632	1321
KHM	2010	country	6_Wpr	Cambodia	7748	8352	16100	0.0211871	0.0228387	0.0440258	365693	680	603	1284
KHM	2011	country	6_Wpr	Cambodia	7377	7499	14876	0.0201237	0.0204563	0.0405801	366582	674	561	1235
KHM	2012	country	6_Wpr	Cambodia	7025	6712	13737	0.0191416	0.0182876	0.0374292	367002	694	501	1194
KHM	2013	country	6_Wpr	Cambodia	6708	6175	12883	0.0182644	0.0168123	0.0350768	367271	734	432	1166
KHM	2014	country	6_Wpr	Cambodia	6441	5723	12164	0.0175279	0.015574	0.0331019	367471	765	434	1199
KHM	2015	country	6_Wpr	Cambodia	6199	5349	11548	0.0168709	0.0145578	0.0314287	367437	779	432	1211
KHM	2016	country	6_Wpr	Cambodia	5987	5013	11000	0.0163091	0.0136567	0.0299659	367095	804	417	1221
KHM	2017	country	6_Wpr	Cambodia	5765	4766	10531	0.0157393	0.0130111	0.0287504	366281	829	398	1227
KHM	2018	country	6_Wpr	Cambodia	5506	4561	10067	0.0150923	0.0125015	0.0275939	364821	821	405	1226
KHM	2019	country	6_Wpr	Cambodia	5257	4426	9683	0.0144907	0.0121988	0.0266895	362783	826	416	1242

iso3	year	level	whoreg6	whoname	nnd	pnd	u5d	nmr	pnmr	u5mr	Livebirths	Neonatal birth defects deaths	1-59 month birth defects deaths	Under five birth defects deaths
KIR	2000	country	6_Wpr	Kiribati	74	108	182	0.0287168	0.041911	0.0706278	2577	7	6	13
KIR	2001	country	6_Wpr	Kiribati	73	105	178	0.0280427	0.0403354	0.0683781	2603	7	6	12
KIR	2002	country	6_Wpr	Kiribati	72	103	175	0.0274822	0.0393148	0.0667969	2620	7	8	14
KIR	2003	country	6_Wpr	Kiribati	72	101	173	0.0270371	0.037927	0.0649641	2663	6	5	12
KIR	2004	country	6_Wpr	Kiribati	72	102	174	0.0266557	0.0377622	0.0644179	2701	6	4	10
KIR	2005	country	6_Wpr	Kiribati	72	104	176	0.0264571	0.0382159	0.064673	2721	7	7	14
KIR	2006	country	6_Wpr	Kiribati	73	107	180	0.0262788	0.0385182	0.064797	2778	7	4	11
KIR	2007	country	6_Wpr	Kiribati	75	108	183	0.0261763	0.0376939	0.0638702	2865	7	9	16
KIR	2008	country	6_Wpr	Kiribati	76	111	187	0.0260487	0.0380448	0.0640936	2918	7	6	13
KIR	2009	country	6_Wpr	Kiribati	77	112	189	0.0258996	0.0376722	0.0635719	2973	7	8	15
KIR	2010	country	6_Wpr	Kiribati	78	112	190	0.0256673	0.0368556	0.062523	3039	8	10	17
KIR	2011	country	6_Wpr	Kiribati	78	112	190	0.0253938	0.0364628	0.0618566	3072	8	10	18
KIR	2012	country	6_Wpr	Kiribati	78	111	189	0.025089	0.0357036	0.0607927	3109	8	10	18
KIR	2013	country	6_Wpr	Kiribati	78	110	188	0.0246664	0.0347859	0.0594523	3162	8	11	19
KIR	2014	country	6_Wpr	Kiribati	78	107	185	0.0243139	0.0333537	0.0576677	3208	8	10	18
KIR	2015	country	6_Wpr	Kiribati	77	105	182	0.0238323	0.0324986	0.0563308	3231	8	9	18
KIR	2016	country	6_Wpr	Kiribati	76	102	178	0.0233902	0.0313922	0.0547824	3249	8	9	17
KIR	2017	country	6_Wpr	Kiribati	74	99	173	0.0230206	0.0307978	0.0538184	3215	8	9	17
KIR	2018	country	6_Wpr	Kiribati	73	96	169	0.0225589	0.0296665	0.0522254	3236	8	9	17
KIR	2019	country	6_Wpr	Kiribati	72	92	164	0.022115	0.0282581	0.0503731	3256	8	9	17
KNA	2000	country	2_Amr	Saint Kitts and Nevi	13	6	19	0.0165889	0.0076564	0.0242453	784	1	2	3
KNA	2001	country	2_Amr	Saint Kitts and Nevi	12	6	18	0.0159488	0.0079744	0.0239232	752	0	1	1
KNA	2002	country	2_Amr	Saint Kitts and Nevi	11	5	16	0.0152475	0.0069307	0.0221782	721	0	1	1
KNA	2003	country	2_Amr	Saint Kitts and Nevi	11	5	16	0.0145263	0.0066029	0.0211292	757	1	0	1
KNA	2004	country	2_Amr	Saint Kitts and Nevi	10	5	15	0.013867	0.0069335	0.0208005	721	1	0	1
KNA	2005	country	2_Amr	Saint Kitts and Nevi	10	4	14	0.0132626	0.005305	0.0185677	754	3	2	5
KNA	2006	country	2_Amr	Saint Kitts and Nevi	9	5	14	0.0127761	0.0070978	0.0198739	704	0	1	1
KNA	2007	country	2_Amr	Saint Kitts and Nevi	9	4	13	0.0123861	0.0055049	0.017891	727	1	1	2
KNA	2008	country	2_Amr	Saint Kitts and Nevi	9	4	13	0.0121008	0.0053781	0.0174789	744	1	0	1
KNA	2009	country	2_Amr	Saint Kitts and Nevi	9	4	13	0.0118946	0.0052865	0.0171811	757	1	0	1
KNA	2010	country	2_Amr	Saint Kitts and Nevi	9	4	13	0.0117339	0.005215	0.0169489	767	0	0	0
KNA	2011	country	2_Amr	Saint Kitts and Nevi	8	5	13	0.0116322	0.0072702	0.0189024	688	0	1	1
KNA	2012	country	2_Amr	Saint Kitts and Nevi	8	5	13	0.0115621	0.0072263	0.0187884	692	3	1	4
KNA	2013	country	2_Amr	Saint Kitts and Nevi	8	5	13	0.0115138	0.0071961	0.01871	695	0	0	0
KNA	2014	country	2_Amr	Saint Kitts and Nevi	8	4	12	0.0114397	0.0057199	0.0171596	699	1	0	1
KNA	2015	country	2_Amr	Saint Kitts and Nevi	8	4	12	0.0112985	0.0056492	0.0169477	708	2	1	3
KNA	2016	country	2_Amr	Saint Kitts and Nevi	8	4	12	0.0110996	0.0055498	0.0166494	721	2	0	2
KNA	2017	country	2_Amr	Saint Kitts and Nevi	8	3	11	0.0108577	0.0040717	0.0149294	737	1	0	2
KNA	2018	country	2_Amr	Saint Kitts and Nevi	7	4	11	0.0105728	0.0060416	0.0166145	662	2	0	2
KNA	2019	country	2_Amr	Saint Kitts and Nevi	7	4	11	0.0102799	0.0058743	0.0161542	681	1	0	2

iso3	year	level	whoreg6	whoname	nnd	pnd	u5d	nmr	pnmr	u5mr	Livebirths	Neonatal birth defects deaths	1-59 month birth defects deaths	Under five birth defects deaths
KOR	2000	country	6_Wpr	Republic of Korea	1938	2510	4448	0.0033992	0.0044025	0.0078016	570136	537	528	1065
KOR	2001	country	6_Wpr	Republic of Korea	1737	2298	4035	0.0031912	0.0042219	0.0074131	544310	421	471	892
KOR	2002	country	6_Wpr	Republic of Korea	1561	2095	3656	0.0029984	0.0040242	0.0070226	520603	419	489	908
KOR	2003	country	6_Wpr	Republic of Korea	1406	1899	3305	0.0028099	0.0037951	0.006605	500376	342	453	794
KOR	2004	country	6_Wpr	Republic of Korea	1264	1720	2984	0.0026125	0.0035549	0.0061674	483837	285	387	672
KOR	2005	country	6_Wpr	Republic of Korea	1129	1565	2694	0.0023919	0.0033156	0.0057075	472009	230	345	575
KOR	2006	country	6_Wpr	Republic of Korea	1011	1435	2446	0.0021767	0.0030895	0.0052662	464475	227	308	534
KOR	2007	country	6_Wpr	Republic of Korea	921	1325	2246	0.0020019	0.00288	0.0048819	460063	203	313	516
KOR	2008	country	6_Wpr	Republic of Korea	860	1232	2092	0.0018795	0.0026925	0.004572	457565	151	301	451
KOR	2009	country	6_Wpr	Republic of Korea	822	1151	1973	0.0018012	0.0025221	0.0043232	456373	155	276	431
KOR	2010	country	6_Wpr	Republic of Korea	798	1082	1880	0.0017538	0.002378	0.0041319	455000	139	276	415
KOR	2011	country	6_Wpr	Republic of Korea	782	1021	1803	0.0017302	0.0022589	0.0039891	451982	158	239	397
KOR	2012	country	6_Wpr	Republic of Korea	763	966	1729	0.0017092	0.002164	0.0038732	446405	144	241	385
KOR	2013	country	6_Wpr	Republic of Korea	739	913	1652	0.0016845	0.0020811	0.0037656	438709	162	237	398
KOR	2014	country	6_Wpr	Republic of Korea	710	862	1572	0.0016569	0.0020117	0.0036686	428499	151	259	410
KOR	2015	country	6_Wpr	Republic of Korea	678	812	1490	0.0016291	0.001951	0.0035801	416189	132	237	369
KOR	2016	country	6_Wpr	Republic of Korea	645	765	1410	0.0016001	0.0018978	0.0034978	403107	138	198	336
KOR	2017	country	6_Wpr	Republic of Korea	614	719	1333	0.0015768	0.0018464	0.0034232	389403	127	204	331
KOR	2018	country	6_Wpr	Republic of Korea	588	675	1263	0.0015575	0.001788	0.0033455	377520	121	192	312
KOR	2019	country	6_Wpr	Republic of Korea	565	635	1200	0.0015375	0.001728	0.0032654	367485	118	180	298
KWT	2000	country	5_Emr	Kuwait	290	250	540	0.0065465	0.0056436	0.0121901	44298	147	107	254
KWT	2001	country	5_Emr	Kuwait	285	251	536	0.0063626	0.0056036	0.0119662	44793	131	112	243
KWT	2002	country	5_Emr	Kuwait	282	254	536	0.0061998	0.0055843	0.0117841	45485	130	106	236
KWT	2003	country	5_Emr	Kuwait	282	257	539	0.0060579	0.0055209	0.0115788	46550	140	110	250
KWT	2004	country	5_Emr	Kuwait	285	259	544	0.0059301	0.0053891	0.0113193	48060	143	114	256
KWT	2005	country	5_Emr	Kuwait	290	262	552	0.0058293	0.0052665	0.0110958	49749	140	115	255
KWT	2006	country	5_Emr	Kuwait	298	266	564	0.005767	0.0051477	0.0109147	51673	144	131	275
KWT	2007	country	5_Emr	Kuwait	308	271	579	0.0057308	0.0050423	0.0107731	53745	134	115	248
KWT	2008	country	5_Emr	Kuwait	317	274	591	0.0056869	0.0049155	0.0106024	55742	157	127	284
KWT	2009	country	5_Emr	Kuwait	322	275	597	0.005602	0.0047844	0.0103864	57479	158	107	265
KWT	2010	country	5_Emr	Kuwait	322	274	596	0.0054836	0.0046662	0.0101499	58720	167	101	268
KWT	2011	country	5_Emr	Kuwait	320	269	589	0.0053504	0.0044977	0.0098481	59809	153	89	242
KWT	2012	country	5_Emr	Kuwait	316	259	575	0.0052234	0.0042812	0.0095045	60497	146	91	237
KWT	2013	country	5_Emr	Kuwait	311	248	559	0.0051052	0.0040711	0.0091763	60918	125	91	216
KWT	2014	country	5_Emr	Kuwait	305	238	543	0.0050043	0.003905	0.0089093	60947	127	78	204
KWT	2015	country	5_Emr	Kuwait	298	230	528	0.0049145	0.0037931	0.0087077	60636	127	80	207
KWT	2016	country	5_Emr	Kuwait	289	222	511	0.0048266	0.0037077	0.0085343	59876	120	77	197
KWT	2017	country	5_Emr	Kuwait	278	214	492	0.004732	0.0036426	0.0083746	58749	116	75	191
KWT	2018	country	5_Emr	Kuwait	265	206	471	0.0046285	0.003598	0.0082264	57254	111	72	183
KWT	2019	country	5_Emr	Kuwait	251	197	448	0.0045186	0.0035465	0.0080651	55548	105	69	173

iso3	year	level	whoreg6	whoname	nnd	pnd	u5d	nmr	pnmr	u5mr	Livebirths	Neonatal birth defects deaths	1-59 month birth defects deaths	Under five birth defects deaths
LAO	2000	country	6_Wpr	Lao People's Demo	6470	12008	18478	0.0380171	0.070557	0.108574	170187	292	81	373
LAO	2001	country	6_Wpr	Lao People's Demo	6235	11230	17465	0.0370835	0.0667941	0.1038777	168134	289	105	394
LAO	2002	country	6_Wpr	Lao People's Demo	6032	10494	16526	0.0361673	0.062923	0.0990903	166780	288	124	412
LAO	2003	country	6_Wpr	Lao People's Demo	5854	10416	16270	0.0352462	0.0627127	0.0979589	166089	287	92	380
LAO	2004	country	6_Wpr	Lao People's Demo	5697	10299	15996	0.034322	0.0620489	0.0963709	165987	297	82	379
LAO	2005	country	6_Wpr	Lao People's Demo	5553	9698	15251	0.0333835	0.0583044	0.0916879	166340	287	96	383
LAO	2006	country	6_Wpr	Lao People's Demo	5410	9098	14508	0.0324108	0.0545054	0.0869162	166920	295	116	411
LAO	2007	country	6_Wpr	Lao People's Demo	5271	9101	14372	0.0314663	0.0543287	0.085795	167512	298	97	395
LAO	2008	country	6_Wpr	Lao People's Demo	5125	7192	12317	0.0305304	0.0428467	0.0733771	167866	302	134	436
LAO	2009	country	6_Wpr	Lao People's Demo	4968	6841	11809	0.0295803	0.0407315	0.0703117	167950	310	164	475
LAO	2010	country	6_Wpr	Lao People's Demo	4802	6513	11315	0.0286228	0.0388225	0.0674453	167768	311	191	503
LAO	2011	country	6_Wpr	Lao People's Demo	4640	6198	10838	0.0277182	0.0370269	0.0647451	167399	321	220	541
LAO	2012	country	6_Wpr	Lao People's Demo	4491	5734	10225	0.0268773	0.0343174	0.0611946	167093	326	235	561
LAO	2013	country	6_Wpr	Lao People's Demo	4342	5410	9752	0.0260148	0.0324144	0.0584292	166905	327	295	622
LAO	2014	country	6_Wpr	Lao People's Demo	4204	5105	9309	0.0251967	0.0305983	0.055795	166847	334	350	683
LAO	2015	country	6_Wpr	Lao People's Demo	4079	4798	8877	0.0244401	0.0287455	0.0531856	166898	340	316	655
LAO	2016	country	6_Wpr	Lao People's Demo	3962	4545	8507	0.0237444	0.0272412	0.0509856	166860	336	212	548
LAO	2017	country	6_Wpr	Lao People's Demo	3850	4320	8170	0.0230947	0.0259126	0.0490073	166705	341	237	579
LAO	2018	country	6_Wpr	Lao People's Demo	3745	4085	7830	0.0225159	0.0245583	0.0470742	166327	343	216	559
LAO	2019	country	6_Wpr	Lao People's Demo	3637	3901	7538	0.0219562	0.0235472	0.0455034	165648	337	211	548
LBN	2000	country	5_Emr	Lebanon	980	692	1672	0.0116914	0.008261	0.0199524	83822	216	233	448
LBN	2001	country	5_Emr	Lebanon	902	645	1547	0.0110043	0.0078689	0.0188733	81968	207	222	429
LBN	2002	country	5_Emr	Lebanon	823	595	1418	0.0103019	0.0074518	0.0177537	79888	199	208	407
LBN	2003	country	5_Emr	Lebanon	750	570	1320	0.009629	0.0073147	0.0169437	77889	188	195	383
LBN	2004	country	5_Emr	Lebanon	681	504	1185	0.008955	0.006621	0.0155761	76047	176	181	357
LBN	2005	country	5_Emr	Lebanon	621	491	1112	0.008285	0.0065446	0.0148295	74955	165	167	332
LBN	2006	country	5_Emr	Lebanon	574	459	1033	0.0076783	0.0061365	0.0138147	74756	153	154	308
LBN	2007	country	5_Emr	Lebanon	540	393	933	0.0071164	0.0051751	0.0122914	75881	146	145	291
LBN	2008	country	5_Emr	Lebanon	520	350	870	0.006656	0.0044743	0.0111303	78125	141	138	280
LBN	2009	country	5_Emr	Lebanon	514	335	849	0.0062959	0.004098	0.0103939	81641	142	134	276
LBN	2010	country	5_Emr	Lebanon	514	329	843	0.0059748	0.0038196	0.0097944	86028	142	133	275
LBN	2011	country	5_Emr	Lebanon	520	329	849	0.0056929	0.0036008	0.0092937	91341	146	135	281
LBN	2012	country	5_Emr	Lebanon	528	332	860	0.0054501	0.0034309	0.008881	96878	147	137	284
LBN	2013	country	5_Emr	Lebanon	534	443	977	0.0052197	0.0043312	0.0095509	102305	147	140	287
LBN	2014	country	5_Emr	Lebanon	536	349	885	0.0050034	0.0032537	0.0082571	107126	150	144	295
LBN	2015	country	5_Emr	Lebanon	535	342	877	0.0048104	0.0030727	0.0078831	111218	149	145	294
LBN	2016	country	5_Emr	Lebanon	529	347	876	0.0046315	0.0030389	0.0076703	114219	148	147	295
LBN	2017	country	5_Emr	Lebanon	519	351	870	0.0044673	0.0030233	0.0074906	116177	146	147	293
LBN	2018	country	5_Emr	Lebanon	505	398	903	0.0043122	0.0033963	0.0077085	117110	139	147	286
LBN	2019	country	5_Emr	Lebanon	485	402	887	0.004152	0.0034433	0.0075953	116811	136	146	282

iso3	year	level	whoreg6	whoname	nnd	pnd	u5d	nmr	pnmr	u5mr	Livebirths	Neonatal birth defects deaths	1-59 month birth defects deaths	Under five birth defects deaths
LBR	2000	country	1_Afr	Liberia	5343	18401	23744	0.0457163	0.1574471	0.2031634	116873	217	16	233
LBR	2001	country	1_Afr	Liberia	5274	16733	22007	0.0437245	0.1387228	0.1824472	120619	220	17	237
LBR	2002	country	1_Afr	Liberia	5186	13853	19039	0.0418063	0.1116761	0.1534824	124048	228	18	247
LBR	2003	country	1_Afr	Liberia	5092	12863	17955	0.040057	0.1011919	0.1412489	127119	227	21	247
LBR	2004	country	1_Afr	Liberia	5008	11908	16916	0.0385633	0.0916944	0.1302577	129864	232	23	254
LBR	2005	country	1_Afr	Liberia	4930	10637	15567	0.0372682	0.0804074	0.1176756	132284	233	48	281
LBR	2006	country	1_Afr	Liberia	4853	10178	15031	0.0360895	0.0756862	0.1117757	134471	242	57	299
LBR	2007	country	1_Afr	Liberia	4784	9771	14555	0.0350248	0.0715322	0.106557	136589	243	77	320
LBR	2008	country	1_Afr	Liberia	4733	9189	13922	0.0341263	0.0662547	0.100381	138691	249	94	343
LBR	2009	country	1_Afr	Liberia	4711	8972	13683	0.0334416	0.0636869	0.0971285	140873	255	124	379
LBR	2010	country	1_Afr	Liberia	4714	10020	14734	0.0329495	0.0700348	0.1029843	143067	264	89	353
LBR	2011	country	1_Afr	Liberia	4755	9300	14055	0.0327283	0.0640108	0.0967391	145287	258	110	368
LBR	2012	country	1_Afr	Liberia	4821	8473	13294	0.032696	0.0574633	0.0901593	147449	268	149	416
LBR	2013	country	1_Afr	Liberia	4899	8416	13315	0.0327677	0.0562926	0.0890603	149507	275	123	399
LBR	2014	country	1_Afr	Liberia	4995	8744	13739	0.0329638	0.0577035	0.0906673	151530	280	79	359
LBR	2015	country	1_Afr	Liberia	5068	10015	15083	0.0330228	0.0652557	0.0982785	153470	282	90	372
LBR	2016	country	1_Afr	Liberia	5138	9283	14421	0.0330658	0.05974	0.0928057	155387	291	112	403
LBR	2017	country	1_Afr	Liberia	5188	9641	14829	0.0329646	0.061257	0.0942216	157381	297	119	416
LBR	2018	country	1_Afr	Liberia	5227	9955	15182	0.0327765	0.0624267	0.0952032	159474	304	166	470
LBR	2019	country	1_Afr	Liberia	5239	9945	15184	0.0324162	0.0615337	0.09395	161617	305	167	473
LBY	2000	country	5_Emr	Libya	1844	1414	3258	0.015869	0.0121685	0.0280375	116201	279	367	645
LBY	2001	country	5_Emr	Libya	1797	1369	3166	0.0154024	0.0117339	0.0271364	116670	285	370	655
LBY	2002	country	5_Emr	Libya	1760	1327	3087	0.0149386	0.0112634	0.026202	117815	290	372	662
LBY	2003	country	5_Emr	Libya	1719	1290	3009	0.0143977	0.0108046	0.0252023	119394	297	382	678
LBY	2004	country	5_Emr	Libya	1671	1249	2920	0.0137527	0.0102795	0.0240322	121504	303	382	685
LBY	2005	country	5_Emr	Libya	1613	1201	2814	0.0130171	0.0096922	0.0227093	123914	307	375	683
LBY	2006	country	5_Emr	Libya	1544	1147	2691	0.0122024	0.0090649	0.0212673	126532	315	367	683
LBY	2007	country	5_Emr	Libya	1468	1088	2556	0.0113678	0.0084252	0.019793	129136	317	361	679
LBY	2008	country	5_Emr	Libya	1393	1032	2425	0.0105926	0.0078475	0.0184401	131507	321	353	674
LBY	2009	country	5_Emr	Libya	1326	986	2312	0.009928	0.0073824	0.0173104	133561	326	344	670
LBY	2010	country	5_Emr	Libya	1266	949	2215	0.0093745	0.0070271	0.0164016	135048	317	336	654
LBY	2011	country	5_Emr	Libya	1213	1145	2358	0.0089263	0.0084259	0.0173523	135890	297	329	626
LBY	2012	country	5_Emr	Libya	1153	886	2039	0.0084812	0.0065172	0.0149984	135948	292	321	613
LBY	2013	country	5_Emr	Libya	1097	854	1951	0.008106	0.0063104	0.0144164	135332	275	313	588
LBY	2014	country	5_Emr	Libya	1045	815	1860	0.0077934	0.0060781	0.0138715	134088	262	302	564
LBY	2015	country	5_Emr	Libya	992	783	1775	0.0074922	0.0059137	0.013406	132404	251	294	545
LBY	2016	country	5_Emr	Libya	940	746	1686	0.0072133	0.0057246	0.0129379	130315	238	283	521
LBY	2017	country	5_Emr	Libya	890	713	1603	0.0069464	0.0055649	0.0125113	128125	228	273	501
LBY	2018	country	5_Emr	Libya	845	677	1522	0.0067106	0.0053764	0.012087	125920	223	262	485
LBY	2019	country	5_Emr	Libya	803	645	1448	0.0064749	0.0052009	0.0116757	124018	212	251	463

iso3	year	level	whoreg6	whoname	nnd	pnd	u5d	nmr	pnmr	u5mr	Livebirths	Neonatal birth defects deaths	1-59 month birth defects deaths	Under five birth defects deaths
LCA	2000	country	2_Amr	Saint Lucia	34	22	56	0.0116976	0.007569	0.0192666	2907	6	3	9
LCA	2001	country	2_Amr	Saint Lucia	33	20	53	0.0119651	0.0072516	0.0192167	2758	5	3	8
LCA	2002	country	2_Amr	Saint Lucia	32	19	51	0.0122267	0.0072596	0.0194864	2617	3	0	3
LCA	2003	country	2_Amr	Saint Lucia	31	17	48	0.0120815	0.0066253	0.0187068	2566	6	5	11
LCA	2004	country	2_Amr	Saint Lucia	31	16	47	0.0127024	0.0065561	0.0192585	2440	5	4	9
LCA	2005	country	2_Amr	Saint Lucia	30	15	45	0.0124913	0.0062457	0.018737	2402	6	2	8
LCA	2006	country	2_Amr	Saint Lucia	30	15	45	0.0126742	0.0063371	0.0190113	2367	1	2	3
LCA	2007	country	2_Amr	Saint Lucia	30	14	44	0.0128599	0.0060013	0.0188612	2333	6	3	9
LCA	2008	country	2_Amr	Saint Lucia	30	14	44	0.0130446	0.0060875	0.019132	2300	16	0	16
LCA	2009	country	2_Amr	Saint Lucia	29	15	44	0.0127993	0.0066203	0.0194196	2266	3	7	10
LCA	2010	country	2_Amr	Saint Lucia	29	15	44	0.0129943	0.0067212	0.0197155	2232	4	7	10
LCA	2011	country	2_Amr	Saint Lucia	29	15	44	0.0127693	0.0066048	0.0193741	2271	2	2	4
LCA	2012	country	2_Amr	Saint Lucia	29	16	45	0.0129502	0.007145	0.0200952	2239	3	5	7
LCA	2013	country	2_Amr	Saint Lucia	29	16	45	0.0131147	0.0072357	0.0203504	2211	4	2	6
LCA	2014	country	2_Amr	Saint Lucia	28	18	46	0.012427	0.0079888	0.0204157	2253	6	0	6
LCA	2015	country	2_Amr	Saint Lucia	28	19	47	0.0125464	0.0085136	0.0210601	2232	4	3	7
LCA	2016	country	2_Amr	Saint Lucia	28	19	47	0.012635	0.0085738	0.0212088	2216	5	3	7
LCA	2017	country	2_Amr	Saint Lucia	28	20	48	0.0126989	0.0090707	0.0217696	2205	5	3	8
LCA	2018	country	2_Amr	Saint Lucia	28	20	48	0.0127121	0.0090801	0.0217922	2203	5	3	7
LCA	2019	country	2_Amr	Saint Lucia	28	20	48	0.0130843	0.0093459	0.0224302	2140	5	3	8
LKA	2000	country	3_Sear	Sri Lanka	3329	2385	5714	0.0095715	0.0068574	0.0164289	347802	775	577	1351
LKA	2001	country	3_Sear	Sri Lanka	2927	2639	5566	0.0083434	0.0075225	0.0158659	350815	689	653	1342
LKA	2002	country	3_Sear	Sri Lanka	2537	2913	5450	0.0071656	0.0082276	0.0153932	354052	598	735	1333
LKA	2003	country	3_Sear	Sri Lanka	2293	3056	5349	0.0064169	0.0085521	0.014969	357338	544	785	1329
LKA	2004	country	3_Sear	Sri Lanka	2336	8004	10340	0.0064828	0.0222126	0.0286955	360336	536	781	1317
LKA	2005	country	3_Sear	Sri Lanka	2332	2765	5097	0.0064291	0.0076229	0.014052	362724	559	735	1294
LKA	2006	country	3_Sear	Sri Lanka	2464	2440	4904	0.0067632	0.0066974	0.0134606	364322	614	662	1276
LKA	2007	country	3_Sear	Sri Lanka	2561	2125	4686	0.0070189	0.005824	0.0128429	364871	646	589	1235
LKA	2008	country	3_Sear	Sri Lanka	2555	1941	4496	0.007008	0.0053239	0.0123319	364584	638	548	1186
LKA	2009	country	3_Sear	Sri Lanka	2444	1905	4349	0.0067261	0.0052427	0.0119688	363362	617	546	1163
LKA	2010	country	3_Sear	Sri Lanka	2305	1897	4202	0.0063795	0.0052503	0.0116297	361316	584	559	1143
LKA	2011	country	3_Sear	Sri Lanka	2208	1809	4017	0.0061536	0.0050416	0.0111952	358814	560	541	1101
LKA	2012	country	3_Sear	Sri Lanka	2141	1653	3794	0.0060153	0.0046442	0.0106595	355927	548	504	1052
LKA	2013	country	3_Sear	Sri Lanka	2059	1486	3545	0.0058324	0.0042093	0.0100417	353030	529	463	992
LKA	2014	country	3_Sear	Sri Lanka	1946	1344	3290	0.0055591	0.0038394	0.0093985	350058	497	428	926
LKA	2015	country	3_Sear	Sri Lanka	1822	1229	3051	0.0052481	0.00354	0.0087882	347172	478	400	878
LKA	2016	country	3_Sear	Sri Lanka	1705	1141	2846	0.0049571	0.0033173	0.0082744	343952	443	379	822
LKA	2017	country	3_Sear	Sri Lanka	1601	1072	2673	0.0047068	0.0031516	0.0078584	340146	429	362	791
LKA	2018	country	3_Sear	Sri Lanka	1505	1011	2516	0.0044787	0.0030086	0.0074874	336033	400	347	747
LKA	2019	country	3_Sear	Sri Lanka	1416	962	2378	0.004273	0.002903	0.007176	331381	377	334	711

iso3	year	level	whoreg6	whoname	nnd	pnd	u5d	nmr	pnmr	u5mr	Livebirths	Neonatal birth defects deaths	1-59 month birth defects deaths	Under five birth defects deaths
LSO	2000	country	1_Afr	Lesotho	2291	4257	6548	0.0369382	0.0686406	0.1055787	62023	116	48	164
LSO	2001	country	1_Afr	Lesotho	2312	4365	6677	0.0371325	0.0701009	0.1072335	62263	119	44	163
LSO	2002	country	1_Afr	Lesotho	2324	4459	6783	0.0373037	0.0715754	0.1088791	62299	125	53	178
LSO	2003	country	1_Afr	Lesotho	2324	4555	6879	0.0374219	0.0733418	0.1107637	62103	123	64	187
LSO	2004	country	1_Afr	Lesotho	2321	4584	6905	0.0376051	0.0742697	0.1118748	61720	127	62	189
LSO	2005	country	1_Afr	Lesotho	2314	4607	6921	0.0378521	0.0753559	0.1132081	61133	129	60	188
LSO	2006	country	1_Afr	Lesotho	2309	4596	6905	0.0381754	0.0759817	0.1141571	60484	130	58	188
LSO	2007	country	1_Afr	Lesotho	2313	4387	6700	0.0386857	0.0733677	0.1120534	59790	132	59	192
LSO	2008	country	1_Afr	Lesotho	2324	4231	6555	0.0392828	0.0715094	0.1107922	59161	135	59	194
LSO	2009	country	1_Afr	Lesotho	2338	3740	6078	0.0398901	0.0638141	0.1037042	58611	135	75	210
LSO	2010	country	1_Afr	Lesotho	2358	3380	5738	0.0405043	0.0580665	0.0985708	58216	139	93	232
LSO	2011	country	1_Afr	Lesotho	2381	3200	5581	0.0411103	0.0552575	0.0963679	57917	144	105	248
LSO	2012	country	1_Afr	Lesotho	2407	3120	5527	0.0417026	0.0540537	0.0957563	57718	142	100	242
LSO	2013	country	1_Afr	Lesotho	2435	3026	5461	0.0423123	0.0525782	0.0948905	57548	147	95	242
LSO	2014	country	1_Afr	Lesotho	2463	2916	5379	0.0429142	0.0508119	0.0937262	57394	148	92	240
LSO	2015	country	1_Afr	Lesotho	2484	2876	5360	0.0433968	0.0502483	0.0936451	57239	151	90	241
LSO	2016	country	1_Afr	Lesotho	2492	2655	5147	0.0436842	0.0465339	0.0902181	57046	150	107	257
LSO	2017	country	1_Afr	Lesotho	2479	2541	5020	0.0436422	0.0447342	0.0883764	56803	156	110	266
LSO	2018	country	1_Afr	Lesotho	2452	2403	4855	0.0434219	0.04256	0.0859819	56469	151	115	266
LSO	2019	country	1_Afr	Lesotho	2401	2428	4829	0.042808	0.0432869	0.0860948	56088	153	111	264
LTU	2000	country	4_Eur	Lithuania	151	224	375	0.0045352	0.0067277	0.0112629	33295	67	70	137
LTU	2001	country	4_Eur	Lithuania	141	203	344	0.0044077	0.0063459	0.0107536	31989	56	73	128
LTU	2002	country	4_Eur	Lithuania	135	183	318	0.0043233	0.0058605	0.0101838	31226	63	55	118
LTU	2003	country	4_Eur	Lithuania	132	167	299	0.0043	0.0054401	0.00974	30698	42	52	94
LTU	2004	country	4_Eur	Lithuania	131	156	287	0.0043057	0.0051274	0.0094332	30425	51	53	104
LTU	2005	country	4_Eur	Lithuania	129	148	277	0.0042472	0.0048727	0.0091199	30373	41	48	89
LTU	2006	country	4_Eur	Lithuania	123	140	263	0.004029	0.0045859	0.0086149	30529	56	43	99
LTU	2007	country	4_Eur	Lithuania	113	133	246	0.0036515	0.0042977	0.0079492	30947	39	50	89
LTU	2008	country	4_Eur	Lithuania	104	123	227	0.003338	0.0039479	0.0072859	31156	37	43	80
LTU	2009	country	4_Eur	Lithuania	97	111	208	0.0030817	0.0035265	0.0066081	31476	30	34	64
LTU	2010	country	4_Eur	Lithuania	91	100	191	0.0028804	0.0031653	0.0060456	31593	32	31	63
LTU	2011	country	4_Eur	Lithuania	85	90	175	0.0026668	0.0028237	0.0054905	31873	26	33	59
LTU	2012	country	4_Eur	Lithuania	80	84	164	0.0025323	0.0026589	0.0051913	31592	24	23	47
LTU	2013	country	4_Eur	Lithuania	76	81	157	0.0024077	0.0025661	0.0049739	31565	27	29	56
LTU	2014	country	4_Eur	Lithuania	74	81	155	0.0023766	0.0026015	0.0049781	31136	34	35	69
LTU	2015	country	4_Eur	Lithuania	71	82	153	0.0023245	0.0026847	0.0050092	30544	28	32	60
LTU	2016	country	4_Eur	Lithuania	69	76	145	0.0022989	0.0025321	0.004831	30015	30	26	56
LTU	2017	country	4_Eur	Lithuania	66	66	132	0.0022156	0.0022156	0.0044312	29789	26	18	43
LTU	2018	country	4_Eur	Lithuania	62	57	119	0.0021413	0.0019686	0.0041099	28955	25	19	44
LTU	2019	country	4_Eur	Lithuania	58	48	106	0.0020479	0.0016948	0.0037428	28321	24	16	40

iso3	year	level	whoreg6	whoname	nnd	pnd	u5d	nmr	pnmr	u5mr	Livebirths	Neonatal birth defects deaths	1-59 month birth defects deaths	Under five birth defects deaths
LUX	2000	country	4_Eur	Luxembourg	13	12	25	0.0024298	0.0022429	0.0046726	5350	3	0	3
LUX	2001	country	4_Eur	Luxembourg	12	12	24	0.002315	0.002315	0.0046301	5183	2	2	3
LUX	2002	country	4_Eur	Luxembourg	12	11	23	0.0022075	0.0020235	0.004231	5436	2	2	3
LUX	2003	country	4_Eur	Luxembourg	11	10	21	0.0020985	0.0019078	0.0040063	5242	2	0	2
LUX	2004	country	4_Eur	Luxembourg	10	10	20	0.0018094	0.0018094	0.0036187	5527	2	2	4
LUX	2005	country	4_Eur	Luxembourg	10	9	19	0.0018881	0.0016993	0.0035873	5296	1	3	4
LUX	2006	country	4_Eur	Luxembourg	10	9	19	0.0017915	0.0016124	0.0034039	5582	0	3	3
LUX	2007	country	4_Eur	Luxembourg	9	9	18	0.0017083	0.0017083	0.0034165	5268	3	2	5
LUX	2008	country	4_Eur	Luxembourg	9	8	17	0.001639	0.0014569	0.0030958	5491	0	0	0
LUX	2009	country	4_Eur	Luxembourg	9	8	17	0.0015852	0.001409	0.0029942	5678	2	0	2
LUX	2010	country	4_Eur	Luxembourg	9	8	17	0.0015443	0.0013727	0.002917	5828	2	3	4
LUX	2011	country	4_Eur	Luxembourg	9	8	17	0.0015129	0.0013448	0.0028577	5949	2	0	2
LUX	2012	country	4_Eur	Luxembourg	9	8	17	0.0014884	0.001323	0.0028114	6047	0	3	3
LUX	2013	country	4_Eur	Luxembourg	9	8	17	0.0014658	0.001303	0.0027688	6140	2	3	5
LUX	2014	country	4_Eur	Luxembourg	9	8	17	0.0014431	0.0012828	0.0027259	6237	0	0	0
LUX	2015	country	4_Eur	Luxembourg	9	9	18	0.0014146	0.0014146	0.0028293	6362	4	3	7
LUX	2016	country	4_Eur	Luxembourg	10	8	18	0.0015338	0.001227	0.0027608	6520	4	0	4
LUX	2017	country	4_Eur	Luxembourg	10	8	18	0.0014944	0.0011955	0.0026899	6692	3	1	3
LUX	2018	country	4_Eur	Luxembourg	10	8	18	0.0016319	0.0013055	0.0029374	6128	4	1	4
LUX	2019	country	4_Eur	Luxembourg	9	9	18	0.001426	0.001426	0.002852	6311	3	1	4
LVA	2000	country	4_Eur	Latvia	138	140	278	0.0071585	0.0072623	0.0144208	19278	42	55	97
LVA	2001	country	4_Eur	Latvia	130	130	260	0.0066193	0.0066193	0.0132386	19640	51	35	86
LVA	2002	country	4_Eur	Latvia	125	124	249	0.0061812	0.0061318	0.012313	20223	38	37	75
LVA	2003	country	4_Eur	Latvia	122	120	242	0.0058328	0.0057371	0.0115699	20916	37	36	73
LVA	2004	country	4_Eur	Latvia	120	114	234	0.0055702	0.0052917	0.0108618	21543	29	39	68
LVA	2005	country	4_Eur	Latvia	118	107	225	0.0053725	0.0048717	0.0102443	21964	32	23	55
LVA	2006	country	4_Eur	Latvia	116	100	216	0.0051917	0.0044756	0.0096674	22343	30	28	58
LVA	2007	country	4_Eur	Latvia	113	94	207	0.0050043	0.0041628	0.0091671	22581	30	36	67
LVA	2008	country	4_Eur	Latvia	108	90	198	0.0047934	0.0039945	0.0087879	22531	29	36	64
LVA	2009	country	4_Eur	Latvia	101	86	187	0.0045253	0.0038532	0.0083785	22319	30	37	67
LVA	2010	country	4_Eur	Latvia	93	81	174	0.0042114	0.003668	0.0078795	22083	19	24	43
LVA	2011	country	4_Eur	Latvia	84	75	159	0.0038698	0.0034552	0.0073251	21706	19	28	47
LVA	2012	country	4_Eur	Latvia	75	69	144	0.0035213	0.0032396	0.0067608	21299	16	23	39
LVA	2013	country	4_Eur	Latvia	67	62	129	0.0031787	0.0029415	0.0061202	21078	14	14	28
LVA	2014	country	4_Eur	Latvia	60	56	116	0.0028676	0.0026765	0.0055441	20923	17	12	28
LVA	2015	country	4_Eur	Latvia	54	51	105	0.0025929	0.0024489	0.0050418	20826	13	18	31
LVA	2016	country	4_Eur	Latvia	49	46	95	0.0023561	0.0022118	0.0045679	20797	12	12	24
LVA	2017	country	4_Eur	Latvia	45	42	87	0.0021546	0.0020109	0.0041655	20886	11	11	22
LVA	2018	country	4_Eur	Latvia	41	39	80	0.0019889	0.0018919	0.0038809	20614	10	10	20
LVA	2019	country	4_Eur	Latvia	37	37	74	0.0018544	0.0018544	0.0037089	19952	9	10	19

iso3	year	level	whoreg6	whoname	nnd	pnd	u5d	nmr	pnmr	u5mr	Livebirths	Neonatal birth defects deaths	1-59 month birth defects deaths	Under five birth defects deaths
MAR	2000	country	5_Emr	Morocco	17206	14401	31607	0.0268669	0.0224869	0.0493538	640416	1122	2527	3650
MAR	2001	country	5_Emr	Morocco	16692	13412	30104	0.026093	0.0209657	0.0470587	639712	1117	1809	2926
MAR	2002	country	5_Emr	Morocco	16256	12505	28761	0.0253877	0.0195296	0.0449173	640311	1133	1652	2785
MAR	2003	country	5_Emr	Morocco	15851	11707	27558	0.0247032	0.0182449	0.0429481	641658	1132	1446	2578
MAR	2004	country	5_Emr	Morocco	15469	10961	26430	0.0240347	0.0170305	0.0410652	643610	1121	1534	2655
MAR	2005	country	5_Emr	Morocco	15110	10271	25381	0.0233673	0.0158839	0.0392512	646630	1134	1536	2669
MAR	2006	country	5_Emr	Morocco	14788	9646	24434	0.0227022	0.0148083	0.0375104	651392	1108	1438	2545
MAR	2007	country	5_Emr	Morocco	14510	9090	23600	0.0220413	0.0138081	0.0358494	658309	1141	1414	2556
MAR	2008	country	5_Emr	Morocco	14264	8563	22827	0.0213781	0.0128338	0.0342118	667225	1142	1406	2548
MAR	2009	country	5_Emr	Morocco	14013	8130	22143	0.020684	0.0120004	0.0326844	677479	1147	1368	2514
MAR	2010	country	5_Emr	Morocco	13741	7753	21494	0.0199751	0.0112705	0.0312456	687905	1164	1296	2459
MAR	2011	country	5_Emr	Morocco	13418	7434	20852	0.0192515	0.0106659	0.0299174	696986	1307	1305	2612
MAR	2012	country	5_Emr	Morocco	13047	7111	20158	0.0185427	0.0101063	0.028649	703621	1392	1295	2686
MAR	2013	country	5_Emr	Morocco	12508	6886	19394	0.0176946	0.0097413	0.0274359	706884	1462	1287	2749
MAR	2014	country	5_Emr	Morocco	11933	6658	18591	0.0168879	0.0094226	0.0263105	706600	1563	1293	2856
MAR	2015	country	5_Emr	Morocco	11338	6394	17732	0.0161278	0.0090952	0.0252229	703012	1613	1300	2912
MAR	2016	country	5_Emr	Morocco	10721	6141	16862	0.0153853	0.0088127	0.0241981	696832	1647	1316	2963
MAR	2017	country	5_Emr	Morocco	10161	5865	16026	0.014738	0.0085069	0.0232448	689444	1683	1319	3002
MAR	2018	country	5_Emr	Morocco	9660	5587	15247	0.0141682	0.0081944	0.0223626	681808	1662	1313	2975
MAR	2019	country	5_Emr	Morocco	9187	5324	14511	0.013619	0.0078924	0.0215114	674573	1644	1303	2947
MCO	2000	country	4_Eur	Monaco	1	1	2	0.0028671	0.0028671	0.0057342	349	0	0	0
MCO	2001	country	4_Eur	Monaco	1	1	2	0.0027752	0.0027752	0.0055504	360	0	0	0
MCO	2002	country	4_Eur	Monaco	1	1	2	0.0026942	0.0026942	0.0053884	371	0	0	0
MCO	2003	country	4_Eur	Monaco	1	1	2	0.0026212	0.0026212	0.0052424	382	0	0	0
MCO	2004	country	4_Eur	Monaco	1	1	2	0.0025497	0.0025497	0.0050994	392	0	0	0
MCO	2005	country	4_Eur	Monaco	1	1	2	0.002488	0.002488	0.0049759	402	0	0	0
MCO	2006	country	4_Eur	Monaco	1	1	2	0.0024304	0.0024304	0.0048609	411	0	0	0
MCO	2007	country	4_Eur	Monaco	1	1	2	0.0023779	0.0023779	0.0047558	421	0	0	0
MCO	2008	country	4_Eur	Monaco	1	1	2	0.0023238	0.0023238	0.0046476	430	0	0	0
MCO	2009	country	4_Eur	Monaco	1	1	2	0.0022738	0.0022738	0.0045476	440	0	0	0
MCO	2010	country	4_Eur	Monaco	1	1	2	0.0022242	0.0022242	0.0044484	450	0	0	0
MCO	2011	country	4_Eur	Monaco	1	1	2	0.0021712	0.0021712	0.0043425	461	0	0	0
MCO	2012	country	4_Eur	Monaco	1	1	2	0.0021194	0.0021194	0.0042389	472	0	0	0
MCO	2013	country	4_Eur	Monaco	1	1	2	0.0020656	0.0020656	0.0041313	484	0	0	0
MCO	2014	country	4_Eur	Monaco	1	1	2	0.0020081	0.0020081	0.0040163	498	0	0	0
MCO	2015	country	4_Eur	Monaco	1	1	2	0.0019452	0.0019452	0.0038905	514	0	0	0
MCO	2016	country	4_Eur	Monaco	1	1	2	0.0018853	0.0018853	0.0037707	530	0	0	0
MCO	2017	country	4_Eur	Monaco	1	1	2	0.0018197	0.0018197	0.0036394	550	0	0	0
MCO	2018	country	4_Eur	Monaco	1	1	2	0.0017585	0.0017585	0.0035171	569	0	0	0
MCO	2019	country	4_Eur	Monaco	1	1	2	0.0016977	0.0016977	0.0033953	589	0	0	0

iso3	year	level	whoreg6	whoname	nnd	pnd	u5d	nmr	pnmr	u5mr	Livebirths	Neonatal birth defects deaths	1-59 month birth defects deaths	Under five birth defects deaths
MDA	2000	country	4_Eur	Republic of Moldov	991	576	1567	0.0207253	0.0120462	0.0327715	47816	302	95	398
MDA	2001	country	4_Eur	Republic of Moldov	874	472	1346	0.0191496	0.0103417	0.0294913	45641	285	105	390
MDA	2002	country	4_Eur	Republic of Moldov	761	397	1158	0.0172821	0.0090158	0.0262979	44034	288	93	381
MDA	2003	country	4_Eur	Republic of Moldov	678	334	1012	0.015761	0.0077643	0.0235253	43018	202	72	274
MDA	2004	country	4_Eur	Republic of Moldov	623	287	910	0.0145914	0.0067219	0.0213133	42696	229	70	299
MDA	2005	country	4_Eur	Republic of Moldov	592	252	844	0.0138001	0.0058744	0.0196744	42898	204	56	260
MDA	2006	country	4_Eur	Republic of Moldov	578	229	807	0.0133228	0.0052784	0.0186011	43384	212	53	265
MDA	2007	country	4_Eur	Republic of Moldov	576	214	790	0.0130457	0.0048468	0.0178925	44152	166	47	213
MDA	2008	country	4_Eur	Republic of Moldov	577	203	780	0.0129059	0.0045406	0.0174465	44708	210	53	263
MDA	2009	country	4_Eur	Republic of Moldov	577	197	774	0.0127823	0.0043642	0.0171465	45140	176	44	220
MDA	2010	country	4_Eur	Republic of Moldov	574	194	768	0.0126762	0.0042843	0.0169605	45282	176	55	230
MDA	2011	country	4_Eur	Republic of Moldov	565	192	757	0.0125094	0.004251	0.0167604	45166	167	46	214
MDA	2012	country	4_Eur	Republic of Moldov	551	190	741	0.0122771	0.0042335	0.0165106	44880	220	60	280
MDA	2013	country	4_Eur	Republic of Moldov	535	187	722	0.0120627	0.0042163	0.016279	44352	167	42	208
MDA	2014	country	4_Eur	Republic of Moldov	518	184	702	0.0118324	0.004203	0.0160353	43778	174	49	223
MDA	2015	country	4_Eur	Republic of Moldov	501	179	680	0.0116036	0.0041458	0.0157494	43176	130	48	178
MDA	2016	country	4_Eur	Republic of Moldov	483	172	655	0.0113784	0.0040519	0.0154303	42449	143	40	183
MDA	2017	country	4_Eur	Republic of Moldov	464	168	632	0.0111368	0.0040323	0.0151691	41664	168	46	215
MDA	2018	country	4_Eur	Republic of Moldov	445	163	608	0.0108813	0.0039857	0.014867	40896	136	42	179
MDA	2019	country	4_Eur	Republic of Moldov	428	156	584	0.0106682	0.0038884	0.0145566	40119	138	40	178
MDG	2000	country	1_Afr	Madagascar	20116	48180	68296	0.0309029	0.0740161	0.104919	650942	1025	407	1433
MDG	2001	country	1_Afr	Madagascar	19828	47342	67170	0.030037	0.0717179	0.1017549	660118	1035	441	1476
MDG	2002	country	1_Afr	Madagascar	19557	46237	65794	0.0292349	0.0691181	0.098353	668961	1020	487	1506
MDG	2003	country	1_Afr	Madagascar	19310	43479	62789	0.0284891	0.0641468	0.0926358	677804	1011	550	1561
MDG	2004	country	1_Afr	Madagascar	19069	40180	59249	0.0277643	0.058502	0.0862663	686817	989	804	1793
MDG	2005	country	1_Afr	Madagascar	18834	36834	55668	0.0270574	0.052917	0.0799744	696076	1006	728	1734
MDG	2006	country	1_Afr	Madagascar	18618	35745	54363	0.0263891	0.0506644	0.0770536	705518	991	704	1695
MDG	2007	country	1_Afr	Madagascar	18426	34618	53044	0.0257666	0.0484092	0.0741758	715112	988	976	1964
MDG	2008	country	1_Afr	Madagascar	18236	33126	51362	0.0251603	0.045704	0.0708642	724793	980	831	1811
MDG	2009	country	1_Afr	Madagascar	18058	32443	50501	0.0245791	0.0441592	0.0687384	734688	982	734	1716
MDG	2010	country	1_Afr	Madagascar	17910	31730	49640	0.0240366	0.0425842	0.0666208	745113	980	808	1788
MDG	2011	country	1_Afr	Madagascar	17811	30315	48126	0.0235503	0.0400828	0.0636331	756298	971	748	1719
MDG	2012	country	1_Afr	Madagascar	17763	29932	47695	0.023116	0.0389525	0.0620685	768428	965	717	1683
MDG	2013	country	1_Afr	Madagascar	17727	29505	47232	0.0226819	0.037752	0.0604339	781549	982	826	1808
MDG	2014	country	1_Afr	Madagascar	17700	28249	45949	0.0222456	0.0355042	0.0577498	795662	966	875	1841
MDG	2015	country	1_Afr	Madagascar	17678	28185	45863	0.0218076	0.0347691	0.0565768	810633	997	734	1731
MDG	2016	country	1_Afr	Madagascar	17677	27884	45561	0.0213899	0.0337404	0.0551303	826418	979	797	1776
MDG	2017	country	1_Afr	Madagascar	17665	26532	44197	0.0209658	0.0314893	0.0524551	842565	984	796	1780
MDG	2018	country	1_Afr	Madagascar	17644	26615	44259	0.0205432	0.030988	0.0515313	858872	987	816	1804
MDG	2019	country	1_Afr	Madagascar	17607	38060	55667	0.0201197	0.043491	0.0636107	875113	973	812	1785

iso3	year	level	whoreg6	whoname	nnd	pnd	u5d	nmr	pnmr	u5mr	Livebirths	Neonatal birth defects deaths	1-59 month birth defects deaths	Under five birth defects deaths
MDV	2000	country	3_Sear	Maldives	132	111	243	0.0218216	0.0183499	0.0401715	6049	13	14	26
MDV	2001	country	3_Sear	Maldives	115	93	208	0.0194557	0.0157338	0.0351895	5911	12	12	24
MDV	2002	country	3_Sear	Maldives	103	99	202	0.0175468	0.0168433	0.0343902	5870	12	10	23
MDV	2003	country	3_Sear	Maldives	91	69	160	0.0153581	0.01171	0.0270681	5925	13	10	22
MDV	2004	country	3_Sear	Maldives	82	79	161	0.0136241	0.0130485	0.0266726	6019	13	10	23
MDV	2005	country	3_Sear	Maldives	75	57	132	0.0120527	0.0091365	0.0211892	6223	13	10	23
MDV	2006	country	3_Sear	Maldives	70	52	122	0.0109018	0.0080272	0.018929	6421	14	10	24
MDV	2007	country	3_Sear	Maldives	66	48	114	0.0098345	0.0071934	0.0170279	6711	14	10	24
MDV	2008	country	3_Sear	Maldives	63	45	108	0.0090933	0.0064952	0.0155884	6928	14	10	24
MDV	2009	country	3_Sear	Maldives	59	44	103	0.0082195	0.0061298	0.0143492	7178	13	11	24
MDV	2010	country	3_Sear	Maldives	56	43	99	0.0075981	0.0058343	0.0134324	7370	12	11	23
MDV	2011	country	3_Sear	Maldives	52	43	95	0.0069496	0.0057467	0.0126963	7482	12	11	23
MDV	2012	country	3_Sear	Maldives	49	42	91	0.0065263	0.005594	0.0121202	7508	11	12	23
MDV	2013	country	3_Sear	Maldives	47	39	86	0.0063031	0.0052302	0.0115333	7457	11	11	22
MDV	2014	country	3_Sear	Maldives	45	36	81	0.0060122	0.0048098	0.010822	7485	10	11	21
MDV	2015	country	3_Sear	Maldives	44	32	76	0.0059051	0.0042946	0.0101998	7451	11	10	20
MDV	2016	country	3_Sear	Maldives	42	28	70	0.0056913	0.0037942	0.0094855	7380	10	9	19
MDV	2017	country	3_Sear	Maldives	40	25	65	0.0054935	0.0034335	0.008927	7281	10	8	18
MDV	2018	country	3_Sear	Maldives	38	21	59	0.005294	0.0029256	0.0082196	7178	9	7	16
MDV	2019	country	3_Sear	Maldives	34	20	54	0.0048146	0.0028321	0.0076467	7062	9	7	15
MEX	2000	country	2_Amr	Mexico	33505	32963	66468	0.0142022	0.0139725	0.0281747	2359138	6572	6320	12892
MEX	2001	country	2_Amr	Mexico	31036	32319	63355	0.0131999	0.0137456	0.0269455	2351228	6198	6311	12509
MEX	2002	country	2_Amr	Mexico	28605	31799	60404	0.012214	0.0135778	0.0257919	2341977	5713	5908	11621
MEX	2003	country	2_Amr	Mexico	26320	31331	57651	0.0112886	0.0134378	0.0247264	2331557	5428	6181	11609
MEX	2004	country	2_Amr	Mexico	24265	30793	55058	0.010458	0.0132715	0.0237295	2320232	5107	6316	11423
MEX	2005	country	2_Amr	Mexico	22632	30063	52695	0.009801	0.013019	0.02282	2309160	4994	6311	11305
MEX	2006	country	2_Amr	Mexico	21593	28954	50547	0.0093895	0.0125904	0.02198	2299686	5129	6304	11433
MEX	2007	country	2_Amr	Mexico	21137	27457	48594	0.0092188	0.0119753	0.0211941	2292805	4973	5915	10888
MEX	2008	country	2_Amr	Mexico	20965	25856	46821	0.009161	0.0112981	0.0204591	2288516	5030	5831	10861
MEX	2009	country	2_Amr	Mexico	20818	24365	45183	0.0091034	0.0106545	0.0197579	2286831	5011	5622	10633
MEX	2010	country	2_Amr	Mexico	20569	23083	43652	0.0089954	0.0100948	0.0190902	2286619	4989	5711	10701
MEX	2011	country	2_Amr	Mexico	20199	22001	42200	0.0088342	0.0096223	0.0184565	2286460	4984	5493	10477
MEX	2012	country	2_Amr	Mexico	19723	21055	40778	0.0086322	0.0092151	0.0178473	2284830	4973	5492	10465
MEX	2013	country	2_Amr	Mexico	19260	20148	39408	0.008446	0.0088354	0.0172814	2280371	5080	5113	10193
MEX	2014	country	2_Amr	Mexico	18917	19121	38038	0.0083231	0.0084129	0.016736	2272830	4837	5341	10179
MEX	2015	country	2_Amr	Mexico	18777	17908	36685	0.0083002	0.007916	0.0162162	2262247	4679	5117	9796
MEX	2016	country	2_Amr	Mexico	18864	16453	35317	0.0083867	0.0073148	0.0157015	2249269	4450	4767	9217
MEX	2017	country	2_Amr	Mexico	19110	14876	33986	0.0085497	0.0066554	0.0152051	2235174	4719	4238	8958
MEX	2018	country	2_Amr	Mexico	19215	13441	32656	0.0086527	0.0060526	0.0147053	2220694	4689	3830	8518
MEX	2019	country	2_Amr	Mexico	18906	12462	31368	0.0085686	0.0056481	0.0142167	2206420	4581	3551	8132

iso3	year	level	whoreg6	whoname	nnd	pnd	u5d	nmr	pnmr	u5mr	Livebirths	Neonatal birth defects deaths	1-59 month birth defects deaths	Under five birth defects deaths
MHL	2000	country	6_Wpr	Marshall Islands	34	42	76	0.0183024	0.0226089	0.0409113	1858	4	6	10
MHL	2001	country	6_Wpr	Marshall Islands	34	42	76	0.0183121	0.0226209	0.040933	1857	4	3	7
MHL	2002	country	6_Wpr	Marshall Islands	34	41	75	0.0182958	0.0220626	0.0403584	1858	4	5	8
MHL	2003	country	6_Wpr	Marshall Islands	33	41	74	0.0182259	0.0226443	0.0408702	1811	4	6	9
MHL	2004	country	6_Wpr	Marshall Islands	33	40	73	0.0181264	0.0219714	0.0400978	1821	4	4	7
MHL	2005	country	6_Wpr	Marshall Islands	32	40	72	0.0180061	0.0225077	0.0405138	1777	4	5	9
MHL	2006	country	6_Wpr	Marshall Islands	32	39	71	0.0179191	0.0218389	0.039758	1786	4	6	10
MHL	2007	country	6_Wpr	Marshall Islands	31	39	70	0.0178536	0.022461	0.0403145	1736	4	6	10
MHL	2008	country	6_Wpr	Marshall Islands	31	38	69	0.0177825	0.0217979	0.0395804	1743	4	5	9
MHL	2009	country	6_Wpr	Marshall Islands	30	37	67	0.0176893	0.0218167	0.039506	1696	4	4	8
MHL	2010	country	6_Wpr	Marshall Islands	29	36	65	0.0176194	0.0218724	0.0394918	1646	3	6	9
MHL	2011	country	6_Wpr	Marshall Islands	28	35	63	0.0175328	0.021916	0.0394488	1597	3	5	8
MHL	2012	country	6_Wpr	Marshall Islands	27	34	61	0.0173985	0.0219092	0.0393077	1552	3	4	7
MHL	2013	country	6_Wpr	Marshall Islands	26	33	59	0.0171459	0.0217621	0.038908	1516	3	4	7
MHL	2014	country	6_Wpr	Marshall Islands	25	31	56	0.0168938	0.0209483	0.0378422	1480	3	3	7
MHL	2015	country	6_Wpr	Marshall Islands	24	29	53	0.0166086	0.0200687	0.0366773	1445	3	3	6
MHL	2016	country	6_Wpr	Marshall Islands	23	28	51	0.0162846	0.0198247	0.0361092	1412	3	3	6
MHL	2017	country	6_Wpr	Marshall Islands	22	26	48	0.0159243	0.0188197	0.034744	1382	3	3	6
MHL	2018	country	6_Wpr	Marshall Islands	22	24	46	0.0156377	0.0170593	0.032697	1407	3	3	6
MHL	2019	country	6_Wpr	Marshall Islands	21	23	44	0.0153136	0.0167721	0.0320857	1371	3	3	6
MKD	2000	country	4_Eur	Republic of North M	240	184	424	0.0090939	0.006972	0.0160658	26391	32	39	71
MKD	2001	country	4_Eur	Republic of North M	223	171	394	0.0086079	0.0066007	0.0152085	25907	33	47	80
MKD	2002	country	4_Eur	Republic of North M	214	159	373	0.0084376	0.0062691	0.0147066	25363	34	47	81
MKD	2003	country	4_Eur	Republic of North M	212	146	358	0.0085246	0.0058707	0.0143953	24869	32	44	77
MKD	2004	country	4_Eur	Republic of North M	214	132	346	0.0087952	0.0054251	0.0142202	24332	37	42	79
MKD	2005	country	4_Eur	Republic of North M	215	117	332	0.0090089	0.0049025	0.0139114	23865	40	40	80
MKD	2006	country	4_Eur	Republic of North M	208	107	315	0.0088545	0.004555	0.0134094	23491	28	47	76
MKD	2007	country	4_Eur	Republic of North M	196	98	294	0.0084678	0.0042339	0.0127017	23147	40	36	75
MKD	2008	country	4_Eur	Republic of North M	179	91	270	0.0077974	0.003964	0.0117615	22956	24	35	59
MKD	2009	country	4_Eur	Republic of North M	163	84	247	0.0070756	0.0036463	0.010722	23037	19	23	42
MKD	2010	country	4_Eur	Republic of North M	152	80	232	0.0066042	0.0034759	0.0100801	23016	12	28	39
MKD	2011	country	4_Eur	Republic of North M	153	77	230	0.006602	0.0033226	0.0099246	23175	13	21	34
MKD	2012	country	4_Eur	Republic of North M	164	78	242	0.0070388	0.0033477	0.0103865	23300	8	12	20
MKD	2013	country	4_Eur	Republic of North M	185	80	265	0.0079195	0.0034246	0.0113441	23360	10	31	40
MKD	2014	country	4_Eur	Republic of North M	211	80	291	0.0090388	0.003427	0.0124659	23344	13	22	34
MKD	2015	country	4_Eur	Republic of North M	223	78	301	0.009605	0.0033596	0.0129646	23217	12	21	33
MKD	2016	country	4_Eur	Republic of North M	203	74	277	0.0088092	0.0032112	0.0120204	23044	11	20	31
MKD	2017	country	4_Eur	Republic of North M	161	67	228	0.0070434	0.0029311	0.0099745	22858	9	18	27
MKD	2018	country	4_Eur	Republic of North M	119	59	178	0.0052845	0.0026201	0.0079046	22519	7	16	22
MKD	2019	country	4_Eur	Republic of North M	87	50	137	0.0039164	0.0022508	0.0061673	22214	5	13	18

iso3	year	level	whoreg6	whoname	nnd	pnd	u5d	nmr	pnmr	u5mr	Livebirths	Neonatal birth defects deaths	1-59 month birth defects deaths	Under five birth defects deaths
MLI	2000	country	1_Afr	Mali	27196	69152	96348	0.050724	0.1289776	0.1797016	536157	964	61	1025
MLI	2001	country	1_Afr	Mali	27080	72833	99913	0.0490332	0.1318775	0.1809108	552279	1007	83	1090
MLI	2002	country	1_Afr	Mali	26958	64979	91937	0.0473714	0.1141832	0.1615546	569077	1034	104	1139
MLI	2003	country	1_Afr	Mali	26885	65099	91984	0.0458539	0.1110305	0.1568844	586319	1077	180	1257
MLI	2004	country	1_Afr	Mali	26893	64911	91804	0.0445417	0.1075087	0.1520505	603771	1152	225	1377
MLI	2005	country	1_Afr	Mali	26955	63604	90559	0.0434001	0.1024091	0.1458092	621082	1166	307	1473
MLI	2006	country	1_Afr	Mali	27047	63320	90367	0.0424029	0.0992704	0.1416733	637858	1211	305	1516
MLI	2007	country	1_Afr	Mali	27143	62699	89842	0.0415105	0.0958872	0.1373977	653883	1246	233	1479
MLI	2008	country	1_Afr	Mali	27183	61433	88616	0.0406301	0.0918231	0.1324532	669036	1226	490	1716
MLI	2009	country	1_Afr	Mali	27145	63900	91045	0.0397295	0.093524	0.1332535	683245	1300	396	1696
MLI	2010	country	1_Afr	Mali	27030	62230	89260	0.0388087	0.089348	0.1281568	696493	1305	475	1780
MLI	2011	country	1_Afr	Mali	26858	59649	86507	0.0378883	0.0841466	0.1220349	708874	1326	418	1745
MLI	2012	country	1_Afr	Mali	26672	57423	84095	0.0370078	0.0796756	0.1166835	720712	1332	491	1824
MLI	2013	country	1_Afr	Mali	26519	56404	82923	0.0362124	0.0770219	0.1132343	732318	1337	275	1612
MLI	2014	country	1_Afr	Mali	26405	55292	81697	0.035495	0.0743261	0.1098211	743907	1396	313	1709
MLI	2015	country	1_Afr	Mali	26271	54014	80285	0.034761	0.0714703	0.1062313	755760	1395	431	1826
MLI	2016	country	1_Afr	Mali	26165	51387	77552	0.0340692	0.0669103	0.1009795	767996	1405	998	2404
MLI	2017	country	1_Afr	Mali	26116	50278	76394	0.0334509	0.0643984	0.0978493	780726	1397	800	2197
MLI	2018	country	1_Afr	Mali	26034	49794	75828	0.0327891	0.0627148	0.0955039	793983	1442	1343	2786
MLI	2019	country	1_Afr	Mali	25958	48718	74676	0.0321344	0.0603099	0.0924442	807796	1425	911	2336
MLT	2000	country	4_Eur	Malta	22	12	34	0.0050526	0.002756	0.0078086	4354	6	0	6
MLT	2001	country	4_Eur	Malta	21	11	32	0.0048843	0.0025585	0.0074428	4299	5	2	8
MLT	2002	country	4_Eur	Malta	19	11	30	0.0047445	0.0027468	0.0074913	4005	10	5	15
MLT	2003	country	4_Eur	Malta	18	10	28	0.0044009	0.0024449	0.0068458	4090	9	3	11
MLT	2004	country	4_Eur	Malta	18	9	27	0.0045665	0.0022833	0.0068498	3942	10	2	12
MLT	2005	country	4_Eur	Malta	17	10	27	0.0045228	0.0026604	0.0071832	3759	4	2	6
MLT	2006	country	4_Eur	Malta	17	9	26	0.0045017	0.0023832	0.0068849	3776	8	2	10
MLT	2007	country	4_Eur	Malta	17	9	26	0.0045018	0.0023833	0.0068851	3776	9	5	13
MLT	2008	country	4_Eur	Malta	18	8	26	0.0045206	0.0020091	0.0065297	3982	8	2	10
MLT	2009	country	4_Eur	Malta	18	8	26	0.0045418	0.0020186	0.0065604	3963	10	3	13
MLT	2010	country	4_Eur	Malta	18	9	27	0.0045687	0.0022844	0.0068531	3940	11	3	14
MLT	2011	country	4_Eur	Malta	18	9	27	0.0045889	0.0022944	0.0068833	3923	8	5	12
MLT	2012	country	4_Eur	Malta	19	8	27	0.0046112	0.0019416	0.0065528	4120	10	0	10
MLT	2013	country	4_Eur	Malta	19	9	28	0.0046387	0.0021973	0.006836	4096	5	2	8
MLT	2014	country	4_Eur	Malta	19	10	29	0.0046729	0.0024594	0.0071323	4066	5	4	9
MLT	2015	country	4_Eur	Malta	20	9	29	0.0047101	0.0021196	0.0068297	4246	8	3	11
MLT	2016	country	4_Eur	Malta	20	10	30	0.0047456	0.0023728	0.0071184	4214	6	4	10
MLT	2017	country	4_Eur	Malta	20	10	30	0.0047666	0.0023833	0.0071499	4196	6	4	10
MLT	2018	country	4_Eur	Malta	20	10	30	0.0047557	0.0023778	0.0071335	4205	7	4	10
MLT	2019	country	4_Eur	Malta	20	10	30	0.0047058	0.0023529	0.0070587	4250	6	4	10

iso3	year	level	whoreg6	whoname	nnd	pnd	u5d	nmr	pnmr	u5mr	Livebirths	Neonatal birth defects deaths	1-59 month birth defects deaths	Under five birth defects deaths
MMR	2000	country	3_Sear	Myanmar	42752	57185	99937	0.0372845	0.0498712	0.0871557	1146644	2959	2090	5049
MMR	2001	country	3_Sear	Myanmar	41708	58981	100689	0.0363082	0.0513452	0.0876535	1148720	2971	1629	4600
MMR	2002	country	3_Sear	Myanmar	40460	55797	96257	0.035294	0.0486724	0.0839665	1146370	2916	1813	4730
MMR	2003	country	3_Sear	Myanmar	39072	53694	92766	0.0342946	0.0471286	0.0814232	1139304	2890	1961	4850
MMR	2004	country	3_Sear	Myanmar	37539	52406	89945	0.0332941	0.0464799	0.079774	1127498	2826	2265	5090
MMR	2005	country	3_Sear	Myanmar	35946	49351	85297	0.0323484	0.0444115	0.07676	1111213	2728	2137	4864
MMR	2006	country	3_Sear	Myanmar	34343	47473	81816	0.0314755	0.0435095	0.074985	1091103	2672	1836	4507
MMR	2007	country	3_Sear	Myanmar	32736	45435	78171	0.0306283	0.0425096	0.073138	1068814	2604	1909	4514
MMR	2008	country	3_Sear	Myanmar	31512	63483	94995	0.0301287	0.060696	0.0908246	1045914	2531	1876	4408
MMR	2009	country	3_Sear	Myanmar	29637	38516	68153	0.0289504	0.0376233	0.0665736	1023718	2442	2033	4475
MMR	2010	country	3_Sear	Myanmar	28284	36006	64290	0.028186	0.0358809	0.0640669	1003477	2359	1967	4325
MMR	2011	country	3_Sear	Myanmar	27078	33957	61035	0.0274551	0.0344304	0.0618855	986264	2278	1924	4202
MMR	2012	country	3_Sear	Myanmar	26013	31804	57817	0.0267541	0.03271	0.0594641	972299	2173	1681	3854
MMR	2013	country	3_Sear	Myanmar	25056	29366	54422	0.0260591	0.0305416	0.0566007	961507	2143	1792	3935
MMR	2014	country	3_Sear	Myanmar	24228	27051	51279	0.0254	0.0283595	0.0537595	953858	2117	1854	3971
MMR	2015	country	3_Sear	Myanmar	23502	25355	48857	0.0247641	0.0267162	0.0514803	949034	2043	1663	3706
MMR	2016	country	3_Sear	Myanmar	22852	23692	46544	0.0241483	0.0250356	0.0491839	946319	2017	1889	3906
MMR	2017	country	3_Sear	Myanmar	22250	23034	45284	0.0235542	0.024384	0.0479382	944631	1999	1673	3672
MMR	2018	country	3_Sear	Myanmar	21728	23342	45070	0.0230379	0.024749	0.0477868	943143	1984	1990	3974
MMR	2019	country	3_Sear	Myanmar	21134	23951	45085	0.02245	0.0254425	0.0478925	941381	1917	1924	3841
MNE	2000	country	4_Eur	Montenegro	74	47	121	0.0087519	0.0055587	0.0143106	8455	6	12	18
MNE	2001	country	4_Eur	Montenegro	71	44	115	0.0084592	0.0052423	0.0137016	8393	6	11	17
MNE	2002	country	4_Eur	Montenegro	69	40	109	0.0083309	0.0048295	0.0131604	8282	6	10	16
MNE	2003	country	4_Eur	Montenegro	66	36	102	0.0080223	0.0043758	0.0123981	8227	5	9	14
MNE	2004	country	4_Eur	Montenegro	62	33	95	0.0075385	0.0040124	0.0115509	8224	6	8	13
MNE	2005	country	4_Eur	Montenegro	57	31	88	0.0068949	0.0037499	0.0106448	8267	6	9	14
MNE	2006	country	4_Eur	Montenegro	53	28	81	0.0064817	0.0034243	0.009906	8177	3	4	7
MNE	2007	country	4_Eur	Montenegro	48	25	73	0.0059439	0.0030958	0.0090397	8075	6	5	11
MNE	2008	country	4_Eur	Montenegro	43	23	66	0.0054133	0.0028955	0.0083087	7943	1	0	1
MNE	2009	country	4_Eur	Montenegro	38	20	58	0.0047646	0.0025077	0.0072723	7976	4	0	4
MNE	2010	country	4_Eur	Montenegro	34	18	52	0.0043877	0.0023229	0.0067106	7749	3	1	4
MNE	2011	country	4_Eur	Montenegro	29	16	45	0.0037764	0.0020835	0.0058599	7679	2	1	3
MNE	2012	country	4_Eur	Montenegro	25	15	40	0.00333	0.001998	0.005328	7507	2	1	3
MNE	2013	country	4_Eur	Montenegro	21	14	35	0.0027965	0.0018644	0.0046609	7509	2	1	3
MNE	2014	country	4_Eur	Montenegro	18	12	30	0.0024323	0.0016216	0.0040539	7400	1	1	2
MNE	2015	country	4_Eur	Montenegro	16	11	27	0.0021215	0.0014586	0.0035801	7542	1	1	2
MNE	2016	country	4_Eur	Montenegro	13	10	23	0.0017319	0.0013322	0.0030641	7506	1	1	2
MNE	2017	country	4_Eur	Montenegro	12	9	21	0.001664	0.001248	0.002912	7211	1	1	2
MNE	2018	country	4_Eur	Montenegro	10	9	19	0.0013799	0.0012419	0.0026218	7247	1	1	1
MNE	2019	country	4_Eur	Montenegro	10	7	17	0.0013988	0.0009791	0.0023779	7149	1	1	1

iso3	year	level	whoreg6	whoname	nnd	pnd	u5d	nmr	pnmr	u5mr	Livebirths	Neonatal birth defects deaths	1-59 month birth defects deaths	Under five birth defects deaths
MNG	2000	country	6_Wpr	Mongolia	1089	2004	3093	0.0235072	0.0432622	0.0667695	46326	117	195	312
MNG	2001	country	6_Wpr	Mongolia	1020	1809	2829	0.0221414	0.039272	0.0614134	46067	115	200	315
MNG	2002	country	6_Wpr	Mongolia	967	1623	2590	0.0208318	0.0349606	0.0557924	46419	111	206	317
MNG	2003	country	6_Wpr	Mongolia	926	1485	2411	0.0195335	0.0313212	0.0508547	47406	108	201	309
MNG	2004	country	6_Wpr	Mongolia	898	1374	2272	0.0183217	0.0280339	0.0463556	49013	109	202	311
MNG	2005	country	6_Wpr	Mongolia	876	1296	2172	0.0170955	0.0252959	0.0423914	51241	112	195	307
MNG	2006	country	6_Wpr	Mongolia	859	1241	2100	0.015909	0.0229902	0.0388992	53995	114	207	321
MNG	2007	country	6_Wpr	Mongolia	843	1208	2051	0.0147453	0.021138	0.0358833	57171	119	208	327
MNG	2008	country	6_Wpr	Mongolia	823	1191	2014	0.0135847	0.0196623	0.033247	60583	124	207	331
MNG	2009	country	6_Wpr	Mongolia	800	1179	1979	0.0124882	0.0184091	0.0308973	64060	127	207	334
MNG	2010	country	6_Wpr	Mongolia	784	1153	1937	0.0116307	0.0171103	0.0287409	67408	136	214	350
MNG	2011	country	6_Wpr	Mongolia	768	1114	1882	0.0109098	0.0158309	0.0267407	70396	137	216	353
MNG	2012	country	6_Wpr	Mongolia	757	1054	1811	0.0103628	0.014435	0.0247978	73050	145	213	357
MNG	2013	country	6_Wpr	Mongolia	753	975	1728	0.0100246	0.01298	0.0230046	75116	151	204	355
MNG	2014	country	6_Wpr	Mongolia	752	884	1636	0.0098242	0.0115487	0.0213729	76545	156	192	348
MNG	2015	country	6_Wpr	Mongolia	743	824	1567	0.0095987	0.0106449	0.0202436	77406	156	179	334
MNG	2016	country	6_Wpr	Mongolia	722	761	1483	0.0093138	0.00982	0.0191338	77519	156	167	323
MNG	2017	country	6_Wpr	Mongolia	690	660	1350	0.0089435	0.0085547	0.0174982	77151	153	157	311
MNG	2018	country	6_Wpr	Mongolia	653	610	1263	0.0085562	0.0079928	0.016549	76319	147	149	296
MNG	2019	country	6_Wpr	Mongolia	611	572	1183	0.0081313	0.0076123	0.0157435	75142	138	143	281
MOZ	2000	country	1_Afr	Mozambique	36753	91796	128549	0.0461353	0.1152295	0.1613647	796635	1456	124	1580
MOZ	2001	country	1_Afr	Mozambique	35984	89739	125723	0.0440733	0.1099129	0.1539862	816458	1479	143	1621
MOZ	2002	country	1_Afr	Mozambique	35264	87781	123045	0.0422226	0.1051021	0.1473246	835193	1496	185	1681
MOZ	2003	country	1_Afr	Mozambique	34601	87099	121700	0.0405686	0.1021209	0.1426895	852902	1516	246	1762
MOZ	2004	country	1_Afr	Mozambique	34023	85331	119354	0.0391258	0.0981289	0.1372547	869579	1545	313	1858
MOZ	2005	country	1_Afr	Mozambique	33500	85506	119006	0.0378395	0.0965823	0.1344218	885318	1579	382	1961
MOZ	2006	country	1_Afr	Mozambique	33111	75361	108472	0.0367773	0.083705	0.1204823	900311	1614	406	2021
MOZ	2007	country	1_Afr	Mozambique	32807	72018	104825	0.03586	0.0787201	0.1145801	914864	1645	437	2082
MOZ	2008	country	1_Afr	Mozambique	32592	68944	101536	0.0350691	0.0741841	0.1092532	929365	1673	501	2175
MOZ	2009	country	1_Afr	Mozambique	32409	65622	98031	0.0343282	0.0695083	0.1038365	944093	1700	571	2271
MOZ	2010	country	1_Afr	Mozambique	32337	66330	98667	0.0337071	0.06914	0.1028472	959352	1731	625	2355
MOZ	2011	country	1_Afr	Mozambique	32302	63150	95452	0.0331202	0.0647493	0.0978695	975295	1762	668	2430
MOZ	2012	country	1_Afr	Mozambique	32294	60243	92537	0.0325539	0.0607274	0.0932813	992017	1794	706	2500
MOZ	2013	country	1_Afr	Mozambique	32351	58132	90483	0.0320478	0.0575867	0.0896346	1009460	1830	799	2628
MOZ	2014	country	1_Afr	Mozambique	32315	56701	89016	0.0314429	0.0551704	0.0866133	1027735	1860	857	2718
MOZ	2015	country	1_Afr	Mozambique	32336	54698	87034	0.0308887	0.0522498	0.0831386	1046854	1896	913	2808
MOZ	2016	country	1_Afr	Mozambique	32376	52584	84960	0.0303475	0.0492896	0.0796371	1066841	1934	971	2905
MOZ	2017	country	1_Afr	Mozambique	32386	51637	84023	0.0297811	0.0474837	0.0772648	1087468	1970	1062	3032
MOZ	2018	country	1_Afr	Mozambique	32342	50848	83190	0.0291733	0.045866	0.0750394	1108615	1995	1207	3202
MOZ	2019	country	1_Afr	Mozambique	32259	49179	81438	0.0285459	0.0435182	0.0720641	1130076	2012	1218	3230

iso3	year	level	whoreg6	whoname	nnd	pnd	u5d	nmr	pnmr	u5mr	Livebirths	Neonatal birth defects deaths	1-59 month birth defects deaths	Under five birth defects deaths
MRT	2000	country	1_Afr	Mauritania	4318	6468	10786	0.0429415	0.0643188	0.1072603	100555	193	43	236
MRT	2001	country	1_Afr	Mauritania	4385	8200	12585	0.0426146	0.0796935	0.1223081	102899	200	61	262
MRT	2002	country	1_Afr	Mauritania	4460	7650	12110	0.0423538	0.0726436	0.1149974	105304	207	138	345
MRT	2003	country	1_Afr	Mauritania	4540	7522	12062	0.0421178	0.0697822	0.1119	107793	216	136	351
MRT	2004	country	1_Afr	Mauritania	4624	8075	12699	0.0419041	0.0731809	0.115085	110347	223	111	333
MRT	2005	country	1_Afr	Mauritania	4685	6677	11362	0.0414522	0.0590733	0.1005255	113022	228	96	324
MRT	2006	country	1_Afr	Mauritania	4744	6855	11599	0.040968	0.0591995	0.1001675	115798	238	94	333
MRT	2007	country	1_Afr	Mauritania	4801	6977	11778	0.0404464	0.05878	0.0992264	118700	248	114	363
MRT	2008	country	1_Afr	Mauritania	4839	7074	11913	0.0397597	0.0581271	0.0978868	121706	254	117	371
MRT	2009	country	1_Afr	Mauritania	4879	7339	12218	0.039104	0.0588236	0.0979276	124770	262	109	371
MRT	2010	country	1_Afr	Mauritania	4895	8303	13198	0.0382934	0.0649501	0.1032435	127829	268	142	410
MRT	2011	country	1_Afr	Mauritania	4915	7672	12587	0.037562	0.0586335	0.0961955	130850	275	150	424
MRT	2012	country	1_Afr	Mauritania	4944	6776	11720	0.0369647	0.0506587	0.0876234	133749	275	189	464
MRT	2013	country	1_Afr	Mauritania	4944	6807	11751	0.0362175	0.049862	0.0860795	136509	277	221	498
MRT	2014	country	1_Afr	Mauritania	4938	6805	11743	0.0354892	0.0489051	0.0843944	139141	282	218	501
MRT	2015	country	1_Afr	Mauritania	4931	6301	11232	0.0348299	0.0445075	0.0793374	141574	294	202	496
MRT	2016	country	1_Afr	Mauritania	4927	6289	11216	0.0342357	0.0436969	0.0779326	143914	302	217	519
MRT	2017	country	1_Afr	Mauritania	4894	6311	11205	0.0334804	0.043173	0.0766534	146175	295	254	548
MRT	2018	country	1_Afr	Mauritania	4858	6301	11159	0.0327455	0.04247	0.0752156	148356	300	257	557
MRT	2019	country	1_Afr	Mauritania	4820	5818	10638	0.0320155	0.0386421	0.0706577	150552	296	259	555
MUS	2000	country	1_Afr	Mauritius	249	132	381	0.0123798	0.0065628	0.0189426	20113	36	45	80
MUS	2001	country	1_Afr	Mauritius	224	125	349	0.0113542	0.006336	0.0176902	19728	32	33	65
MUS	2002	country	1_Afr	Mauritius	205	120	325	0.010548	0.0061744	0.0167224	19435	43	28	71
MUS	2003	country	1_Afr	Mauritius	193	115	308	0.0101987	0.0060769	0.0162756	18924	44	24	68
MUS	2004	country	1_Afr	Mauritius	187	109	296	0.0101007	0.0058876	0.0159883	18513	37	28	65
MUS	2005	country	1_Afr	Mauritius	182	104	286	0.010157	0.005804	0.015961	17919	48	32	80
MUS	2006	country	1_Afr	Mauritius	175	99	274	0.0100926	0.0057095	0.0158021	17339	33	22	55
MUS	2007	country	1_Afr	Mauritius	167	94	261	0.0099938	0.0056253	0.0156191	16710	29	23	52
MUS	2008	country	1_Afr	Mauritius	157	90	247	0.0097051	0.0055635	0.0152686	16177	28	26	55
MUS	2009	country	1_Afr	Mauritius	147	87	234	0.009441	0.0055875	0.0150285	15570	39	23	62
MUS	2010	country	1_Afr	Mauritius	140	84	224	0.0093069	0.0055841	0.014891	15043	30	29	59
MUS	2011	country	1_Afr	Mauritius	136	83	219	0.0093476	0.0057048	0.0150525	14549	35	26	61
MUS	2012	country	1_Afr	Mauritius	134	83	217	0.0094745	0.0058685	0.015343	14143	35	20	55
MUS	2013	country	1_Afr	Mauritius	131	81	212	0.0094944	0.0058706	0.015365	13798	27	20	47
MUS	2014	country	1_Afr	Mauritius	125	78	203	0.0092502	0.0057721	0.0150223	13513	36	24	59
MUS	2015	country	1_Afr	Mauritius	121	75	196	0.0090706	0.0056223	0.0146928	13340	18	27	45
MUS	2016	country	1_Afr	Mauritius	119	74	193	0.0090634	0.0056361	0.0146995	13130	25	27	53
MUS	2017	country	1_Afr	Mauritius	121	74	195	0.0093153	0.005697	0.0150123	12989	25	21	46
MUS	2018	country	1_Afr	Mauritius	125	74	199	0.0097377	0.0057647	0.0155024	12837	24	25	49
MUS	2019	country	1_Afr	Mauritius	130	76	206	0.0101816	0.0059523	0.0161339	12768	26	26	52

iso3	year	level	whoreg6	whoname	nnd	pnd	u5d	nmr	pnmr	u5mr	Livebirths	Neonatal birth defects deaths	1-59 month birth defects deaths	Under five birth defects deaths
MWI	2000	country	1_Afr	Malawi	18821	61186	80007	0.0387	0.1258114	0.1645114	486330	998	155	1153
MWI	2001	country	1_Afr	Malawi	18179	56947	75126	0.0365762	0.1145783	0.1511545	497017	1026	228	1254
MWI	2002	country	1_Afr	Malawi	17516	52280	69796	0.034427	0.1027536	0.1371806	508787	1012	222	1234
MWI	2003	country	1_Afr	Malawi	16943	47860	64803	0.0325046	0.0918188	0.1243234	521249	995	352	1347
MWI	2004	country	1_Afr	Malawi	16504	44151	60655	0.0309081	0.0826847	0.1135928	533969	1014	459	1473
MWI	2005	country	1_Afr	Malawi	16268	41374	57642	0.0297795	0.0757367	0.1055162	546281	1013	530	1543
MWI	2006	country	1_Afr	Malawi	16297	38863	55160	0.0292309	0.0697054	0.0989363	557527	1032	582	1613
MWI	2007	country	1_Afr	Malawi	16478	37842	54320	0.0290471	0.0667077	0.0957549	567285	1058	533	1591
MWI	2008	country	1_Afr	Malawi	16647	36099	52746	0.0289365	0.0627488	0.0916853	575294	1101	602	1703
MWI	2009	country	1_Afr	Malawi	16683	33978	50661	0.0286877	0.0584273	0.087115	581539	1115	683	1798
MWI	2010	country	1_Afr	Malawi	16536	33232	49768	0.0282147	0.0567016	0.0849164	586077	1132	728	1860
MWI	2011	country	1_Afr	Malawi	16127	29377	45504	0.0273727	0.0498627	0.0772354	589163	1126	857	1983
MWI	2012	country	1_Afr	Malawi	15546	26084	41630	0.026282	0.0440975	0.0703794	591508	1130	954	2084
MWI	2013	country	1_Afr	Malawi	14908	22815	37723	0.0251086	0.0384253	0.0635339	593741	1088	1030	2117
MWI	2014	country	1_Afr	Malawi	14261	19896	34157	0.0239113	0.0333587	0.05727	596412	1057	1008	2066
MWI	2015	country	1_Afr	Malawi	13695	18266	31961	0.0228209	0.0304381	0.0532589	600109	1052	1065	2117
MWI	2016	country	1_Afr	Malawi	13265	16737	30002	0.0219174	0.0276541	0.0495716	605226	1038	907	1946
MWI	2017	country	1_Afr	Malawi	12923	15518	28441	0.0211168	0.0253564	0.0464732	611978	1034	919	1953
MWI	2018	country	1_Afr	Malawi	12654	14013	26667	0.0204012	0.0225928	0.042994	620256	1010	982	1992
MWI	2019	country	1_Afr	Malawi	12469	13191	25660	0.0197908	0.0209374	0.0407282	630040	1006	956	1962
MYS	2000	country	6_Wpr	Malaysia	2501	2752	5253	0.0049223	0.0054163	0.0103385	508099	569	790	1359
MYS	2001	country	6_Wpr	Malaysia	2274	2494	4768	0.0045837	0.0050272	0.0096109	496104	528	735	1264
MYS	2002	country	6_Wpr	Malaysia	2099	2286	4385	0.004327	0.0047125	0.0090396	485090	490	691	1181
MYS	2003	country	6_Wpr	Malaysia	1987	2143	4130	0.0041714	0.0044989	0.0086703	476340	468	660	1128
MYS	2004	country	6_Wpr	Malaysia	1923	2044	3967	0.0040856	0.0043427	0.0084283	470676	464	639	1103
MYS	2005	country	6_Wpr	Malaysia	1889	1978	3867	0.0040344	0.0042244	0.0082588	468227	454	624	1078
MYS	2006	country	6_Wpr	Malaysia	1883	1934	3817	0.0040166	0.0041254	0.008142	468805	462	615	1077
MYS	2007	country	6_Wpr	Malaysia	1903	1905	3808	0.004037	0.0040412	0.0080782	471392	472	610	1081
MYS	2008	country	6_Wpr	Malaysia	1945	1883	3828	0.004091	0.0039606	0.0080517	475429	480	604	1085
MYS	2009	country	6_Wpr	Malaysia	1989	1869	3858	0.0041409	0.0038911	0.0080319	480332	494	601	1095
MYS	2010	country	6_Wpr	Malaysia	2022	1856	3878	0.0041626	0.0038209	0.0079835	485749	503	598	1101
MYS	2011	country	6_Wpr	Malaysia	2041	1853	3894	0.0041518	0.0037693	0.0079211	491599	513	597	1110
MYS	2012	country	6_Wpr	Malaysia	2053	1868	3921	0.0041242	0.0037525	0.0078767	497796	526	603	1129
MYS	2013	country	6_Wpr	Malaysia	2072	1895	3967	0.0041109	0.0037597	0.0078707	504023	535	612	1148
MYS	2014	country	6_Wpr	Malaysia	2107	1926	4033	0.0041301	0.0037753	0.0079053	510162	547	623	1170
MYS	2015	country	6_Wpr	Malaysia	2167	1945	4112	0.0042006	0.0037702	0.0079708	515883	566	631	1197
MYS	2016	country	6_Wpr	Malaysia	2246	1960	4206	0.0043113	0.0037623	0.0080735	520962	592	638	1230
MYS	2017	country	6_Wpr	Malaysia	2328	1984	4312	0.0044328	0.0037778	0.0082105	525180	611	649	1260
MYS	2018	country	6_Wpr	Malaysia	2400	2024	4424	0.0045434	0.0038316	0.0083749	528244	627	666	1293
MYS	2019	country	6_Wpr	Malaysia	2448	2065	4513	0.0046131	0.0038914	0.0085045	530659	640	684	1325

iso3	year	level	whoreg6	whoname	nnd	pnd	u5d	nmr	pnmr	u5mr	Livebirths	Neonatal birth defects deaths	1-59 month birth defects deaths	Under five birth defects deaths
NAM	2000	country	1_Afr	Namibia	1248	2914	4162	0.0226038	0.0527857	0.0753895	55212	90	29	119
NAM	2001	country	1_Afr	Namibia	1215	2901	4116	0.0221096	0.0527819	0.0748915	54954	91	21	111
NAM	2002	country	1_Afr	Namibia	1188	2932	4120	0.0216025	0.053307	0.0749095	54994	90	27	117
NAM	2003	country	1_Afr	Namibia	1169	2925	4094	0.0211198	0.0528373	0.0739571	55351	87	29	117
NAM	2004	country	1_Afr	Namibia	1158	2902	4060	0.0206619	0.0517719	0.0724338	56045	90	28	118
NAM	2005	country	1_Afr	Namibia	1157	2745	3902	0.0202677	0.0480811	0.0683488	57086	89	35	123
NAM	2006	country	1_Afr	Namibia	1166	2543	3709	0.0199595	0.0435224	0.063482	58418	92	30	123
NAM	2007	country	1_Afr	Namibia	1184	2325	3509	0.0197667	0.0388193	0.0585861	59899	94	42	136
NAM	2008	country	1_Afr	Namibia	1214	2102	3316	0.0197427	0.0341821	0.0539248	61491	97	57	154
NAM	2009	country	1_Afr	Namibia	1253	1955	3208	0.0198729	0.0310011	0.050874	63051	102	70	173
NAM	2010	country	1_Afr	Namibia	1295	1845	3140	0.0200733	0.0286015	0.0486748	64514	105	78	183
NAM	2011	country	1_Afr	Namibia	1334	1932	3266	0.0202755	0.0293694	0.0496449	65794	108	82	190
NAM	2012	country	1_Afr	Namibia	1367	2090	3457	0.0204163	0.0312086	0.0516249	66956	111	87	198
NAM	2013	country	1_Afr	Namibia	1391	1926	3317	0.0204737	0.0283458	0.0488195	67941	115	105	220
NAM	2014	country	1_Afr	Namibia	1401	1835	3236	0.0203936	0.0267145	0.0471081	68698	116	110	226
NAM	2015	country	1_Afr	Namibia	1403	1854	3257	0.0202369	0.0267382	0.0469751	69329	116	129	245
NAM	2016	country	1_Afr	Namibia	1398	1788	3186	0.0200556	0.0256572	0.0457128	69706	117	104	221
NAM	2017	country	1_Afr	Namibia	1384	1696	3080	0.0197794	0.0242383	0.0440177	69972	119	111	230
NAM	2018	country	1_Afr	Namibia	1364	1644	3008	0.0194514	0.0234428	0.0428942	70124	119	116	235
NAM	2019	country	1_Afr	Namibia	1343	1610	2953	0.0191207	0.0229216	0.0420424	70238	119	118	237
NER	2000	country	1_Afr	Niger	26194	101783	127977	0.0430647	0.1673382	0.2104029	608247	900	52	952
NER	2001	country	1_Afr	Niger	26180	112445	138625	0.0417393	0.1792728	0.221012	627227	930	66	996
NER	2002	country	1_Afr	Niger	26094	102617	128711	0.0403525	0.1586905	0.199043	646651	958	90	1049
NER	2003	country	1_Afr	Niger	25988	105685	131673	0.0389899	0.1585602	0.19755	666532	988	116	1104
NER	2004	country	1_Afr	Niger	25879	105139	131018	0.0376752	0.1530641	0.1907393	686898	1018	146	1165
NER	2005	country	1_Afr	Niger	25793	84744	110537	0.0364398	0.1197238	0.1561635	707826	1051	179	1230
NER	2006	country	1_Afr	Niger	25664	81712	107376	0.0351832	0.1120206	0.1472039	729438	1084	242	1327
NER	2007	country	1_Afr	Niger	25525	78521	104046	0.0339546	0.1044526	0.1384071	751740	1117	217	1334
NER	2008	country	1_Afr	Niger	25328	75652	100980	0.03269	0.0976414	0.1303314	774793	1146	408	1554
NER	2009	country	1_Afr	Niger	25176	71722	96898	0.0315251	0.0898097	0.1213348	798601	1177	462	1639
NER	2010	country	1_Afr	Niger	25077	68968	94045	0.0304682	0.0837949	0.1142631	823055	1210	448	1658
NER	2011	country	1_Afr	Niger	24992	66070	91062	0.0294711	0.0779112	0.1073824	848017	1244	333	1577
NER	2012	country	1_Afr	Niger	24988	64152	89140	0.02861	0.0734514	0.1020614	873401	1277	405	1682
NER	2013	country	1_Afr	Niger	25064	61981	87045	0.0278735	0.068929	0.0968025	899204	1314	499	1813
NER	2014	country	1_Afr	Niger	25111	60995	86106	0.0271365	0.0659153	0.0930518	925358	1344	574	1918
NER	2015	country	1_Afr	Niger	25253	60805	86058	0.0265254	0.0638682	0.0903936	952032	1380	1150	2530
NER	2016	country	1_Afr	Niger	25385	58584	83969	0.0259211	0.0598208	0.0857419	979320	1416	740	2155
NER	2017	country	1_Afr	Niger	25530	57692	83222	0.025343	0.0572699	0.0826129	1007377	1456	1458	2914
NER	2018	country	1_Afr	Niger	25686	57424	83110	0.0247862	0.0554127	0.0801989	1036301	1490	982	2472
NER	2019	country	1_Afr	Niger	25861	57709	83570	0.0242569	0.0541293	0.0783862	1066132	1522	1008	2530

iso3	year	level	whoreg6	whoname	nnd	pnd	u5d	nmr	pnmr	u5mr	Livebirths	Neonatal birth defects deaths	1-59 month birth defects deaths	Under five birth defects deaths
NGA	2000	country	1_Afr	Nigeria	244605	774479	1019084	0.0463138	0.1466406	0.1929543	5281475	8543	591	9134
NGA	2001	country	1_Afr	Nigeria	244322	742392	986714	0.0451826	0.1372911	0.1824738	5407431	8697	430	9127
NGA	2002	country	1_Afr	Nigeria	243391	672278	915669	0.0439792	0.1214764	0.1654556	5534227	8835	475	9309
NGA	2003	country	1_Afr	Nigeria	242293	760923	1003216	0.0427986	0.1344093	0.1772078	5661240	8966	904	9870
NGA	2004	country	1_Afr	Nigeria	241209	660162	901371	0.041674	0.1140571	0.1557311	5788000	9092	1068	10160
NGA	2005	country	1_Afr	Nigeria	240743	717310	958053	0.0407015	0.1212728	0.1619742	5914850	9228	1264	10492
NGA	2006	country	1_Afr	Nigeria	240884	611254	852138	0.0398644	0.1011576	0.141022	6042590	9375	967	10342
NGA	2007	country	1_Afr	Nigeria	241863	602159	844022	0.0391889	0.0975674	0.1367563	6171727	9539	973	10513
NGA	2008	country	1_Afr	Nigeria	243556	606220	849776	0.0386468	0.0961933	0.1348401	6302104	9721	2207	11928
NGA	2009	country	1_Afr	Nigeria	246103	593047	839150	0.0382573	0.0921907	0.130448	6432833	9931	3268	13199
NGA	2010	country	1_Afr	Nigeria	249215	600918	850133	0.0379788	0.091576	0.1295548	6561954	10164	2984	13148
NGA	2011	country	1_Afr	Nigeria	252363	608947	861310	0.0377382	0.0910615	0.1287997	6687205	10393	1880	12273
NGA	2012	country	1_Afr	Nigeria	255666	598974	854640	0.0375589	0.087993	0.1255519	6807069	10622	1755	12377
NGA	2013	country	1_Afr	Nigeria	258745	638219	896964	0.0373868	0.0922181	0.1296049	6920752	10833	1917	12749
NGA	2014	country	1_Afr	Nigeria	261842	606468	868310	0.0372546	0.0862876	0.1235421	7028451	11048	2186	13233
NGA	2015	country	1_Afr	Nigeria	264817	618048	882865	0.037136	0.0866705	0.1238065	7131009	11268	2380	13649
NGA	2016	country	1_Afr	Nigeria	266983	615089	882072	0.036927	0.0850743	0.1220013	7230020	11478	4345	15823
NGA	2017	country	1_Afr	Nigeria	268731	608313	877044	0.0366721	0.0830128	0.1196849	7327944	11679	6447	18126
NGA	2018	country	1_Afr	Nigeria	269615	594506	864121	0.0363023	0.0800473	0.1163496	7426939	11823	4226	16050
NGA	2019	country	1_Afr	Nigeria	269897	608580	878477	0.0358507	0.0808382	0.1166888	7528368	11917	4328	16245
NIC	2000	country	2_Amr	Nicaragua	2257	3029	5286	0.0164616	0.0220922	0.0385538	137107	470	303	773
NIC	2001	country	2_Amr	Nicaragua	2169	2805	4974	0.0158983	0.02056	0.0364583	136430	452	300	751
NIC	2002	country	2_Amr	Nicaragua	2095	2603	4698	0.0154078	0.0191439	0.0345517	135970	436	301	737
NIC	2003	country	2_Amr	Nicaragua	2039	2416	4455	0.0150186	0.0177955	0.0328141	135765	424	306	730
NIC	2004	country	2_Amr	Nicaragua	2007	2234	4241	0.0147829	0.0164549	0.0312377	135765	418	313	731
NIC	2005	country	2_Amr	Nicaragua	1990	2053	4043	0.0146368	0.0151002	0.029737	135959	414	318	732
NIC	2006	country	2_Amr	Nicaragua	1970	1893	3863	0.0144632	0.0138979	0.0283612	136207	410	323	733
NIC	2007	country	2_Amr	Nicaragua	1940	1760	3700	0.014209	0.0128907	0.0270997	136533	404	325	730
NIC	2008	country	2_Amr	Nicaragua	1914	1631	3545	0.0139865	0.0119185	0.0259051	136846	399	322	721
NIC	2009	country	2_Amr	Nicaragua	1888	1514	3402	0.0137693	0.0110417	0.024811	137117	392	315	708
NIC	2010	country	2_Amr	Nicaragua	1860	1404	3264	0.0135503	0.0102283	0.0237786	137266	389	305	694
NIC	2011	country	2_Amr	Nicaragua	1833	1299	3132	0.0133477	0.0094591	0.0228068	137328	381	291	672
NIC	2012	country	2_Amr	Nicaragua	1800	1206	3006	0.0131211	0.0087912	0.0219123	137183	371	277	648
NIC	2013	country	2_Amr	Nicaragua	1757	1126	2883	0.012827	0.0082204	0.0210474	136977	375	264	639
NIC	2014	country	2_Amr	Nicaragua	1696	1068	2764	0.0124149	0.0078179	0.0202328	136610	346	231	577
NIC	2015	country	2_Amr	Nicaragua	1624	1020	2644	0.0119368	0.0074972	0.019434	136050	326	272	599
NIC	2016	country	2_Amr	Nicaragua	1551	979	2530	0.0114576	0.0072321	0.0186898	135368	364	253	617
NIC	2017	country	2_Amr	Nicaragua	1482	939	2421	0.011017	0.0069804	0.0179973	134520	357	217	575
NIC	2018	country	2_Amr	Nicaragua	1416	899	2315	0.0106092	0.0067357	0.0173449	133468	320	227	546
NIC	2019	country	2_Amr	Nicaragua	1352	860	2212	0.0102195	0.0065006	0.0167201	132296	316	217	533

iso3	year	level	whoreg6	whoname	nnd	pnd	u5d	nmr	pnmr	u5mr	Livebirths	Neonatal birth defects deaths	1-59 month birth defects deaths	Under five birth defects deaths
NIU	2000	country	6_Wpr	Niue	0	1	1				0	0	0	0
NIU	2001	country	6_Wpr	Niue	0	1	1				0	0	0	0
NIU	2002	country	6_Wpr	Niue	0	1	1				0	0	0	0
NIU	2003	country	6_Wpr	Niue	0	1	1				0	0	0	0
NIU	2004	country	6_Wpr	Niue	0	1	1				0	0	0	0
NIU	2005	country	6_Wpr	Niue	1	0	1	0.0163638	0	0.0163638	61	0	0	0
NIU	2006	country	6_Wpr	Niue	0	1	1				0	0	0	0
NIU	2007	country	6_Wpr	Niue	0	1	1				0	0	0	0
NIU	2008	country	6_Wpr	Niue	0	1	1				0	0	0	0
NIU	2009	country	6_Wpr	Niue	0	1	1				0	0	0	0
NIU	2010	country	6_Wpr	Niue	0	1	1				0	0	0	0
NIU	2011	country	6_Wpr	Niue	0	1	1				0	0	0	0
NIU	2012	country	6_Wpr	Niue	0	1	1				0	0	0	0
NIU	2013	country	6_Wpr	Niue	0	1	1				0	0	0	0
NIU	2014	country	6_Wpr	Niue	0	1	1				0	0	0	0
NIU	2015	country	6_Wpr	Niue	0	1	1				0	0	0	0
NIU	2016	country	6_Wpr	Niue	0	1	1				0	0	0	0
NIU	2017	country	6_Wpr	Niue	0	1	1				0	0	0	0
NIU	2018	country	6_Wpr	Niue	0	1	1				0	0	0	0
NIU	2019	country	6_Wpr	Niue	0	1	1				0	0	0	0
NLD	2000	country	4_Eur	Netherlands	756	461	1217	0.0038284	0.0023345	0.006163	197470	311	125	436
NLD	2001	country	4_Eur	Netherlands	753	442	1195	0.0038023	0.0022319	0.0060342	198039	290	139	429
NLD	2002	country	4_Eur	Netherlands	739	428	1167	0.0037292	0.0021598	0.0058891	198164	273	119	392
NLD	2003	country	4_Eur	Netherlands	718	412	1130	0.0036358	0.0020863	0.005722	197483	278	141	419
NLD	2004	country	4_Eur	Netherlands	695	392	1087	0.0035465	0.0020003	0.0055468	195970	262	126	388
NLD	2005	country	4_Eur	Netherlands	666	372	1038	0.0034345	0.0019184	0.0053529	193912	219	105	325
NLD	2006	country	4_Eur	Netherlands	631	356	987	0.0032997	0.0018616	0.0051613	191232	245	106	351
NLD	2007	country	4_Eur	Netherlands	593	343	936	0.0031432	0.0018181	0.0049612	188662	175	104	280
NLD	2008	country	4_Eur	Netherlands	558	331	889	0.0030006	0.0017799	0.0047806	185961	183	88	271
NLD	2009	country	4_Eur	Netherlands	528	319	847	0.0028764	0.0017378	0.0046143	183562	181	81	262
NLD	2010	country	4_Eur	Netherlands	506	305	811	0.0027847	0.0016785	0.0044632	181708	180	90	270
NLD	2011	country	4_Eur	Netherlands	491	290	781	0.0027321	0.0016137	0.0043458	179715	178	84	262
NLD	2012	country	4_Eur	Netherlands	481	275	756	0.0027012	0.0015444	0.0042456	178066	147	78	225
NLD	2013	country	4_Eur	Netherlands	472	264	736	0.0026739	0.0014956	0.0041695	176519	147	62	210
NLD	2014	country	4_Eur	Netherlands	464	256	720	0.0026485	0.0014612	0.0041097	175194	138	68	205
NLD	2015	country	4_Eur	Netherlands	457	251	708	0.0026276	0.0014432	0.0040707	173924	134	70	204
NLD	2016	country	4_Eur	Netherlands	452	248	700	0.0026111	0.0014327	0.0040438	173105	126	70	196
NLD	2017	country	4_Eur	Netherlands	449	247	696	0.0026061	0.0014337	0.0040398	172285	130	68	198
NLD	2018	country	4_Eur	Netherlands	447	248	695	0.0025943	0.0014393	0.0040336	172304	128	68	197
NLD	2019	country	4_Eur	Netherlands	446	250	696	0.002587	0.0014501	0.0040371	172400	127	69	196

iso3	year	level	whoreg6	whoname	nnd	pnd	u5d	nmr	pnmr	u5mr	Livebirths	Neonatal birth defects deaths	1-59 month birth defects deaths	Under five birth defects deaths
NOR	2000	country	4_Eur	Norway	155	128	283	0.0026873	0.0022192	0.0049065	57679	61	44	105
NOR	2001	country	4_Eur	Norway	149	124	273	0.002604	0.0021671	0.0047711	57219	56	45	101
NOR	2002	country	4_Eur	Norway	144	118	262	0.0025179	0.0020633	0.0045811	57191	55	35	90
NOR	2003	country	4_Eur	Norway	139	113	252	0.002424	0.0019706	0.0043946	57343	40	30	71
NOR	2004	country	4_Eur	Norway	135	107	242	0.0023419	0.0018561	0.004198	57647	57	41	98
NOR	2005	country	4_Eur	Norway	131	103	234	0.0022511	0.0017699	0.004021	58194	36	34	70
NOR	2006	country	4_Eur	Norway	128	98	226	0.0021718	0.0016628	0.0038345	58938	37	28	65
NOR	2007	country	4_Eur	Norway	125	94	219	0.0021026	0.0015811	0.0036837	59451	37	25	62
NOR	2008	country	4_Eur	Norway	121	91	212	0.0020311	0.0015275	0.0035586	59574	37	29	66
NOR	2009	country	4_Eur	Norway	117	87	204	0.0019445	0.0014459	0.0033905	60168	35	25	61
NOR	2010	country	4_Eur	Norway	112	84	196	0.001864	0.001398	0.0032619	60087	38	28	66
NOR	2011	country	4_Eur	Norway	108	80	188	0.0017961	0.0013304	0.0031266	60130	38	27	65
NOR	2012	country	4_Eur	Norway	104	76	180	0.0017407	0.001272	0.0030127	59747	35	19	54
NOR	2013	country	4_Eur	Norway	101	72	173	0.0016909	0.0012054	0.0028964	59730	28	27	55
NOR	2014	country	4_Eur	Norway	97	70	167	0.0016414	0.0011845	0.0028259	59097	29	15	44
NOR	2015	country	4_Eur	Norway	94	67	161	0.0015908	0.0011339	0.0027248	59088	35	21	56
NOR	2016	country	4_Eur	Norway	91	65	156	0.0015451	0.0011036	0.0026487	58897	29	22	51
NOR	2017	country	4_Eur	Norway	88	64	152	0.0014868	0.0010813	0.002568	59189	29	18	47
NOR	2018	country	4_Eur	Norway	86	62	148	0.0014497	0.0010452	0.0024949	59321	29	18	47
NOR	2019	country	4_Eur	Norway	83	62	145	0.001398	0.0010443	0.0024424	59369	27	18	45
NPL	2000	country	3_Sear	Nepal	30395	32054	62449	0.0403709	0.0425741	0.0829449	752894	1779	517	2295
NPL	2001	country	3_Sear	Nepal	28702	31104	59806	0.0388884	0.0421427	0.081031	738062	1755	526	2282
NPL	2002	country	3_Sear	Nepal	27002	28035	55037	0.0374269	0.0388586	0.0762855	721459	1728	531	2258
NPL	2003	country	3_Sear	Nepal	25338	28226	53564	0.035981	0.0400818	0.0760628	704204	1694	582	2277
NPL	2004	country	3_Sear	Nepal	23802	26692	50494	0.0346411	0.0388473	0.0734883	687104	1662	548	2210
NPL	2005	country	3_Sear	Nepal	22336	20064	42400	0.0332969	0.0299103	0.0632072	670813	1627	549	2176
NPL	2006	country	3_Sear	Nepal	20995	17401	38396	0.032016	0.0265361	0.0585521	655765	1596	693	2289
NPL	2007	country	3_Sear	Nepal	19779	15997	35776	0.030814	0.0249216	0.0557356	641884	1568	611	2180
NPL	2008	country	3_Sear	Nepal	18642	14857	33499	0.0296315	0.0236146	0.0532461	629127	1543	565	2108
NPL	2009	country	3_Sear	Nepal	17588	13112	30700	0.0284771	0.0212302	0.0497073	617619	1520	711	2231
NPL	2010	country	3_Sear	Nepal	16603	12131	28734	0.0273313	0.0199697	0.047301	607471	1498	641	2139
NPL	2011	country	3_Sear	Nepal	15694	11408	27102	0.0262211	0.0190598	0.0452809	598526	1480	669	2148
NPL	2012	country	3_Sear	Nepal	14861	10875	25736	0.0251631	0.0184145	0.0435776	590588	1463	630	2092
NPL	2013	country	3_Sear	Nepal	14109	9179	23288	0.0241796	0.0157304	0.03991	583508	1448	639	2087
NPL	2014	country	3_Sear	Nepal	13429	8502	21931	0.0232666	0.0147303	0.0379969	577180	1434	613	2048
NPL	2015	country	3_Sear	Nepal	12827	7945	20772	0.0224325	0.0138943	0.0363268	571806	1425	555	1980
NPL	2016	country	3_Sear	Nepal	12299	7429	19728	0.0216644	0.0130859	0.0347503	567706	1418	527	1945
NPL	2017	country	3_Sear	Nepal	11847	6918	18765	0.0209684	0.0122437	0.0332121	564992	1415	573	1988
NPL	2018	country	3_Sear	Nepal	11470	6515	17985	0.0203591	0.0115642	0.0319233	563385	1407	543	1950
NPL	2019	country	3_Sear	Nepal	11128	6176	17304	0.0197799	0.0109772	0.0307572	562590	1393	536	1930

iso3	year	level	whoreg6	whoname	nnd	pnd	u5d	nmr	pnmr	u5mr	Livebirths	Neonatal birth defects deaths	1-59 month birth defects deaths	Under five birth defects deaths
NRU	2000	country	6_Wpr	Nauru	8	6	14	0.0244658	0.0183493	0.0428151	327	1	0	1
NRU	2001	country	6_Wpr	Nauru	8	5	13	0.02421	0.0151313	0.0393413	330	1	1	2
NRU	2002	country	6_Wpr	Nauru	8	5	13	0.0240363	0.0150227	0.039059	333	1	0	1
NRU	2003	country	6_Wpr	Nauru	7	5	12	0.02394	0.0171	0.04104	292	1	0	1
NRU	2004	country	6_Wpr	Nauru	7	5	12	0.0239144	0.0170817	0.0409962	293	1	0	1
NRU	2005	country	6_Wpr	Nauru	7	5	12	0.0239548	0.0171106	0.0410654	292	1	0	1
NRU	2006	country	6_Wpr	Nauru	7	5	12	0.0240389	0.0171707	0.0412096	291	1	1	1
NRU	2007	country	6_Wpr	Nauru	7	5	12	0.0240998	0.0172142	0.041314	290	1	1	1
NRU	2008	country	6_Wpr	Nauru	8	4	12	0.0241407	0.0120703	0.036211	331	1	1	1
NRU	2009	country	6_Wpr	Nauru	8	4	12	0.024129	0.0120645	0.0361935	332	1	1	1
NRU	2010	country	6_Wpr	Nauru	8	4	12	0.0239343	0.0119672	0.0359015	334	1	1	1
NRU	2011	country	6_Wpr	Nauru	8	4	12	0.0236756	0.0118378	0.0355134	338	1	1	2
NRU	2012	country	6_Wpr	Nauru	7	5	12	0.0233334	0.0166667	0.0400001	300	1	1	2
NRU	2013	country	6_Wpr	Nauru	7	5	12	0.0229373	0.0163838	0.039321	305	1	1	2
NRU	2014	country	6_Wpr	Nauru	7	4	11	0.0224587	0.0128336	0.0352923	312	1	1	1
NRU	2015	country	6_Wpr	Nauru	7	4	11	0.0220226	0.0125844	0.034607	318	1	1	1
NRU	2016	country	6_Wpr	Nauru	7	4	11	0.0215023	0.0122871	0.0337894	326	1	1	1
NRU	2017	country	6_Wpr	Nauru	6	4	10	0.0210261	0.0140174	0.0350435	285	1	1	1
NRU	2018	country	6_Wpr	Nauru	6	4	10	0.0204467	0.0136311	0.0340778	293	1	1	1
NRU	2019	country	6_Wpr	Nauru	6	3	9	0.0199415	0.0099708	0.0299123	301	1	0	1
NZL	2000	country	6_Wpr	New Zealand	195	218	413	0.0034885	0.0039	0.0073885	55897	60	59	119
NZL	2001	country	6_Wpr	New Zealand	196	207	403	0.0034864	0.003682	0.0071684	56219	70	43	113
NZL	2002	country	6_Wpr	New Zealand	197	199	396	0.0034741	0.0035094	0.0069835	56705	53	53	107
NZL	2003	country	6_Wpr	New Zealand	195	197	392	0.003375	0.0034096	0.0067846	57778	48	67	114
NZL	2004	country	6_Wpr	New Zealand	192	198	390	0.0032488	0.0033503	0.0065991	59099	48	60	108
NZL	2005	country	6_Wpr	New Zealand	188	203	391	0.0031242	0.0033735	0.0064977	60176	52	45	97
NZL	2006	country	6_Wpr	New Zealand	183	209	392	0.002982	0.0034057	0.0063878	61367	61	54	115
NZL	2007	country	6_Wpr	New Zealand	181	213	394	0.0028942	0.0034059	0.0063	62539	54	48	103
NZL	2008	country	6_Wpr	New Zealand	186	208	394	0.0029527	0.0033019	0.0062546	62994	53	43	97
NZL	2009	country	6_Wpr	New Zealand	196	196	392	0.0030975	0.0030975	0.006195	63277	53	47	101
NZL	2010	country	6_Wpr	New Zealand	205	182	387	0.0032403	0.0028768	0.0061171	63265	58	35	93
NZL	2011	country	6_Wpr	New Zealand	209	171	380	0.0033334	0.0027273	0.0060607	62699	62	42	104
NZL	2012	country	6_Wpr	New Zealand	208	162	370	0.0033385	0.0026002	0.0059387	62303	64	33	97
NZL	2013	country	6_Wpr	New Zealand	204	154	358	0.0033232	0.0025087	0.0058319	61386	64	27	91
NZL	2014	country	6_Wpr	New Zealand	197	149	346	0.0032257	0.0024397	0.0056654	61072	57	20	77
NZL	2015	country	6_Wpr	New Zealand	189	143	332	0.0031305	0.0023686	0.0054991	60374	57	25	82
NZL	2016	country	6_Wpr	New Zealand	180	138	318	0.0029886	0.0022912	0.0052798	60229	54	24	78
NZL	2017	country	6_Wpr	New Zealand	171	134	305	0.0028641	0.0022444	0.0051085	59705	51	23	74
NZL	2018	country	6_Wpr	New Zealand	163	131	294	0.002732	0.0021957	0.0049277	59663	49	23	71
NZL	2019	country	6_Wpr	New Zealand	157	126	283	0.0026374	0.0021167	0.0047541	59528	47	22	69

iso3	year	level	whoreg6	whoname	nnd	pnd	u5d	nmr	pnmr	u5mr	Livebirths	Neonatal birth defects deaths	1-59 month birth defects deaths	Under five birth defects deaths
OMN	2000	country	5_Emr	Oman	421	511	932	0.0075534	0.0091681	0.0167215	55737	106	176	281
OMN	2001	country	5_Emr	Oman	387	468	855	0.0070906	0.0085747	0.0156653	54579	101	165	265
OMN	2002	country	5_Emr	Oman	361	432	793	0.0066943	0.0080109	0.0147052	53927	96	155	251
OMN	2003	country	5_Emr	Oman	343	405	748	0.0063737	0.0075258	0.0138994	53815	91	148	239
OMN	2004	country	5_Emr	Oman	332	385	717	0.0061285	0.0071068	0.0132353	54173	92	142	234
OMN	2005	country	5_Emr	Oman	327	374	701	0.0059075	0.0067566	0.0126641	55353	90	140	230
OMN	2006	country	5_Emr	Oman	329	368	697	0.0057676	0.0064514	0.012219	57042	89	139	228
OMN	2007	country	5_Emr	Oman	335	370	705	0.0056348	0.0062235	0.0118582	59452	93	141	234
OMN	2008	country	5_Emr	Oman	345	376	721	0.0055551	0.0060542	0.0116093	62105	95	144	238
OMN	2009	country	5_Emr	Oman	357	388	745	0.0054717	0.0059468	0.0114185	65245	96	150	245
OMN	2010	country	5_Emr	Oman	371	401	772	0.0053971	0.0058335	0.0112306	68741	100	156	256
OMN	2011	country	5_Emr	Oman	386	417	803	0.0053381	0.0057668	0.0111049	72310	103	164	267
OMN	2012	country	5_Emr	Oman	402	433	835	0.0052759	0.0056828	0.0109587	76195	106	171	277
OMN	2013	country	5_Emr	Oman	417	452	869	0.0052144	0.0056521	0.0108664	79971	109	179	288
OMN	2014	country	5_Emr	Oman	433	470	903	0.0051839	0.0056268	0.0108107	83528	111	187	298
OMN	2015	country	5_Emr	Oman	448	492	940	0.005171	0.0056789	0.0108499	86637	115	196	311
OMN	2016	country	5_Emr	Oman	462	514	976	0.0051858	0.0057695	0.0109554	89089	118	205	322
OMN	2017	country	5_Emr	Oman	472	534	1006	0.005216	0.0059011	0.0111171	90491	121	213	334
OMN	2018	country	5_Emr	Oman	478	549	1027	0.005246	0.0060252	0.0112711	91118	124	219	343
OMN	2019	country	5_Emr	Oman	477	557	1034	0.0052435	0.0061229	0.0113664	90970	123	222	344
PAK	2000	country	5_Emr	Pakistan	280411	239835	520246	0.0569059	0.0486715	0.1055774	4927630	14546	6355	20900
PAK	2001	country	5_Emr	Pakistan	276845	234325	511170	0.0558695	0.0472886	0.1031581	4955205	14838	6834	21672
PAK	2002	country	5_Emr	Pakistan	273833	228503	502336	0.0549381	0.0458436	0.1007818	4984388	15166	7139	22305
PAK	2003	country	5_Emr	Pakistan	271289	224073	495362	0.0540792	0.0446671	0.0987463	5016512	15513	7613	23126
PAK	2004	country	5_Emr	Pakistan	269379	218005	487384	0.0533103	0.0431434	0.0964537	5053035	15885	7931	23816
PAK	2005	country	5_Emr	Pakistan	268446	212800	481246	0.0526849	0.041764	0.0944489	5095309	16300	7618	23917
PAK	2006	country	5_Emr	Pakistan	267803	208348	476151	0.0520592	0.0405014	0.0925606	5144204	16735	7341	24076
PAK	2007	country	5_Emr	Pakistan	267951	204455	472406	0.0515361	0.0393237	0.0908598	5199291	17225	7093	24318
PAK	2008	country	5_Emr	Pakistan	268460	198642	467102	0.0510387	0.0377652	0.088804	5259928	17770	7055	24825
PAK	2009	country	5_Emr	Pakistan	269195	193223	462418	0.0505459	0.0362809	0.0868268	5325753	18345	7224	25569
PAK	2010	country	5_Emr	Pakistan	269460	192758	462218	0.0499242	0.0357132	0.0856375	5397377	18892	7535	26427
PAK	2011	country	5_Emr	Pakistan	268904	188159	457063	0.0491101	0.0343637	0.0834737	5475537	19384	8043	27427
PAK	2012	country	5_Emr	Pakistan	267877	182328	450205	0.0481866	0.0327977	0.0809842	5559164	19878	8953	28831
PAK	2013	country	5_Emr	Pakistan	266653	183318	449971	0.0472298	0.0324694	0.0796991	5645865	20399	10887	31286
PAK	2014	country	5_Emr	Pakistan	265067	172472	437539	0.046239	0.0300864	0.0763254	5732545	20945	11803	32748
PAK	2015	country	5_Emr	Pakistan	262925	167589	430514	0.0452175	0.0288218	0.0740392	5814680	21476	13073	34549
PAK	2016	country	5_Emr	Pakistan	260351	164287	424638	0.0442208	0.0279042	0.072125	5887530	21993	13164	35157
PAK	2017	country	5_Emr	Pakistan	256984	160584	417568	0.0432054	0.0269981	0.0702034	5947964	22415	13524	35939
PAK	2018	country	5_Emr	Pakistan	252868	166195	419063	0.0421862	0.0277264	0.0699126	5994098	22598	13711	36309
PAK	2019	country	5_Emr	Pakistan	248342	150176	398518	0.0412171	0.0249246	0.0661416	6025224	22605	13546	36150

iso3	year	level	whoreg6	whoname	nnd	pnd	u5d	nmr	pnmr	u5mr	Livebirths	Neonatal birth defects deaths	1-59 month birth defects deaths	Under five birth defects deaths
PAN	2000	country	2_Amr	Panama	1080	746	1826	0.015198	0.0104979	0.0256959	71062	310	191	501
PAN	2001	country	2_Amr	Panama	1062	735	1797	0.0148579	0.010283	0.0251409	71477	262	200	461
PAN	2002	country	2_Amr	Panama	1040	723	1763	0.0144602	0.0100526	0.0245127	71922	272	239	511
PAN	2003	country	2_Amr	Panama	1019	710	1729	0.0140836	0.0098129	0.0238965	72354	293	210	503
PAN	2004	country	2_Amr	Panama	998	699	1697	0.013704	0.0095983	0.0233023	72825	303	185	488
PAN	2005	country	2_Amr	Panama	981	684	1665	0.0133605	0.0093156	0.0226761	73425	297	118	414
PAN	2006	country	2_Amr	Panama	964	671	1635	0.0130107	0.0090562	0.0220669	74093	239	195	434
PAN	2007	country	2_Amr	Panama	944	661	1605	0.0126189	0.0088359	0.0214548	74808	263	126	388
PAN	2008	country	2_Amr	Panama	925	648	1573	0.0122572	0.0085866	0.0208438	75466	285	162	447
PAN	2009	country	2_Amr	Panama	905	639	1544	0.01187	0.0083811	0.0202511	76243	247	136	383
PAN	2010	country	2_Amr	Panama	883	631	1514	0.0114814	0.0082047	0.0196861	76907	272	112	384
PAN	2011	country	2_Amr	Panama	862	619	1481	0.0111179	0.0079838	0.0191017	77532	244	149	393
PAN	2012	country	2_Amr	Panama	841	603	1444	0.0107759	0.0077264	0.0185023	78045	231	161	391
PAN	2013	country	2_Amr	Panama	820	590	1410	0.0104511	0.0075197	0.0179708	78461	191	146	337
PAN	2014	country	2_Amr	Panama	796	575	1371	0.0101205	0.0073107	0.0174311	78652	194	188	382
PAN	2015	country	2_Amr	Panama	773	556	1329	0.0097963	0.0070463	0.0168426	78907	233	157	390
PAN	2016	country	2_Amr	Panama	750	542	1292	0.0094907	0.0068586	0.0163494	79025	210	167	377
PAN	2017	country	2_Amr	Panama	723	530	1253	0.0091395	0.0066998	0.0158392	79107	199	162	361
PAN	2018	country	2_Amr	Panama	702	508	1210	0.0088644	0.0064147	0.0152791	79193	201	155	356
PAN	2019	country	2_Amr	Panama	680	495	1175	0.0085703	0.0062387	0.014809	79344	191	151	342
PER	2000	country	2_Amr	Peru	9793	13966	23759	0.0159252	0.0227113	0.0386365	614937	1196	2461	3657
PER	2001	country	2_Amr	Peru	9220	12590	21810	0.0150519	0.0205536	0.0356055	612546	1194	2303	3497
PER	2002	country	2_Amr	Peru	8780	11369	20149	0.0143402	0.0185688	0.032909	612264	1220	1840	3059
PER	2003	country	2_Amr	Peru	8438	10285	18723	0.0137501	0.0167598	0.0305099	613670	1248	1507	2754
PER	2004	country	2_Amr	Peru	8105	9405	17510	0.0131518	0.0152613	0.0284132	616264	1259	1171	2430
PER	2005	country	2_Amr	Peru	7680	8762	16442	0.0124102	0.0141586	0.0265689	618845	1272	1047	2319
PER	2006	country	2_Amr	Peru	7276	8193	15469	0.0117367	0.0132159	0.0249527	619934	1284	1035	2319
PER	2007	country	2_Amr	Peru	6875	7683	14558	0.0111127	0.0124187	0.0235313	618664	1272	1053	2325
PER	2008	country	2_Amr	Peru	6480	7201	13681	0.0105438	0.011717	0.0222608	614577	1241	1068	2309
PER	2009	country	2_Amr	Peru	6098	6739	12837	0.0100342	0.011089	0.0211232	607719	1205	1073	2278
PER	2010	country	2_Amr	Peru	5728	6303	12031	0.0095635	0.0105235	0.020087	598943	1158	1072	2229
PER	2011	country	2_Amr	Peru	5379	5895	11274	0.0091239	0.0099991	0.019123	589553	1145	1062	2207
PER	2012	country	2_Amr	Peru	5062	5526	10588	0.0087106	0.009509	0.0182195	581134	1087	1049	2137
PER	2013	country	2_Amr	Peru	4777	5201	9978	0.0083122	0.00905	0.0173622	574698	1013	1034	2047
PER	2014	country	2_Amr	Peru	4529	4907	9436	0.0079352	0.0085975	0.0165326	570750	995	1022	2017
PER	2015	country	2_Amr	Peru	4311	4646	8957	0.0075722	0.0081607	0.0157329	569316	955	1010	1965
PER	2016	country	2_Amr	Peru	4121	4415	8536	0.0072316	0.0077475	0.0149792	569858	939	1000	1939
PER	2017	country	2_Amr	Peru	3952	4226	8178	0.0069139	0.0073933	0.0143072	571601	880	987	1867
PER	2018	country	2_Amr	Peru	3796	4055	7851	0.0066213	0.007073	0.0136943	573304	853	977	1830
PER	2019	country	2_Amr	Peru	3653	3910	7563	0.0063578	0.0068051	0.0131628	574573	837	973	1810

iso3	year	level	whoreg6	whoname	nnd	pnd	u5d	nmr	pnmr	u5mr	Livebirths	Neonatal birth defects deaths	1-59 month birth defects deaths	Under five birth defects deaths
PHL	2000	country	6_Wpr	Philippines	37714	47609	85323	0.016317	0.0205981	0.0369151	2311332	3896	3980	7876
PHL	2001	country	6_Wpr	Philippines	37852	46965	84817	0.0162195	0.0201244	0.0363439	2333730	3980	4225	8205
PHL	2002	country	6_Wpr	Philippines	37863	46440	84303	0.0161147	0.0197651	0.0358798	2349594	4017	4288	8306
PHL	2003	country	6_Wpr	Philippines	37692	45905	83597	0.0159823	0.0194649	0.0354473	2358353	4095	4731	8826
PHL	2004	country	6_Wpr	Philippines	37415	44791	82206	0.0158512	0.0189763	0.0348275	2360386	4157	5187	9344
PHL	2005	country	6_Wpr	Philippines	36991	43245	80236	0.0156888	0.0183411	0.0340299	2357796	4227	6948	11175
PHL	2006	country	6_Wpr	Philippines	36564	42245	78809	0.0155333	0.0179467	0.03348	2353918	4280	4723	9003
PHL	2007	country	6_Wpr	Philippines	36150	41311	77461	0.0153736	0.0175683	0.032942	2351428	4237	4523	8761
PHL	2008	country	6_Wpr	Philippines	35822	40344	76166	0.0152313	0.017154	0.0323852	2351871	4302	4366	8668
PHL	2009	country	6_Wpr	Philippines	35574	39613	75187	0.0151057	0.0168208	0.0319265	2354999	4330	3949	8279
PHL	2010	country	6_Wpr	Philippines	35325	39133	74458	0.0149811	0.0165961	0.0315772	2357972	4402	3404	7806
PHL	2011	country	6_Wpr	Philippines	35030	38658	73688	0.0148646	0.016404	0.0312687	2356601	4461	3785	8246
PHL	2012	country	6_Wpr	Philippines	34638	37733	72371	0.0147562	0.0160748	0.0308309	2347359	4451	3777	8227
PHL	2013	country	6_Wpr	Philippines	34100	37082	71182	0.0146424	0.0159229	0.0305653	2328855	4440	3815	8256
PHL	2014	country	6_Wpr	Philippines	33494	37471	70965	0.0145504	0.0162779	0.0308283	2301925	4468	3390	7857
PHL	2015	country	6_Wpr	Philippines	32738	35629	68367	0.0144228	0.0156964	0.0301193	2269877	4399	3516	7914
PHL	2016	country	6_Wpr	Philippines	31919	34379	66298	0.0142649	0.0153643	0.0296291	2237594	4361	3428	7789
PHL	2017	country	6_Wpr	Philippines	30894	33257	64151	0.0139785	0.0150475	0.029026	2210108	4312	3225	7537
PHL	2018	country	6_Wpr	Philippines	29908	32031	61939	0.0136495	0.0146186	0.028268	2191144	4265	3106	7371
PHL	2019	country	6_Wpr	Philippines	28992	31401	60393	0.0132875	0.0143916	0.0276791	2181896	4132	3079	7211
PLW	2000	country	6_Wpr	Palau	5	4	9	0.0158535	0.0126828	0.0285363	315	1	1	1
PLW	2001	country	6_Wpr	Palau	5	4	9	0.0154973	0.0123978	0.0278951	323	1	1	1
PLW	2002	country	6_Wpr	Palau	5	3	8	0.0152046	0.0091228	0.0243273	329	1	1	1
PLW	2003	country	6_Wpr	Palau	4	4	8	0.0149135	0.0149135	0.0298271	268	1	1	1
PLW	2004	country	6_Wpr	Palau	4	4	8	0.0146158	0.0146158	0.0292315	274	1	1	1
PLW	2005	country	6_Wpr	Palau	4	3	7	0.014304	0.010728	0.025032	280	1	1	1
PLW	2006	country	6_Wpr	Palau	4	3	7	0.013989	0.0104917	0.0244807	286	1	1	1
PLW	2007	country	6_Wpr	Palau	4	3	7	0.013656	0.010242	0.023898	293	1	1	1
PLW	2008	country	6_Wpr	Palau	3	3	6	0.0132935	0.0132935	0.0265869	226	0	1	1
PLW	2009	country	6_Wpr	Palau	3	3	6	0.0129188	0.0129188	0.0258375	232	0	1	1
PLW	2010	country	6_Wpr	Palau	3	3	6	0.0125429	0.0125429	0.0250857	239	1	1	1
PLW	2011	country	6_Wpr	Palau	3	3	6	0.0121594	0.0121594	0.0243189	247	1	1	1
PLW	2012	country	6_Wpr	Palau	3	2	5	0.0118092	0.0078728	0.0196819	254	1	0	1
PLW	2013	country	6_Wpr	Palau	3	2	5	0.0114303	0.0076202	0.0190505	262	1	0	1
PLW	2014	country	6_Wpr	Palau	3	2	5	0.0110403	0.0073602	0.0184005	272	1	0	1
PLW	2015	country	6_Wpr	Palau	3	2	5	0.010652	0.0071013	0.0177534	282	1	0	1
PLW	2016	country	6_Wpr	Palau	3	2	5	0.0103061	0.0068707	0.0171768	291	1	0	1
PLW	2017	country	6_Wpr	Palau	2	2	4	0.0099877	0.0099877	0.0199754	200	0	0	1
PLW	2018	country	6_Wpr	Palau	2	2	4	0.0096437	0.0096437	0.0192875	207	0	0	1
PLW	2019	country	6_Wpr	Palau	2	2	4	0.0093628	0.0093628	0.0187256	214	0	1	1

iso3	year	level	whoreg6	whoname	nnd	pnd	u5d	nmr	pnmr	u5mr	Livebirths	Neonatal birth defects deaths	1-59 month birth defects deaths	Under five birth defects deaths
PNG	2000	country	6_Wpr	Papua New Guinea	6091	8152	14243	0.0307319	0.0411301	0.071862	198198	406	186	592
PNG	2001	country	6_Wpr	Papua New Guinea	6050	8122	14172	0.0301356	0.0404559	0.0705915	200760	404	174	579
PNG	2002	country	6_Wpr	Papua New Guinea	6004	8896	14900	0.0295577	0.0437936	0.0733513	203128	417	232	649
PNG	2003	country	6_Wpr	Papua New Guinea	5963	8192	14155	0.0290378	0.0398904	0.0689282	205353	425	249	674
PNG	2004	country	6_Wpr	Papua New Guinea	5937	7553	13490	0.0286039	0.0363896	0.0649935	207559	427	213	640
PNG	2005	country	6_Wpr	Papua New Guinea	5918	7285	13203	0.0282232	0.0347445	0.0629677	209685	426	280	706
PNG	2006	country	6_Wpr	Papua New Guinea	5900	6937	12837	0.0278652	0.032764	0.0606292	211733	444	283	726
PNG	2007	country	6_Wpr	Papua New Guinea	5873	6859	12732	0.0274898	0.0321042	0.0595941	213643	445	256	701
PNG	2008	country	6_Wpr	Papua New Guinea	5838	6764	12602	0.0270976	0.031397	0.0584946	215444	448	240	688
PNG	2009	country	6_Wpr	Papua New Guinea	5782	6670	12452	0.0266272	0.0307166	0.0573438	217146	444	208	652
PNG	2010	country	6_Wpr	Papua New Guinea	5716	6517	12233	0.0261289	0.0297899	0.0559188	218762	448	259	707
PNG	2011	country	6_Wpr	Papua New Guinea	5643	6438	12081	0.0256108	0.0292169	0.0548277	220337	448	304	753
PNG	2012	country	6_Wpr	Papua New Guinea	5578	6312	11890	0.0251303	0.0284364	0.0535667	221964	450	316	765
PNG	2013	country	6_Wpr	Papua New Guinea	5516	6140	11656	0.0246679	0.02746	0.0521279	223611	464	281	745
PNG	2014	country	6_Wpr	Papua New Guinea	5452	6206	11658	0.0241942	0.0275395	0.0517337	225343	470	267	737
PNG	2015	country	6_Wpr	Papua New Guinea	5386	6011	11397	0.0237132	0.0264643	0.0501775	227131	466	307	772
PNG	2016	country	6_Wpr	Papua New Guinea	5335	5760	11095	0.0232924	0.025147	0.0484394	229045	461	214	675
PNG	2017	country	6_Wpr	Papua New Guinea	5276	5660	10936	0.0228335	0.0244948	0.0473284	231064	473	172	646
PNG	2018	country	6_Wpr	Papua New Guinea	5211	5567	10778	0.0223514	0.023877	0.0462284	233139	460	161	621
PNG	2019	country	6_Wpr	Papua New Guinea	5160	5369	10529	0.0219373	0.0228265	0.0447637	235216	456	170	626
POL	2000	country	4_Eur	Poland	2179	1442	3621	0.0057584	0.0038108	0.0095692	378402	769	547	1316
POL	2001	country	4_Eur	Poland	2014	1318	3332	0.0054365	0.0035578	0.0089943	370456	679	506	1185
POL	2002	country	4_Eur	Poland	1913	1218	3131	0.0052115	0.0033182	0.0085297	367072	627	464	1091
POL	2003	country	4_Eur	Poland	1843	1150	2993	0.0050078	0.0031248	0.0081325	368029	576	441	1017
POL	2004	country	4_Eur	Poland	1786	1111	2897	0.0047936	0.0029819	0.0077755	372581	583	443	1026
POL	2005	country	4_Eur	Poland	1740	1092	2832	0.0045884	0.0028796	0.0074679	379221	608	414	1022
POL	2006	country	4_Eur	Poland	1698	1084	2782	0.0043932	0.0028046	0.0071979	386504	504	408	912
POL	2007	country	4_Eur	Poland	1652	1069	2721	0.0042087	0.0027234	0.0069321	392521	543	429	971
POL	2008	country	4_Eur	Poland	1590	1037	2627	0.0040146	0.0026183	0.0066329	396057	478	382	860
POL	2009	country	4_Eur	Poland	1508	987	2495	0.0038003	0.0024873	0.0062876	396810	437	418	855
POL	2010	country	4_Eur	Poland	1417	934	2351	0.0035896	0.002366	0.0059556	394754	462	391	854
POL	2011	country	4_Eur	Poland	1327	895	2222	0.0033941	0.0022892	0.0056833	390972	422	394	816
POL	2012	country	4_Eur	Poland	1247	865	2112	0.0032245	0.0022367	0.0054612	386730	393	402	795
POL	2013	country	4_Eur	Poland	1183	830	2013	0.0030885	0.0021669	0.0052555	383030	406	352	758
POL	2014	country	4_Eur	Poland	1133	789	1922	0.0029799	0.0020751	0.005055	380219	393	347	740
POL	2015	country	4_Eur	Poland	1097	746	1843	0.0029018	0.0019734	0.0048752	378036	341	352	693
POL	2016	country	4_Eur	Poland	1070	706	1776	0.0028434	0.0018761	0.0047195	376312	333	323	656
POL	2017	country	4_Eur	Poland	1048	672	1720	0.0028006	0.0017958	0.0045965	374201	338	307	645
POL	2018	country	4_Eur	Poland	1025	645	1670	0.0027606	0.0017372	0.0044978	371292	323	295	617
POL	2019	country	4_Eur	Poland	997	627	1624	0.0027166	0.0017085	0.0044251	366999	316	286	602

iso3	year	level	whoreg6	whoname	nnd	pnd	u5d	nmr	pnmr	u5mr	Livebirths	Neonatal birth defects deaths	1-59 month birth defects deaths	Under five birth defects deaths
PRK	2000	country	3_Sear	Democratic People	10858	14063	24921	0.0263189	0.0340876	0.0604065	412555	1162	871	2033
PRK	2001	country	3_Sear	Democratic People	9742	11559	21301	0.0240398	0.0285235	0.0525633	405245	1050	1109	2158
PRK	2002	country	3_Sear	Democratic People	8606	9346	17952	0.0216708	0.0235342	0.045205	397125	987	1138	2125
PRK	2003	country	3_Sear	Democratic People	7652	7664	15316	0.0197045	0.0197354	0.0394399	388338	887	950	1837
PRK	2004	country	3_Sear	Democratic People	6939	6550	13489	0.0182943	0.0172687	0.035563	379299	834	856	1690
PRK	2005	country	3_Sear	Democratic People	6500	5941	12441	0.0175433	0.0160345	0.0335778	370513	827	850	1677
PRK	2006	country	3_Sear	Democratic People	6268	5684	11952	0.0172901	0.0156791	0.0329692	362520	836	829	1665
PRK	2007	country	3_Sear	Democratic People	6133	5509	11642	0.0172419	0.0154876	0.0327295	355703	830	838	1669
PRK	2008	country	3_Sear	Democratic People	5983	5314	11297	0.0170789	0.0151692	0.0322481	350315	831	805	1636
PRK	2009	country	3_Sear	Democratic People	5803	5054	10857	0.0167421	0.0145812	0.0313233	346611	827	784	1610
PRK	2010	country	3_Sear	Democratic People	5575	4651	10226	0.0161816	0.0134997	0.0296813	344527	826	769	1595
PRK	2011	country	3_Sear	Democratic People	5247	4299	9546	0.0152481	0.0124932	0.0277413	344108	812	763	1576
PRK	2012	country	3_Sear	Democratic People	4887	3954	8841	0.0141721	0.0114664	0.0256385	344833	812	773	1584
PRK	2013	country	3_Sear	Democratic People	4555	3713	8268	0.0131516	0.0107205	0.023872	346347	796	778	1574
PRK	2014	country	3_Sear	Democratic People	4281	3463	7744	0.0122919	0.0099432	0.0222352	348277	782	753	1534
PRK	2015	country	3_Sear	Democratic People	4058	3272	7330	0.0115849	0.009341	0.020926	350282	797	733	1530
PRK	2016	country	3_Sear	Democratic People	3870	3114	6984	0.0109909	0.0088438	0.0198347	352110	792	716	1508
PRK	2017	country	3_Sear	Democratic People	3701	2968	6669	0.0104664	0.0083935	0.0188599	353607	786	699	1485
PRK	2018	country	3_Sear	Democratic People	3536	2879	6415	0.009969	0.0081168	0.0180858	354698	766	691	1458
PRK	2019	country	3_Sear	Democratic People	3376	2740	6116	0.0095032	0.0077129	0.0172161	355249	751	673	1424
PRT	2000	country	4_Eur	Portugal	379	429	808	0.0033539	0.0037964	0.0071503	113002	109	145	253
PRT	2001	country	4_Eur	Portugal	360	386	746	0.0031883	0.0034185	0.0066068	112913	105	101	207
PRT	2002	country	4_Eur	Portugal	340	341	681	0.0030301	0.003039	0.0060691	112208	100	73	173
PRT	2003	country	4_Eur	Portugal	314	303	617	0.0028135	0.002715	0.0055285	111604	93	74	167
PRT	2004	country	4_Eur	Portugal	283	276	559	0.0025668	0.0025033	0.0050701	110255	82	53	135
PRT	2005	country	4_Eur	Portugal	255	255	510	0.0023429	0.0023429	0.0046859	108838	77	25	101
PRT	2006	country	4_Eur	Portugal	234	239	473	0.0021952	0.0022421	0.0044372	106598	70	26	96
PRT	2007	country	4_Eur	Portugal	220	223	443	0.0021083	0.0021371	0.0042454	104348	67	27	94
PRT	2008	country	4_Eur	Portugal	212	206	418	0.0020835	0.0020245	0.004108	101752	65	28	93
PRT	2009	country	4_Eur	Portugal	208	188	396	0.0021045	0.0019021	0.0040066	98836	64	29	93
PRT	2010	country	4_Eur	Portugal	204	171	375	0.0021296	0.0017851	0.0039147	95793	62	30	92
PRT	2011	country	4_Eur	Portugal	199	159	358	0.0021301	0.0017019	0.003832	93423	61	32	93
PRT	2012	country	4_Eur	Portugal	192	150	342	0.002117	0.0016539	0.003771	90693	59	35	94
PRT	2013	country	4_Eur	Portugal	186	143	329	0.0021094	0.0016217	0.0037311	88177	57	38	95
PRT	2014	country	4_Eur	Portugal	181	138	319	0.0021031	0.0016035	0.0037066	86063	55	43	98
PRT	2015	country	4_Eur	Portugal	177	134	311	0.0021049	0.0015935	0.0036984	84091	54	42	96
PRT	2016	country	4_Eur	Portugal	172	133	305	0.0020823	0.0016101	0.0036924	82601	53	37	91
PRT	2017	country	4_Eur	Portugal	166	135	301	0.0020455	0.0016635	0.0037089	81156	52	41	92
PRT	2018	country	4_Eur	Portugal	161	137	298	0.0020153	0.0017149	0.0037302	79888	50	41	92
PRT	2019	country	4_Eur	Portugal	156	139	295	0.0019741	0.001759	0.0037331	79023	48	42	90

iso3	year	level	whoreg6	whoname	nnd	pnd	u5d	nmr	pnmr	u5mr	Livebirths	Neonatal birth defects deaths	1-59 month birth defects deaths	Under five birth defects deaths
PRY	2000	country	2_Amr	Paraguay	2602	2238	4840	0.0181496	0.0156106	0.0337602	143364	297	349	645
PRY	2001	country	2_Amr	Paraguay	2538	2155	4693	0.0178562	0.0151616	0.0330177	142136	297	319	616
PRY	2002	country	2_Amr	Paraguay	2475	2078	4553	0.0175501	0.014735	0.0322851	141025	295	289	584
PRY	2003	country	2_Amr	Paraguay	2419	1998	4417	0.0172401	0.0142397	0.0314798	140312	298	305	602
PRY	2004	country	2_Amr	Paraguay	2369	1928	4297	0.016935	0.0137824	0.0307174	139888	300	297	597
PRY	2005	country	2_Amr	Paraguay	2321	1868	4189	0.016591	0.0133529	0.0299439	139895	303	289	592
PRY	2006	country	2_Amr	Paraguay	2282	1797	4079	0.0162887	0.0128268	0.0291155	140097	304	281	585
PRY	2007	country	2_Amr	Paraguay	2229	1744	3973	0.0158864	0.0124298	0.0283162	140308	302	277	579
PRY	2008	country	2_Amr	Paraguay	2169	1696	3865	0.0154322	0.0120668	0.027499	140551	306	274	579
PRY	2009	country	2_Amr	Paraguay	2108	1651	3759	0.0149905	0.0117406	0.0267311	140623	301	272	572
PRY	2010	country	2_Amr	Paraguay	2048	1597	3645	0.0145591	0.011353	0.0259121	140668	301	272	573
PRY	2011	country	2_Amr	Paraguay	1986	1548	3534	0.0141152	0.0110022	0.0251174	140700	297	271	568
PRY	2012	country	2_Amr	Paraguay	1920	1506	3426	0.0136389	0.010698	0.0243369	140774	297	273	570
PRY	2013	country	2_Amr	Paraguay	1863	1458	3321	0.0132071	0.010336	0.0235432	141060	296	273	569
PRY	2014	country	2_Amr	Paraguay	1808	1422	3230	0.012784	0.0100547	0.0228387	141427	293	274	567
PRY	2015	country	2_Amr	Paraguay	1760	1375	3135	0.0124088	0.0096943	0.0221031	141835	290	272	562
PRY	2016	country	2_Amr	Paraguay	1705	1337	3042	0.0119768	0.0093918	0.0213685	142359	292	272	563
PRY	2017	country	2_Amr	Paraguay	1656	1299	2955	0.0115971	0.009097	0.0206941	142794	289	271	560
PRY	2018	country	2_Amr	Paraguay	1605	1262	2867	0.0112177	0.0088204	0.0200382	143077	285	270	555
PRY	2019	country	2_Amr	Paraguay	1555	1219	2774	0.0108552	0.0085097	0.0193649	143249	279	267	546
QAT	2000	country	5_Emr	Qatar	78	65	143	0.0066133	0.0055111	0.0121243	11794	19	25	43
QAT	2001	country	5_Emr	Qatar	78	65	143	0.0063418	0.0052849	0.0116267	12299	19	25	44
QAT	2002	country	5_Emr	Qatar	78	65	143	0.0061044	0.005087	0.0111913	12778	19	25	44
QAT	2003	country	5_Emr	Qatar	79	65	144	0.0058898	0.004846	0.0107359	13413	20	25	45
QAT	2004	country	5_Emr	Qatar	80	65	145	0.0056789	0.0046141	0.0102929	14087	20	25	45
QAT	2005	country	5_Emr	Qatar	81	67	148	0.0054675	0.0045225	0.00999	14815	21	26	47
QAT	2006	country	5_Emr	Qatar	83	68	151	0.0052678	0.0043158	0.0095836	15756	21	27	48
QAT	2007	country	5_Emr	Qatar	85	71	156	0.0050853	0.0042477	0.009333	16715	22	27	49
QAT	2008	country	5_Emr	Qatar	88	74	162	0.0049118	0.0041303	0.0090421	17916	23	29	52
QAT	2009	country	5_Emr	Qatar	91	78	169	0.0047516	0.0040728	0.0088243	19152	24	31	55
QAT	2010	country	5_Emr	Qatar	94	82	176	0.004607	0.0040189	0.008626	20404	24	32	57
QAT	2011	country	5_Emr	Qatar	96	87	183	0.0044678	0.0040489	0.0085167	21487	26	34	60
QAT	2012	country	5_Emr	Qatar	99	89	188	0.0043416	0.0039031	0.0082447	22803	26	35	61
QAT	2013	country	5_Emr	Qatar	100	92	192	0.004218	0.0038806	0.0080986	23708	27	37	64
QAT	2014	country	5_Emr	Qatar	101	93	194	0.0040971	0.0037726	0.0078696	24652	27	38	65
QAT	2015	country	5_Emr	Qatar	100	93	193	0.0039687	0.0036909	0.0076596	25197	27	38	65
QAT	2016	country	5_Emr	Qatar	99	91	190	0.003838	0.0035278	0.0073658	25795	27	37	64
QAT	2017	country	5_Emr	Qatar	97	87	184	0.0036899	0.0033095	0.0069994	26288	26	36	62
QAT	2018	country	5_Emr	Qatar	94	84	178	0.0035393	0.0031628	0.0067021	26559	25	35	60
QAT	2019	country	5_Emr	Qatar	90	82	172	0.0033941	0.0030924	0.0064865	26517	24	34	58

iso3	year	level	whoreg6	whoname	nnd	pnd	u5d	nmr	pnmr	u5mr	Livebirths	Neonatal birth defects deaths	1-59 month birth defects deaths	Under five birth defects deaths
ROU	2000	country	4_Eur	Romania	2268	2407	4675	0.0104691	0.0111107	0.0215799	216637	528	387	914
ROU	2001	country	4_Eur	Romania	2143	2301	4444	0.0099626	0.0106971	0.0206598	215104	573	375	949
ROU	2002	country	4_Eur	Romania	2086	2265	4351	0.0097002	0.0105326	0.0202328	215047	651	394	1046
ROU	2003	country	4_Eur	Romania	2048	2239	4287	0.0094724	0.0103558	0.0198282	216208	557	463	1020
ROU	2004	country	4_Eur	Romania	1989	2184	4173	0.0091046	0.0099972	0.0191018	218461	475	351	826
ROU	2005	country	4_Eur	Romania	1883	2097	3980	0.0085242	0.009493	0.0180173	220899	489	434	923
ROU	2006	country	4_Eur	Romania	1727	1965	3692	0.0077503	0.0088184	0.0165687	222830	445	349	794
ROU	2007	country	4_Eur	Romania	1549	1817	3366	0.0069352	0.0081351	0.0150703	223353	402	352	754
ROU	2008	country	4_Eur	Romania	1389	1695	3084	0.0062417	0.0076167	0.0138584	222536	374	273	647
ROU	2009	country	4_Eur	Romania	1266	1605	2871	0.0057592	0.0073013	0.0130605	219823	364	295	659
ROU	2010	country	4_Eur	Romania	1176	1518	2694	0.0054487	0.0070333	0.012482	215831	358	261	619
ROU	2011	country	4_Eur	Romania	1115	1414	2529	0.0052864	0.0067039	0.0119903	210921	322	254	575
ROU	2012	country	4_Eur	Romania	1062	1297	2359	0.0051573	0.0062986	0.0114559	205920	272	288	560
ROU	2013	country	4_Eur	Romania	1000	1174	2174	0.0049617	0.005825	0.0107867	201544	261	242	503
ROU	2014	country	4_Eur	Romania	920	1060	1980	0.0046451	0.005352	0.0099971	198057	226	210	437
ROU	2015	country	4_Eur	Romania	839	968	1807	0.0042946	0.0049549	0.0092495	195361	220	205	425
ROU	2016	country	4_Eur	Romania	770	901	1671	0.0039811	0.0046584	0.0086394	193415	187	193	380
ROU	2017	country	4_Eur	Romania	715	842	1557	0.0037353	0.0043987	0.008134	191419	181	176	358
ROU	2018	country	4_Eur	Romania	674	766	1440	0.0035615	0.0040477	0.0076092	189246	170	162	333
ROU	2019	country	4_Eur	Romania	644	673	1317	0.0034476	0.0036028	0.0070504	186799	161	143	303
RUS	2000	country	4_Eur	Russian Federatior	11934	13457	25391	0.008995	0.010143	0.019138	1326730	2668	3112	5780
RUS	2001	country	4_Eur	Russian Federatior	11624	12814	24438	0.0085853	0.0094642	0.0180495	1353945	2594	2997	5591
RUS	2002	country	4_Eur	Russian Federatior	11066	12376	23442	0.007965	0.0089079	0.0168729	1389330	2521	2930	5452
RUS	2003	country	4_Eur	Russian Federatior	10527	11895	22422	0.0073606	0.0083171	0.0156777	1430185	2412	2875	5287
RUS	2004	country	4_Eur	Russian Federatior	10097	11305	21402	0.00685	0.0076695	0.0145195	1474017	2353	2800	5154
RUS	2005	country	4_Eur	Russian Federatior	9698	10715	20413	0.0063806	0.0070497	0.0134303	1519925	2275	2724	4998
RUS	2006	country	4_Eur	Russian Federatior	9250	10253	19503	0.0058993	0.006539	0.0124384	1567972	2168	2672	4840
RUS	2007	country	4_Eur	Russian Federatior	8766	9985	18751	0.0054157	0.0061688	0.0115846	1618617	2095	2654	4749
RUS	2008	country	4_Eur	Russian Federatior	8291	9948	18239	0.0049611	0.0059526	0.0109136	1671213	1988	2690	4677
RUS	2009	country	4_Eur	Russian Federatior	7909	10068	17977	0.0045895	0.0058423	0.0104318	1723282	1955	2813	4768
RUS	2010	country	4_Eur	Russian Federatior	7857	10054	17911	0.0044327	0.0056721	0.0101048	1772528	1935	2871	4807
RUS	2011	country	4_Eur	Russian Federatior	8293	9654	17947	0.0045668	0.0053163	0.0098831	1815929	2042	2719	4762
RUS	2012	country	4_Eur	Russian Federatior	8880	9015	17895	0.0047952	0.0048681	0.0096634	1851836	2197	2471	4668
RUS	2013	country	4_Eur	Russian Federatior	8948	8554	17502	0.0047637	0.0045539	0.0093176	1878391	2207	2355	4562
RUS	2014	country	4_Eur	Russian Federatior	8270	8400	16670	0.0043654	0.0044341	0.0087995	1894423	2056	2431	4487
RUS	2015	country	4_Eur	Russian Federatior	7346	8191	15537	0.0038695	0.0043146	0.0081841	1898446	1867	2463	4330
RUS	2016	country	4_Eur	Russian Federatior	6608	7641	14249	0.0034967	0.0040433	0.00754	1889783	1677	2352	4028
RUS	2017	country	4_Eur	Russian Federatior	6046	6885	12931	0.0032324	0.003681	0.0069134	1870418	1552	2127	3679
RUS	2018	country	4_Eur	Russian Federatior	5432	6251	11683	0.0029487	0.0033932	0.0063419	1842197	1452	1973	3425
RUS	2019	country	4_Eur	Russian Federatior	4751	5805	10556	0.0026287	0.0032119	0.0058406	1807352	1265	1866	3132

iso3	year	level	whoreg6	whoname	nnd	pnd	u5d	nmr	pnmr	u5mr	Livebirths	Neonatal birth defects deaths	1-59 month birth defects deaths	Under five birth defects deaths
RWA	2000	country	1_Afr	Rwanda	12241	39205	51446	0.0408613	0.1308676	0.1717289	299575	534	285	819
RWA	2001	country	1_Afr	Rwanda	12027	36666	48693	0.0392748	0.1197344	0.1590092	306227	568	195	762
RWA	2002	country	1_Afr	Rwanda	11721	33924	45645	0.037438	0.1083566	0.1457945	313078	556	396	952
RWA	2003	country	1_Afr	Rwanda	11294	30696	41990	0.0353308	0.0960272	0.131358	319664	581	559	1139
RWA	2004	country	1_Afr	Rwanda	10832	27153	37985	0.0332552	0.0833621	0.1166173	325723	583	603	1186
RWA	2005	country	1_Afr	Rwanda	10330	24282	34612	0.0312173	0.0733805	0.1045977	330907	585	792	1376
RWA	2006	country	1_Afr	Rwanda	9827	21722	31549	0.0293263	0.0648235	0.0941497	335092	594	1003	1598
RWA	2007	country	1_Afr	Rwanda	9348	19318	28666	0.027616	0.0570701	0.0846861	338500	588	1038	1626
RWA	2008	country	1_Afr	Rwanda	8894	17224	26118	0.026047	0.0504435	0.0764905	341459	589	1022	1611
RWA	2009	country	1_Afr	Rwanda	8453	15380	23833	0.0245678	0.0446992	0.069267	344068	586	1098	1684
RWA	2010	country	1_Afr	Rwanda	8023	13639	21662	0.0231332	0.0393251	0.0624583	346818	571	1163	1734
RWA	2011	country	1_Afr	Rwanda	7621	12039	19660	0.0217611	0.0343759	0.056137	350212	564	1169	1733
RWA	2012	country	1_Afr	Rwanda	7275	10852	18127	0.0205248	0.0306153	0.0511402	354449	556	1234	1790
RWA	2013	country	1_Afr	Rwanda	6995	9876	16871	0.0194482	0.0274574	0.0469057	359673	572	1174	1746
RWA	2014	country	1_Afr	Rwanda	6809	9003	15812	0.0186181	0.0246171	0.0432352	365720	589	1187	1776
RWA	2015	country	1_Afr	Rwanda	6649	8427	15076	0.0178565	0.0226325	0.040489	372358	609	1132	1741
RWA	2016	country	1_Afr	Rwanda	6537	7986	14523	0.017248	0.0210718	0.0383199	379000	643	1069	1713
RWA	2017	country	1_Afr	Rwanda	6443	7682	14125	0.0167307	0.0199489	0.0366796	385101	672	1080	1752
RWA	2018	country	1_Afr	Rwanda	6346	7375	13721	0.016258	0.0188947	0.0351527	390330	692	1114	1807
RWA	2019	country	1_Afr	Rwanda	6261	7085	13346	0.0158751	0.0179635	0.0338386	394391	698	1081	1780
SAU	2000	country	5_Emr	Saudi Arabia	6700	5373	12073	0.0121595	0.0097512	0.0219106	551011	1394	1611	3005
SAU	2001	country	5_Emr	Saudi Arabia	6359	5041	11400	0.0114831	0.009103	0.0205861	553773	1427	1561	2988
SAU	2002	country	5_Emr	Saudi Arabia	6050	4756	10806	0.0108352	0.0085177	0.019353	558364	1418	1522	2941
SAU	2003	country	5_Emr	Saudi Arabia	5778	4506	10284	0.010241	0.0079865	0.0182276	564201	1405	1477	2882
SAU	2004	country	5_Emr	Saudi Arabia	5521	4288	9809	0.0096699	0.0075104	0.0171803	570945	1378	1437	2815
SAU	2005	country	5_Emr	Saudi Arabia	5280	4077	9357	0.0091329	0.0070521	0.016185	578128	1339	1398	2737
SAU	2006	country	5_Emr	Saudi Arabia	5038	3875	8913	0.0086032	0.0066172	0.0152204	585594	1306	1357	2663
SAU	2007	country	5_Emr	Saudi Arabia	4791	3696	8487	0.0080818	0.0062347	0.0143165	592811	1241	1317	2558
SAU	2008	country	5_Emr	Saudi Arabia	4556	3505	8061	0.007598	0.0058452	0.0134432	599632	1183	1277	2460
SAU	2009	country	5_Emr	Saudi Arabia	4308	3327	7635	0.007115	0.0054948	0.0126098	605483	1143	1234	2378
SAU	2010	country	5_Emr	Saudi Arabia	4059	3147	7206	0.0066531	0.0051582	0.0118113	610093	1100	1189	2289
SAU	2011	country	5_Emr	Saudi Arabia	3817	2971	6788	0.006225	0.0048453	0.0110704	613168	1042	1141	2183
SAU	2012	country	5_Emr	Saudi Arabia	3573	2793	6366	0.0058102	0.0045418	0.010352	614954	975	1090	2064
SAU	2013	country	5_Emr	Saudi Arabia	3333	2629	5962	0.0054182	0.0042737	0.0096919	615153	911	1041	1952
SAU	2014	country	5_Emr	Saudi Arabia	3091	2469	5560	0.0050325	0.0040198	0.0090522	614213	851	991	1842
SAU	2015	country	5_Emr	Saudi Arabia	2870	2293	5163	0.0046909	0.0037478	0.0084387	611827	801	933	1734
SAU	2016	country	5_Emr	Saudi Arabia	2650	2143	4793	0.0043578	0.003524	0.0078818	608112	748	883	1631
SAU	2017	country	5_Emr	Saudi Arabia	2460	2018	4478	0.0040763	0.0033439	0.0074201	603493	709	840	1549
SAU	2018	country	5_Emr	Saudi Arabia	2303	1897	4200	0.0038499	0.0031712	0.007021	598205	655	796	1451
SAU	2019	country	5_Emr	Saudi Arabia	2170	1794	3964	0.0036656	0.0030304	0.006696	591993	619	759	1378

iso3	year	level	whoreg6	whoname	nnd	pnd	u5d	nmr	pnmr	u5mr	Livebirths	Neonatal birth defects deaths	1-59 month birth defects deaths	Under five birth defects deaths
SDN	2000	country	5_Emr	Sudan	39942	70258	110200	0.0368932	0.0648955	0.1017886	1082639	2469	1209	3678
SDN	2001	country	5_Emr	Sudan	39870	70297	110167	0.0362189	0.0638596	0.1000784	1100808	2560	1251	3811
SDN	2002	country	5_Emr	Sudan	39755	69173	108928	0.0355582	0.0618705	0.0974287	1118026	2657	1319	3976
SDN	2003	country	5_Emr	Sudan	39592	67441	107033	0.034904	0.0594553	0.0943592	1134313	2675	1058	3733
SDN	2004	country	5_Emr	Sudan	39382	62243	101625	0.0342538	0.0541379	0.0883917	1149712	2794	1744	4539
SDN	2005	country	5_Emr	Sudan	39219	59277	98496	0.0336847	0.050912	0.0845966	1164299	2888	1868	4756
SDN	2006	country	5_Emr	Sudan	39123	57340	96463	0.0332082	0.0486715	0.0818797	1178112	2893	2117	5011
SDN	2007	country	5_Emr	Sudan	39077	55549	94626	0.0328026	0.0466298	0.0794324	1191278	2990	2540	5530
SDN	2008	country	5_Emr	Sudan	39053	53947	93000	0.0324357	0.0448058	0.0772414	1204014	3089	1773	4862
SDN	2009	country	5_Emr	Sudan	39016	52436	91452	0.0320719	0.0431032	0.0751751	1216515	3086	1967	5053
SDN	2010	country	5_Emr	Sudan	38951	51346	90297	0.0316914	0.0417763	0.0734677	1229070	3217	2424	5641
SDN	2011	country	5_Emr	Sudan	38926	50537	89463	0.0313464	0.0406963	0.0720427	1241802	3219	2310	5529
SDN	2012	country	5_Emr	Sudan	38824	50121	88945	0.0309389	0.0399413	0.0708803	1254860	3280	3372	6651
SDN	2013	country	5_Emr	Sudan	38687	49375	88062	0.0305005	0.0389267	0.0694271	1268407	3393	3502	6895
SDN	2014	country	5_Emr	Sudan	38418	45581	83999	0.0299545	0.0355398	0.0654944	1282544	3411	2613	6024
SDN	2015	country	5_Emr	Sudan	38147	45352	83499	0.0294031	0.0349569	0.06436	1297380	3414	2664	6078
SDN	2016	country	5_Emr	Sudan	37882	45385	83267	0.0288511	0.0345656	0.0634167	1313018	3430	2603	6033
SDN	2017	country	5_Emr	Sudan	37634	43516	81150	0.0283096	0.0327345	0.0610441	1329373	3555	2830	6385
SDN	2018	country	5_Emr	Sudan	37371	44552	81923	0.0277575	0.0330915	0.060849	1346340	3525	2687	6212
SDN	2019	country	5_Emr	Sudan	37126	46439	83565	0.02722	0.0340478	0.0612678	1363925	3568	2658	6226
SEN	2000	country	1_Afr	Senegal	14583	35068	49651	0.0378782	0.0910857	0.1289639	384997	684	277	961
SEN	2001	country	1_Afr	Senegal	14475	38477	52952	0.0369032	0.0980943	0.1349975	392243	693	193	886
SEN	2002	country	1_Afr	Senegal	14325	43793	58118	0.035794	0.109426	0.14522	400207	709	258	967
SEN	2003	country	1_Afr	Senegal	14135	29694	43829	0.0345606	0.0726041	0.1071647	408991	722	340	1062
SEN	2004	country	1_Afr	Senegal	13928	24854	38782	0.0332736	0.0593757	0.0926493	418590	721	367	1088
SEN	2005	country	1_Afr	Senegal	13723	23396	37119	0.0319889	0.0545362	0.0865251	428993	728	630	1358
SEN	2006	country	1_Afr	Senegal	13541	21978	35519	0.0307621	0.0499282	0.0806903	440185	745	793	1537
SEN	2007	country	1_Afr	Senegal	13403	20522	33925	0.0296629	0.0454174	0.0750803	451844	753	923	1676
SEN	2008	country	1_Afr	Senegal	13261	19641	32902	0.0285971	0.0423562	0.0709533	463718	757	806	1563
SEN	2009	country	1_Afr	Senegal	13149	19234	32383	0.0276538	0.0404513	0.0681051	475486	768	855	1623
SEN	2010	country	1_Afr	Senegal	13045	18381	31426	0.0267956	0.0377554	0.064551	486834	773	910	1682
SEN	2011	country	1_Afr	Senegal	12967	17402	30369	0.0260722	0.0349902	0.0610624	497350	767	992	1760
SEN	2012	country	1_Afr	Senegal	12903	16739	29642	0.0254535	0.0330212	0.0584747	506925	789	1449	2238
SEN	2013	country	1_Afr	Senegal	12825	16128	28953	0.0248764	0.0312831	0.0561594	515550	786	1021	1807
SEN	2014	country	1_Afr	Senegal	12768	15152	27920	0.0244066	0.0289641	0.0533707	523138	799	1011	1811
SEN	2015	country	1_Afr	Senegal	12696	14613	27309	0.0239599	0.0275781	0.051538	529885	808	1032	1840
SEN	2016	country	1_Afr	Senegal	12591	14045	26636	0.0234907	0.0262026	0.0496933	536000	803	1347	2150
SEN	2017	country	1_Afr	Senegal	12460	13512	25972	0.0229989	0.0249416	0.0479406	541764	811	1226	2038
SEN	2018	country	1_Afr	Senegal	12322	12950	25272	0.0225054	0.0236528	0.0461582	547512	802	962	1764
SEN	2019	country	1_Afr	Senegal	12156	12591	24747	0.0219631	0.0227498	0.044713	553473	805	942	1746

iso3	year	level	whoreg6	whoname	nnd	pnd	u5d	nmr	pnmr	u5mr	Livebirths	Neonatal birth defects deaths	1-59 month birth defects deaths	Under five birth defects deaths
SGP	2000	country	6_Wpr	Singapore	81	119	200	0.0016088	0.0023636	0.0039724	50347	36	31	67
SGP	2001	country	6_Wpr	Singapore	73	109	182	0.0014845	0.0022166	0.0037011	49174	41	28	70
SGP	2002	country	6_Wpr	Singapore	67	99	166	0.0013953	0.0020617	0.003457	48019	20	22	42
SGP	2003	country	6_Wpr	Singapore	63	91	154	0.0013297	0.0019207	0.0032505	47378	20	20	40
SGP	2004	country	6_Wpr	Singapore	60	84	144	0.0012838	0.0017973	0.0030811	46736	35	15	50
SGP	2005	country	6_Wpr	Singapore	58	80	138	0.0012515	0.0017263	0.0029778	46343	31	16	47
SGP	2006	country	6_Wpr	Singapore	57	78	135	0.0012298	0.0016828	0.0029126	46350	18	34	52
SGP	2007	country	6_Wpr	Singapore	57	77	134	0.0012121	0.0016374	0.0028496	47024	19	23	42
SGP	2008	country	6_Wpr	Singapore	56	79	135	0.0011691	0.0016492	0.0028183	47901	19	24	43
SGP	2009	country	6_Wpr	Singapore	56	80	136	0.0011596	0.0016566	0.0028162	48292	18	17	34
SGP	2010	country	6_Wpr	Singapore	55	83	138	0.0011242	0.0016965	0.0028208	48923	24	22	46
SGP	2011	country	6_Wpr	Singapore	54	84	138	0.001087	0.0016909	0.0027778	49679	15	27	42
SGP	2012	country	6_Wpr	Singapore	54	85	139	0.0010717	0.0016869	0.0027585	50389	21	25	46
SGP	2013	country	6_Wpr	Singapore	53	86	139	0.0010504	0.0017044	0.0027548	50457	17	17	34
SGP	2014	country	6_Wpr	Singapore	52	87	139	0.0010299	0.0017232	0.0027531	50488	12	31	43
SGP	2015	country	6_Wpr	Singapore	51	87	138	0.0010067	0.0017174	0.0027241	50659	18	36	54
SGP	2016	country	6_Wpr	Singapore	50	87	137	0.0009929	0.0017277	0.0027206	50356	21	37	59
SGP	2017	country	6_Wpr	Singapore	49	86	135	0.0009669	0.001697	0.0026638	50679	17	34	51
SGP	2018	country	6_Wpr	Singapore	47	85	132	0.0009356	0.0016921	0.0026277	50233	17	34	51
SGP	2019	country	6_Wpr	Singapore	46	82	128	0.0009109	0.0016238	0.0025347	50500	17	33	50
SLB	2000	country	6_Wpr	Solomon Islands	190	248	438	0.0129231	0.016868	0.0297911	14702	27	19	46
SLB	2001	country	6_Wpr	Solomon Islands	192	249	441	0.0128215	0.0166278	0.0294493	14975	27	17	45
SLB	2002	country	6_Wpr	Solomon Islands	194	250	444	0.0127343	0.0164102	0.0291446	15234	29	17	45
SLB	2003	country	6_Wpr	Solomon Islands	196	252	448	0.0126265	0.016234	0.0288605	15523	29	17	46
SLB	2004	country	6_Wpr	Solomon Islands	198	254	452	0.0124859	0.0160173	0.0285032	15858	30	18	48
SLB	2005	country	6_Wpr	Solomon Islands	199	256	455	0.0123164	0.0158442	0.0281607	16157	31	19	49
SLB	2006	country	6_Wpr	Solomon Islands	199	258	457	0.0121062	0.0156955	0.0278018	16438	31	21	52
SLB	2007	country	6_Wpr	Solomon Islands	199	259	458	0.0118596	0.0154353	0.0272949	16780	31	20	51
SLB	2008	country	6_Wpr	Solomon Islands	198	260	458	0.0115615	0.0151818	0.0267433	17126	33	19	52
SLB	2009	country	6_Wpr	Solomon Islands	198	260	458	0.0112553	0.0147796	0.0260349	17592	34	23	57
SLB	2010	country	6_Wpr	Solomon Islands	197	259	456	0.0109425	0.0143863	0.0253288	18003	35	22	57
SLB	2011	country	6_Wpr	Solomon Islands	196	258	454	0.0106137	0.0139711	0.0245848	18467	36	27	64
SLB	2012	country	6_Wpr	Solomon Islands	195	257	452	0.0103039	0.01358	0.0238839	18925	38	29	67
SLB	2013	country	6_Wpr	Solomon Islands	194	256	450	0.0099817	0.0131717	0.0231535	19436	38	30	68
SLB	2014	country	6_Wpr	Solomon Islands	192	255	447	0.0096664	0.0128382	0.0225046	19863	38	32	70
SLB	2015	country	6_Wpr	Solomon Islands	189	254	443	0.0093532	0.0125699	0.0219232	20207	39	32	71
SLB	2016	country	6_Wpr	Solomon Islands	187	251	438	0.0090554	0.0121545	0.0212099	20651	39	30	68
SLB	2017	country	6_Wpr	Solomon Islands	183	248	431	0.008772	0.0118878	0.0206598	20862	38	27	66
SLB	2018	country	6_Wpr	Solomon Islands	180	243	423	0.0085137	0.0114935	0.0200072	21142	38	29	67
SLB	2019	country	6_Wpr	Solomon Islands	176	240	416	0.0082314	0.0112246	0.019456	21382	38	23	60

iso3	year	level	whoreg6	whoname	nnd	pnd	u5d	nmr	pnmr	u5mr	Livebirths	Neonatal birth defects deaths	1-59 month birth defects deaths	Under five birth defects deaths
SLE	2000	country	1_Afr	Sierra Leone	10231	41607	51838	0.0492069	0.2001104	0.2493173	207918	348	10	357
SLE	2001	country	1_Afr	Sierra Leone	10332	38483	48815	0.04859	0.1809817	0.2295717	212637	362	14	376
SLE	2002	country	1_Afr	Sierra Leone	10452	37159	47611	0.0479557	0.1704932	0.2184489	217951	379	23	402
SLE	2003	country	1_Afr	Sierra Leone	10561	36417	46978	0.0472514	0.1629327	0.2101841	223506	403	38	441
SLE	2004	country	1_Afr	Sierra Leone	10639	32425	43064	0.046451	0.14157	0.1880211	229037	433	52	485
SLE	2005	country	1_Afr	Sierra Leone	10665	32441	43106	0.045537	0.1385149	0.1840519	234205	444	56	500
SLE	2006	country	1_Afr	Sierra Leone	10632	32455	43087	0.0445415	0.1359648	0.1805063	238699	465	56	521
SLE	2007	country	1_Afr	Sierra Leone	10510	31524	42034	0.0433688	0.1300807	0.1734495	242340	487	55	542
SLE	2008	country	1_Afr	Sierra Leone	10326	31072	41398	0.0421251	0.1267572	0.1688823	245127	498	75	573
SLE	2009	country	1_Afr	Sierra Leone	10073	30422	40495	0.0407753	0.1231467	0.163922	247037	500	84	584
SLE	2010	country	1_Afr	Sierra Leone	9779	28844	38623	0.0393963	0.1162046	0.1556009	248221	501	83	584
SLE	2011	country	1_Afr	Sierra Leone	9487	28330	37817	0.0381162	0.1138214	0.1519376	248897	519	105	624
SLE	2012	country	1_Afr	Sierra Leone	9216	27627	36843	0.036953	0.1107765	0.1477294	249398	519	127	647
SLE	2013	country	1_Afr	Sierra Leone	8977	25381	34358	0.0359122	0.1015376	0.1374497	249971	524	147	670
SLE	2014	country	1_Afr	Sierra Leone	8782	25716	34498	0.035022	0.1025539	0.1375759	250757	528	159	688
SLE	2015	country	1_Afr	Sierra Leone	8632	26479	35111	0.0342763	0.1051428	0.1394191	251836	540	167	707
SLE	2016	country	1_Afr	Sierra Leone	8427	22271	30698	0.0332931	0.0879858	0.1212789	253116	546	221	767
SLE	2017	country	1_Afr	Sierra Leone	8279	21193	29472	0.0325347	0.0832852	0.1158199	254467	544	225	769
SLE	2018	country	1_Afr	Sierra Leone	8136	20296	28432	0.031808	0.0793493	0.1111574	255785	533	269	802
SLE	2019	country	1_Afr	Sierra Leone	8013	19522	27535	0.0311788	0.0759618	0.1071406	257002	550	275	824
SLV	2000	country	2_Amr	El Salvador	2166	2727	4893	0.0147735	0.0185999	0.0333734	146614	297	485	782
SLV	2001	country	2_Amr	El Salvador	2023	2477	4500	0.014213	0.0174026	0.0316156	142335	288	425	712
SLV	2002	country	2_Amr	El Salvador	1892	2244	4136	0.0136805	0.0162257	0.0299062	138299	291	385	676
SLV	2003	country	2_Amr	El Salvador	1750	2057	3807	0.0129912	0.0152702	0.0282613	134707	284	355	639
SLV	2004	country	2_Amr	El Salvador	1622	1891	3513	0.0123299	0.0143747	0.0267046	131550	272	340	612
SLV	2005	country	2_Amr	El Salvador	1514	1739	3253	0.0117273	0.0134702	0.0251975	129100	271	321	593
SLV	2006	country	2_Amr	El Salvador	1419	1608	3027	0.0111641	0.0126511	0.0238152	127104	270	309	579
SLV	2007	country	2_Amr	El Salvador	1339	1488	2827	0.0106797	0.0118681	0.0225478	125378	267	296	563
SLV	2008	country	2_Amr	El Salvador	1269	1381	2650	0.0102349	0.0111382	0.0213731	123988	263	284	547
SLV	2009	country	2_Amr	El Salvador	1202	1290	2492	0.0097967	0.0105139	0.0203106	122695	255	274	529
SLV	2010	country	2_Amr	El Salvador	1142	1202	2344	0.0093871	0.0098803	0.0192675	121656	249	263	512
SLV	2011	country	2_Amr	El Salvador	1084	1129	2213	0.008978	0.0093507	0.0183286	120740	239	254	493
SLV	2012	country	2_Amr	El Salvador	1031	1064	2095	0.0085987	0.0088739	0.0174726	119902	232	248	480
SLV	2013	country	2_Amr	El Salvador	983	1010	1993	0.0082376	0.0084639	0.0167014	119331	227	242	468
SLV	2014	country	2_Amr	El Salvador	940	958	1898	0.0079006	0.0080519	0.0159524	118979	216	234	450
SLV	2015	country	2_Amr	El Salvador	901	915	1816	0.0075964	0.0077144	0.0153109	118609	207	229	435
SLV	2016	country	2_Amr	El Salvador	863	881	1744	0.0073006	0.0074529	0.0147535	118210	201	224	426
SLV	2017	country	2_Amr	El Salvador	829	849	1678	0.0070354	0.0072052	0.0142406	117832	196	219	415
SLV	2018	country	2_Amr	El Salvador	796	815	1611	0.0067912	0.0069533	0.0137446	117210	191	214	405
SLV	2019	country	2_Amr	El Salvador	765	787	1552	0.0065613	0.00675	0.0133114	116592	183	209	393

iso3	year	level	whoreg6	whoname	nnd	pnd	u5d	nmr	pnmr	u5mr	Livebirths	Neonatal birth defects deaths	1-59 month birth defects deaths	Under five birth defects deaths
SMR	2000	country	4_Eur	San Marino	1	1	2	0.0030423	0.0030423	0.0060846	329	1	0	1
SMR	2001	country	4_Eur	San Marino	1	0	1	0.0027778	0	0.0027778	360	1	0	1
SMR	2002	country	4_Eur	San Marino	1	0	1	0.0025287	0	0.0025287	395	1	0	1
SMR	2003	country	4_Eur	San Marino	1	0	1	0.0023043	0	0.0023043	434	1	0	1
SMR	2004	country	4_Eur	San Marino	1	0	1	0.0021039	0	0.0021039	475	1	0	1
SMR	2005	country	4_Eur	San Marino	1	0	1	0.0019167	0	0.0019167	522	1	0	1
SMR	2006	country	4_Eur	San Marino	0	1	1				0	0	0	0
SMR	2007	country	4_Eur	San Marino	0	1	1				0	0	0	0
SMR	2008	country	4_Eur	San Marino	0	1	1				0	0	0	0
SMR	2009	country	4_Eur	San Marino	0	1	1				0	0	0	0
SMR	2010	country	4_Eur	San Marino	0	1	1				0	0	0	0
SMR	2011	country	4_Eur	San Marino	0	1	1				0	0	0	0
SMR	2012	country	4_Eur	San Marino	0	1	1				0	0	0	0
SMR	2013	country	4_Eur	San Marino	0	1	1				0	0	0	0
SMR	2014	country	4_Eur	San Marino	0	1	1				0	0	0	0
SMR	2015	country	4_Eur	San Marino	0	1	1				0	0	0	0
SMR	2016	country	4_Eur	San Marino	0	1	1				0	0	0	0
SMR	2017	country	4_Eur	San Marino	0	0	0				0	0	0	0
SMR	2018	country	4_Eur	San Marino	0	0	0				0	0	0	0
SMR	2019	country	4_Eur	San Marino	0	0	0				0	0	0	0
SOM	2000	country	5_Emr	Somalia	19072	53185	72257	0.0439988	0.1226962	0.166695	433466	609	69	678
SOM	2001	country	5_Emr	Somalia	19686	53147	72833	0.0443974	0.1198605	0.164258	443404	628	97	725
SOM	2002	country	5_Emr	Somalia	20302	66461	86763	0.0447582	0.1465216	0.1912798	453593	659	196	854
SOM	2003	country	5_Emr	Somalia	20903	64721	85624	0.0450512	0.139489	0.1845402	463983	679	116	795
SOM	2004	country	5_Emr	Somalia	21434	73251	94685	0.0451695	0.1543671	0.1995367	474523	700	120	820
SOM	2005	country	5_Emr	Somalia	21941	60665	82606	0.0452394	0.1250826	0.1703221	484997	736	106	842
SOM	2006	country	5_Emr	Somalia	22351	51682	74033	0.0451413	0.1043793	0.1495206	495134	744	109	853
SOM	2007	country	5_Emr	Somalia	22702	53067	75769	0.0449702	0.1051192	0.1500893	504824	772	116	889
SOM	2008	country	5_Emr	Somalia	22903	52515	75418	0.0445532	0.1021564	0.1467095	514060	792	124	916
SOM	2009	country	5_Emr	Somalia	22948	51686	74634	0.0438805	0.0988316	0.1427121	522966	812	168	980
SOM	2010	country	5_Emr	Somalia	22984	49647	72631	0.0432204	0.093359	0.1365794	531786	826	191	1017
SOM	2011	country	5_Emr	Somalia	22959	64049	87008	0.0424498	0.1184229	0.1608727	540851	851	203	1055
SOM	2012	country	5_Emr	Somalia	22940	56078	79018	0.0416728	0.1018716	0.1435444	550479	858	214	1071
SOM	2013	country	5_Emr	Somalia	22952	51149	74101	0.0409133	0.0911771	0.1320904	560991	872	221	1093
SOM	2014	country	5_Emr	Somalia	23037	63788	86825	0.0402416	0.1114259	0.1516675	572467	894	228	1122
SOM	2015	country	5_Emr	Somalia	23141	57872	81013	0.0395558	0.0989225	0.1384783	585021	906	237	1143
SOM	2016	country	5_Emr	Somalia	23353	53043	76396	0.0390106	0.0886072	0.1276178	598632	944	246	1190
SOM	2017	country	5_Emr	Somalia	23417	62661	86078	0.038197	0.1022113	0.1404084	613058	956	257	1213
SOM	2018	country	5_Emr	Somalia	23601	58328	81928	0.0375758	0.0928647	0.1304405	628091	989	270	1258
SOM	2019	country	5_Emr	Somalia	23723	48449	72172	0.0368624	0.0752827	0.1121451	643556	983	279	1262

iso3	year	level	whoreg6	whoname	nnd	pnd	u5d	nmr	pnmr	u5mr	Livebirths	Neonatal birth defects deaths	1-59 month birth defects deaths	Under five birth defects deaths
SRB	2000	country	4_Eur	Serbia	918	601	1519	0.0077997	0.0051063	0.012906	117697	162	242	404
SRB	2001	country	4_Eur	Serbia	862	566	1428	0.0074862	0.0049155	0.0124017	115145	150	223	372
SRB	2002	country	4_Eur	Serbia	792	518	1310	0.0070384	0.0046034	0.0116419	112525	133	169	302
SRB	2003	country	4_Eur	Serbia	716	463	1179	0.0065154	0.0042132	0.0107286	109893	131	159	290
SRB	2004	country	4_Eur	Serbia	644	412	1056	0.0059942	0.0038348	0.009829	107437	132	174	306
SRB	2005	country	4_Eur	Serbia	583	369	952	0.0055459	0.0035102	0.0090561	105122	92	128	221
SRB	2006	country	4_Eur	Serbia	536	340	876	0.0052252	0.0033145	0.0085398	102579	94	115	209
SRB	2007	country	4_Eur	Serbia	506	322	828	0.0050505	0.003214	0.0082645	100188	89	103	192
SRB	2008	country	4_Eur	Serbia	484	308	792	0.0049516	0.003151	0.0081026	97746	88	122	210
SRB	2009	country	4_Eur	Serbia	463	294	757	0.0048651	0.0030893	0.0079544	95167	68	93	161
SRB	2010	country	4_Eur	Serbia	443	277	720	0.0047695	0.0029823	0.0077517	92882	72	90	162
SRB	2011	country	4_Eur	Serbia	423	259	682	0.0046573	0.0028517	0.007509	90825	74	77	151
SRB	2012	country	4_Eur	Serbia	402	243	645	0.0045267	0.0027363	0.007263	88806	69	53	122
SRB	2013	country	4_Eur	Serbia	382	227	609	0.0043697	0.0025967	0.0069664	87420	49	71	121
SRB	2014	country	4_Eur	Serbia	360	214	574	0.0041709	0.0024794	0.0066502	86313	68	63	132
SRB	2015	country	4_Eur	Serbia	337	204	541	0.0039515	0.002392	0.0063435	85284	41	67	108
SRB	2016	country	4_Eur	Serbia	316	196	512	0.0037352	0.0023167	0.0060519	84601	44	69	113
SRB	2017	country	4_Eur	Serbia	297	190	487	0.0035385	0.0022637	0.0058022	83934	45	62	106
SRB	2018	country	4_Eur	Serbia	280	183	463	0.0033629	0.0021979	0.0055608	83261	38	59	98
SRB	2019	country	4_Eur	Serbia	265	176	441	0.0032192	0.0021381	0.0053573	82317	38	57	95
SSD	2000	country	1_Afr	South Sudan	14829	31593	46422	0.0559637	0.1192306	0.1751943	264976	387	41	428
SSD	2001	country	1_Afr	South Sudan	14821	30594	45415	0.054449	0.1123965	0.1668454	272200	394	31	425
SSD	2002	country	1_Afr	South Sudan	14887	29455	44342	0.0530769	0.1050175	0.1580944	280480	403	37	440
SSD	2003	country	1_Afr	South Sudan	14924	28384	43308	0.0515368	0.0980176	0.1495543	289580	420	49	469
SSD	2004	country	1_Afr	South Sudan	15043	26356	41399	0.0502638	0.088063	0.1383268	299281	438	59	497
SSD	2005	country	1_Afr	South Sudan	15176	25012	40188	0.0490567	0.0808509	0.1299077	309356	458	104	562
SSD	2006	country	1_Afr	South Sudan	15218	24121	39339	0.0476073	0.07546	0.1230673	319657	460	125	585
SSD	2007	country	1_Afr	South Sudan	15232	23323	38555	0.0461656	0.0706871	0.1168527	329943	482	156	638
SSD	2008	country	1_Afr	South Sudan	15220	22649	37869	0.0447696	0.0666228	0.1113924	339963	498	114	612
SSD	2009	country	1_Afr	South Sudan	15158	21905	37063	0.0433777	0.0626847	0.1060624	349442	516	132	648
SSD	2010	country	1_Afr	South Sudan	15028	21326	36354	0.0419804	0.0595734	0.1015538	357977	518	167	685
SSD	2011	country	1_Afr	South Sudan	14884	20841	35725	0.0407436	0.0570496	0.0977932	365309	525	172	697
SSD	2012	country	1_Afr	South Sudan	14761	20768	35529	0.0397473	0.0559219	0.0956692	371371	546	173	720
SSD	2013	country	1_Afr	South Sudan	14722	20981	35703	0.0391335	0.0557711	0.0949046	376200	554	255	809
SSD	2014	country	1_Afr	South Sudan	14799	20551	35350	0.0389618	0.0541052	0.093067	379834	562	162	724
SSD	2015	country	1_Afr	South Sudan	14883	21105	35988	0.0389147	0.0551846	0.0940993	382452	565	157	722
SSD	2016	country	1_Afr	South Sudan	14886	21716	36602	0.0387398	0.0565147	0.0952545	384256	596	149	745
SSD	2017	country	1_Afr	South Sudan	14888	21767	36655	0.038615	0.0564577	0.0950727	385550	594	149	743
SSD	2018	country	1_Afr	South Sudan	14905	22523	37428	0.0385463	0.0582467	0.096793	386678	614	233	846
SSD	2019	country	1_Afr	South Sudan	14976	23412	38388	0.0386046	0.06035	0.0989546	387933	617	154	771

iso3	year	level	whoreg6	whoname	nnd	pnd	u5d	nmr	pnmr	u5mr	Livebirths	Neonatal birth defects deaths	1-59 month birth defects deaths	Under five birth defects deaths
STP	2000	country	1_Afr	Sao Tome and Prin	128	310	438	0.0225774	0.0547207	0.0772981	5669	10	5	15
STP	2001	country	1_Afr	Sao Tome and Prin	128	305	433	0.0221677	0.052837	0.0750047	5774	10	5	15
STP	2002	country	1_Afr	Sao Tome and Prin	127	297	424	0.0216432	0.0506266	0.0722698	5868	10	11	20
STP	2003	country	1_Afr	Sao Tome and Prin	126	285	411	0.0210472	0.0476131	0.0686604	5987	10	7	17
STP	2004	country	1_Afr	Sao Tome and Prin	124	271	395	0.0203697	0.0445189	0.0648886	6087	10	13	23
STP	2005	country	1_Afr	Sao Tome and Prin	122	256	378	0.0197714	0.041505	0.0612764	6171	10	12	22
STP	2006	country	1_Afr	Sao Tome and Prin	120	244	364	0.0192808	0.0391258	0.0584066	6224	10	15	26
STP	2007	country	1_Afr	Sao Tome and Prin	119	230	349	0.0188751	0.036411	0.0552861	6305	10	16	27
STP	2008	country	1_Afr	Sao Tome and Prin	118	200	318	0.0185046	0.0313638	0.0498684	6377	11	19	30
STP	2009	country	1_Afr	Sao Tome and Prin	117	183	300	0.0181902	0.0283993	0.0465895	6432	11	16	28
STP	2010	country	1_Afr	Sao Tome and Prin	115	176	291	0.0178871	0.0273541	0.0452411	6429	11	18	29
STP	2011	country	1_Afr	Sao Tome and Prin	114	167	281	0.0175457	0.0257522	0.0432979	6497	12	16	28
STP	2012	country	1_Afr	Sao Tome and Prin	112	160	272	0.0171223	0.0244621	0.0415844	6541	12	16	28
STP	2013	country	1_Afr	Sao Tome and Prin	109	141	250	0.0166344	0.0214484	0.0380828	6553	12	16	29
STP	2014	country	1_Afr	Sao Tome and Prin	106	134	240	0.0161465	0.0204427	0.0365892	6565	12	17	29
STP	2015	country	1_Afr	Sao Tome and Prin	103	130	233	0.0156905	0.0197474	0.0354379	6564	12	17	29
STP	2016	country	1_Afr	Sao Tome and Prin	101	124	225	0.0152379	0.0186508	0.0338886	6628	13	16	29
STP	2017	country	1_Afr	Sao Tome and Prin	98	113	211	0.0148362	0.0171071	0.0319433	6605	13	15	28
STP	2018	country	1_Afr	Sao Tome and Prin	96	107	203	0.0144295	0.0160688	0.0304982	6653	13	16	28
STP	2019	country	1_Afr	Sao Tome and Prin	94	104	198	0.0140575	0.0155191	0.0295766	6687	13	15	28
SUR	2000	country	2_Amr	Suriname	193	155	348	0.0172336	0.0138404	0.031074	11199	29	13	43
SUR	2001	country	2_Amr	Suriname	193	142	335	0.0172389	0.0126835	0.0299224	11196	26	17	43
SUR	2002	country	2_Amr	Suriname	192	131	323	0.0172491	0.0117689	0.029018	11131	32	14	46
SUR	2003	country	2_Amr	Suriname	191	121	312	0.0171513	0.0108655	0.0280168	11136	25	16	41
SUR	2004	country	2_Amr	Suriname	188	113	301	0.0170269	0.0102343	0.0272612	11041	29	16	45
SUR	2005	country	2_Amr	Suriname	185	106	291	0.0168053	0.009629	0.0264343	11008	26	10	36
SUR	2006	country	2_Amr	Suriname	181	101	282	0.0165033	0.009209	0.0257123	10968	22	17	40
SUR	2007	country	2_Amr	Suriname	176	97	273	0.0161043	0.0088757	0.02498	10929	27	18	45
SUR	2008	country	2_Amr	Suriname	170	94	264	0.01566	0.0086591	0.024319	10856	37	13	50
SUR	2009	country	2_Amr	Suriname	165	92	257	0.0151938	0.0084717	0.0236655	10860	28	13	40
SUR	2010	country	2_Amr	Suriname	160	90	250	0.0147582	0.0083015	0.0230596	10841	26	16	42
SUR	2011	country	2_Amr	Suriname	155	88	243	0.0143366	0.0081395	0.0224761	10812	27	27	54
SUR	2012	country	2_Amr	Suriname	151	85	236	0.0139175	0.0078343	0.0217518	10850	26	9	35
SUR	2013	country	2_Amr	Suriname	146	84	230	0.013491	0.007762	0.021253	10822	25	16	41
SUR	2014	country	2_Amr	Suriname	141	83	224	0.0130824	0.007701	0.0207833	10778	22	13	34
SUR	2015	country	2_Amr	Suriname	137	81	218	0.0127162	0.0075183	0.0202345	10774	23	12	35
SUR	2016	country	2_Amr	Suriname	132	79	211	0.0123228	0.007375	0.0196978	10712	21	12	34
SUR	2017	country	2_Amr	Suriname	128	76	204	0.011967	0.0071054	0.0190723	10696	20	12	32
SUR	2018	country	2_Amr	Suriname	124	74	198	0.0115788	0.0069099	0.0184888	10709	20	11	31
SUR	2019	country	2_Amr	Suriname	119	72	191	0.0112152	0.0067856	0.0180008	10611	19	11	30

iso3	year	level	whoreg6	whoname	nnd	pnd	u5d	nmr	pnmr	u5mr	Livebirths	Neonatal birth defects deaths	1-59 month birth defects deaths	Under five birth defects deaths
SVK	2000	country	4_Eur	Slovakia	273	266	539	0.0050924	0.0049618	0.0100542	53609	83	64	147
SVK	2001	country	4_Eur	Slovakia	255	252	507	0.0048242	0.0047675	0.0095917	52858	95	51	146
SVK	2002	country	4_Eur	Slovakia	239	243	482	0.0045574	0.0046337	0.0091911	52442	93	67	159
SVK	2003	country	4_Eur	Slovakia	225	238	463	0.0042812	0.0045286	0.0088098	52555	77	58	135
SVK	2004	country	4_Eur	Slovakia	213	235	448	0.004019	0.0044342	0.0084532	52998	58	60	119
SVK	2005	country	4_Eur	Slovakia	204	233	437	0.0038012	0.0043416	0.0081428	53667	71	64	134
SVK	2006	country	4_Eur	Slovakia	198	229	427	0.0036339	0.0042028	0.0078366	54488	60	75	135
SVK	2007	country	4_Eur	Slovakia	193	226	419	0.0034912	0.0040881	0.0075793	55282	63	61	123
SVK	2008	country	4_Eur	Slovakia	191	220	411	0.0034224	0.0039421	0.0073645	55809	64	50	114
SVK	2009	country	4_Eur	Slovakia	191	211	402	0.0033804	0.0037343	0.0071147	56503	78	57	136
SVK	2010	country	4_Eur	Slovakia	190	204	394	0.0033557	0.0036029	0.0069586	56620	63	68	131
SVK	2011	country	4_Eur	Slovakia	189	196	385	0.0033313	0.0034547	0.006786	56735	66	62	128
SVK	2012	country	4_Eur	Slovakia	187	190	377	0.0032778	0.0033303	0.0066081	57051	58	54	112
SVK	2013	country	4_Eur	Slovakia	183	186	369	0.0032049	0.0032575	0.0064624	57100	70	70	140
SVK	2014	country	4_Eur	Slovakia	179	183	362	0.0031382	0.0032083	0.0063465	57039	61	64	125
SVK	2015	country	4_Eur	Slovakia	175	180	355	0.0030524	0.0031396	0.0061919	57333	60	61	121
SVK	2016	country	4_Eur	Slovakia	171	177	348	0.0029868	0.0030916	0.0060785	57251	61	60	120
SVK	2017	country	4_Eur	Slovakia	169	172	341	0.0029662	0.0030188	0.005985	56976	58	58	116
SVK	2018	country	4_Eur	Slovakia	166	168	334	0.0029319	0.0029672	0.0058992	56618	58	57	115
SVK	2019	country	4_Eur	Slovakia	164	161	325	0.00292	0.0028666	0.0057866	56164	57	54	112
SVN	2000	country	4_Eur	Slovenia	57	40	97	0.0032593	0.0022873	0.0055466	17488	18	19	38
SVN	2001	country	4_Eur	Slovenia	54	37	91	0.0031068	0.0021287	0.0052355	17381	14	20	34
SVN	2002	country	4_Eur	Slovenia	51	35	86	0.0029412	0.0020184	0.0049596	17340	20	15	35
SVN	2003	country	4_Eur	Slovenia	49	33	82	0.0027744	0.0018685	0.0046428	17662	17	7	24
SVN	2004	country	4_Eur	Slovenia	47	32	79	0.0025525	0.0017379	0.0042903	18414	14	13	27
SVN	2005	country	4_Eur	Slovenia	46	31	77	0.0024453	0.0016479	0.0040932	18812	11	10	21
SVN	2006	country	4_Eur	Slovenia	44	31	75	0.0022949	0.0016169	0.0039118	19173	9	11	20
SVN	2007	country	4_Eur	Slovenia	43	30	73	0.002157	0.0015049	0.0036619	19935	20	8	28
SVN	2008	country	4_Eur	Slovenia	41	31	72	0.0019815	0.0014982	0.0034797	20691	9	8	17
SVN	2009	country	4_Eur	Slovenia	40	30	70	0.0019098	0.0014324	0.0033422	20944	10	9	18
SVN	2010	country	4_Eur	Slovenia	38	29	67	0.0018014	0.0013748	0.0031762	21095	4	4	8
SVN	2011	country	4_Eur	Slovenia	36	29	65	0.0016548	0.001333	0.0029878	21755	8	9	17
SVN	2012	country	4_Eur	Slovenia	34	28	62	0.0015611	0.0012856	0.0028467	21780	15	9	23
SVN	2013	country	4_Eur	Slovenia	32	27	59	0.0014751	0.0012447	0.0027198	21693	12	1	14
SVN	2014	country	4_Eur	Slovenia	31	25	56	0.0014438	0.0011643	0.0026081	21471	10	10	20
SVN	2015	country	4_Eur	Slovenia	29	24	53	0.0013725	0.0011359	0.0025084	21129	8	5	13
SVN	2016	country	4_Eur	Slovenia	27	23	50	0.0013053	0.0011119	0.0024172	20685	9	5	14
SVN	2017	country	4_Eur	Slovenia	26	21	47	0.0012924	0.0010439	0.0023363	20117	8	4	13
SVN	2018	country	4_Eur	Slovenia	24	20	44	0.001186	0.0009883	0.0021743	20236	8	4	12
SVN	2019	country	4_Eur	Slovenia	23	19	42	0.0011873	0.0009808	0.0021681	19372	7	4	12

iso3	year	level	whoreg6	whoname	nnd	pnd	u5d	nmr	pnmr	u5mr	Livebirths	Neonatal birth defects deaths	1-59 month birth defects deaths	Under five birth defects deaths
SWE	2000	country	4_Eur	Sweden	213	166	379	0.002332	0.0018174	0.0041494	91338	92	78	171
SWE	2001	country	4_Eur	Sweden	213	162	375	0.0022969	0.0017469	0.0040438	92734	75	54	128
SWE	2002	country	4_Eur	Sweden	213	160	373	0.002243	0.0016849	0.0039279	94961	70	51	121
SWE	2003	country	4_Eur	Sweden	210	161	371	0.0021524	0.0016502	0.0038026	97564	73	46	119
SWE	2004	country	4_Eur	Sweden	205	162	367	0.0020456	0.0016165	0.0036621	100216	86	37	124
SWE	2005	country	4_Eur	Sweden	199	162	361	0.0019344	0.0015747	0.0035091	102875	67	46	113
SWE	2006	country	4_Eur	Sweden	195	161	356	0.0018459	0.0015241	0.00337	105638	71	56	127
SWE	2007	country	4_Eur	Sweden	192	158	350	0.0017785	0.0014636	0.0032421	107955	70	48	118
SWE	2008	country	4_Eur	Sweden	189	157	346	0.0017216	0.0014301	0.0031517	109784	61	53	114
SWE	2009	country	4_Eur	Sweden	185	158	343	0.0016619	0.0014193	0.0030812	111320	61	46	107
SWE	2010	country	4_Eur	Sweden	183	158	341	0.0016271	0.0014048	0.003032	112468	59	43	102
SWE	2011	country	4_Eur	Sweden	182	157	339	0.0015981	0.0013786	0.0029767	113884	55	37	92
SWE	2012	country	4_Eur	Sweden	183	155	338	0.0015986	0.001354	0.0029526	114477	47	43	90
SWE	2013	country	4_Eur	Sweden	185	152	337	0.0016062	0.0013197	0.0029259	115180	61	48	109
SWE	2014	country	4_Eur	Sweden	186	150	336	0.0016041	0.0012936	0.0028977	115953	53	45	98
SWE	2015	country	4_Eur	Sweden	185	148	333	0.0015877	0.0012702	0.0028579	116520	44	33	77
SWE	2016	country	4_Eur	Sweden	183	146	329	0.0015609	0.0012453	0.0028062	117241	53	50	103
SWE	2017	country	4_Eur	Sweden	178	144	322	0.0015125	0.0012236	0.0027361	117684	48	42	90
SWE	2018	country	4_Eur	Sweden	172	142	314	0.0014505	0.0011975	0.0026481	118577	46	41	87
SWE	2019	country	4_Eur	Sweden	165	140	305	0.0013815	0.0011722	0.0025537	119433	45	41	86
SWZ	2000	country	1_Afr	Eswatini	695	2829	3524	0.021571	0.0878047	0.1093757	32219	48	52	99
SWZ	2001	country	1_Afr	Eswatini	693	2931	3624	0.0214311	0.0906414	0.1120725	32336	49	52	101
SWZ	2002	country	1_Afr	Eswatini	688	3000	3688	0.0211816	0.0923615	0.113543	32481	49	52	101
SWZ	2003	country	1_Afr	Eswatini	680	3065	3745	0.0207915	0.0937147	0.1145062	32706	50	52	101
SWZ	2004	country	1_Afr	Eswatini	672	3112	3784	0.0204068	0.094503	0.1149099	32930	49	52	101
SWZ	2005	country	1_Afr	Eswatini	663	3142	3805	0.0200103	0.09483	0.1148403	33133	50	51	102
SWZ	2006	country	1_Afr	Eswatini	655	2898	3553	0.0197164	0.0872336	0.1069499	33221	50	60	109
SWZ	2007	country	1_Afr	Eswatini	650	2788	3438	0.0195693	0.0839373	0.1035066	33215	50	65	115
SWZ	2008	country	1_Afr	Eswatini	647	2734	3381	0.0195683	0.082689	0.1022573	33064	51	66	116
SWZ	2009	country	1_Afr	Eswatini	644	2519	3163	0.0196572	0.0768888	0.0965459	32762	51	72	124
SWZ	2010	country	1_Afr	Eswatini	641	2169	2810	0.019812	0.0670393	0.0868513	32354	51	82	134
SWZ	2011	country	1_Afr	Eswatini	636	1832	2468	0.0199358	0.057425	0.0773608	31902	52	77	129
SWZ	2012	country	1_Afr	Eswatini	630	1614	2244	0.0200485	0.0513622	0.0714107	31424	52	82	134
SWZ	2013	country	1_Afr	Eswatini	621	1503	2124	0.0199822	0.0483628	0.0683451	31078	52	97	149
SWZ	2014	country	1_Afr	Eswatini	611	1454	2065	0.0198731	0.0472922	0.0671654	30745	53	104	156
SWZ	2015	country	1_Afr	Eswatini	600	1210	1810	0.0196431	0.0396135	0.0592566	30545	52	96	147
SWZ	2016	country	1_Afr	Eswatini	588	1203	1791	0.0193792	0.0396482	0.0590274	30342	52	94	145
SWZ	2017	country	1_Afr	Eswatini	576	1308	1884	0.0190924	0.0433558	0.0624482	30169	51	85	136
SWZ	2018	country	1_Afr	Eswatini	562	1097	1659	0.0187578	0.0366145	0.0553723	29961	50	88	138
SWZ	2019	country	1_Afr	Eswatini	548	920	1468	0.0184217	0.0309269	0.0493486	29748	50	89	139

iso3	year	level	whoreg6	whoname	nnd	pnd	u5d	nmr	pnmr	u5mr	Livebirths	Neonatal birth defects deaths	1-59 month birth defects deaths	Under five birth defects deaths
SYC	2000	country	1_Afr	Seychelles	13	8	21	0.0086941	0.0053502	0.0140443	1495	3	2	5
SYC	2001	country	1_Afr	Seychelles	14	7	21	0.0086884	0.0043442	0.0130326	1611	3	2	5
SYC	2002	country	1_Afr	Seychelles	14	8	22	0.0086825	0.0049614	0.0136439	1612	3	2	5
SYC	2003	country	1_Afr	Seychelles	14	8	22	0.0086708	0.0049548	0.0136256	1615	3	2	5
SYC	2004	country	1_Afr	Seychelles	14	8	22	0.0086658	0.0049519	0.0136176	1616	3	2	5
SYC	2005	country	1_Afr	Seychelles	15	8	23	0.0086481	0.0046123	0.0132604	1734	3	2	5
SYC	2006	country	1_Afr	Seychelles	15	8	23	0.0086295	0.0046024	0.013232	1738	3	2	5
SYC	2007	country	1_Afr	Seychelles	15	8	23	0.008623	0.0045989	0.0132219	1740	3	2	5
SYC	2008	country	1_Afr	Seychelles	15	9	24	0.0086367	0.005182	0.0138186	1737	3	2	6
SYC	2009	country	1_Afr	Seychelles	15	9	24	0.0086665	0.0051999	0.0138664	1731	3	2	6
SYC	2010	country	1_Afr	Seychelles	15	9	24	0.0087072	0.0052243	0.0139315	1723	3	2	6
SYC	2011	country	1_Afr	Seychelles	15	9	24	0.0087487	0.0052492	0.013998	1715	3	2	6
SYC	2012	country	1_Afr	Seychelles	15	9	24	0.008805	0.005283	0.014088	1704	3	2	6
SYC	2013	country	1_Afr	Seychelles	15	9	24	0.0088719	0.0053231	0.014195	1691	3	2	6
SYC	2014	country	1_Afr	Seychelles	15	9	24	0.0089372	0.0053623	0.0142996	1678	3	2	6
SYC	2015	country	1_Afr	Seychelles	15	9	24	0.0089782	0.0053869	0.0143651	1671	3	2	6
SYC	2016	country	1_Afr	Seychelles	15	9	24	0.0089839	0.0053903	0.0143742	1670	3	2	6
SYC	2017	country	1_Afr	Seychelles	14	10	24	0.0089209	0.0063721	0.015293	1569	3	3	6
SYC	2018	country	1_Afr	Seychelles	14	9	23	0.0087984	0.0056561	0.0144544	1591	3	2	5
SYC	2019	country	1_Afr	Seychelles	14	9	23	0.00861	0.005535	0.014145	1626	3	2	5
SYR	2000	country	5_Emr	Syrian Arab Republ	6257	5335	11592	0.0122099	0.010411	0.0226209	512454	1075	1393	2468
SYR	2001	country	5_Emr	Syrian Arab Republ	6182	5155	11337	0.0118639	0.0098923	0.0217562	521077	1115	1421	2536
SYR	2002	country	5_Emr	Syrian Arab Republ	6136	5023	11159	0.0115415	0.0094476	0.0209891	531647	1131	1424	2555
SYR	2003	country	5_Emr	Syrian Arab Republ	6125	4974	11099	0.0112564	0.009142	0.0203985	544133	1150	1436	2586
SYR	2004	country	5_Emr	Syrian Arab Republ	6139	4913	11052	0.0110057	0.0088082	0.0198139	557804	1211	1454	2665
SYR	2005	country	5_Emr	Syrian Arab Republ	6155	4921	11076	0.0107824	0.0086202	0.0194026	570839	1235	1484	2719
SYR	2006	country	5_Emr	Syrian Arab Republ	6145	4979	11124	0.0105787	0.008572	0.0191507	580886	1289	1523	2812
SYR	2007	country	5_Emr	Syrian Arab Republ	6092	5052	11144	0.0103928	0.0086189	0.0190116	586177	1297	1566	2863
SYR	2008	country	5_Emr	Syrian Arab Republ	6009	5063	11072	0.0102604	0.0086458	0.0189062	585649	1286	1599	2885
SYR	2009	country	5_Emr	Syrian Arab Republ	5883	5109	10992	0.0101648	0.0088267	0.0189914	578763	1270	1625	2895
SYR	2010	country	5_Emr	Syrian Arab Republ	5725	5123	10848	0.0101159	0.0090529	0.0191688	565940	1230	1638	2868
SYR	2011	country	5_Emr	Syrian Arab Republ	5542	5271	10813	0.0101154	0.0096212	0.0197366	547878	1184	1634	2818
SYR	2012	country	5_Emr	Syrian Arab Republ	5371	6482	11853	0.010199	0.0123086	0.0225077	526618	1144	1605	2749
SYR	2013	country	5_Emr	Syrian Arab Republ	5187	6969	12156	0.0102801	0.013812	0.0240921	504567	1094	1570	2665
SYR	2014	country	5_Emr	Syrian Arab Republ	5016	7090	12106	0.010385	0.014678	0.025063	483003	1021	1531	2552
SYR	2015	country	5_Emr	Syrian Arab Republ	4860	6365	11225	0.0104846	0.013732	0.0242166	463538	1009	1492	2501
SYR	2016	country	5_Emr	Syrian Arab Republ	4742	6084	10826	0.010595	0.0135934	0.0241884	447568	972	1454	2426
SYR	2017	country	5_Emr	Syrian Arab Republ	4667	5657	10324	0.0107235	0.0129974	0.0237208	435214	956	1417	2372
SYR	2018	country	5_Emr	Syrian Arab Republ	4599	5038	9637	0.010783	0.0118122	0.0225952	426504	950	1381	2331
SYR	2019	country	5_Emr	Syrian Arab Republ	4545	4650	9195	0.0107774	0.0110264	0.0218038	421715	935	1353	2288

iso3	year	level	whoreg6	whoname	nnd	pnd	u5d	nmr	pnmr	u5mr	Livebirths	Neonatal birth defects deaths	1-59 month birth defects deaths	Under five birth defects deaths
TCD	2000	country	1_Afr	Chad	18979	53760	72739	0.0443579	0.1256493	0.1700072	427860	567	46	613
TCD	2001	country	1_Afr	Chad	19161	63149	82310	0.0433546	0.1428842	0.1862388	441960	569	73	643
TCD	2002	country	1_Afr	Chad	19347	60414	79761	0.0424224	0.1324698	0.1748923	456056	591	53	644
TCD	2003	country	1_Afr	Chad	19559	64114	83673	0.0416133	0.1364078	0.178021	470018	609	86	695
TCD	2004	country	1_Afr	Chad	19754	64390	84144	0.0408354	0.1331071	0.1739425	483747	626	79	706
TCD	2005	country	1_Afr	Chad	19956	61948	81904	0.0401421	0.1246098	0.1647519	497134	634	114	747
TCD	2006	country	1_Afr	Chad	20179	58462	78641	0.0395591	0.1146094	0.1541686	510097	658	108	766
TCD	2007	country	1_Afr	Chad	20431	57493	77924	0.0390921	0.1100054	0.1490976	522637	680	144	824
TCD	2008	country	1_Afr	Chad	20711	59805	80516	0.0387215	0.1118116	0.1505331	534871	685	140	825
TCD	2009	country	1_Afr	Chad	20984	57070	78054	0.0383776	0.1043755	0.1427531	546778	717	187	904
TCD	2010	country	1_Afr	Chad	21245	56524	77769	0.0380405	0.10121	0.1392506	558483	726	257	982
TCD	2011	country	1_Afr	Chad	21431	59187	80618	0.0375919	0.10382	0.1414119	570097	730	343	1074
TCD	2012	country	1_Afr	Chad	21597	57762	79359	0.0371283	0.0993017	0.13643	581686	750	307	1056
TCD	2013	country	1_Afr	Chad	21737	55272	77009	0.0366329	0.093149	0.1297819	593374	757	284	1041
TCD	2014	country	1_Afr	Chad	21866	56781	78647	0.0361309	0.093823	0.1299539	605189	781	224	1004
TCD	2015	country	1_Afr	Chad	21959	53936	75895	0.0355825	0.0873987	0.1229812	617130	790	243	1033
TCD	2016	country	1_Afr	Chad	22047	55150	77197	0.0350405	0.0876531	0.1226936	629187	818	201	1019
TCD	2017	country	1_Afr	Chad	22112	52594	74706	0.0344832	0.082019	0.1165022	641240	813	209	1022
TCD	2018	country	1_Afr	Chad	22137	52997	75134	0.0338878	0.0811289	0.1150167	653244	838	222	1060
TCD	2019	country	1_Afr	Chad	22123	52433	74556	0.03326	0.0788287	0.1120887	665154	847	231	1079
TGO	2000	country	1_Afr	Togo	7073	16666	23739	0.0361878	0.0852706	0.1214584	195453	331	59	390
TGO	2001	country	1_Afr	Togo	7117	15911	23028	0.0354022	0.0791463	0.1145485	201033	337	40	377
TGO	2002	country	1_Afr	Togo	7153	14292	21445	0.0346409	0.0692124	0.1038533	206490	344	51	394
TGO	2003	country	1_Afr	Togo	7180	14468	21648	0.0339085	0.0683292	0.1022377	211746	355	83	438
TGO	2004	country	1_Afr	Togo	7183	14462	21645	0.0331538	0.0667491	0.0999029	216657	358	81	439
TGO	2005	country	1_Afr	Togo	7176	14214	21390	0.032439	0.0642555	0.0966945	221215	359	78	437
TGO	2006	country	1_Afr	Togo	7160	14165	21325	0.0317717	0.0628537	0.0946254	225358	366	103	469
TGO	2007	country	1_Afr	Togo	7128	14079	21207	0.0311058	0.0614386	0.0925444	229154	373	105	477
TGO	2008	country	1_Afr	Togo	7086	14001	21087	0.0304524	0.0601697	0.090622	232691	369	116	485
TGO	2009	country	1_Afr	Togo	7035	13714	20749	0.0298102	0.0581101	0.0879203	235993	363	158	521
TGO	2010	country	1_Afr	Togo	6982	13551	20533	0.029202	0.0566776	0.0858797	239093	359	185	544
TGO	2011	country	1_Afr	Togo	6930	13186	20116	0.028636	0.054485	0.083121	242003	359	214	572
TGO	2012	country	1_Afr	Togo	6876	13037	19913	0.0280964	0.0532692	0.0813656	244729	360	197	557
TGO	2013	country	1_Afr	Togo	6833	12914	19747	0.0276168	0.0521948	0.0798116	247422	357	173	530
TGO	2014	country	1_Afr	Togo	6782	12355	19137	0.0271246	0.0494152	0.0765398	250031	355	228	584
TGO	2015	country	1_Afr	Togo	6738	12107	18845	0.0266617	0.047906	0.0745677	252722	360	282	643
TGO	2016	country	1_Afr	Togo	6694	11763	18457	0.0262035	0.0460474	0.0722509	255462	360	512	872
TGO	2017	country	1_Afr	Togo	6653	11511	18164	0.0257526	0.0445586	0.0703113	258342	360	439	799
TGO	2018	country	1_Afr	Togo	6612	11299	17911	0.0253009	0.0432361	0.068537	261335	363	389	753
TGO	2019	country	1_Afr	Togo	6563	10725	17288	0.0248118	0.0405483	0.0653601	264511	363	388	751

iso3	year	level	whoreg6	whoname	nnd	pnd	u5d	nmr	pnmr	u5mr	Livebirths	Neonatal birth defects deaths	1-59 month birth defects deaths	Under five birth defects deaths
THA	2000	country	3_Sear	Thailand	11716	8569	20285	0.0128409	0.0093913	0.0222322	912397	2151	1289	3440
THA	2001	country	3_Sear	Thailand	10922	8041	18963	0.0122016	0.0089832	0.0211848	895132	2102	1254	3356
THA	2002	country	3_Sear	Thailand	10218	7557	17775	0.0116182	0.0085921	0.0202103	879481	2104	1232	3336
THA	2003	country	3_Sear	Thailand	9594	6991	16585	0.0110855	0.0080778	0.0191632	865458	2040	1229	3268
THA	2004	country	3_Sear	Thailand	9009	6533	15542	0.0105625	0.0076591	0.0182216	852921	1969	1225	3194
THA	2005	country	3_Sear	Thailand	8472	6130	14602	0.0100643	0.0072819	0.0173462	841784	1945	1258	3203
THA	2006	country	3_Sear	Thailand	7970	5770	13740	0.0095872	0.0069409	0.0165282	831314	1790	1256	3046
THA	2007	country	3_Sear	Thailand	7511	5426	12937	0.0091449	0.0066067	0.0157516	821332	1736	1231	2967
THA	2008	country	3_Sear	Thailand	7079	5167	12246	0.008727	0.00637	0.015097	811158	1709	1228	2937
THA	2009	country	3_Sear	Thailand	6670	4855	11525	0.0083291	0.0060622	0.0143912	800811	1613	1220	2833
THA	2010	country	3_Sear	Thailand	6284	4545	10829	0.0079502	0.0057503	0.0137005	790421	1571	1186	2756
THA	2011	country	3_Sear	Thailand	5922	4278	10200	0.0075918	0.0054846	0.0130764	780050	1500	1151	2650
THA	2012	country	3_Sear	Thailand	5594	4056	9650	0.0072622	0.0052661	0.0125283	770288	1441	1107	2548
THA	2013	country	3_Sear	Thailand	5288	3803	9091	0.0069463	0.0049954	0.0119417	761271	1405	1067	2473
THA	2014	country	3_Sear	Thailand	4992	3577	8569	0.0066316	0.004752	0.0113836	752756	1351	1031	2381
THA	2015	country	3_Sear	Thailand	4723	3363	8086	0.0063443	0.0045174	0.0108618	744442	1283	997	2281
THA	2016	country	3_Sear	Thailand	4469	3170	7639	0.0060702	0.004306	0.0103762	736221	1217	958	2175
THA	2017	country	3_Sear	Thailand	4219	2999	7218	0.0058003	0.0041234	0.0099237	727379	1158	921	2079
THA	2018	country	3_Sear	Thailand	3980	2900	6880	0.0055444	0.0040397	0.0095842	717836	1102	882	1983
THA	2019	country	3_Sear	Thailand	3759	2709	6468	0.0053116	0.0038274	0.009139	707697	1031	841	1872
TJK	2000	country	4_Eur	Tajikistan	5417	10472	15889	0.0284013	0.0549047	0.083306	190731	502	659	1161
TJK	2001	country	4_Eur	Tajikistan	5193	9412	14605	0.0272658	0.0494176	0.0766834	190458	493	650	1143
TJK	2002	country	4_Eur	Tajikistan	4970	8490	13460	0.0259587	0.0443439	0.0703025	191458	477	655	1132
TJK	2003	country	4_Eur	Tajikistan	4807	7658	12465	0.0248097	0.0395241	0.0643338	193755	478	668	1146
TJK	2004	country	4_Eur	Tajikistan	4708	6955	11663	0.023842	0.0352211	0.0590631	197467	465	665	1130
TJK	2005	country	4_Eur	Tajikistan	4682	6327	11009	0.0231254	0.0312504	0.0543758	202461	475	550	1025
TJK	2006	country	4_Eur	Tajikistan	4708	5825	10533	0.0225656	0.0279194	0.050485	208636	479	500	979
TJK	2007	country	4_Eur	Tajikistan	4769	5454	10223	0.0221119	0.0252879	0.0473998	215676	501	526	1027
TJK	2008	country	4_Eur	Tajikistan	4822	5206	10028	0.0215999	0.02332	0.0449199	223242	506	542	1048
TJK	2009	country	4_Eur	Tajikistan	4841	5104	9945	0.0209621	0.0221009	0.043063	230941	523	591	1114
TJK	2010	country	4_Eur	Tajikistan	4820	5099	9919	0.0201931	0.021362	0.0415551	238695	519	670	1190
TJK	2011	country	4_Eur	Tajikistan	4748	5196	9944	0.0192801	0.0210993	0.0403794	246264	517	748	1265
TJK	2012	country	4_Eur	Tajikistan	4652	5340	9992	0.0183411	0.0210536	0.0393947	253638	522	712	1233
TJK	2013	country	4_Eur	Tajikistan	4550	5495	10045	0.0174606	0.021087	0.0385475	260587	525	705	1230
TJK	2014	country	4_Eur	Tajikistan	4471	5598	10069	0.0167504	0.0209726	0.037723	266920	526	810	1336
TJK	2015	country	4_Eur	Tajikistan	4413	5641	10054	0.0162132	0.0207249	0.0369381	272185	535	813	1348
TJK	2016	country	4_Eur	Tajikistan	4368	5617	9985	0.0158186	0.0203418	0.0361604	276131	545	807	1352
TJK	2017	country	4_Eur	Tajikistan	4335	5503	9838	0.0155612	0.019754	0.0353152	278577	546	806	1352
TJK	2018	country	4_Eur	Tajikistan	4277	5359	9636	0.0152979	0.019168	0.034466	279580	545	779	1323
TJK	2019	country	4_Eur	Tajikistan	4183	5194	9377	0.0149777	0.0185977	0.0335754	279282	540	750	1290

iso3	year	level	whoreg6	whoname	nnd	pnd	u5d	nmr	pnmr	u5mr	Livebirths	Neonatal birth defects deaths	1-59 month birth defects deaths	Under five birth defects deaths
TKM	2000	country	4_Eur	Turkmenistan	3171	4312	7483	0.0297293	0.0404265	0.0701558	106663	329	457	786
TKM	2001	country	4_Eur	Turkmenistan	3099	3917	7016	0.0291173	0.036803	0.0659203	106432	321	456	777
TKM	2002	country	4_Eur	Turkmenistan	3030	3562	6592	0.0283955	0.0333811	0.0617766	106707	322	351	672
TKM	2003	country	4_Eur	Turkmenistan	2953	3265	6218	0.027543	0.0304531	0.0579961	107214	317	408	725
TKM	2004	country	4_Eur	Turkmenistan	2876	3011	5887	0.026627	0.0278769	0.0545038	108011	310	392	702
TKM	2005	country	4_Eur	Turkmenistan	2809	2798	5607	0.0256801	0.0255795	0.0512596	109384	312	393	705
TKM	2006	country	4_Eur	Turkmenistan	2771	2625	5396	0.0248252	0.0235172	0.0483424	111620	308	378	687
TKM	2007	country	4_Eur	Turkmenistan	2767	2506	5273	0.0240648	0.0217948	0.0458596	114981	307	367	675
TKM	2008	country	4_Eur	Turkmenistan	2802	2429	5231	0.0234733	0.0203486	0.0438219	119369	316	363	679
TKM	2009	country	4_Eur	Turkmenistan	2869	2396	5265	0.0230356	0.0192378	0.0422734	124547	331	364	695
TKM	2010	country	4_Eur	Turkmenistan	2959	2397	5356	0.0227517	0.0184305	0.0411823	130056	337	373	710
TKM	2011	country	4_Eur	Turkmenistan	3058	2435	5493	0.0226155	0.0180081	0.0406235	135217	354	385	739
TKM	2012	country	4_Eur	Turkmenistan	3147	2493	5640	0.0225496	0.0178634	0.040413	139559	366	397	763
TKM	2013	country	4_Eur	Turkmenistan	3226	2560	5786	0.0226279	0.0179564	0.0405843	142568	374	405	779
TKM	2014	country	4_Eur	Turkmenistan	3285	2618	5903	0.022789	0.0181618	0.0409508	144149	384	409	793
TKM	2015	country	4_Eur	Turkmenistan	3327	2655	5982	0.0230594	0.0184018	0.0414612	144280	393	407	800
TKM	2016	country	4_Eur	Turkmenistan	3350	2667	6017	0.023403	0.0186316	0.0420346	143144	382	401	783
TKM	2017	country	4_Eur	Turkmenistan	3338	2673	6011	0.0236246	0.018918	0.0425426	141294	387	394	780
TKM	2018	country	4_Eur	Turkmenistan	3297	2638	5935	0.0237058	0.0189675	0.0426733	139080	383	382	765
TKM	2019	country	4_Eur	Turkmenistan	3233	2560	5793	0.0236278	0.0187093	0.0423372	136830	375	366	740
TLS	2000	country	3_Sear	Timor-Leste	1312	2451	3763	0.0368194	0.0687839	0.1056033	35633	62	26	88
TLS	2001	country	3_Sear	Timor-Leste	1263	2342	3605	0.0351282	0.0651388	0.100267	35954	62	27	89
TLS	2002	country	3_Sear	Timor-Leste	1213	2224	3437	0.0335601	0.0615315	0.0950916	36144	63	26	89
TLS	2003	country	3_Sear	Timor-Leste	1159	2103	3262	0.0320333	0.0581243	0.0901576	36181	62	26	88
TLS	2004	country	3_Sear	Timor-Leste	1101	1976	3077	0.0305629	0.0548523	0.0854152	36024	63	24	87
TLS	2005	country	3_Sear	Timor-Leste	1044	1842	2886	0.029199	0.0515177	0.0807167	35755	61	21	82
TLS	2006	country	3_Sear	Timor-Leste	988	1709	2697	0.0279673	0.0483766	0.0763438	35327	61	29	90
TLS	2007	country	3_Sear	Timor-Leste	935	1577	2512	0.0268738	0.0453262	0.0722001	34792	59	30	89
TLS	2008	country	3_Sear	Timor-Leste	890	1456	2346	0.0259455	0.0424457	0.0683912	34303	60	36	96
TLS	2009	country	3_Sear	Timor-Leste	852	1350	2202	0.0251429	0.0398391	0.064982	33886	61	34	95
TLS	2010	country	3_Sear	Timor-Leste	822	1253	2075	0.0244838	0.0373215	0.0618053	33573	58	30	88
TLS	2011	country	3_Sear	Timor-Leste	797	1178	1975	0.0238031	0.0351819	0.058985	33483	60	29	89
TLS	2012	country	3_Sear	Timor-Leste	778	1115	1893	0.0231469	0.0331732	0.05632	33611	61	38	99
TLS	2013	country	3_Sear	Timor-Leste	766	1063	1829	0.0225407	0.0312804	0.0538212	33983	62	36	98
TLS	2014	country	3_Sear	Timor-Leste	759	1027	1786	0.0219802	0.0297413	0.0517215	34531	63	40	103
TLS	2015	country	3_Sear	Timor-Leste	755	996	1751	0.0214334	0.028275	0.0497084	35225	64	36	100
TLS	2016	country	3_Sear	Timor-Leste	754	970	1724	0.0209685	0.0269754	0.047944	35959	66	40	106
TLS	2017	country	3_Sear	Timor-Leste	752	945	1697	0.0204903	0.0257491	0.0462394	36700	69	42	112
TLS	2018	country	3_Sear	Timor-Leste	749	920	1669	0.0200406	0.0246159	0.0446565	37374	69	42	111
TLS	2019	country	3_Sear	Timor-Leste	742	903	1645	0.019581	0.0238297	0.0434107	37894	70	42	111

iso3	year	level	whoreg6	whoname	nnd	pnd	u5d	nmr	pnmr	u5mr	Livebirths	Neonatal birth defects deaths	1-59 month birth defects deaths	Under five birth defects deaths
TON	2000	country	6_Wpr	Tonga	22	28	50	0.0078036	0.0099318	0.0177354	2819	4	6	10
TON	2001	country	6_Wpr	Tonga	22	27	49	0.0079881	0.0098036	0.0177917	2754	4	6	10
TON	2002	country	6_Wpr	Tonga	21	28	49	0.0074995	0.0099994	0.0174989	2800	4	7	10
TON	2003	country	6_Wpr	Tonga	21	28	49	0.0073808	0.0098411	0.0172219	2845	4	7	10
TON	2004	country	6_Wpr	Tonga	21	28	49	0.0072962	0.0097282	0.0170244	2878	4	7	10
TON	2005	country	6_Wpr	Tonga	21	28	49	0.0072481	0.0096642	0.0169123	2897	4	7	11
TON	2006	country	6_Wpr	Tonga	21	28	49	0.0072382	0.009651	0.0168892	2901	4	7	11
TON	2007	country	6_Wpr	Tonga	21	28	49	0.0072616	0.0096822	0.0169438	2892	4	7	11
TON	2008	country	6_Wpr	Tonga	21	27	48	0.0073096	0.0093981	0.0167076	2873	4	6	10
TON	2009	country	6_Wpr	Tonga	21	27	48	0.0073803	0.009489	0.0168693	2845	4	6	10
TON	2010	country	6_Wpr	Tonga	20	28	48	0.0071213	0.0099699	0.0170912	2808	4	7	10
TON	2011	country	6_Wpr	Tonga	20	27	47	0.0071927	0.0097102	0.0169029	2781	4	6	10
TON	2012	country	6_Wpr	Tonga	20	26	46	0.007204	0.0093653	0.0165693	2776	4	6	10
TON	2013	country	6_Wpr	Tonga	20	26	46	0.0075061	0.009758	0.0172641	2664	4	6	10
TON	2014	country	6_Wpr	Tonga	20	25	45	0.0074273	0.0092841	0.0167114	2693	4	6	10
TON	2015	country	6_Wpr	Tonga	19	25	44	0.0072731	0.0095699	0.016843	2612	4	6	10
TON	2016	country	6_Wpr	Tonga	19	25	44	0.0075067	0.0098772	0.0173839	2531	4	6	10
TON	2017	country	6_Wpr	Tonga	19	24	43	0.0073145	0.0092393	0.0165538	2598	4	6	10
TON	2018	country	6_Wpr	Tonga	19	24	43	0.0075355	0.0095186	0.0170541	2521	4	6	10
TON	2019	country	6_Wpr	Tonga	19	23	42	0.0077797	0.0094176	0.0171973	2442	4	6	9
TTO	2000	country	2_Amr	Trinidad and Tobag	352	175	527	0.0188286	0.0093608	0.0281895	18695	35	30	65
TTO	2001	country	2_Amr	Trinidad and Tobag	352	173	525	0.0187301	0.0092054	0.0279355	18793	73	37	110
TTO	2002	country	2_Amr	Trinidad and Tobag	351	171	522	0.0185393	0.009032	0.0275713	18933	98	18	116
TTO	2003	country	2_Amr	Trinidad and Tobag	350	169	519	0.018252	0.0088131	0.0270651	19176	73	28	101
TTO	2004	country	2_Amr	Trinidad and Tobag	348	167	515	0.0179094	0.0085944	0.0265038	19431	70	37	107
TTO	2005	country	2_Amr	Trinidad and Tobag	345	165	510	0.0175654	0.0084008	0.0259662	19641	77	48	125
TTO	2006	country	2_Amr	Trinidad and Tobag	340	163	503	0.0171131	0.0082042	0.0253173	19868	100	39	138
TTO	2007	country	2_Amr	Trinidad and Tobag	333	162	495	0.0166437	0.0080969	0.0247406	20008	74	45	120
TTO	2008	country	2_Amr	Trinidad and Tobag	327	158	485	0.0162072	0.007831	0.0240382	20176	78	43	122
TTO	2009	country	2_Amr	Trinidad and Tobag	319	156	475	0.0157946	0.007724	0.0235186	20197	45	44	88
TTO	2010	country	2_Amr	Trinidad and Tobag	310	152	462	0.015323	0.0075132	0.0228363	20231	53	37	90
TTO	2011	country	2_Amr	Trinidad and Tobag	298	149	447	0.0148093	0.0074046	0.0222139	20123	61	50	112
TTO	2012	country	2_Amr	Trinidad and Tobag	287	144	431	0.0143341	0.007192	0.0215262	20022	65	50	116
TTO	2013	country	2_Amr	Trinidad and Tobag	275	139	414	0.0138988	0.0070252	0.0209241	19786	55	43	98
TTO	2014	country	2_Amr	Trinidad and Tobag	263	135	398	0.0135043	0.0069319	0.0204361	19475	56	42	97
TTO	2015	country	2_Amr	Trinidad and Tobag	251	130	381	0.0130755	0.0067722	0.0198476	19196	54	40	94
TTO	2016	country	2_Amr	Trinidad and Tobag	238	125	363	0.012638	0.0066376	0.0192756	18832	50	39	88
TTO	2017	country	2_Amr	Trinidad and Tobag	226	120	346	0.0122697	0.0065149	0.0187846	18419	48	37	85
TTO	2018	country	2_Amr	Trinidad and Tobag	214	115	329	0.0118945	0.0063919	0.0182864	17991	45	36	81
TTO	2019	country	2_Amr	Trinidad and Tobag	202	110	312	0.0115339	0.0062808	0.0178148	17514	43	34	77

iso3	year	level	whoreg6	whoname	nnd	pnd	u5d	nmr	pnmr	u5mr	Livebirths	Neonatal birth defects deaths	1-59 month birth defects deaths	Under five birth defects deaths
TUN	2000	country	5_Emr	Tunisia	3155	1913	5068	0.0188132	0.0114072	0.0302204	167701	374	369	742
TUN	2001	country	5_Emr	Tunisia	2940	1714	4654	0.0177362	0.0103401	0.0280763	165763	385	340	725
TUN	2002	country	5_Emr	Tunisia	2753	1563	4316	0.0167165	0.0094907	0.0262072	164687	394	327	721
TUN	2003	country	5_Emr	Tunisia	2604	1433	4037	0.0158326	0.0087128	0.0245454	164471	405	319	724
TUN	2004	country	5_Emr	Tunisia	2489	1327	3816	0.0150646	0.0080316	0.0230962	165222	424	300	725
TUN	2005	country	5_Emr	Tunisia	2407	1239	3646	0.0144006	0.0074127	0.0218132	167146	436	283	719
TUN	2006	country	5_Emr	Tunisia	2358	1169	3527	0.0138326	0.0068576	0.0206903	170467	458	270	729
TUN	2007	country	5_Emr	Tunisia	2337	1118	3455	0.013357	0.0063899	0.0197469	174965	475	260	735
TUN	2008	country	5_Emr	Tunisia	2344	1080	3424	0.0129829	0.0059819	0.0189648	180545	495	252	747
TUN	2009	country	5_Emr	Tunisia	2368	1059	3427	0.0126842	0.0056725	0.0183567	186689	517	247	764
TUN	2010	country	5_Emr	Tunisia	2405	1048	3453	0.0124642	0.0054314	0.0178956	192952	530	243	774
TUN	2011	country	5_Emr	Tunisia	2445	1043	3488	0.0122998	0.0052469	0.0175467	198784	550	241	790
TUN	2012	country	5_Emr	Tunisia	2483	1044	3527	0.0121935	0.0051269	0.0173204	203633	570	243	813
TUN	2013	country	5_Emr	Tunisia	2511	1045	3556	0.0121273	0.005047	0.0171744	207053	583	241	824
TUN	2014	country	5_Emr	Tunisia	2525	1047	3572	0.0120797	0.0050089	0.0170886	209028	587	239	826
TUN	2015	country	5_Emr	Tunisia	2525	1046	3571	0.0120575	0.0049949	0.0170524	209414	591	236	826
TUN	2016	country	5_Emr	Tunisia	2511	1047	3558	0.0120595	0.0050284	0.0170879	208217	590	232	823
TUN	2017	country	5_Emr	Tunisia	2485	1044	3529	0.0120669	0.0050695	0.0171364	205936	588	228	816
TUN	2018	country	5_Emr	Tunisia	2442	1034	3476	0.0120314	0.0050944	0.0171258	202969	579	222	801
TUN	2019	country	5_Emr	Tunisia	2381	1017	3398	0.0119269	0.0050943	0.0170212	199633	568	215	783
TUR	2000	country	4_Eur	Turkey	25558	27248	52806	0.0186527	0.0198861	0.0385388	1370203	7153	6758	13911
TUR	2001	country	4_Eur	Turkey	23927	24881	48808	0.0175764	0.0182772	0.0358535	1361317	6694	6426	13120
TUR	2002	country	4_Eur	Turkey	22439	22566	45005	0.0166042	0.0166982	0.0333024	1351403	6295	6060	12355
TUR	2003	country	4_Eur	Turkey	21061	20377	41438	0.0156986	0.0151888	0.0308874	1341582	5883	5682	11565
TUR	2004	country	4_Eur	Turkey	19631	18521	38152	0.014734	0.0139009	0.0286349	1332361	5486	5353	10839
TUR	2005	country	4_Eur	Turkey	17991	17139	35130	0.013586	0.0129426	0.0265286	1324233	5088	5126	10213
TUR	2006	country	4_Eur	Turkey	16503	15846	32349	0.0125269	0.0120282	0.0245551	1317407	4550	4893	9444
TUR	2007	country	4_Eur	Turkey	15136	14710	29846	0.0115383	0.0112135	0.0227518	1311810	4236	4680	8916
TUR	2008	country	4_Eur	Turkey	13894	13673	27567	0.0106267	0.0104577	0.0210844	1307457	4068	4471	8539
TUR	2009	country	4_Eur	Turkey	12785	12743	25528	0.0098029	0.0097707	0.0195736	1304204	3255	3921	7175
TUR	2010	country	4_Eur	Turkey	11800	11876	23676	0.0090593	0.0091177	0.018177	1302524	3447	4325	7772
TUR	2011	country	4_Eur	Turkey	10913	11125	22038	0.0083784	0.0085412	0.0169195	1302517	3619	4231	7850
TUR	2012	country	4_Eur	Turkey	10125	10436	20561	0.0077639	0.0080023	0.0157662	1304119	2917	3587	6504
TUR	2013	country	4_Eur	Turkey	9421	9817	19238	0.0072097	0.0075128	0.0147225	1306707	2782	3420	6202
TUR	2014	country	4_Eur	Turkey	8821	9223	18044	0.0067334	0.0070403	0.0137737	1310033	2528	3280	5808
TUR	2015	country	4_Eur	Turkey	8322	8621	16943	0.0063382	0.0065659	0.012904	1313001	2306	3261	5568
TUR	2016	country	4_Eur	Turkey	7919	8004	15923	0.0060227	0.0060873	0.01211	1314868	2125	2981	5106
TUR	2017	country	4_Eur	Turkey	7579	7374	14953	0.0057632	0.0056073	0.0113706	1315062	2102	2719	4822
TUR	2018	country	4_Eur	Turkey	7246	6784	14030	0.0055196	0.0051677	0.0106873	1312774	1987	2502	4489
TUR	2019	country	4_Eur	Turkey	6905	6244	13149	0.0052781	0.0047729	0.010051	1308227	1887	2303	4190

iso3	year	level	whoreg6	whoname	nnd	pnd	u5d	nmr	pnmr	u5mr	Livebirths	Neonatal birth defects deaths	1-59 month birth defects deaths	Under five birth defects deaths
TUV	2000	country	6_Wpr	Tuvalu	6	4	10	0.0241724	0.0161149	0.0402874	248	1	0	1
TUV	2001	country	6_Wpr	Tuvalu	6	4	10	0.0239672	0.0159782	0.0399454	250	1	1	1
TUV	2002	country	6_Wpr	Tuvalu	6	4	10	0.0238201	0.0158801	0.0397002	252	1	1	1
TUV	2003	country	6_Wpr	Tuvalu	6	4	10	0.0236822	0.0157882	0.0394704	253	1	1	1
TUV	2004	country	6_Wpr	Tuvalu	6	4	10	0.0235435	0.0156957	0.0392392	255	1	1	1
TUV	2005	country	6_Wpr	Tuvalu	6	4	10	0.0232927	0.0155285	0.0388211	258	1	0	1
TUV	2006	country	6_Wpr	Tuvalu	6	3	9	0.0229555	0.0114777	0.0344332	261	1	0	1
TUV	2007	country	6_Wpr	Tuvalu	6	3	9	0.0225607	0.0112803	0.033841	266	1	0	1
TUV	2008	country	6_Wpr	Tuvalu	6	3	9	0.0221196	0.0110598	0.0331794	271	1	0	1
TUV	2009	country	6_Wpr	Tuvalu	6	3	9	0.0216074	0.0108037	0.0324112	278	1	0	1
TUV	2010	country	6_Wpr	Tuvalu	6	3	9	0.021109	0.0105545	0.0316635	284	1	0	1
TUV	2011	country	6_Wpr	Tuvalu	6	3	9	0.0205666	0.0102833	0.0308498	292	1	0	1
TUV	2012	country	6_Wpr	Tuvalu	6	2	8	0.0200412	0.0066804	0.0267215	299	1	0	1
TUV	2013	country	6_Wpr	Tuvalu	6	2	8	0.0195154	0.0065051	0.0260205	307	1	0	1
TUV	2014	country	6_Wpr	Tuvalu	5	3	8	0.0189205	0.0113523	0.0302728	264	1	1	1
TUV	2015	country	6_Wpr	Tuvalu	5	3	8	0.0183267	0.010996	0.0293227	273	1	1	1
TUV	2016	country	6_Wpr	Tuvalu	5	3	8	0.0176792	0.0106075	0.0282867	283	1	1	1
TUV	2017	country	6_Wpr	Tuvalu	5	2	7	0.0171058	0.0068423	0.0239481	292	1	0	1
TUV	2018	country	6_Wpr	Tuvalu	5	2	7	0.0165831	0.0066332	0.0232163	302	1	0	1
TUV	2019	country	6_Wpr	Tuvalu	5	2	7	0.0160294	0.0064118	0.0224412	312	1	0	1
TZA	2000	country	1_Afr	United Republic of	47036	125162	172198	0.0335516	0.0892801	0.1228317	1401902	2535	773	3307
TZA	2001	country	1_Afr	United Republic of	46661	119441	166102	0.0323996	0.0829352	0.1153348	1440173	2572	1044	3616
TZA	2002	country	1_Afr	United Republic of	46254	113391	159645	0.0312419	0.0765889	0.1078308	1480514	2610	1974	4584
TZA	2003	country	1_Afr	United Republic of	45883	107733	153616	0.0301427	0.0707748	0.1009174	1522195	2648	1881	4529
TZA	2004	country	1_Afr	United Republic of	45482	102829	148311	0.0290703	0.0657243	0.0947946	1564551	2681	2017	4698
TZA	2005	country	1_Afr	United Republic of	45231	98354	143585	0.0281533	0.0612188	0.0893721	1606598	2719	2115	4835
TZA	2006	country	1_Afr	United Republic of	45015	94519	139534	0.0273289	0.0573832	0.0847121	1647155	2757	2473	5230
TZA	2007	country	1_Afr	United Republic of	44828	90578	135406	0.0265932	0.0537333	0.0803265	1685695	2796	2531	5327
TZA	2008	country	1_Afr	United Republic of	44651	87282	131933	0.025931	0.050689	0.07662	1721914	2833	2636	5469
TZA	2009	country	1_Afr	United Republic of	44457	83433	127890	0.0253195	0.0475174	0.0728369	1755841	2871	3128	5999
TZA	2010	country	1_Afr	United Republic of	44220	80244	124464	0.0247317	0.0448795	0.0696111	1787990	2904	3466	6370
TZA	2011	country	1_Afr	United Republic of	44014	76636	120650	0.0241949	0.0421275	0.0663225	1819142	2941	3794	6735
TZA	2012	country	1_Afr	United Republic of	43789	73656	117445	0.0236636	0.0398038	0.0634674	1850477	2976	4413	7389
TZA	2013	country	1_Afr	United Republic of	43540	71078	114618	0.023124	0.0377494	0.0608735	1882889	3009	5252	8261
TZA	2014	country	1_Afr	United Republic of	43323	68400	111723	0.0226006	0.0356828	0.0582834	1916892	3041	5380	8421
TZA	2015	country	1_Afr	United Republic of	43162	66911	110073	0.0221036	0.0342657	0.0563693	1952711	3075	5441	8516
TZA	2016	country	1_Afr	United Republic of	43032	64968	108000	0.0216206	0.0326419	0.0542624	1990327	3108	4491	7598
TZA	2017	country	1_Afr	United Republic of	42920	63181	106101	0.0211506	0.031135	0.0522856	2029260	3140	5448	8588
TZA	2018	country	1_Afr	United Republic of	42820	61488	104308	0.0206948	0.0297169	0.0504117	2069122	3164	5419	8583
TZA	2019	country	1_Afr	United Republic of	42814	60408	103222	0.0202927	0.0286318	0.0489246	2109819	3189	5360	8548

iso3	year	level	whoreg6	whoname	nnd	pnd	u5d	nmr	pnmr	u5mr	Livebirths	Neonatal birth defects deaths	1-59 month birth defects deaths	Under five birth defects deaths
UGA	2000	country	1_Afr	Uganda	36959	131324	168283	0.0320544	0.113897	0.1459514	1153009	1759	259	2018
UGA	2001	country	1_Afr	Uganda	36823	125416	162239	0.031141	0.1060638	0.1372048	1182460	1877	245	2123
UGA	2002	country	1_Afr	Uganda	36691	125236	161927	0.0302577	0.1032777	0.1335354	1212617	1926	292	2218
UGA	2003	country	1_Afr	Uganda	36510	117058	153568	0.0293623	0.0941409	0.1235032	1243432	1900	566	2466
UGA	2004	country	1_Afr	Uganda	36430	102309	138739	0.0285798	0.0802624	0.1088422	1274677	2035	541	2576
UGA	2005	country	1_Afr	Uganda	36474	96315	132789	0.0279262	0.0737428	0.101669	1306087	2049	1150	3199
UGA	2006	country	1_Afr	Uganda	36594	90716	127310	0.0273626	0.0678313	0.0951939	1337372	2119	1563	3683
UGA	2007	country	1_Afr	Uganda	36684	85545	122229	0.0268142	0.0625291	0.0893432	1368082	2197	1626	3823
UGA	2008	country	1_Afr	Uganda	36722	80514	117236	0.0262701	0.0575976	0.0838676	1397864	2219	886	3105
UGA	2009	country	1_Afr	Uganda	36718	76091	112809	0.0257413	0.0533442	0.0790855	1426424	2314	965	3279
UGA	2010	country	1_Afr	Uganda	36708	71000	107708	0.0252559	0.0488498	0.0741057	1453441	2356	947	3303
UGA	2011	country	1_Afr	Uganda	36639	67136	103776	0.0247786	0.0454037	0.0701823	1478656	2400	1133	3534
UGA	2012	country	1_Afr	Uganda	36414	62178	98592	0.0242406	0.0413918	0.0656324	1502188	2407	1535	3942
UGA	2013	country	1_Afr	Uganda	36044	57729	93773	0.0236462	0.0378725	0.0615188	1524303	2456	1979	4434
UGA	2014	country	1_Afr	Uganda	35550	54034	89584	0.0230061	0.0349681	0.0579743	1545241	2434	2319	4753
UGA	2015	country	1_Afr	Uganda	34920	51007	85927	0.0223065	0.0325827	0.0548892	1565463	2476	1959	4435
UGA	2016	country	1_Afr	Uganda	34338	47537	81875	0.0216578	0.0299828	0.0516406	1585480	2529	1667	4196
UGA	2017	country	1_Afr	Uganda	33824	45246	79070	0.0210645	0.028178	0.0492425	1605733	2520	1452	3972
UGA	2018	country	1_Afr	Uganda	33349	43261	76610	0.0205047	0.0265992	0.0471039	1626409	2532	2090	4622
UGA	2019	country	1_Afr	Uganda	32914	41367	74281	0.0199779	0.0251089	0.0450868	1647522	2515	2050	4566
UKR	2000	country	4_Eur	Ukraine	4507	3065	7572	0.0111618	0.0075907	0.0187525	403786	1018	709	1727
UKR	2001	country	4_Eur	Ukraine	4253	2839	7092	0.0106202	0.0070893	0.0177095	400464	981	673	1654
UKR	2002	country	4_Eur	Ukraine	4068	2656	6724	0.0100899	0.0065877	0.0166776	403174	990	646	1636
UKR	2003	country	4_Eur	Ukraine	3940	2521	6461	0.0095875	0.0061345	0.015722	410952	973	627	1600
UKR	2004	country	4_Eur	Ukraine	3858	2433	6291	0.0091242	0.005754	0.0148782	422833	971	616	1587
UKR	2005	country	4_Eur	Ukraine	3815	2377	6192	0.0087206	0.0054335	0.0141541	437470	956	612	1568
UKR	2006	country	4_Eur	Ukraine	3791	2354	6145	0.008367	0.0051954	0.0135624	453091	973	616	1589
UKR	2007	country	4_Eur	Ukraine	3767	2343	6110	0.0080458	0.0050043	0.0130501	468196	969	627	1596
UKR	2008	country	4_Eur	Ukraine	3711	2330	6041	0.0077148	0.0048438	0.0125586	481026	967	643	1611
UKR	2009	country	4_Eur	Ukraine	3613	2299	5912	0.0073628	0.004685	0.0120478	490712	938	649	1587
UKR	2010	country	4_Eur	Ukraine	3477	2252	5729	0.0070099	0.0045402	0.0115502	496011	912	643	1555
UKR	2011	country	4_Eur	Ukraine	3314	2188	5502	0.0066756	0.0044074	0.0110831	496433	879	634	1513
UKR	2012	country	4_Eur	Ukraine	3144	2109	5253	0.006381	0.0042804	0.0106613	492716	844	626	1470
UKR	2013	country	4_Eur	Ukraine	2967	2028	4995	0.0061097	0.0041761	0.0102858	485620	779	611	1390
UKR	2014	country	4_Eur	Ukraine	2794	1939	4733	0.0058727	0.0040756	0.0099483	475760	733	589	1322
UKR	2015	country	4_Eur	Ukraine	2628	1846	4474	0.0056663	0.0039802	0.0096465	463795	689	566	1255
UKR	2016	country	4_Eur	Ukraine	2468	1750	4218	0.0054823	0.0038874	0.0093697	450175	650	546	1196
UKR	2017	country	4_Eur	Ukraine	2317	1654	3971	0.0053137	0.0037932	0.0091069	436043	618	521	1139
UKR	2018	country	4_Eur	Ukraine	2177	1559	3736	0.0051623	0.0036968	0.0088591	421711	589	493	1082
UKR	2019	country	4_Eur	Ukraine	2047	1467	3514	0.005013	0.0035926	0.0086055	408342	546	467	1013

iso3	year	level	whoreg6	whoname	nnd	pnd	u5d	nmr	pnmr	u5mr	Livebirths	Neonatal birth defects deaths	1-59 month birth defects deaths	Under five birth defects deaths
URY	2000	country	2_Amr	Uruguay	455	466	921	0.0084294	0.0086331	0.0170625	53978	112	127	239
URY	2001	country	2_Amr	Uruguay	446	441	887	0.00836	0.0082663	0.0166263	53349	117	118	235
URY	2002	country	2_Amr	Uruguay	435	422	857	0.0082682	0.0080211	0.0162894	52611	112	136	247
URY	2003	country	2_Amr	Uruguay	418	404	822	0.0080594	0.0077895	0.0158489	51865	114	112	226
URY	2004	country	2_Amr	Uruguay	394	384	778	0.0077248	0.0075287	0.0152535	51005	112	115	227
URY	2005	country	2_Amr	Uruguay	363	365	728	0.0072155	0.0072553	0.0144708	50308	107	100	207
URY	2006	country	2_Amr	Uruguay	330	345	675	0.0066605	0.0069632	0.0136237	49546	95	104	199
URY	2007	country	2_Amr	Uruguay	296	329	625	0.0060373	0.0067103	0.0127476	49029	73	81	154
URY	2008	country	2_Amr	Uruguay	267	313	580	0.0055078	0.0064568	0.0119646	48476	88	83	172
URY	2009	country	2_Amr	Uruguay	245	299	544	0.0050728	0.0061909	0.0112637	48297	73	81	154
URY	2010	country	2_Amr	Uruguay	232	284	516	0.0048263	0.0059081	0.0107345	48069	68	101	169
URY	2011	country	2_Amr	Uruguay	231	264	495	0.0048047	0.0054911	0.0102958	48078	72	91	163
URY	2012	country	2_Amr	Uruguay	238	241	479	0.004936	0.0049983	0.0099343	48217	78	92	169
URY	2013	country	2_Amr	Uruguay	245	218	463	0.0050838	0.0045236	0.0096074	48192	81	82	163
URY	2014	country	2_Amr	Uruguay	246	200	446	0.0050892	0.0041376	0.0092268	48337	74	71	144
URY	2015	country	2_Amr	Uruguay	241	185	426	0.0049942	0.0038337	0.008828	48256	75	84	159
URY	2016	country	2_Amr	Uruguay	231	174	405	0.0048056	0.0036198	0.0084254	48069	82	73	154
URY	2017	country	2_Amr	Uruguay	220	162	382	0.0045811	0.0033734	0.0079545	48023	71	66	137
URY	2018	country	2_Amr	Uruguay	209	150	359	0.0043678	0.0031348	0.0075026	47850	69	61	130
URY	2019	country	2_Amr	Uruguay	200	137	337	0.0041967	0.0028748	0.0070715	47656	67	56	123
USA	2000	country	2_Amr	United States of Am	18384	14926	33310	0.004642	0.0037689	0.0084109	3960330	4811	3622	8433
USA	2001	country	2_Amr	United States of Am	18456	14587	33043	0.0046235	0.0036543	0.0082778	3991760	4577	3618	8195
USA	2002	country	2_Amr	United States of Am	18634	14271	32905	0.0046243	0.0035415	0.0081658	4029605	4676	3542	8218
USA	2003	country	2_Amr	United States of Am	18778	14060	32838	0.0046154	0.0034557	0.0080711	4068585	4616	3438	8054
USA	2004	country	2_Amr	United States of Am	18760	14027	32787	0.0045699	0.003417	0.0079869	4105098	4683	3451	8135
USA	2005	country	2_Amr	United States of Am	18574	14101	32675	0.0044926	0.0034107	0.0079032	4134378	4415	3499	7914
USA	2006	country	2_Amr	United States of Am	18277	14167	32444	0.0044036	0.0034134	0.007817	4150462	4622	3478	8100
USA	2007	country	2_Amr	United States of Am	17920	14137	32057	0.0043159	0.0034048	0.0077208	4152053	4435	3561	7997
USA	2008	country	2_Amr	United States of Am	17520	13984	31504	0.004233	0.0033787	0.0076117	4138869	4359	3471	7829
USA	2009	country	2_Amr	United States of Am	17096	13709	30805	0.0041574	0.0033338	0.0074912	4112160	4240	3391	7631
USA	2010	country	2_Amr	United States of Am	16696	13325	30021	0.0040974	0.0032702	0.0073676	4074736	4271	3386	7658
USA	2011	country	2_Amr	United States of Am	16373	12834	29207	0.0040615	0.0031836	0.0072451	4031258	4221	3297	7518
USA	2012	country	2_Amr	United States of Am	16134	12295	28429	0.0040462	0.0030834	0.0071297	3987422	4151	3134	7285
USA	2013	country	2_Amr	United States of Am	15892	11835	27727	0.0040245	0.0029971	0.0070217	3948784	3869	3025	6894
USA	2014	country	2_Amr	United States of Am	15561	11567	27128	0.003971	0.0029518	0.0069227	3918674	3804	2951	6754
USA	2015	country	2_Amr	United States of Am	15201	11438	26639	0.0038977	0.0029328	0.0068304	3900036	3880	2852	6732
USA	2016	country	2_Amr	United States of Am	14929	11333	26262	0.0038338	0.0029103	0.0067441	3894049	3768	2830	6597
USA	2017	country	2_Amr	United States of Am	14781	11185	25966	0.0037911	0.0028688	0.0066598	3898888	3705	2812	6517
USA	2018	country	2_Amr	United States of Am	14681	10994	25675	0.0037529	0.0028104	0.0065634	3911866	3711	2763	6474
USA	2019	country	2_Amr	United States of Am	14546	10806	25352	0.0036997	0.0027485	0.0064482	3931631	3665	2716	6381

iso3	year	level	whoreg6	whoname	nnd	pnd	u5d	nmr	pnmr	u5mr	Livebirths	Neonatal birth defects deaths	1-59 month birth defects deaths	Under five birth defects deaths
UZB	2000	country	4_Eur	Uzbekistan	15846	19760	35606	0.028127	0.0350745	0.0632015	563373	1689	2489	4178
UZB	2001	country	4_Eur	Uzbekistan	15108	18063	33171	0.0272947	0.0326333	0.0599281	553514	1643	2360	4002
UZB	2002	country	4_Eur	Uzbekistan	14474	16501	30975	0.0263409	0.0300298	0.0563706	549488	1579	2150	3729
UZB	2003	country	4_Eur	Uzbekistan	13986	15176	29162	0.0253756	0.0275347	0.0529103	551159	1542	2134	3676
UZB	2004	country	4_Eur	Uzbekistan	13620	13999	27619	0.0243992	0.0250782	0.0494774	558215	1531	1973	3503
UZB	2005	country	4_Eur	Uzbekistan	13331	13026	26357	0.023389	0.0228539	0.046243	569968	1485	1919	3405
UZB	2006	country	4_Eur	Uzbekistan	13098	12154	25252	0.0223786	0.0207658	0.0431444	585290	1485	1798	3283
UZB	2007	country	4_Eur	Uzbekistan	12885	11353	24238	0.021387	0.0188442	0.0402312	602468	1524	1746	3269
UZB	2008	country	4_Eur	Uzbekistan	12632	10624	23256	0.0203782	0.0171388	0.037517	619879	1494	1713	3207
UZB	2009	country	4_Eur	Uzbekistan	12325	9921	22246	0.0193685	0.0155907	0.0349592	636341	1477	1602	3079
UZB	2010	country	4_Eur	Uzbekistan	11970	9216	21186	0.0183815	0.0141523	0.0325338	651200	1520	1653	3172
UZB	2011	country	4_Eur	Uzbekistan	11559	8549	20108	0.0173992	0.0128684	0.0302675	664342	1544	1600	3144
UZB	2012	country	4_Eur	Uzbekistan	11056	7957	19013	0.0163549	0.0117706	0.0281256	676004	1571	1501	3072
UZB	2013	country	4_Eur	Uzbekistan	10394	7537	17931	0.015145	0.0109821	0.0261271	686298	1564	1425	2989
UZB	2014	country	4_Eur	Uzbekistan	9734	7110	16844	0.0140118	0.0102346	0.0242464	694702	1582	1423	3005
UZB	2015	country	4_Eur	Uzbekistan	9095	6679	15774	0.012983	0.0095342	0.0225172	700533	1564	1391	2955
UZB	2016	country	4_Eur	Uzbekistan	8465	6249	14714	0.0120446	0.0088915	0.0209361	702804	1556	1358	2914
UZB	2017	country	4_Eur	Uzbekistan	7856	5879	13735	0.011199	0.0083807	0.0195798	701490	1558	1323	2881
UZB	2018	country	4_Eur	Uzbekistan	7316	5530	12846	0.010504	0.0079398	0.0184438	696495	1477	1282	2759
UZB	2019	country	4_Eur	Uzbekistan	6827	5218	12045	0.0099182	0.0075807	0.0174989	688329	1430	1241	2670
VCT	2000	country	2_Amr	Saint Vincent and th	28	21	49	0.0131628	0.0098721	0.0230349	2127	6	2	8
VCT	2001	country	2_Amr	Saint Vincent and th	28	19	47	0.0131351	0.0089131	0.0220482	2132	5	2	7
VCT	2002	country	2_Amr	Saint Vincent and th	27	19	46	0.013114	0.0092284	0.0223423	2059	0	1	1
VCT	2003	country	2_Amr	Saint Vincent and th	26	18	44	0.0130595	0.0090412	0.0221006	1991	1	1	2
VCT	2004	country	2_Amr	Saint Vincent and th	26	17	43	0.0129775	0.0084853	0.0214629	2003	5	3	7
VCT	2005	country	2_Amr	Saint Vincent and th	25	17	42	0.0129059	0.008776	0.0216819	1937	5	3	8
VCT	2006	country	2_Amr	Saint Vincent and th	24	17	41	0.0128619	0.0091105	0.0219724	1866	1	5	7
VCT	2007	country	2_Amr	Saint Vincent and th	24	16	40	0.0128308	0.0085539	0.0213847	1870	3	1	4
VCT	2008	country	2_Amr	Saint Vincent and th	23	15	38	0.0127856	0.0083385	0.0211241	1799	2	1	4
VCT	2009	country	2_Amr	Saint Vincent and th	23	14	37	0.0126915	0.0077252	0.0204167	1812	1	0	1
VCT	2010	country	2_Amr	Saint Vincent and th	22	13	35	0.012503	0.0073882	0.0198912	1760	3	3	6
VCT	2011	country	2_Amr	Saint Vincent and th	21	13	34	0.0122322	0.0075723	0.0198045	1717	3	7	10
VCT	2012	country	2_Amr	Saint Vincent and th	20	12	32	0.0118797	0.0071278	0.0190076	1684	0	3	3
VCT	2013	country	2_Amr	Saint Vincent and th	19	11	30	0.0114609	0.0066353	0.0180962	1658	3	1	4
VCT	2014	country	2_Amr	Saint Vincent and th	18	11	29	0.0110285	0.0067396	0.0177681	1632	4	2	6
VCT	2015	country	2_Amr	Saint Vincent and th	17	10	27	0.0105762	0.0062213	0.0167976	1607	4	3	7
VCT	2016	country	2_Amr	Saint Vincent and th	16	10	26	0.0101465	0.0063416	0.016488	1577	2	3	5
VCT	2017	country	2_Amr	Saint Vincent and th	15	10	25	0.0097533	0.0065022	0.0162555	1538	3	3	6
VCT	2018	country	2_Amr	Saint Vincent and th	15	9	24	0.0094136	0.0056481	0.0150617	1593	3	2	5
VCT	2019	country	2_Amr	Saint Vincent and th	14	9	23	0.0091006	0.0058504	0.014951	1538	3	2	5

iso3	year	level	whoreg6	whoname	nnd	pnd	u5d	nmr	pnmr	u5mr	Livebirths	Neonatal birth defects deaths	1-59 month birth defects deaths	Under five birth defects deaths
VEN	2000	country	2_Amr	Venezuela (Bolivari	6372	5989	12361	0.0110589	0.0103942	0.0214531	576188	897	954	1851
VEN	2001	country	2_Amr	Venezuela (Bolivari	6128	5892	12020	0.010593	0.0101851	0.0207781	578494	910	956	1866
VEN	2002	country	2_Amr	Venezuela (Bolivari	5841	5903	11744	0.0100559	0.0101627	0.0202186	580852	868	1056	1924
VEN	2003	country	2_Amr	Venezuela (Bolivari	5773	5685	11458	0.0098993	0.0097484	0.0196477	583173	764	787	1551
VEN	2004	country	2_Amr	Venezuela (Bolivari	5979	5146	11125	0.0102185	0.0087948	0.0190133	585116	894	898	1792
VEN	2005	country	2_Amr	Venezuela (Bolivari	6193	4593	10786	0.0105503	0.0078246	0.0183749	586996	899	968	1867
VEN	2006	country	2_Amr	Venezuela (Bolivari	6190	4295	10485	0.0105124	0.0072942	0.0178066	588826	999	935	1934
VEN	2007	country	2_Amr	Venezuela (Bolivari	6049	4205	10254	0.0102428	0.0071204	0.0173632	590561	965	978	1943
VEN	2008	country	2_Amr	Venezuela (Bolivari	5962	4152	10114	0.0100714	0.0070138	0.0170853	591972	933	949	1881
VEN	2009	country	2_Amr	Venezuela (Bolivari	6037	4034	10071	0.010182	0.0068037	0.0169857	592909	938	882	1820
VEN	2010	country	2_Amr	Venezuela (Bolivari	6226	3862	10088	0.0105079	0.0065181	0.017026	592506	1061	931	1992
VEN	2011	country	2_Amr	Venezuela (Bolivari	6461	3664	10125	0.0109472	0.0062081	0.0171552	590199	1045	890	1935
VEN	2012	country	2_Amr	Venezuela (Bolivari	6640	3534	10174	0.0113428	0.0060369	0.0173797	585395	1216	886	2101
VEN	2013	country	2_Amr	Venezuela (Bolivari	6652	3594	10246	0.0115024	0.0062146	0.017717	578316	1193	900	2093
VEN	2014	country	2_Amr	Venezuela (Bolivari	6450	3882	10332	0.0113331	0.006821	0.0181541	569127	1127	960	2087
VEN	2015	country	2_Amr	Venezuela (Bolivari	8067	2737	10804	0.0144486	0.0049022	0.0193507	558326	1444	673	2117
VEN	2016	country	2_Amr	Venezuela (Bolivari	7989	5426	13415	0.014616	0.0099269	0.0245429	546595	1419	1336	2755
VEN	2017	country	2_Amr	Venezuela (Bolivari	7835	5295	13130	0.0146461	0.0098981	0.0245442	534953	1388	1293	2681
VEN	2018	country	2_Amr	Venezuela (Bolivari	7657	5204	12861	0.0146042	0.0099256	0.0245298	524302	1363	1270	2632
VEN	2019	country	2_Amr	Venezuela (Bolivari	7520	5100	12620	0.0145924	0.0098964	0.0244888	515338	1335	1245	2581
VNM	2000	country	6_Wpr	Viet Nam	21505	22490	43995	0.0154227	0.0161293	0.031552	1394375	2819	2106	4925
VNM	2001	country	6_Wpr	Viet Nam	20327	20579	40906	0.0147578	0.0149409	0.0296987	1377371	2796	2013	4808
VNM	2002	country	6_Wpr	Viet Nam	19403	19316	38719	0.0141245	0.0140611	0.0281856	1373715	2828	1943	4771
VNM	2003	country	6_Wpr	Viet Nam	18771	17557	36328	0.0136072	0.0127274	0.0263346	1379490	2872	1924	4796
VNM	2004	country	6_Wpr	Viet Nam	18359	16530	34889	0.0131841	0.0118707	0.0250548	1392511	2870	1982	4852
VNM	2005	country	6_Wpr	Viet Nam	18148	16231	34379	0.0128644	0.0115059	0.0243703	1410710	2921	2027	4948
VNM	2006	country	6_Wpr	Viet Nam	18036	16282	34318	0.0125932	0.0113686	0.0239618	1432198	3016	2108	5124
VNM	2007	country	6_Wpr	Viet Nam	18039	16131	34170	0.0123939	0.0110831	0.0234771	1455468	3112	2196	5308
VNM	2008	country	6_Wpr	Viet Nam	18083	15947	34030	0.0122269	0.0107829	0.0230098	1478952	3202	2295	5496
VNM	2009	country	6_Wpr	Viet Nam	18158	16428	34586	0.0120958	0.0109431	0.0230388	1501186	3232	2394	5627
VNM	2010	country	6_Wpr	Viet Nam	18238	16361	34599	0.0119878	0.0107542	0.0227421	1521377	3298	2551	5848
VNM	2011	country	6_Wpr	Viet Nam	18278	15908	34186	0.0118695	0.0103307	0.0222002	1539908	3369	2667	6036
VNM	2012	country	6_Wpr	Viet Nam	18290	15944	34234	0.0117442	0.0102378	0.021982	1557366	3451	2754	6205
VNM	2013	country	6_Wpr	Viet Nam	18279	16027	34306	0.011614	0.0101832	0.0217972	1573874	3542	2805	6347
VNM	2014	country	6_Wpr	Viet Nam	18261	17998	36259	0.0114957	0.01133	0.0228257	1588504	3614	2909	6522
VNM	2015	country	6_Wpr	Viet Nam	18185	15724	33909	0.0113679	0.0098292	0.0211971	1599684	3684	2976	6660
VNM	2016	country	6_Wpr	Viet Nam	17959	15686	33645	0.0111856	0.00977	0.0209556	1605540	3641	3025	6666
VNM	2017	country	6_Wpr	Viet Nam	17621	15558	33179	0.0109776	0.0096926	0.0206702	1605178	3601	3063	6663
VNM	2018	country	6_Wpr	Viet Nam	17143	15416	32559	0.0107264	0.0096459	0.0203723	1598202	3540	3068	6608
VNM	2019	country	6_Wpr	Viet Nam	16587	16340	32927	0.0104648	0.0103088	0.0207735	1585032	3491	3069	6560

iso3	year	level	whoreg6	whoname	nnd	pnd	u5d	nmr	pnmr	u5mr	Livebirths	Neonatal birth defects deaths	1-59 month birth defects deaths	Under five birth defects deaths
VUT	2000	country	6_Wpr	Vanuatu	78	95	173	0.0125462	0.0152807	0.0278269	6217	12	9	21
VUT	2001	country	6_Wpr	Vanuatu	79	97	176	0.0124198	0.0151891	0.0276089	6361	12	10	22
VUT	2002	country	6_Wpr	Vanuatu	80	100	180	0.0123755	0.0154756	0.0278511	6464	12	9	21
VUT	2003	country	6_Wpr	Vanuatu	82	104	186	0.0124163	0.015702	0.0281183	6604	12	8	20
VUT	2004	country	6_Wpr	Vanuatu	84	108	192	0.0125434	0.016197	0.0287404	6697	13	9	22
VUT	2005	country	6_Wpr	Vanuatu	87	107	194	0.0126116	0.0154664	0.028078	6898	13	10	23
VUT	2006	country	6_Wpr	Vanuatu	89	109	198	0.0127285	0.0155906	0.0283192	6992	13	10	24
VUT	2007	country	6_Wpr	Vanuatu	92	111	203	0.0128676	0.015525	0.0283926	7150	14	11	25
VUT	2008	country	6_Wpr	Vanuatu	95	114	209	0.0129462	0.0155354	0.0284816	7338	14	11	25
VUT	2009	country	6_Wpr	Vanuatu	97	119	216	0.0129559	0.0159265	0.0288825	7487	14	12	27
VUT	2010	country	6_Wpr	Vanuatu	100	122	222	0.0130163	0.0158799	0.0288963	7683	15	13	28
VUT	2011	country	6_Wpr	Vanuatu	102	126	228	0.0130881	0.0161677	0.0292558	7793	15	15	30
VUT	2012	country	6_Wpr	Vanuatu	104	128	232	0.0130922	0.0161135	0.0292057	7944	16	16	32
VUT	2013	country	6_Wpr	Vanuatu	105	130	235	0.0130024	0.0160982	0.0291006	8075	16	17	33
VUT	2014	country	6_Wpr	Vanuatu	106	130	236	0.0128696	0.0157835	0.0286531	8236	16	18	34
VUT	2015	country	6_Wpr	Vanuatu	106	131	237	0.0126706	0.0156165	0.0282871	8366	16	16	33
VUT	2016	country	6_Wpr	Vanuatu	105	128	233	0.0124174	0.0151374	0.0275547	8456	17	17	34
VUT	2017	country	6_Wpr	Vanuatu	104	126	230	0.0121148	0.0146775	0.0267923	8585	17	17	34
VUT	2018	country	6_Wpr	Vanuatu	102	126	228	0.011746	0.0145098	0.0262558	8684	17	17	34
VUT	2019	country	6_Wpr	Vanuatu	100	124	224	0.0113938	0.0141283	0.0255222	8777	17	17	33
WSM	2000	country	6_Wpr	Samoa	61	49	110	0.011458	0.0092813	0.0207393	5324	9	6	15
WSM	2001	country	6_Wpr	Samoa	59	50	109	0.0110722	0.0093679	0.0204402	5329	9	6	15
WSM	2002	country	6_Wpr	Samoa	57	45	102	0.0107799	0.0084928	0.0192727	5288	9	6	15
WSM	2003	country	6_Wpr	Samoa	56	47	103	0.0105738	0.0088484	0.0194222	5296	9	6	15
WSM	2004	country	6_Wpr	Samoa	55	44	99	0.0104418	0.0083105	0.0187523	5267	9	6	14
WSM	2005	country	6_Wpr	Samoa	55	49	104	0.0103508	0.0092153	0.0195662	5314	9	6	15
WSM	2006	country	6_Wpr	Samoa	55	50	105	0.0103081	0.0092943	0.0196024	5336	9	6	14
WSM	2007	country	6_Wpr	Samoa	55	46	101	0.0102903	0.0085714	0.0188618	5345	9	6	14
WSM	2008	country	6_Wpr	Samoa	55	48	103	0.0102901	0.0090096	0.0192997	5345	9	6	15
WSM	2009	country	6_Wpr	Samoa	54	72	126	0.0102568	0.0137189	0.0239757	5265	9	6	15
WSM	2010	country	6_Wpr	Samoa	53	43	96	0.0101654	0.0082847	0.0184502	5214	9	6	15
WSM	2011	country	6_Wpr	Samoa	52	46	98	0.0100608	0.0089887	0.0190495	5169	9	6	15
WSM	2012	country	6_Wpr	Samoa	50	41	91	0.0099206	0.008215	0.0181356	5040	9	6	14
WSM	2013	country	6_Wpr	Samoa	49	38	87	0.0097315	0.0075449	0.0172764	5035	9	5	14
WSM	2014	country	6_Wpr	Samoa	47	37	84	0.0095101	0.0074874	0.0169974	4942	9	5	14
WSM	2015	country	6_Wpr	Samoa	45	37	82	0.0092756	0.0075482	0.0168238	4851	8	5	13
WSM	2016	country	6_Wpr	Samoa	44	34	78	0.0090295	0.0069269	0.0159564	4873	8	5	13
WSM	2017	country	6_Wpr	Samoa	42	33	75	0.0087799	0.0069595	0.0157394	4784	8	5	12
WSM	2018	country	6_Wpr	Samoa	41	27	68	0.0084857	0.0055983	0.0140839	4832	7	4	12
WSM	2019	country	6_Wpr	Samoa	39	63	102	0.008219	0.0132016	0.0214206	4745	7	4	11

iso3	year	level	whoreg6	whoname	nnd	pnd	u5d	nmr	pnmr	u5mr	Livebirths	Neonatal birth defects deaths	1-59 month birth defects deaths	Under five birth defects deaths
YEM	2000	country	5_Emr	Yemen	25454	42709	68163	0.0368245	0.0617879	0.0986124	691225	1296	718	2014
YEM	2001	country	5_Emr	Yemen	24963	36695	61658	0.0357537	0.0525576	0.0883113	698194	1327	768	2095
YEM	2002	country	5_Emr	Yemen	24483	34292	58775	0.0346584	0.0485436	0.083202	706410	1324	610	1933
YEM	2003	country	5_Emr	Yemen	24034	33721	57755	0.0335963	0.047137	0.0807334	715376	1337	653	1991
YEM	2004	country	5_Emr	Yemen	23554	33169	56723	0.0324867	0.0457481	0.0782348	725036	1382	858	2239
YEM	2005	country	5_Emr	Yemen	23105	30137	53242	0.0314139	0.0409747	0.0723886	735502	1371	863	2235
YEM	2006	country	5_Emr	Yemen	22658	28769	51427	0.0303341	0.0385156	0.0688496	746949	1382	727	2110
YEM	2007	country	5_Emr	Yemen	22256	24991	47247	0.0293086	0.0329104	0.062219	759368	1367	842	2208
YEM	2008	country	5_Emr	Yemen	21850	23681	45531	0.0282813	0.0306511	0.0589324	772595	1414	815	2228
YEM	2009	country	5_Emr	Yemen	21548	22620	44168	0.0274049	0.0287684	0.0561733	786283	1410	732	2142
YEM	2010	country	5_Emr	Yemen	21467	22142	43609	0.0268374	0.027681	0.0545184	799892	1427	765	2192
YEM	2011	country	5_Emr	Yemen	21636	22519	44155	0.0266133	0.0276997	0.054313	812976	1465	784	2249
YEM	2012	country	5_Emr	Yemen	21941	22924	44865	0.0265941	0.0277854	0.0543795	825031	1499	770	2269
YEM	2013	country	5_Emr	Yemen	22217	22682	44899	0.0265834	0.0271403	0.0537237	835747	1540	953	2493
YEM	2014	country	5_Emr	Yemen	22466	23300	45766	0.0265907	0.027578	0.0541687	844881	1560	907	2467
YEM	2015	country	5_Emr	Yemen	22712	24946	47658	0.0266443	0.0292657	0.05591	852414	1627	913	2540
YEM	2016	country	5_Emr	Yemen	22856	25085	47941	0.0266244	0.0292211	0.0558455	858462	1639	984	2623
YEM	2017	country	5_Emr	Yemen	23030	25456	48486	0.0266739	0.0294837	0.0561576	863391	1676	876	2553
YEM	2018	country	5_Emr	Yemen	23123	30220	53343	0.0266524	0.0348328	0.0614852	867577	1701	858	2560
YEM	2019	country	5_Emr	Yemen	23220	29491	52711	0.0266541	0.0338521	0.0605062	871160	1703	852	2555
ZAF	2000	country	1_Afr	South Africa	15288	57117	72405	0.0148948	0.0556475	0.0705423	1026399	895	1656	2551
ZAF	2001	country	1_Afr	South Africa	14919	59464	74383	0.0144204	0.057477	0.0718975	1034573	873	1514	2387
ZAF	2002	country	1_Afr	South Africa	14625	61923	76548	0.0139625	0.0591174	0.0730799	1047451	863	1350	2213
ZAF	2003	country	1_Afr	South Africa	14387	64787	79174	0.0135156	0.060863	0.0743786	1064478	835	1247	2082
ZAF	2004	country	1_Afr	South Africa	14202	68481	82683	0.013093	0.063133	0.076226	1084703	831	1289	2120
ZAF	2005	country	1_Afr	South Africa	14054	70649	84703	0.0126978	0.063832	0.0765299	1106803	851	1323	2174
ZAF	2006	country	1_Afr	South Africa	13939	72744	86683	0.0123426	0.0644123	0.0767549	1129344	767	1496	2263
ZAF	2007	country	1_Afr	South Africa	13833	69912	83745	0.0120207	0.0607524	0.0727731	1150768	831	1562	2392
ZAF	2008	country	1_Afr	South Africa	13711	63636	77347	0.0117222	0.0544054	0.0661277	1169659	912	1905	2817
ZAF	2009	country	1_Afr	South Africa	13569	55523	69092	0.0114493	0.0468497	0.0582989	1185140	879	2325	3204
ZAF	2010	country	1_Afr	South Africa	13396	47987	61383	0.0111968	0.040109	0.0513058	1196413	906	2354	3261
ZAF	2011	country	1_Afr	South Africa	13239	40673	53912	0.0110026	0.0338025	0.0448051	1203265	1127	2789	3916
ZAF	2012	country	1_Afr	South Africa	13111	36281	49392	0.0108673	0.0300721	0.0409395	1206461	1250	3076	4326
ZAF	2013	country	1_Afr	South Africa	13068	34331	47399	0.0108279	0.0284463	0.0392742	1206879	1257	3074	4332
ZAF	2014	country	1_Afr	South Africa	13125	32386	45511	0.0108922	0.0268766	0.0377688	1204993	1333	3478	4811
ZAF	2015	country	1_Afr	South Africa	13267	31096	44363	0.0110437	0.0258852	0.0369289	1201316	1373	3571	4944
ZAF	2016	country	1_Afr	South Africa	13434	29777	43211	0.0112304	0.0248929	0.0361233	1196219	1349	3369	4718
ZAF	2017	country	1_Afr	South Africa	13526	28931	42457	0.0113639	0.0243065	0.0356705	1190260	1377	2374	3751
ZAF	2018	country	1_Afr	South Africa	13551	27785	41336	0.0114451	0.0234671	0.0349123	1183997	1381	2528	3909
ZAF	2019	country	1_Afr	South Africa	13489	27083	40572	0.0114524	0.0229937	0.0344461	1177837	1368	2527	3894

iso3	year	level	whoreg6	whoname	nnd	pnd	u5d	nmr	pnmr	u5mr	Livebirths	Neonatal birth defects deaths	1-59 month birth defects deaths	Under five birth defects deaths
ZMB	2000	country	1_Afr	Zambia	15977	54968	70945	0.0339018	0.1166363	0.1505381	471273	818	329	1147
ZMB	2001	country	1_Afr	Zambia	15923	51440	67363	0.033004	0.1066218	0.1396258	482456	819	345	1164
ZMB	2002	country	1_Afr	Zambia	15705	49956	65661	0.031858	0.1013376	0.1331956	492969	828	412	1240
ZMB	2003	country	1_Afr	Zambia	15392	44478	59870	0.0306163	0.0884713	0.1190876	502739	825	496	1321
ZMB	2004	country	1_Afr	Zambia	15042	40835	55877	0.0293896	0.0797846	0.1091742	511813	834	634	1468
ZMB	2005	country	1_Afr	Zambia	14731	38197	52928	0.0283087	0.0734029	0.1017116	520369	837	761	1598
ZMB	2006	country	1_Afr	Zambia	14526	35874	50400	0.0274775	0.0678602	0.0953377	528651	828	868	1696
ZMB	2007	country	1_Afr	Zambia	14427	34227	48654	0.0268666	0.0637391	0.0906057	536987	859	1147	2006
ZMB	2008	country	1_Afr	Zambia	14399	32514	46913	0.0263936	0.0595989	0.0859925	545549	835	1064	1899
ZMB	2009	country	1_Afr	Zambia	14435	30722	45157	0.0260362	0.0554121	0.0814483	554420	871	1187	2058
ZMB	2010	country	1_Afr	Zambia	14498	31250	45748	0.0257331	0.0554668	0.0811999	563400	881	1384	2265
ZMB	2011	country	1_Afr	Zambia	14566	30072	44638	0.0254618	0.0525666	0.0780284	572073	891	1015	1905
ZMB	2012	country	1_Afr	Zambia	14630	28469	43099	0.0252142	0.0490657	0.07428	580228	906	974	1880
ZMB	2013	country	1_Afr	Zambia	14691	26830	41521	0.0249927	0.0456429	0.0706356	587813	902	900	1802
ZMB	2014	country	1_Afr	Zambia	14739	25830	40569	0.024773	0.043415	0.068188	594962	897	1126	2023
ZMB	2015	country	1_Afr	Zambia	14769	25611	40380	0.0245302	0.0425371	0.0670672	602075	918	1319	2236
ZMB	2016	country	1_Afr	Zambia	14790	24951	39741	0.0242546	0.0409183	0.0651729	609782	920	1466	2386
ZMB	2017	country	1_Afr	Zambia	14806	24063	38869	0.0239396	0.0389073	0.0628469	618473	936	1624	2560
ZMB	2018	country	1_Afr	Zambia	14849	23988	38837	0.0236236	0.0381629	0.0617865	628567	938	1578	2516
ZMB	2019	country	1_Afr	Zambia	14902	23578	38480	0.0232805	0.0368343	0.0601147	640107	951	1575	2526
ZWE	2000	country	1_Afr	Zimbabwe	9945	23909	33854	0.0264489	0.0635871	0.090036	376009	665	225	890
ZWE	2001	country	1_Afr	Zimbabwe	10268	23485	33753	0.0270161	0.0617907	0.0888067	380070	687	211	898
ZWE	2002	country	1_Afr	Zimbabwe	10774	23265	34039	0.0279472	0.060349	0.0882962	385513	710	194	904
ZWE	2003	country	1_Afr	Zimbabwe	11372	22948	34320	0.0289878	0.0584957	0.0874835	392302	759	187	946
ZWE	2004	country	1_Afr	Zimbabwe	11982	23355	35337	0.0299296	0.0583384	0.088268	400340	785	184	969
ZWE	2005	country	1_Afr	Zimbabwe	12588	23964	36552	0.0307281	0.0584984	0.0892266	409657	812	199	1011
ZWE	2006	country	1_Afr	Zimbabwe	13224	25116	38340	0.0314592	0.0597487	0.091208	420353	849	223	1072
ZWE	2007	country	1_Afr	Zimbabwe	13858	25557	39415	0.0320741	0.0591511	0.0912252	432062	894	224	1118
ZWE	2008	country	1_Afr	Zimbabwe	14389	25909	40298	0.0323987	0.0583376	0.0907363	444123	922	326	1247
ZWE	2009	country	1_Afr	Zimbabwe	14718	25688	40406	0.0322914	0.0563602	0.0886515	455787	947	426	1373
ZWE	2010	country	1_Afr	Zimbabwe	14781	24771	39552	0.0317268	0.0531697	0.0848965	465883	959	669	1628
ZWE	2011	country	1_Afr	Zimbabwe	14618	22941	37559	0.0308986	0.0484917	0.0793904	473096	942	840	1782
ZWE	2012	country	1_Afr	Zimbabwe	14313	20217	34530	0.0300144	0.0423953	0.0724097	476871	950	1108	2057
ZWE	2013	country	1_Afr	Zimbabwe	13926	17914	31840	0.0291982	0.0375605	0.0667587	476947	924	1148	2072
ZWE	2014	country	1_Afr	Zimbabwe	13494	16879	30373	0.0284959	0.0356438	0.0641397	473542	922	1119	2042
ZWE	2015	country	1_Afr	Zimbabwe	13033	16160	29193	0.0278916	0.0345837	0.0624752	467274	908	988	1896
ZWE	2016	country	1_Afr	Zimbabwe	12550	15001	27551	0.0273335	0.0326719	0.0600054	459143	887	1235	2122
ZWE	2017	country	1_Afr	Zimbabwe	12113	14466	26579	0.0268886	0.032112	0.0590006	450489	865	994	1860
ZWE	2018	country	1_Afr	Zimbabwe	11673	13412	25085	0.0263719	0.0303014	0.0566733	442631	826	1023	1849
ZWE	2019	country	1_Afr	Zimbabwe	11283	12877	24160	0.0258649	0.0295191	0.055384	436228	798	967	1765
